# Macrocyclization of bis-indole quinolines for selective stabilization of G-quadruplex DNA structures†

**DOI:** 10.1039/d0sc03519j

**Published:** 2020-09-16

**Authors:** Rabindra Nath Das, Måns Andréasson, Rajendra Kumar, Erik Chorell

**Affiliations:** Department of Chemistry, Umeå University 90187 Umeå Sweden erik.chorell@umu.se

## Abstract

The recognition of G-quadruplex (G4) DNA structures as important regulatory elements in biological mechanisms, and the connection between G4s and the evolvement of different diseases, has sparked interest in developing small organic molecules targeting G4s. However, such compounds often lack drug-like properties and selectivity. Here, we describe the design and synthesis of a novel class of macrocyclic bis-indole quinolines based on their non-macrocyclic lead compounds. The effects of the macrocyclization on the ability to interact with G4 DNA structures were investigated using biophysical assays and molecular dynamic simulations. Overall, this revealed compounds with potent abilities to interact with and stabilize G4 structures and a clear selectivity for both G4 DNA over dsDNA and for parallel/hybrid G4 topologies, which could be attributed to the macrocyclic structure. Moreover, we obtained knowledge about the structure–activity relationship of importance for the macrocyclic design and how structural modifications could be made to construct improved macrocyclic compounds. Thus, the macrocyclization of G4 ligands can serve as a basis for the optimization of research tools to study G4 biology and potential therapeutics targeting G4-related diseases.

## Abstract 2

Macrocyclization improves the selectivity, affinity, and ability to stabilize G4 DNA structures.

## Graphical abstract

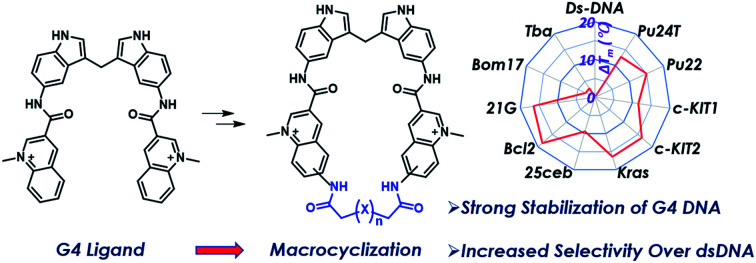

## Introduction

Secondary nucleic acid structures known as G-quadruplexes, discovered in the mid 20^th^ century, have over the last decades received a lot of attention for their established relevance as important biological regulators.^1^ G-quadruplex (G4) structures can form in guanine-rich nucleotide sequences, including both DNA and RNA. G4 structures are formed by π–π stacking of G-quartets which are stabilized by Hoogsteen hydrogen bonds and cation coordination, usually to Na^+^ or K^+^. These secondary structures also display different topologies, such as parallel, anti-parallel, and hybrid arrangements. The type of topology is dependent on the nature of the base sequence, G4-loop (nucleotides connecting the guanines) length, strand orientation, and the type of cations, which all directly correlate to the energetic properties of the G4.^2,3^ In fact, the G4s can be more thermodynamically stable than the DNA-helix and can have rapid folding kinetics.^4^ The formation of DNA G4-structures requires disruption of the helical structure and they are thus believed to be formed during *e.g.* replication, transcription, or negative supercoiling.^5^

Approximately 700 000 nucleotide sequences with potential to form a G4-structure have been identified.^6^ Many of these are evolutionary conserved and display a non-random distribution across the human genome, with increased abundance in sequences such as promoter regions, transcriptional regulators, and telomeric ends.^7–9^ G4 structures are also more common in oncogenes and have found to play a role in telomeric maintenance and the development of diseases, such as neurodegenerative disorders and different types of cancers.^10^ In addition, there is a 90% occurrence rate of potential G4-forming sequences at the DNA replication origins, which together with their stability and rapid folding kinetics suggest that G4s can play a central role in the regulation of replication.^7,11–13^ As an example, the *c-MYC* gene is associated with cell cycle regulation and is upregulated in 70% of all identified cancers.^14^ Expression of the *c-MYC* gene is mostly (∼90%) regulated by the gene promoter region (NHEIII) containing the guanine-rich Pu27 which can fold into multiple G4-structures. The predominant G4 structure in Pu27 is an intramolecular-parallel structure that dominates the mutated sequences Pu22 (deletion of G1-3/G19-20 and G14/G23 to T exchange) and Pu24T (deletion of G1-3 and G10 to T exchange).^15–17^ Ligand-induced stabilization of the G4 in this sequence has shown to prevent the expression of *c-MYC*.^18^ This can offer a novel cancer therapeutic strategy that avoids direct interactions with the MYC protein which has a short half-life and is difficult to target with common ligand–protein interactions.^19^ Another example of G4s associated with prevalent oncogenes is the *c-KIT* and its dysregulation has been reported in the development of several types of cancers and is the main cause of gastrointestinal cancer.^20^ There are two sequences in the *c-KIT* promoter region capable of folding into G4s, the *c-KIT* G4 and, *c-KIT1* G4. The G4s are located upstream of the transcription initiation site, and there is a correlation between the stabilization of these G4-structures and the downregulation of *c-KIT* gene-expression.^21^

Despite extensive studies over the past years, the biological roles of G4s are far from fully explored. Small organic molecules with the ability to selectively bind and stabilize G4s thus represent a powerful tool to gain further insight into G4 biology. Furthermore, such compounds can serve as starting points for further developments towards therapeutics targeting G4-structures. A large pool of ligands has indeed been reported to bind and stabilize G4-structures but reports that describe the further developments of these towards potent and selective compounds with drug-like properties are still scarce. A deeper knowledge of how to advance G4 ligands in terms of what properties make them potent and selective stabilizers of G4s would, therefore, be a valuable addition to the scientific field.^22–24^ Along this line, macrocyclic structures are known to have positive effects on the pharmacokinetics and allow properties beyond Lipinski's rule of 5. It is also attributed that macrocycles can interact selectively with flat and featureless binding sites which is a very important property, considering the non-specific G4 binding surface.^25,26^ Furthermore, a macrocyclic structure should also increase the selectivity for G4 DNA over dsDNA because of its conformational constraints. There are indeed macrocycles reported with good abilities to bind and stabilize G4 DNA, *e.g.* isolated from natural sources.^27,28^ Despite this, the development of novel macrocyclic-based ligands or macrocyclization of known ligands targeting G4s has barely been attempted to date.^29^

**Fig. 1:**
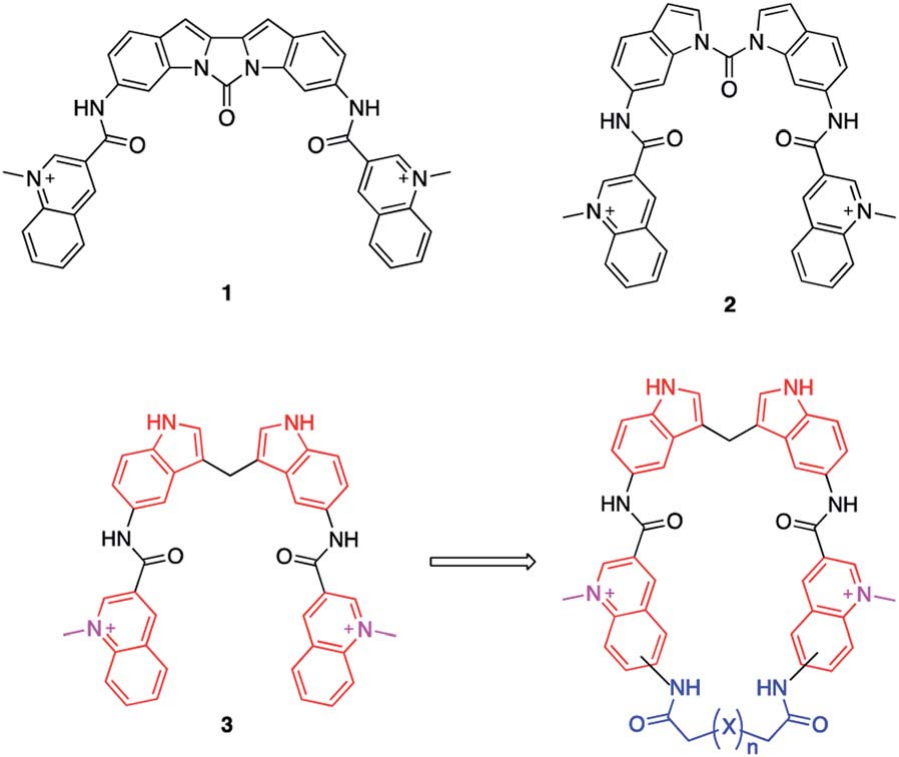
Reported bis-indole compounds (**1–3**) that efficiently bind and stabilize G4 DNA structures. The transition from a compound with linear conformations (**3**) to a semi-rigid macrocyclic compound will lock the compound closer to the bio-active conformation and prevent potential off-target binding to dsDNA and thus improve both binding and selectivity.

Bis-indoles **2** & **3** have displayed excellent abilities to bind and stabilize G4 DNA structures.^30–32^ However, energy minimizations show that this compound class prefers mixed non-planar low energy conformations in contrast to the more planar crescent-shaped bioactive conformation illustrated in Fig. 1. This is believed to result in a higher energetic penalty upon binding to a G4 because it will force the compound out of its most energetically favourable conformation and restrict the number of possible conformations it could occupy. This issue has previously been attempted to be counteracted by locking the structure in a more rigid conformation, as in compound **1**, illustrated in Fig. 1. Unfortunately, this ultimately resulted in a too static compound not optimally accommodating the G4 binding surface which caused a reduction in stabilization as compared to **2**.^31^ This thus constitutes an ideal model system to evaluate if macrocyclization of known G4 ligands can generate improved compounds as this will lock the compounds closer to the bioactive conformation which would reduce the energetic penalty upon binding, but still retain some degree of flexibility (Fig. 1).

**Fig. 2:**
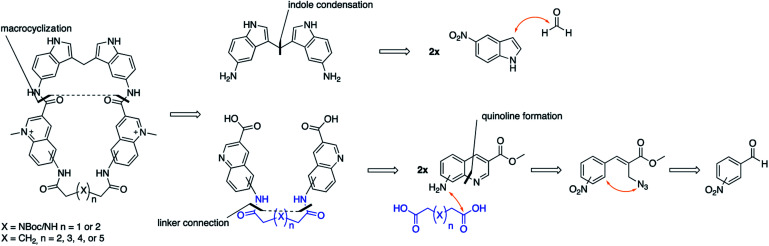
Retrosynthetic outline on how we envisioned the assembly of our macrocyclic compounds.

In this project, we have therefore developed synthetic methods to construct a novel set of macrocyclic bis-indole quinolines with variations in both the ring-size and conformation to investigate the ideal design for optimal stacking on the G4-surface. Additionally, examples with a nitrogen atom in the linker were made with the aim to improve solubility and reach additional interactions with the DNA (Fig. 2). The ability of these novel macrocyclic ligands to bind selectively and stabilize G4-structures was evaluated by using several assays; fluorescence intercalator displacement (FID) assay, fluorescence resonance energy transfer (FRET) melting assay, binding induced fluorescence quenching assay, circular dichroism (CD) spectroscopy, and NMR experiments. The evaluation of the results obtained from the assays was further corroborated with molecular dynamics (MD) simulations. We also expanded the scope of the general concept of macrocyclization using a simple bis-quinoline to both show on the general applicability of the synthetic method and to corroborate the positive effects of macrocyclization on G4 DNA stabilization. From our results, we have identified novel macrocycles that display strong selective binding and stabilization of G4-structures together with important structure–activity relationships which, taken together, show that macrocyclization can indeed be an efficient strategy to optimize G4 ligands.

## Results & discussion

### Synthesis of macrocycles

The novel macrocyclic compounds were planned to be constructed *via* the convergent pathway depicted in Fig. 2. The macrocyclization was proposed to be achieved *via* the combination of the bis-indole amine and the bis-quinoline acids *via* an initial amide-coupling followed by a theoretically more favourable intramolecular amide-cyclization as compared to a second intermolecular amide-coupling. Synthesis of the bis-indole has previously been reported by the condensation of two indoles with an aldehyde.^33^ Generation of the bis-quinoline acids were proposed to be achieved with a tandem amide coupling of two quinoline amines with a di-acid linker. This assembly was considered attractive since it would easily allow variations, both in the amine position on the quinoline and in the length and composition of the linker prior to the crucial macrocyclization step. Finally, our quinolines were proposed to be made from the azide precursors *via* a light-mediated radical intramolecular cyclization reaction.

**Fig. 3:**
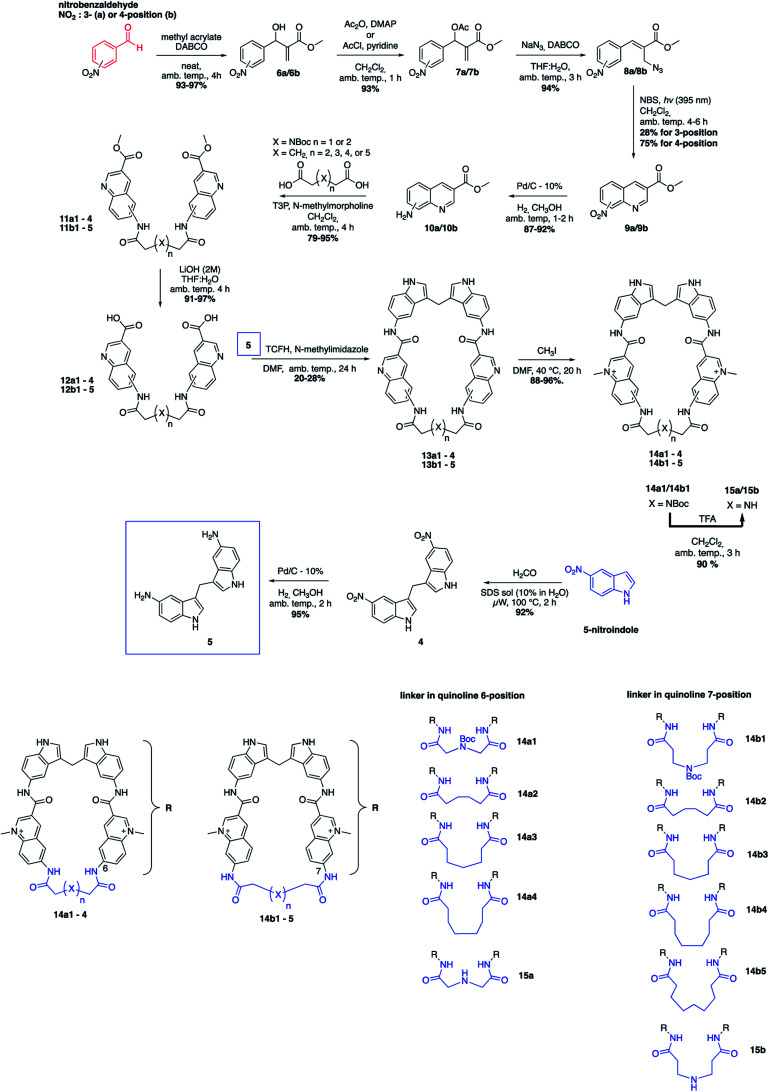
Total synthetic scheme for the macrocyclic compounds and a summary of the **11** different macrocycles.

The bis-indole amine **5** was synthesized as planned from 5-nitroindole by condensation with formaldehyde at elevated temperature followed by subsequent nitro reduction to the di-amine **5** with hydrogen in the presence Pd/C-10% (Fig. 3). The synthesis of the azide precursors **8a/b** for the quinoline formation was initiated from nitro benzaldehyde, with the nitro-group either in the *meta*- or *para*-position, through a Baylis–Hillman reaction to give **6a/b** followed by acetylation of the newly formed alcohol to generate **7a/b**. The acetylated compounds were treated with NaN_3_ followed by a light-mediated radical promoted cyclization of **8a/b** in the presence of NBS (*N*-bromosuccinimide) to give the quinoline products **9a/b**. The concentration for this reaction proved to be critical and when performed at 0.2 M, mostly intermolecular radical side reactions took place resulting in a quinoline yield of only 15–20%. However, when the reaction was performed at a lower concentration (∼20 mM) a significant improvement of the yield was observed (75–87%). Several radical initiators were also investigated; NCS (*N*-chlorosuccinimide), NIS (*N*-iodosuccinimide), AIBN (azobisisobutyronitrile), TBHP (*t*-butyl hydroperoxide) and DBPO (benzoyl peroxide), but none of them gave any improvement in the yield. In the case of 3-nitro substituted (**8a**), the cyclization can occur either in *ortho*- or *para*-position to the nitro-group and unfortunately the unwanted *ortho*-cyclized derivative was always obtained as the major product (59% yield) and the para-cyclized derivative as the minor product (28% yield) likely because of steric effects and electronic directions by the nitro-group. The nitro-group in **9a/b** was next reduced to the amines **10a/b** (87–92% yield) through palladium catalysed hydrogenation. The amide-coupling between the different di-acid linkers and the quinoline amines **10a/b** was attempted with several different coupling agents, including PyBOP (benzotriazol-1-yl-oxytripyrrolidinophosphonium hexafluorophosphate), PyAOP ((7-azabenzotriazol-1-yloxy)tripyrrolidinophosphonium hexafluorophosphate), EDC·HCl (*N*-(3-dimethylaminopropyl)-*N*′-ethylcarbodiimide hydrochloride) and TFFH (fluoro-*N*,*N*,*N*′,*N*′-tetramethylformamidinium hexafluorophosphate), but none of these conditions gave satisfactory results.

However, propanephosphonic acid anhydride (T3P) has been used successfully in the coupling of acids with poorly nucleophilic amines without undesired side reactions between the amine and the coupling reagent.^34^ Fortunately, the amide-coupling between the different di-acid linkers and the quinoline amines proceeded smoothly with T3P as a coupling reagent and *N*-methylmorpholine as the base in CH_2_Cl_2_ at ambient temperature to afford the different bis-quinoline linkers **11** in satisfactory yields (79–95%) without the need for chromatographic purification. Subsequent saponification of the ethyl ester afforded bis-quinoline acids **12** in high yields (Fig. 3).

Our hypothesis for the key macrocyclization reaction between di-amine **5** and di-acids **12** was that a first intermolecular amide-coupling would occur and that an intramolecular cyclization then would be faster and more favourable compared to another intermolecular amide-coupling that would result in unwanted polymer/dimer side-products. The reaction was first attempted with the same conditions as for the quinoline linker-coupling from **10** to **11**, with DMF as the solvent instead of CH_2_Cl_2_. Upon monitoring the reaction, the desired product was formed in very small amounts and after extensive condition-screening we only obtained the macrocycles in very low yields (<5%), giving a dimeric product in excess. We instead performed the reaction at high dilutions (2–6 mM) with several known amide-coupling reagents, hoping to avoid di-amine side-reactions and reduce dimer-formation. The change in conditions, run at both elevated temperature and ambient temperature, gave slight improvements in yield. We then utilized TCFH (*N*,*N*,*N*′,*N*′-tetramethylchloroformamidinium hexafluorophosphate) as activating reagent and *N*-methylimidazole as the base^35^ in DMF and ran the reaction with a dilution of 2 mM. This afforded the macrocycles in yields between 20–28%, which we deemed to be acceptable. Furthermore, this reaction setup also offered us easier purifications by column chromatography. Subsequent methylations of the quinolines were performed at 40 °C using methyl iodide (CH_3_I) and afforded the different methylated macrocycles (**14**) in near quantitative yields. In the case of the NBoc analogues (**14a1** and **14b1**) the nitrogen was deprotected in the presence of 10% TFA in CH_2_Cl_2_ at ambient temperature. LC-MS and HRMS analysis suggested the formation of the desired macrocycles (**15a**/**15b**) and although we were not able to obtain clear NMR spectra to fully characterize these compounds, we thus still decided to include them in the evaluation of the compounds' abilities to stabilize G4 DNA (Fig. 3).

### FRET melting assay

**Fig. 4:**
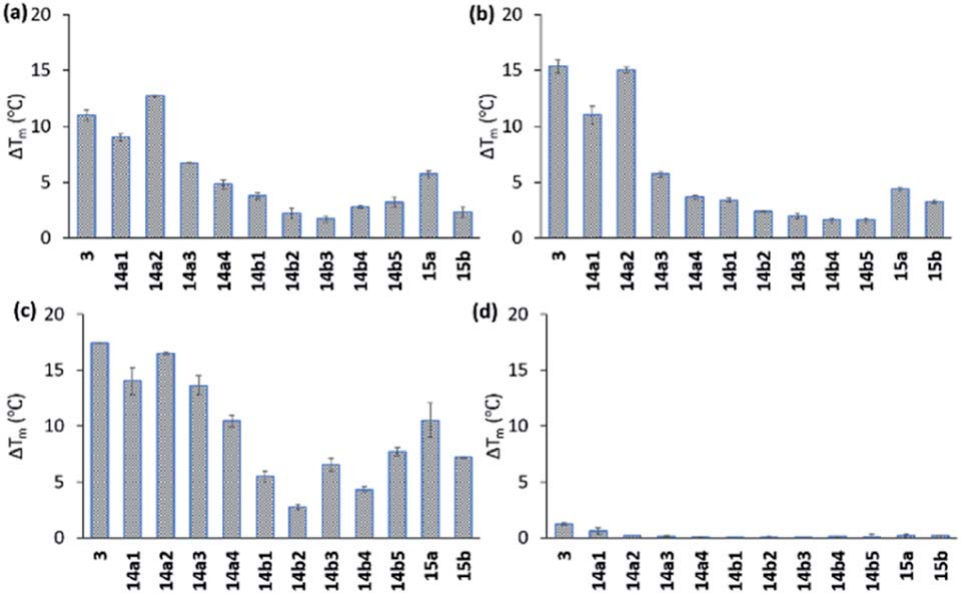
Stabilizing ability of the ligands by FRET melting assay at 2 μM of concentration for (a) Pu24T G4 DNA, (b) Pu22 G4 DNA, (c) *c-KIT2* G4 DNA, and (d) double standard DNA. DNA concentration is 0.2 μM. *T*_m_ in absence of ligands of Pu24T G4 DNA is 62.5 ± 0.3 °C, Pu22 G4 DNA is 64.3 ± 0.2 °C, *c-KIT2* G4 DNA is 63.9 ± 0.1 °C and ds DNA is 68.0 ± 0.2 °C.

All compounds displayed good solubility properties and were initially screened in a FRET melting assay. In this assay, we measure the differences in the melting temperature (Δ*T*_m_) of fluorescently labelled G4 DNA (*c-MYC* Pu22, *c-MYC* Pu24T, and *c-KIT2*, for sequences see Table S1†) in the presence and absence of macrocycles **14a1–4** and **14b1–5** (Fig. 4) to investigate their abilities to stabilize G4 structures at different concentrations (1, 2, and 5 μM) using the most efficient bis-indole quinoline reported (**3**) as a reference (Fig. 4a–c and S1–S3†).

All the compounds were able to stabilize both of the two parallel *c-MYC* and the parallel *c-KIT2* G4 structures. Macrocycles **14a1**, **14a2**, and **14a3**, with the linker in position 6 on the quinoline, displayed highly efficient stabilization effects with a Δ*T*_m_ of almost 20 °C (at 5 μM of **14a2**, see Fig. 4a–c and S1 and S2†), thus being comparable to the strongly stabilizing reference compound **3**. The macrocycles **14b1–14b5**, with the linker in position 7 on the quinoline, show a large drop in their activity compared to macrocycles **14a1**, **14a2** and **14a3** with the linker in position 6 on the quinoline. A clear trend in the size of the macrocycles with the linker in position 6 (**14a1–4**) was observed where the smaller macrocycles more effectively stabilized the G4 structures. The same trend could not be seen for the macrocycles with the linker in position 7 (**14b1–5**). The macrocycles with an aliphatic amine in the linker (**15a**) had an apparent reduced stabilization potential compared to the boc-protected version (**14a1**). This might be due to solvent penalties for the amine upon binding to the G4 surface or by breaking up internal hydrogen bonding. However, since the purity and characterization of **15a** was not confirmed, we cannot fully validate these results. More importantly, FRET melting experiments to investigate possible interactions with double-stranded DNA (ds26) were also performed, and while compound **3** showed a Δ*T*_m_ of around 8 °C at 5 μM the macrocyclic compounds (except for **14a1**) had a very small or unmeasurable effect on the stabilization of dsDNA (Fig. 4d and S3†). This supports our hypothesis that the macrocyclic design increases the selectivity by preventing conformations that can interact with dsDNA.

To confirm that the compounds do not influence the topological change of G4-DNA, we measured CD spectra in the absence and presence of the compounds. No major changes were observed which confirms that the conformation of the G4 structures remained intact in the presence of the macrocycles (Fig. S4†). CD melting studies were also performed for G4 DNA with **14a1** and **14a2** and 3–18 °C stabilization was observed (Table S2 and Fig. S5†).

### FID and fluorescence quenching assays

**Fig. 5:**
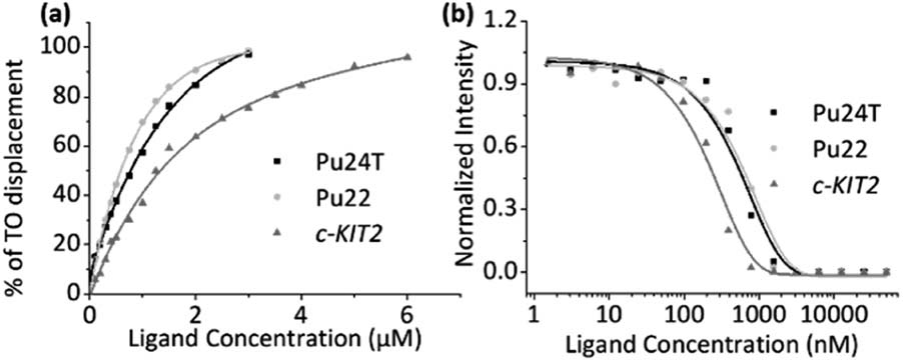
(a) FID based binding affinity plot induced by the ligand **14a2** for Pu24T, Pu22 and *c-KIT2* G4-DNA. (b) Fluorescence quenching based binding affinity plot of **14a2** for Pu24T, Pu22, and *c-KIT2* G4-DNA.

To investigate the binding affinities of the macrocyclic ligands we performed a Fluorescence Intercalator Displacement (FID) assay. The FID assay was performed with a selection of the most interesting macrocycles from the first FRET screen (**14a2**, **14a3**, **14b3**, and **14b5**). In the FID assay, we investigate the ability of the macrocycles to displace the well-known G4 binder thiazole orange (TO) upon binding to the G4 DNAs (*c-MYC* Pu22, *c-MYC* Pu24T, and *c-KIT2*), which can be measured by a decrease in fluorescence (Fig. 5a and S6–S10†). The results (see Table 1) follow the same trend as for the FRET assay, **14a2** and **14a3** have the lowest *K*_d_ of the tested macrocycles with **14a2** as the best compound with binding constants of 0.99 μM, 0.23 μM, and 0.44 μM for Pu24T, Pu22, and *c-KIT2* G4 DNA respectively. The results underscore the importance of the macrocyclic ring-size and the position of the macrocyclic linker position on the quinoline for an optimal binding to the G4 surface. FID assay was further employed for dsDNA with the best macrocycle **14a2** and reference compound **3** to compare their ability to displace TO from dsDNA. This shows that both **14a2** and **3** can displace TO at higher concentrations which is not surprising considering that the compounds are positively charged and are therefore likely to interact in an unspecific manner with the negatively charged DNA in this *in vitro* setting. Importantly, macrocyclization of **3** to form **14a2** reduce this unspecific interaction (^ds^DC_50_ (**3**) = 1.34 μM; ^ds^DC_50_ (**14a2**) = 2.40 μM) (Fig. S11a and S12†). We have also investigated the compounds ability to stabilize dsDNA using CD (Fig. S11b†). This shows that **14a2** does not affect dsDNA whereas compound **3** does, in fact, affect dsDNA stability which again underscores the value of macrocyclization to achieve selectivity.

**Table 1:** Summary of the apparent *K*_d_-values obtained from the FID assay and *K*_d_-values obtained from the MST assay with the different G4 DNA (*c-MYC* Pu24T, *c-MYC* Pu22, and *c-KIT2*) for the macrocycles (**14a2**, **14a3**, **14b3**, and **14b5**)

Macrocycle	*K*_d_ (μM) for Pu24T^a^	*K*_d_ (μM) for Pu22^a^	*K*_d_ (μM) for *c-KIT2*^a^	*K*_d_ (μM) for Pu24T^b^	*K*_d_ (μM) for Pu22^b^	*K*_d_ (μM) for *c-KIT2*^b^
**14a2**	0.99 ± 0.02	0.23 ± 0.01	0.44 ± 0.02	0.77 ± 0.09	0.90 ± 0.11	0.31 ± 0.03
**14a3**	1.25 ± 0.04	0.33 ± 0.02	0.54 ± 0.06	1.32 ± 0.24	1.09 ± 0.10	0.35 ± 0.04
**14b3**	2.31 ± 0.06	0.60 ± 0.04	0.93 ± 0.04	1.54 ± 0.21	0.74 ± 0.09	0.62 ± 0.08
**14b5**	1.80 ± 0.03	0.68 ± 0.05	1.22 ± 0.08	1.39 ± 0.20	3.56 ± 0.45	Nd

a Using the FID assay (see also Fig. 5a and S6–S10).

b Using fluorescence quenching assay (see also Fig. 5b and S13). ‘nd’ indicates not determined.

To confirm the binding affinities obtained from the FID assay, we also employed a ligand induced fluorescence-quenching assay with a 5′-Cy5-labelled G4 DNA (*c-MYC* Pu22, *c-MYC* Pu24T, and *c-KIT2*) by using Microscale Thermophoresis (MST). The results from the binding induced fluorescence quenching assays corroborate the FID data with a very similar internal trend in the binding affinities for the different macrocycles (Fig. 5b and S13†). Once again, **14a2** proved to be the better overall binder compared to the other macrocycles. The binding constants (*K*_d_) obtained from the FID and MST assays are summarized in Table 1.

### ^1^H NMR titrations

**Fig. 6:**
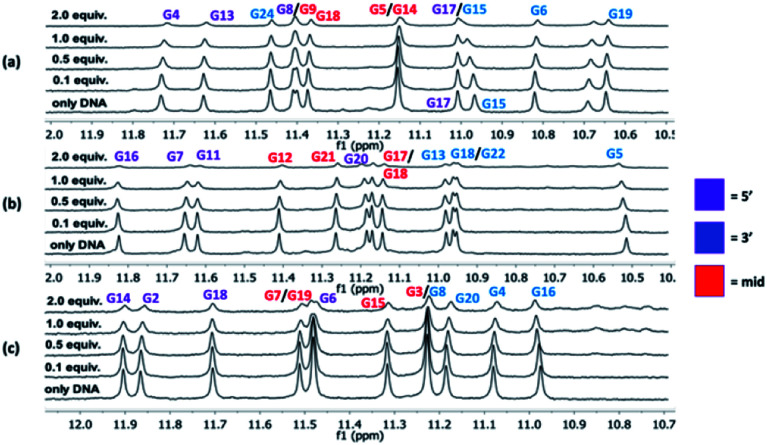
^1^H NMR (850 MHz) titrations for *c-MYC* Pu24T (a), *c-MYC* Pu22 (b), and *c-KIT2* (c) with **14a2**. The initial DNA concentration was 90 μM and macrocycle was then added so the last addition corresponded to a total molar ratio of G4 DNA : macrocycle 1 : 2.

NMR experiments were next utilized to probe both the macrocycles' abilities to bind the G4 structures (*c-MYC* Pu22, *c-MYC* Pu24T, and *c-KIT2*) and to learn more about these interactions. In these experiments, the G4-DNA was titrated with macrocycle spanning a ratio from 1 : 0.1 to 1 : 2 (DNA : macrocycle) and the changes in the imino protons for the hydrogen bonding guanines in the G-tetrads in the NMR spectrum were compared at the different concentration ratios. No significant changes in the chemical shift for the imino protons were observed upon binding of the macrocycles. However, an evident broadening was instead seen, and upon the addition of 2 equivalents of macrocycle **14a2**, the intensities of the peaks from the imino protons in the c-MYC G4s are strongly reduced (Fig. 6 and S14–S16†).

The broadening of the imino proton signals is most likely due to an on–off rate in the interaction between the DNA and macrocycle on a timescale that yields line-broadening due to chemical exchange. A clear broadening of the imino protons was also observed for **14a3** and **14b3** in all three G4 structures, the effect was stronger with **14b3** indicating a difference in their binding interactions (Fig. S15 and S16†). The appearance of new peaks was noticed (*e.g.* when increased amounts of **14a2** were added to the *c-KIT2* G4 DNA) which indicates a slower exchange (lower on–off rate) where both the free and bound form of DNA are observed which indicate a stronger binding.

### Competition assay and selectivity

In the FRET screening assay, we concluded that the macrocycles had very little or no effect on dsDNA and to further confirm these results a FRET melting competition assay was conducted.

**Fig. 7:**
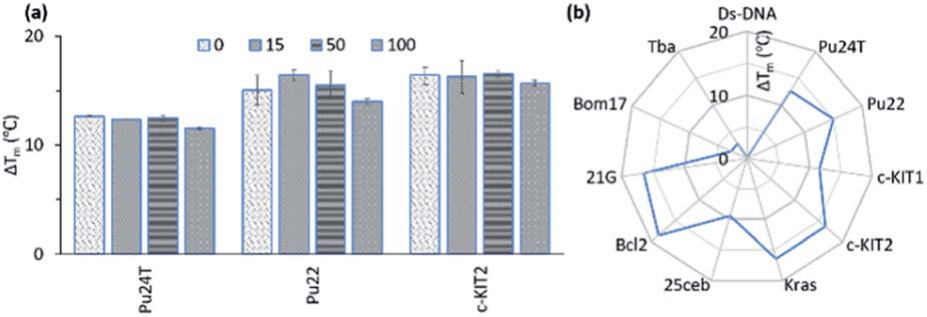
(a) Stabilization ability of **14a2** (2 μM) for Pu24T, Pu22 and *c-KIT2* G4-DNA (0.2 μM) in presence of excess amount (0–100 equivalent) of a double-stranded competitor dsDNA (ds26). (b) Selectivity study of **14a2** (2 μM) for their ability to stabilize of Ds-DNA (0.2 μM) and different G4 DNA (0.2 μM) (see also Fig. S14 and S15†). Pu24T, Pu22 (*c-MYC* promoter); *c-KIt1*, *c-Kit2* (*c-KIT* promoter); *Kras* (*K-RAS* gene) and 25ceb (human minisatellite) are parallel G4 forming sequences. *Bcl-2* (*BCL-2* promoter) and 21g (human telomere) are hybrid G4 forming sequences. Bom17 (*Bombyx* telomere) and TBA (thrombin binding aptamer) are antiparallel G4 forming sequences.

The thermal stability was first measured only for the respective G4-DNA with **14a2** and compound **3** (0.2 μM DNA : 2 μM **14a2/3**) and dsDNA were sequentially added to investigate if any major changes in the thermal stability were observed. In the presence of a large excess of dsDNA (20 μM, 100 equivalents compared to G4 DNA), no major changes in the thermal stabilization for the G4 DNA were observed in case of **14a2**, but for compound **3** the stabilization (Δ*T*_m_) decreases by 2–4 °C. This show that macrocycle **14a2** has a strong preference for binding G4 DNA over dsDNA (Fig. 7a and S17†) and confirm that macrocyclization also increases the selectivity. We next investigated if the macrocycles display selectivity over different G4 topologies by measuring their ability to stabilize different G4 structures using FRET. This shows that **14a2** strongly stabilizes parallel and hybrid G4 structures whilst completely discriminating against the anti-parallel structures (Fig. 7b and S18, and S19†). This discrimination could not be seen with the open-chain analogue **3** which also stabilizes the anti-parallel structures.^28^ This difference in selectivity could thus be attributed to the macrocyclic design of the compounds. The other macrocyclic compounds also showed the same strong selectivity for G4 DNA over dsDNA and also the same selectivity trend between different types of G4 DNA structures, which support the value of the macrocyclic design in the observed selectivity.

### Computational studies

**Fig. 8:**
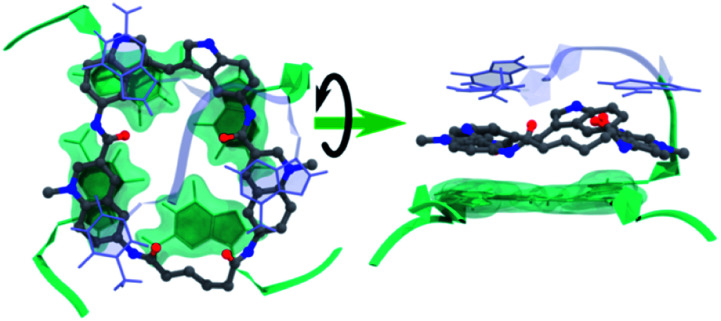
Representative binding pose of **14a2** with Pu24T *c-MYC* G4 DNA during MD simulations. The 5′-G-tetrad (green), nucleotides flanking the G4 DNA structure (light blue), and compounds (ball-stick model) from the largest cluster's central structure.

Molecular dynamics simulations were next performed to study the details of the interaction between macrocycle **14a2** and G4 DNA. Based on previous studies of the reference compound **3**, we expect the macrocycles to bind by end-stacking to the terminal G-tetrads. The Pu22 *c-MYC* G4 has both G-tetrads accessible for binding and the macrocycles thus likely bind at both the 3′- and the 5′-G-tetrads. However, the Pu24T *c-MYC* G4 has an accessible 5′-G-tetrad and a partly blocked 3′-G-tetrad. We thus modelled **14a2** on the top of the 5′-G-tetrad of the Pu24T *c-MYC* G4 structure and performed molecular dynamics simulations for a total of 1 μs. This showed that **14a2** efficiently stack on the top G-tetrad and is further sandwiched by strong interactions with the nucleotides flanking the G-tetrad (Fig. 8, Table S4 and Fig. S20†). From the simulations, it becomes evident that increasing the linker length in macrocycle **14a2** will be detrimental to the ability to efficiently stack on the top G-tetrad as it would partly push one of the quinolines out of the G-tetrad. This is in well agreement with the experimentally determined structure–activity relationships for the macrocycles. Furthermore, this model can also explain the reduced activity observed by the macrocycles linked in position 7 on the quinolines as the planar conformation required to efficiently stack on the G-tetrad would be energetically disfavoured in this design. These macrocycles would also be less dependent on the linker length, which also is in good agreement with the observed results for the **14b** macrocycles linked in position 7 of the quinolines.

The MD simulations also show that the size of the macrocycle may not be optimal for occupying the G4-surface. With the present design, only 3 of the 4 aromatic systems seem to be able to partake in stacking interactions with the G-tetrad. This suggests that further improvements of the macrocyclic design should be possible by adjusting the ring-size and the number of aromatic systems to achieve a more optimal size and an even further improved stacking interaction with the G4-surface.

Next, we calculated and compared the drug-like descriptors (molecular weight, log *P*, hydrogen bond-donor, hydrogen bond-acceptor, and the total polar surface area (TPSA)) between the macrocycles and compound **3** (Table S3†). This show that the smaller macrocycles have a lower log *P* and a dramatically increased TPSA in comparison to **3** despite their increased molecular weight. Furthermore, we notice that the lowest energy conformations of the unbound form of the macrocycles can shield functionalities through intramolecular hydrogen bonds, non-classical hydrogen bonds, and π–π interactions (Fig. S20–S23†), which can have a positive impact on drug-like properties such as cellular uptake.^25^ This again underscores the potential of macrocyclization to improve drug-like properties.

### General applicability of the strategy

**Fig. 9:**
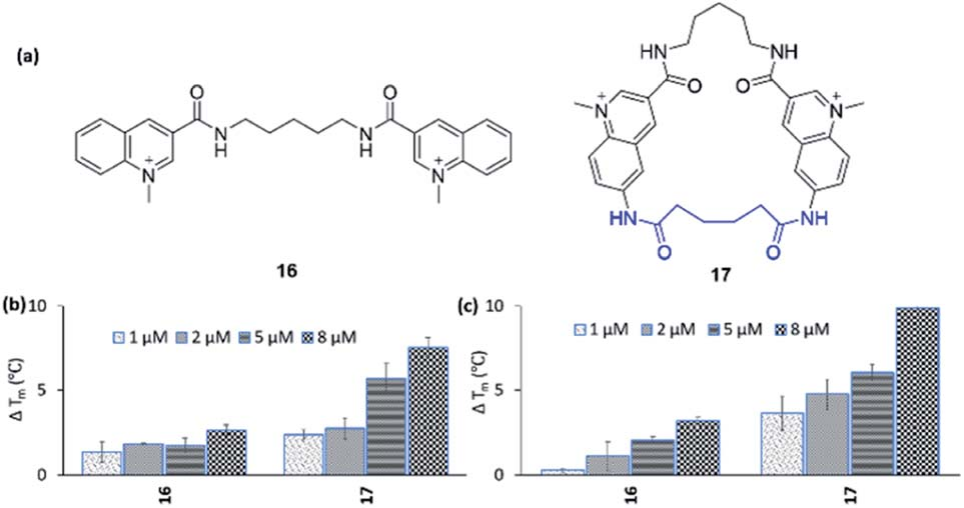
(a) Bis-quinoline **16** and a macrocyclic analogue **17**. Stabilizing ability of the ligands by FRET melting assay at 1, 2, 5, and 8 μM concentrations for (b) Pu24T G4 DNA, (c) Pu22 G4 DNA. DNA concentration is 0.2 μM. *T*_m_ in absence of ligands of Pu24T G4 DNA is 62.5 ± 0.3 °C and Pu22 G4 DNA is 64.3 ± 0.2 °C.

To expand the scope of this study, both in terms of the general applicability of the synthetic strategy and the macrocyclization strategy to improve G4 binding and stabilization, we employed our strategy on the simple bis-quinoline **16** to afford a macrocyclic counterpart **17** (Fig. 9a). The two compounds' abilities to stabilize G4 DNA (*c-MYC* Pu24T and *c-MYC* Pu22) were evaluated by the FRET melting assay. This show that macrocyclization of **16** clearly increased the ability to stabilize G4 DNA (at 8 μM **16**: Δ*T*_m_ = 2.6 °C (Pu24T) and Δ*T*_m_ = 3.2 °C (Pu22), **17**: Δ*T*_m_ = 7.5 °C (Pu24T) and Δ*T*_m_ = 9.9 °C (Pu22)) (Fig. 9b and c). Furthermore, none of **16** and **17** were able to stabilize dsDNA, and macrocycle **17** also displayed great selectivity for G4 DNA over dsDNA (Fig. S21 and S22†). This thus emphasizes the value of macrocyclization as a design element in the optimization of G4 interacting ligands in general and for the frequently appearing bis-quinoline derivatives in particular.

## Conclusions

We have used bis-indole quinoline G4 ligands as a model system to evaluate the potential of macrocyclization in the optimization of G4 ligands in terms of G4 binding and stabilization properties. Key to succeed with this was substantial synthetic developments for the construction of the novel macrocyclic compounds with variations in both the amide-linker length and the quinoline-amine position. All the synthesized target macrocycles were first evaluated by a FRET melting assay and three different G4 structures which identified highly efficient G4 stabilizing compounds. Their stabilizing abilities were confirmed using CD melting and four highly promising derivatives **14a2**, **14a3**, **14b3**, and **14b5** were selected for further studies. NMR, MST, and FID assays with the same three G4 DNA structures showed that these derivatives also strongly bind G4 DNA with *K*_d_-values in low micromolar or even sub-micromolar (0.2–2.3 μM) concentrations. Importantly, the macrocycles did not affect the overall structure of the G4s, which is of key importance for their use as research tools in native studies of G4 DNA. In line with the hypothesis, the macrocyclic design also proved to generate improved selectivity for G4 DNA over dsDNA compared to the open-chain analogue. Also, the macrocycles were selective for parallel and hybrid G4 structures over anti-parallel G4 DNA and this ability to discriminate between G4 topologies could be directly related to the macrocyclic design of the compounds. Further correlations between the macrocyclic structure and the assay data revealed that the linker position on the quinoline is crucial for strong G4 interactions with position 6 being preferred over position 7. Furthermore, the linker length/ring size is also essential for the effect of the macrocycles linked in position 6 on the quinoline. To investigate the structural basis for these observed structure–activity relationships, MD simulations based on the NMR data were performed. This clarified the reason behind the observed SAR with the ring size and linker position being of key importance to keep the macrocycle aligned for efficient end-stacking on the top G-tetrad. In addition, the MD simulations also suggest that it should be possible to construct macrocyclic compounds with even further improved affinity, selectivity, and stabilization for G4 structures. Finally, we applied our strategy on a simpler bis-quinoline derivative which resulted in increased G4 stabilization with great G4 selectivity which confirmed the potential of macrocyclization.

Taken together, we have shown that macrocyclization is a valuable design-element for the advancement of ligands that target G4 DNA structures, with the potential for improved drug-like properties, G4 stabilization, selectivity, and affinity. We show that this strategy can be adopted on various G4 ligands with the aim to advance them towards research tools, diagnostic tools, and as therapeutics for the treatment of G4-related diseases.

## Conflicts of interest

There are no conflicts to declare.

## Supplementary Material

SC-011-D0SC03519J-s001
