# Supplementary material for: Macrocyclization of bis-indole quinolines for selective stabilization of G-quadruplex DNA structures
Source: Chem Sci. 2020 Sep 16;11(38):10529–37. doi: 10.1039/d0sc03519j (PMC8162405; doi:10.1039/d0sc03519j)
Supplement: SC-011-D0SC03519J-s001 [file SC-011-D0SC03519J-s001.pdf]

Electronic Supplementary Material (ESI) for Chemical Science.
This journal is © The Royal Society of Chemistry 2020

The Macrocyclization of Bis-Indole Quinolines for Selective Stabilization of G-quadruplex DNA Structures

Table of contents

Table of contents.....2
Table S1.....4
Folding of G4 structures for FRET study.....5
FRET melting assay.....5
Figure S1.....5
Figure S2.....6
Figure S3.....6
Circular dichroism (CD) Spectra.....7
Figure S4.....7
Table S2.....7
Figure S5.....7
Fluorescence Intercalator Displacement Experiments.....7
Micro Scale Thermophoresis (MST).....8
Figure S6.....8
Figure S7.....9
Figure S8.....9
Figure S9.....9
Figure S11.....10
Figure S12.....10
Figure S13.....10
Nuclear Magnetic Resonance (NMR) Titrations.....11
^{1}H NMR experiments with Ligand and DNA.....11
Figure S14.....11
Figure S15.....12
Figure S16.....12
Figure S17.....12
Figure S18.....13
Figure S19.....13
G4 complex modeling.....13
Table S3.....14
Figure S20.....14
Figure S21.....15
Figure S22.....15
Figure S23.....16

Molecular dynamics simulations 16
Table S4 16
Figure S24 17
Figure S25 17
Figure S26 17
General Experimental 18
General Procedure 1: Quinoline and Linker Amide Coupling 18
General Procedure 2: Bis-Quinoline-Ester Hydrolysis 18
General Procedure 3: Macrocyclization 18
General Procedure 4: Methylation of Macrocycle 19
NMR spectra of Synthesized Molecules 48
References 100

Table S1. Oligonucleotides used in this study.

**Sequence name**	**Sequence (5'-3')**	**Technic**	**Function**
FPu24TT	Fam-TGAG$_3$TG$_2$TGAG$_3$TG$_4$A$_2$G$_2$-Tamra	FRET	Labelled G4 (DNA)
FPu22T	Fam- TGAG$_3$TG$_3$TAG$_3$TG$_3$TA$_2$ -Tamra	FRET melting	Labelled G4 (DNA)
Fc-KIT1T	Fam-G$_3$AG$_3$CGCTG$_3$AG$_2$AG$_3$-Tamra	FRET melting	Labelled G4 (DNA)
Fc-KIT2T	Fam-G$_3$CG$_3$CGCGAG$_3$AG$_4$-Tamra	FRET melting	Labelled G4 (DNA)
FKrasT	Fam-AG$_3$CG$_2$TGTG$_3$AATAG$_3$AA-Tamra	FRET melting	Labelled G4 (DNA)
F25cebT	Fam- AG$_3$TG$_3$TGTAAGTGTG$_3$TG$_3$T –Tamra	FRET melting	Labelled G4 (DNA)
Fbom17T	Fam- G$_2$TTAG$_2$TTAG$_2$TTG$_2$ -Tamra	FRET melting	Labelled G4 (DNA)
FBcl2T	Fam-G$_3$CGCG$_3$AG$_2$AATTG$_3$CG$_3$-Tamra	FRET melting	Labelled G4 (DNA)
F21GT	Fam- G$_3$TTAG$_3$TTAG$_3$TTAG$_3$ -Tamra	FRET melting	Labelled G4 (DNA)
FtbaT	Fam- G$_2$TTG$_2$TGT G$_2$TTG-Tamra	FRET melting	Labelled G4 (DNA)
Fds26T	Fam-CAATCGGATCGAATTCGATCCGATTG-Tamra	FRET melting	Labelled G4 (DNA)
ds26	CAATCGGATCGAATTCGATCCGATTG	FRET melting	Competitor (DNA)
Pu24T	TGAG$_3$TG$_2$TGAG$_3$TG$_4$A$_2$G$_2$	FID	G4 DNA
Pu22	TGAG$_3$TG$_3$TAG$_3$TG$_3$TA$_2$	FID	G4 DNA
Ckit2	G$_3$CG$_3$CGCGAG$_3$AG$_4$	FID	G4 DNA
5cy5-Pu24T	Cy5-TGAG$_3$TG$_2$TGAG$_3$TG$_4$A$_2$G$_2$	MST	Labelled G4 (DNA)
5cy5-Pu22	Cy5-TGAG$_3$TG$_3$TAG$_3$TG$_3$TA$_2$	MST	Labelled G4 (DNA)
5cy5-*c-KIT2*	Cy5-G$_3$CG$_3$CGCGAG$_3$AG	MST	Labelled G4 (DNA)

Folding of G4 structures for FRET study

Synthetic labelled oligonucleotides for FRET study were purchased from Eurofins Genomics. Stock solutions were prepared in water at 100 µM concentration. The sequences used are listed in supplementary Table S1. All the oligonucleotides except Pu22 were prefolded in 10 mM lithium cacodylate buffer (pH 7.4), with 10 mM KCl and 90 mM LiCl by heating for 5 min at 95 °C and then quick cooling on ice. Pu22 was folded in 10 mM lithium cacodylate buffer (pH 7.4), with 2 mM KCl and 98 mM LiCl.

FRET melting assay

The fluorescence resonance energy transfer (FRET) occurs between two dyes (5'-FAM as donor and 3'- TAMRA as acceptor) linked at both extremities of a DNA oligonucleotide. When the oligonucleotides are folded into G4 structures, the donor and acceptor are in close proximity, which results in an energy transfer from the donor to the acceptor. This process can be detected by a reduction in the fluorescence emission of the donor. Fluorescence emission of the donor is recovered when the temperature increment triggers the thermal denaturation of the G4 structure. The experiments were performed in a Bio-rad CFX96 real-time PCR device at temperatures from 10 to 95 °C at 1.5 °C/m heating rate using a 492-nm excitation wavelength and a 516-nm detection wavelength in 96-well plates. Each condition was tested in duplicate and analysis of the data was carried out by using Excel and Origin 8 software. In each well, 0.2 µM of labelled oligonucleotide was heated in the presence or absence of the ligand (and with or without the competitor dsDNA) at the specified concentrations. Emission of 5'-FAM was normalized between 0 and 1, and the melting temperature (T_m) is defined as the temperature at which 50% of the G4 structures are denatured (the temperature when the normalized emission was 0.5). The stabilization (ΔT_m) is calculated from comparison of T_m of the fluorescently labelled oligonucleotide in the presence or absence of the ligand.

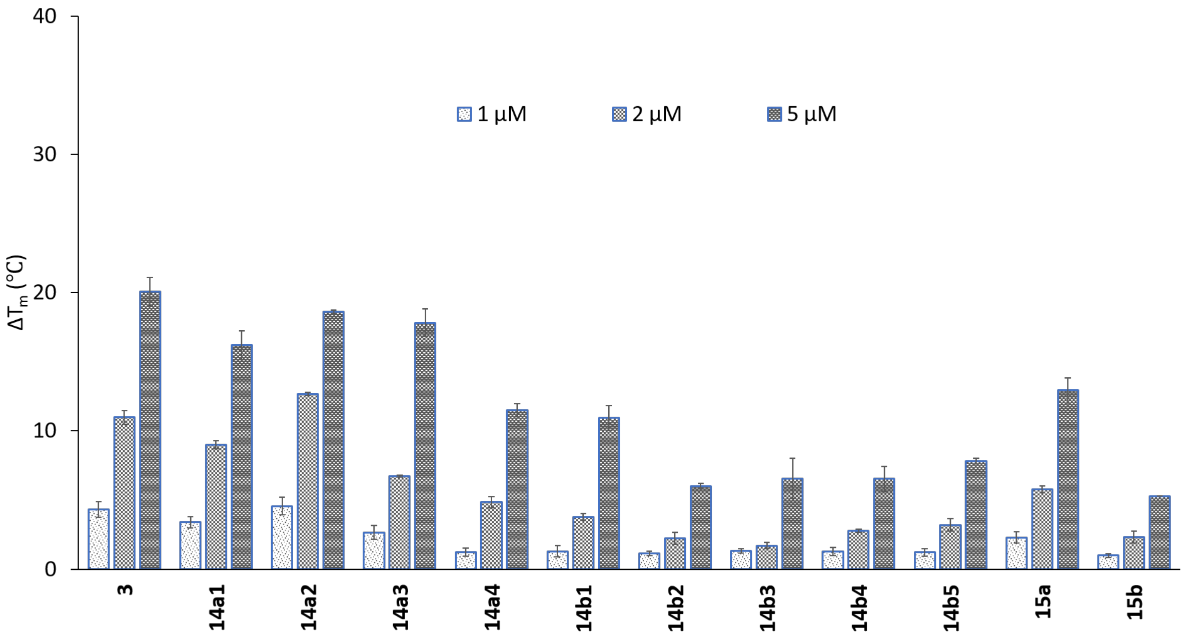

Figure S1. FRET melting assay of the 9 macrocycles with reference compound **3**. Experiments were performed with 0.2 µM FPu24TT, 1, 2, or 5 µM ligand in 10 mM lithium cacodylate buffer (pH 7.2), 90 mM LiCl, and 10 mM KCl. T_m in absence of ligands of FPu24TT was 62.7 ± 0.3 °C, and y-axis shows ΔT_m that corresponds to the T_m change in the absence and presence of ligand. Error bars correspond to SD of at least three independent experiments.

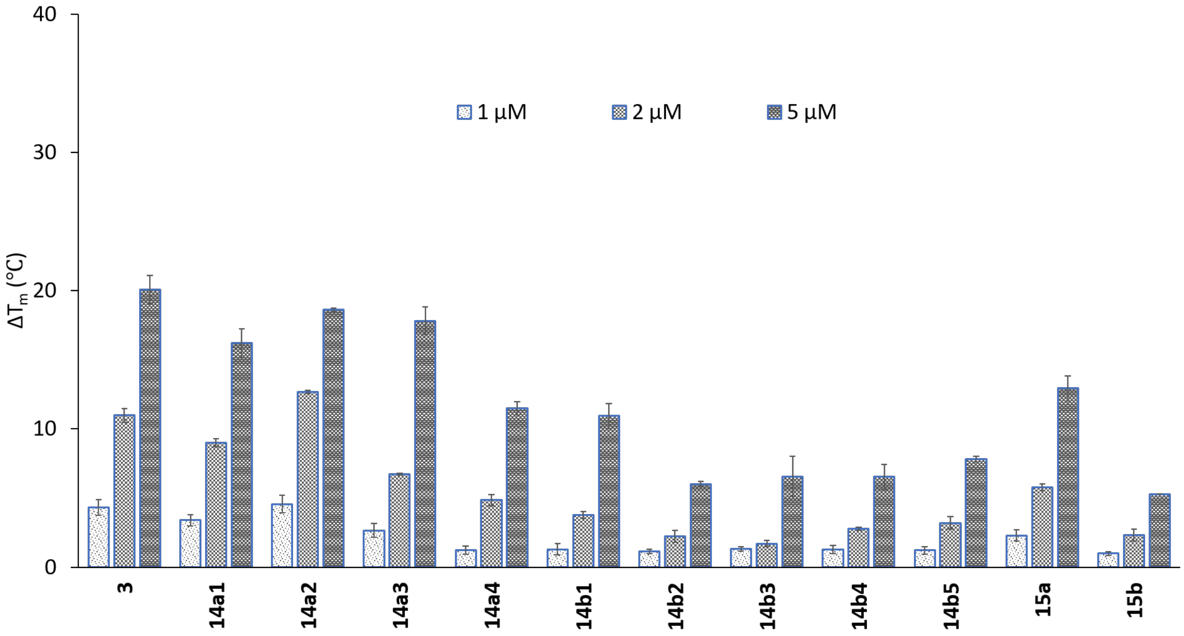

Figure S2. FRET melting assay of the 9 macrocycles with reference compound **3**. Experiments were performed with 0.2 µM FPu22T, 1, 2, or 5 µM ligand in 10 mM lithium cacodylate buffer (pH 7.2), 90 mM LiCl, and 10 mM KCl. T_m in absence of ligands of FPu22T was 68.2 ± 0.5 °C, and y-axis shows ΔT_m that corresponds to the T_m change in the absence and presence of ligand. Error bars correspond to SD of at least three independent experiments.

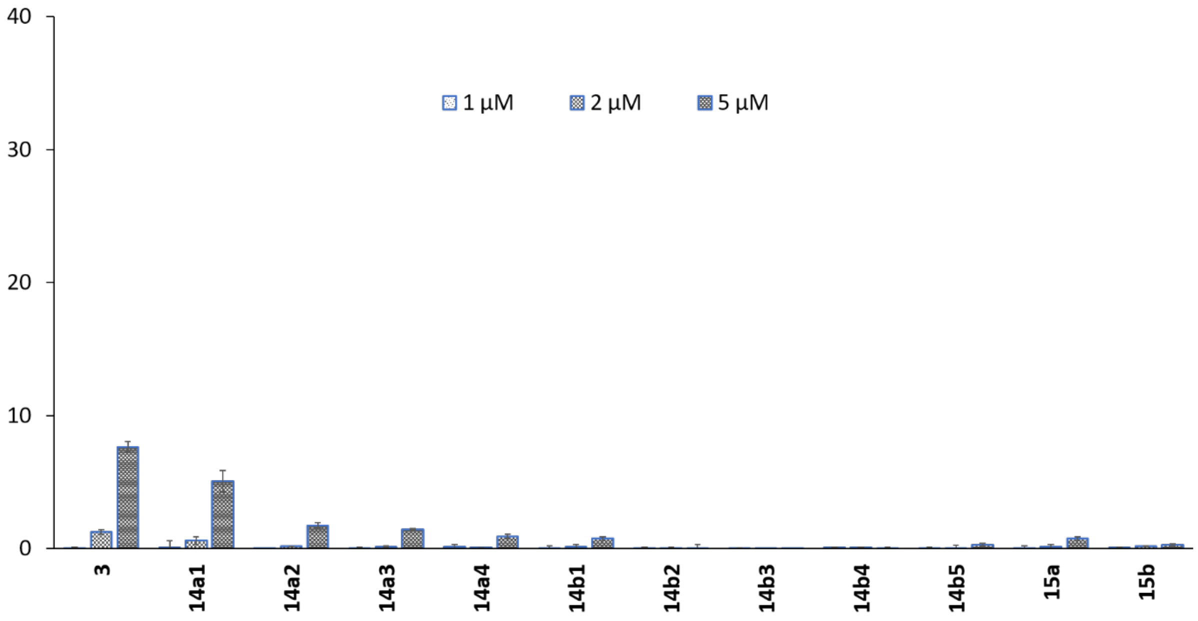

Figure S3. FRET melting study using dsDNA. Stabilization of Fds26T (0.2 µM) by the ligands (1, 2 and 5 µM). Experiments were performed in 10 mM lithium cacodylate buffer (pH 7.2), 90 mM LiCl, and 10 mM KCl. T_m in absence of ligands of Fds26T is 67.8 ± 0.3 °C. Error bars correspond to SD of at least three independent experiments.

Circular dichroism (CD) Spectra

5 µM of Pu22 G4-DNA was folded in 10 mM K-phosphate buffer (pH 7.4), with 5 mM (for Pu24T and Ckit2) or 1 mM (for Pu22) KCl by heating for 5 min at 95 °C and then allowed for cooling to room temperature. A quartz cuvette with a path length of 1 mm was used for the measurements by JASCO-720 spectropolarimeter (Jasco Internatiol Co. Ltd.). CD spectra were recorded at 25 °C over λ = 210-350 nm with an interval of 0.2nm and a scan rate of 100 nm/min. CD Melting curves for G4 DNA were recorded at 263 nm between 20-95°C at a speed of 1 °C/min. Melting temperature (T_m) is defined as the temperature at which 50% of the G4 structures are unfolded.

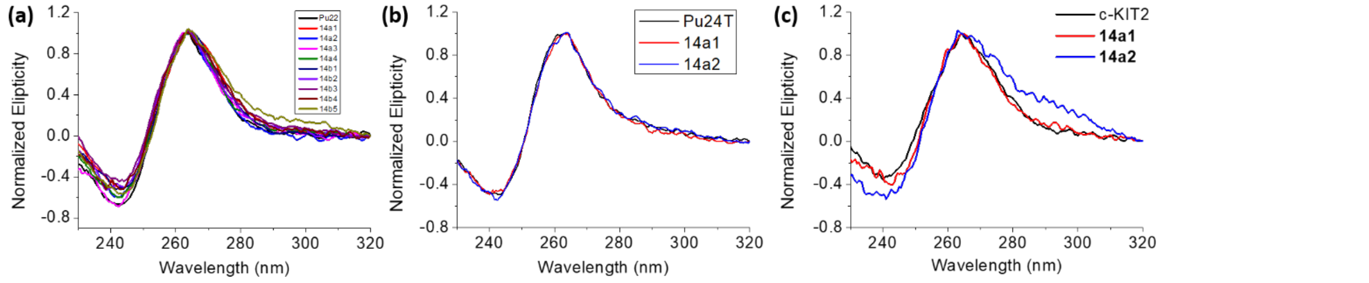

Figure S4. Normalized CD spectra of (a) Pu22 G4 DNA (5 µM) in absence and presence of different macrocycles (10 µM). Normalized CD spectra of (b) Pu24T G4 DNA (5 µM) (c) c-kIT2 G4 DNA (5 µM) in absence and presence **14a1** and **14a2** (10 µM).

Table S2. CD melting (ΔT_m in °C) data

Macrocycles	Pu24T	Pu22	c-KIT2
**14a1**	3.7 ± 0.2	6.7± 0.3	12.4 ± 0.4
**14a2**	3.3 ± 0.1	13.8 ± 0.2	18.8 ± 0.2
T_m in absence of ligands of Pu24T is 69.8 ± 0.2 °C, Pu22 is 76.1 ± 0.3 °C and c-KIT2 is 57.4 ± 0.2 °C. Error bars correspond to SD of two independent experiments.			

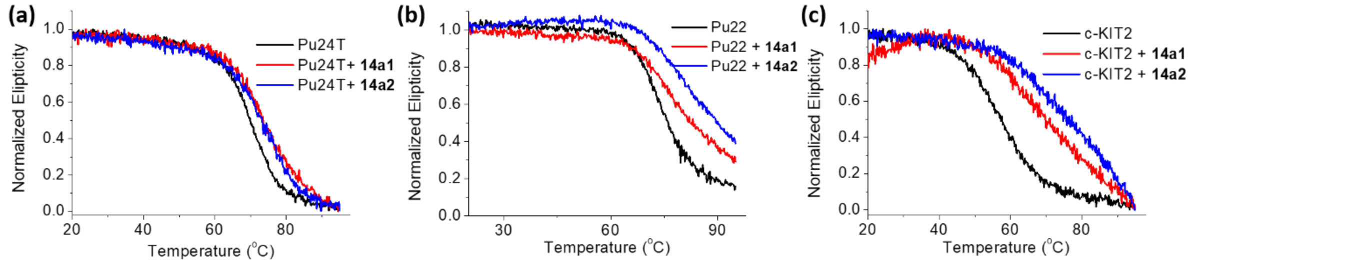

Figure S5. CD melting plot of (a) Pu24T (b) Pu22 and (c) c-KIT2 G4 DNA (5 µM) in absence and presence of **14a1** (10 µM) and **14a2** (10 µM).

Fluorescence Intercalator Displacement Experiments

Synthetic oligonucleotides for FID study were purchased from Eurofins Genomics. Stock solutions were prepared in water at 1 mM concentration. The sequences used are listed in supplementary Table S1. All the oligonucleotides (0.25 µM) were folded in 10 mM K-phosphate buffer (pH 7.4), with 100 mM KCl by heating for 5 min at 95 °C and then allowed for cooling to room temperature. The experiments were performed at 25 °C on a Jasco FP- 6500 spectrofluorometer equipped with a temperature controller. The prefolded G4-DNA (0.25 µM) was mixed with 0.50 µM Thiazole Orange (TO) and incubated for 2 minutes before the fluorescence spectrum

was recorded (λ_{ex} = 501 nm; λ_{em} = 510 – 650 nm). In case of dsDNA (0.25 µM), 0.75 µM Thiazole Orange (TO) was mixed. Then ligands were added to the mixture stepwise with a 2 min equilibration period, and the fluorescence spectra were recorded. The percentage of TO displacement was calculated from the fluorescence intensity (F) at the emission maxima, using the following equation:

$$Percentage\ of\ TO\ displacement = 100 - (\frac{F}{F_0} X100)$$

where, F_0 is the initial fluorescence intensity of TO bound to G4-DNA.

The percentage of TO displacement was plotted as a function of the concentration of added ligands and DC_{50} is determined. The binding constant (K_d) of the ligands were calculated from the following equations using K_a^{To} as 1.55 X 10^6 M^{-1}, 5.01X10^6 M^{-1} and 6 X 10^6 M^{-1} for Pu24T, Pu22 and *c-KIT2* respectively:

$$K_a^{ligand} = \frac{K_a^{TO} X\ [TO]}{[ligand]_{50}} \qquad K_d^{ligand} = \frac{1}{K_a^{ligand}}$$

Micro Scale Thermophoresis (MST)

5'- Cy5 labelled G4 DNAs for this study were purchased from Eurofins Genomics. Stock solutions were prepared in water at 100 µM concentration. The sequences used are listed in supplementary Table S1. The G4 DNA sequences were folded in KCl buffer (10 mM phosphate, 100 mM KCl, pH 7.4) by heating at 95 °C for 5 min and then cooling to room temperature. All the experiments were performed in 10 mM phosphate pH 7.4, 100 mM KCl, 0.05% Tween20 and 4% BSA. The labelled DNA concentration is held constant at 25 nM and ligand concentration is varied from 0.15 nM to 10 µM (fourteen 1:1 dilutions). The samples were loaded into standard MST graded glass capillaries and initial fluorescence intensity of the capillary were measured using Monolith NT.115 (Nano Temper, Germany) with 40% LED power. The change in fluorescence with ligand's concentrations were plotted in OriginPro 8 and fitted through non-linear equation to obtain the binding constants.

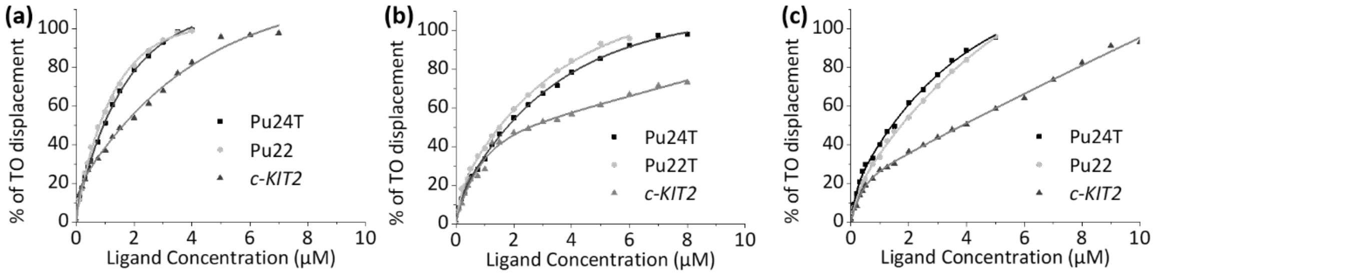

Figure S6. FID binding affinity plot for the ligands (a) **14a3**, (b) **14b3**, and (c) **14b5**. Thiazole orange (TO; 0.50 µM) displacement from the prefolded Pu24T, Pu22 and *c-KIT2* G4-DNA (0.25 µM), ligands concentration were varied from 0 to 13 µM. The experiments were carried in 10 mM potassium phosphate (pH 7.4) and 100 mM KCl buffer (λex= 501 nm; λ_{em}= 510 – 650 nm).

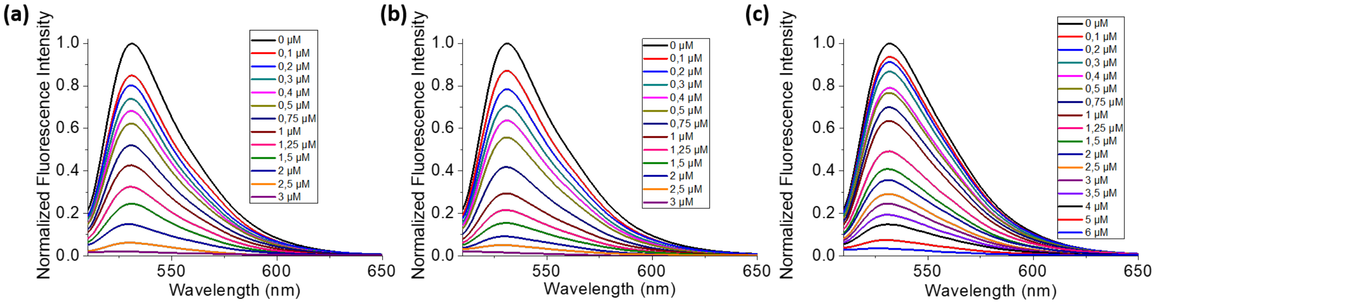

Figure S7. Thiazole orange (TO; 0.50 µM) displacement from the prefolded (a) Pu24T, (b) Pu22, (c) *c-KIT2* G4-DNA (0.25 µM) with compounds **14a2**. The experiments were carried in 10 mM potassium phosphate (pH 7.4) and 100 mM KCl buffer. The ligand concentration was varied from 0 to 6 µM (λ_{ex} = 501 nm; λ_{em} = 510 – 650 nm).

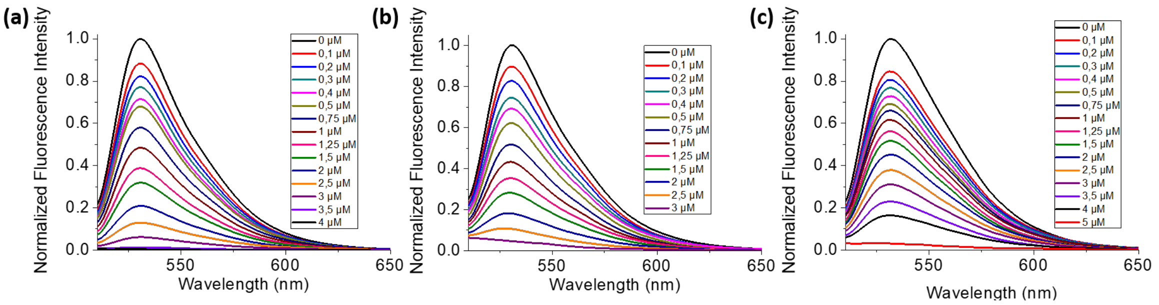

Figure S8. Thiazole orange (TO; 0.50 µM) displacement from the prefolded (a) Pu24T, (b) Pu22, (c) *c-KIT2* G4-DNA (0.25 µM) with compounds **14a3**. The experiments were carried in 10 mM potassium phosphate (pH 7.4) and 100 mM KCl buffer. The ligand concentration was varied from 0 to 5 µM (λ_{ex} = 501 nm; λ_{em} = 510 – 650 nm).

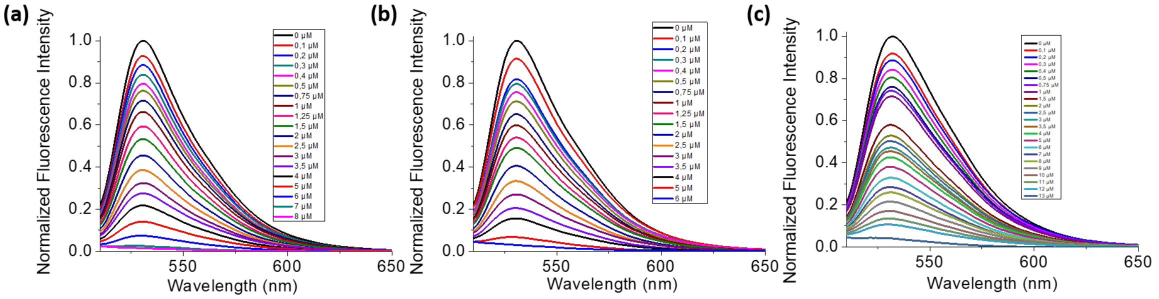

Figure S9. Thiazole orange (TO; 0.50 µM) displacement from the prefolded (a) Pu24T, (b) Pu22, (c) *c-KIT2* G4-DNA (0.25 µM) with compounds **14b3**. The experiments were carried in 10 mM potassium phosphate (pH 7.4) and 100 mM KCl buffer. The ligand concentration was varied from 0 to 13 µM (λ_{ex} = 501 nm; λ_{em} = 510 – 650 nm).

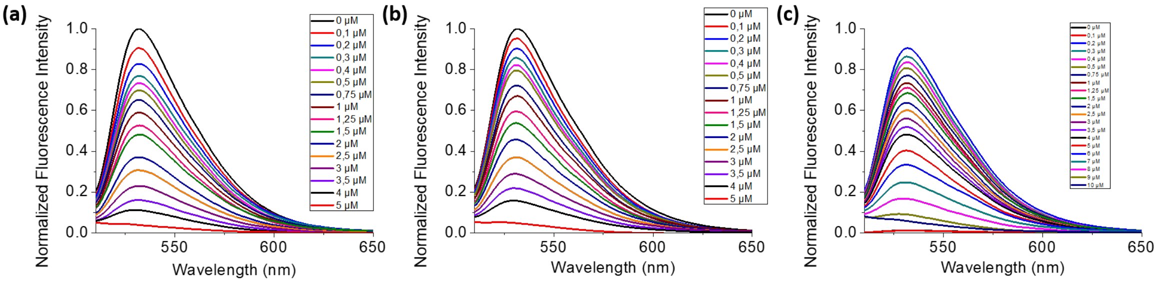

Figure S10. Thiazole orange (TO; 0.50 µM) displacement from the prefolded (a) Pu24T, (b) Pu22, (c) *c-KIT2* G4-DNA (0.25 µM) with compounds **14b5**. The experiments were carried in 10 mM potassium phosphate (pH 7.4) and 100 mM KCl buffer. The ligand concentration was varied from 0 to 13 µM (λ_{ex} = 501 nm; λ_{em} = 510 – 650 nm).

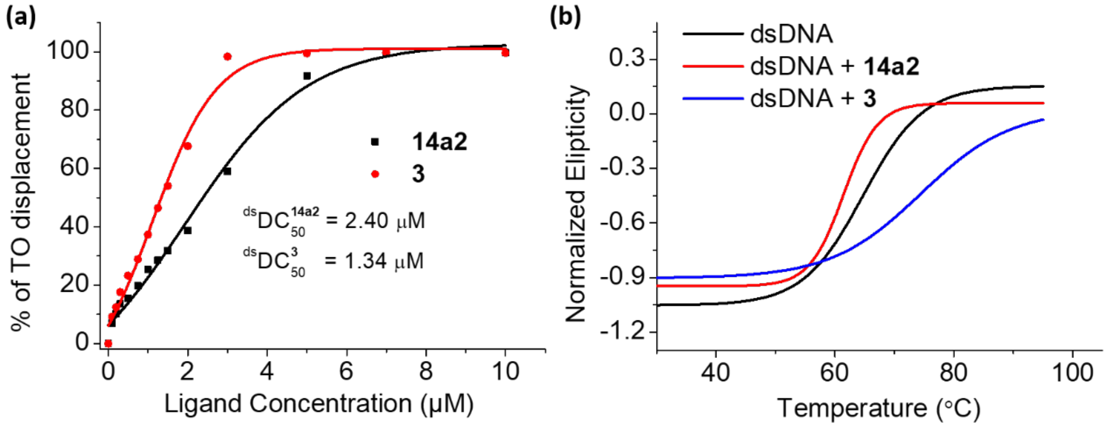

Figure S11. (a) % of TO displacement plot for the ligands **14a2** and **3**. Thiazole orange (TO; 0.75 µM) displacement from the dsDNA (ds26, 0.25 µM), ligands concentration were varied from 0 to 10 µM. The experiments were carried in 10 mM potassium phosphate (pH 7.4) and 100 mM KCl buffer (λex= 501 nm; λ_{em}= 510 – 650 nm). (b) CD melting curves ds26 DNA (10 µM) in absence and presence of **14a1** and **3** (30 µM) in 10 mM Li-cacodylate pH 7.4 buffer.

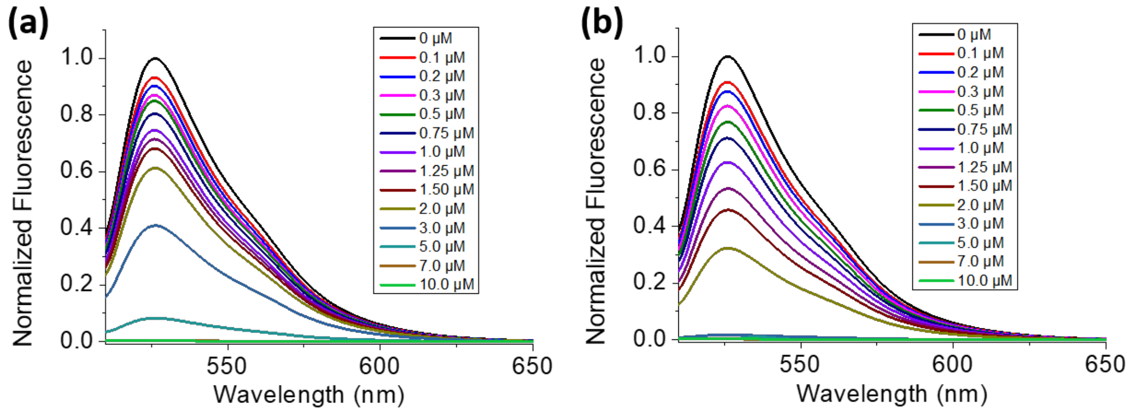

Figure S12. Thiazole orange (TO; 0.75 µM) displacement from the dsDNA (ds26; 0.25 µM) for (a) **14a2** and (b) Reference compound **3**. The experiments were carried in 10 mM potassium phosphate (pH 7.4) and 100 mM KCl buffer. The ligand concentration was varied from 0 to 10 µM (λ_{ex} = 501 nm; λ_{em} = 510 – 650 nm).

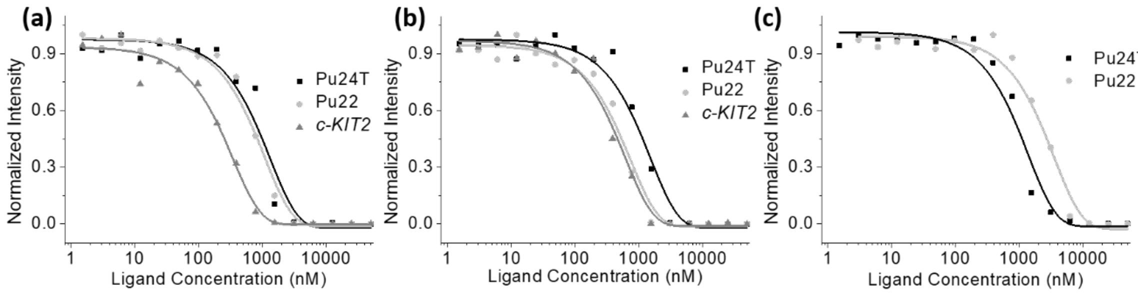

Figure S13. Fluorescence quenching based binding affinity plot of (a) **14a3**, (b) **14b3**, and (c) **14b5** for Pu24T, Pu22 and c-KIT2 G4 DNA

Nuclear Magnetic Resonance (NMR) Titrations

The G4 DNA stock solutions were prepared by folding 100 µM *c-MYC* Pu24T, Pu22 or *c-KIT* c-kit2 in 10 mM potassium phosphate buffer (pH = 7.4) and 35 mM KCl by heating to 95 °C and cooling to ambient temperature over ice. 10% D_2O was added to the DNA stock solutions, yielding a final DNA concentration of 90 µM. NMR samples were prepared by sequential addition of **14a2**, **14a3**, and **14b3** from 5 mM DMSO-d_6 stock solutions to 200 µL of the DNA solution which was then transferred to 3 mm NMR tubes. Control samples with Pu24T/Pu22 *c-MYC,* and c-kit2 *c-KIT* G4 DNA with and without 10% DMSO-d_6 was also performed to verify that DMSO did not have a significant effect on the DNA structure. All spectra were recorded at 298 K on a Bruker 850 MHz Avance III HD spectrometer equipped with a 5 mm TCI cryoprobe. Excitation sculpting was used in the 1D 1H experiments, and 256 scans were recorded. Processing of spectra was performed in MestreNova 10.0.2.

^{1}H NMR experiments with Ligand and DNA

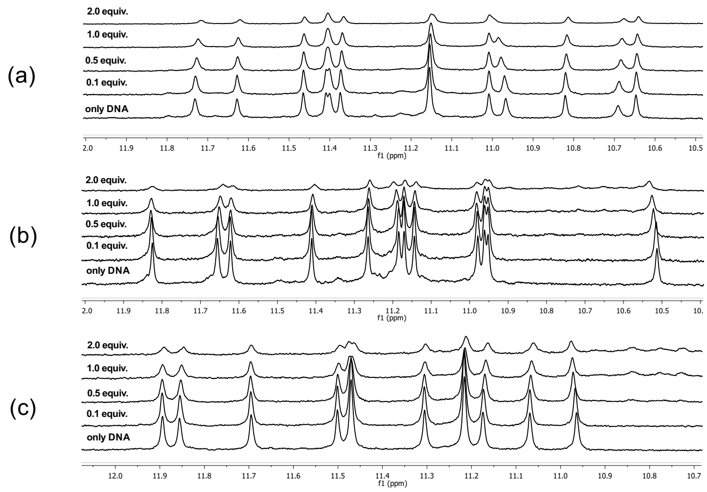

Figure S14. ^{1}H NMR (850 MHz) titrations for *c-MYC* Pu24T (**a**), *c-MYC* Pu22 (**b**), and *c-KIT2* (**c**) with **14a2**. The initial DNA concentration was 90 µM and macrocycle was then added so the last addition corresponded to a total molar ratio of DNA:macrocycle 1:2.

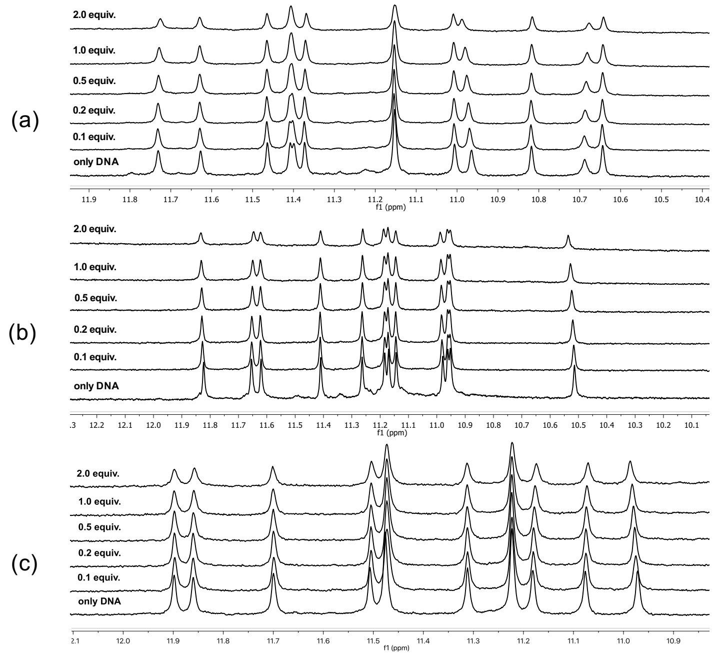

Figure S15. ^{1}H NMR (850 MHz) titrations for *c-MYC* Pu24T (**a**), *c-MYC* Pu22 (**b**), and *c-KIT2* (**c**) with **14a3**. The initial DNA concentration was 90 µM and macrocycle was then added so the last addition corresponded to a total molar ratio of DNA:macrocycle 1:2.

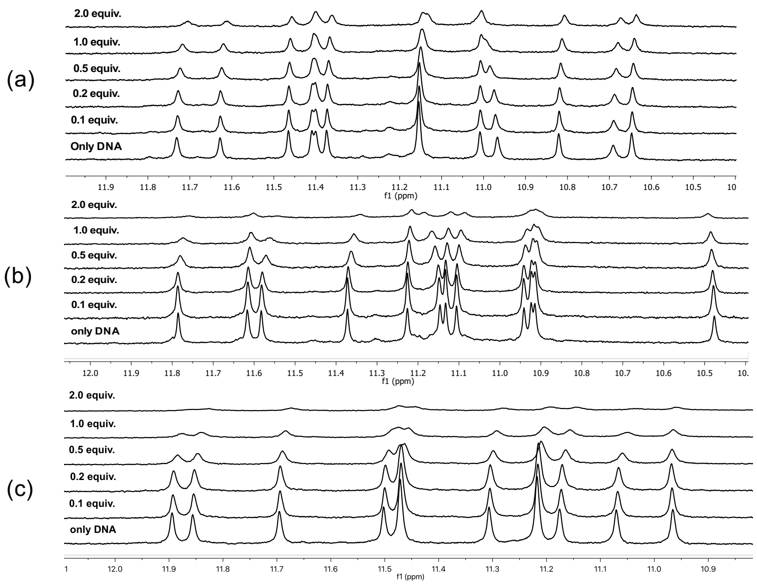

Figure S16. ^{1}H NMR (850 MHz) titrations for *c-MYC* Pu24T (**a**), *c-MYC* Pu22 (**b**), and *c-KIT2* (**c**) with **14b3**. The initial DNA concentration was 90 µM and macrocycle was then added so the last addition corresponded to a total molar ratio of DNA:macrocycle 1:2.

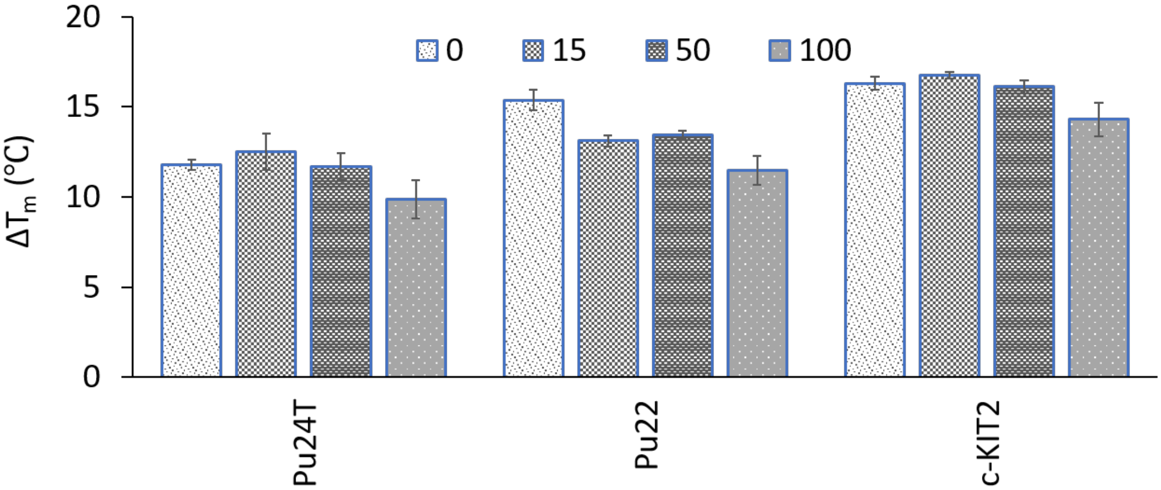

Figure S17. Stabilization ability of reference compound **3** (2 µM) for Pu24T, Pu22 and c-KIT2 G4-DNA (0.2 µM) in presence of excess amount (0-100 equivalent) of a double-stranded competitor dsDNA (ds26).

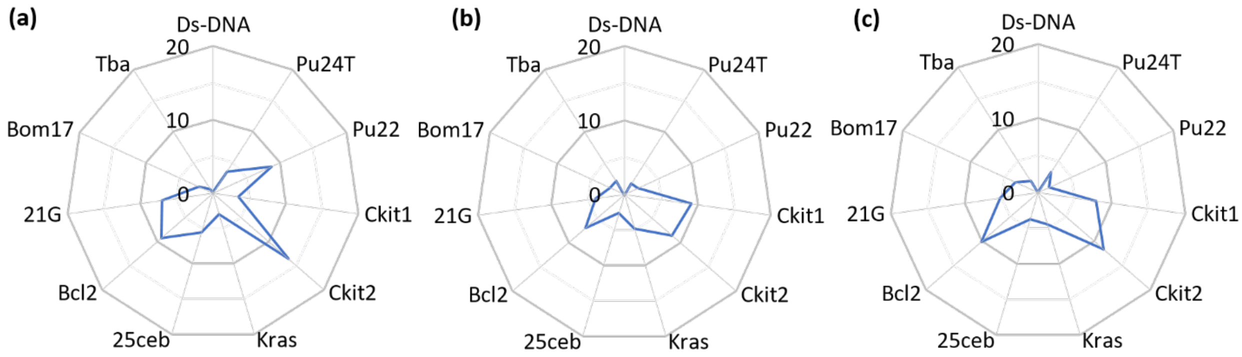

Figure S18. FRET melting assay of different G4 DNAs (0.2 µM) in presence of 2 µM of (a) **14a3**, (b) **14b3**, (c) **14b5**. All the experiments except for Pu22 were performed in 10 mM lithium cacodylate buffer (pH 7.2), 90 mM LiCl, and 10 mM KCl. T_m in absence of ligands for dsDNA is 68.0 ± 0.2 °C; Pu24T is 62.7 ± 0.3 °C; *c-KIT1* is 51.7 ± 0.2 °C; *c-KIT2* is 65.3 ± 0.1 °C; KRAS is 42.8 ± 0.4 °C; 25ceb is 74.4 ± 0.07 °C; Bcl2 is 60.2 ± 0.5 °C; 21G is 55.4 ± 0.3 °C and Bom17 is 38.7 ± 0.05°C. For Pu22 10 mM lithium cacodylate buffer (pH 7.2), 98 mM LiCl, and 2 mM KCl buffer was used. T_m in absence of ligands for Pu22 is 68.2 ± 0.5 °C.

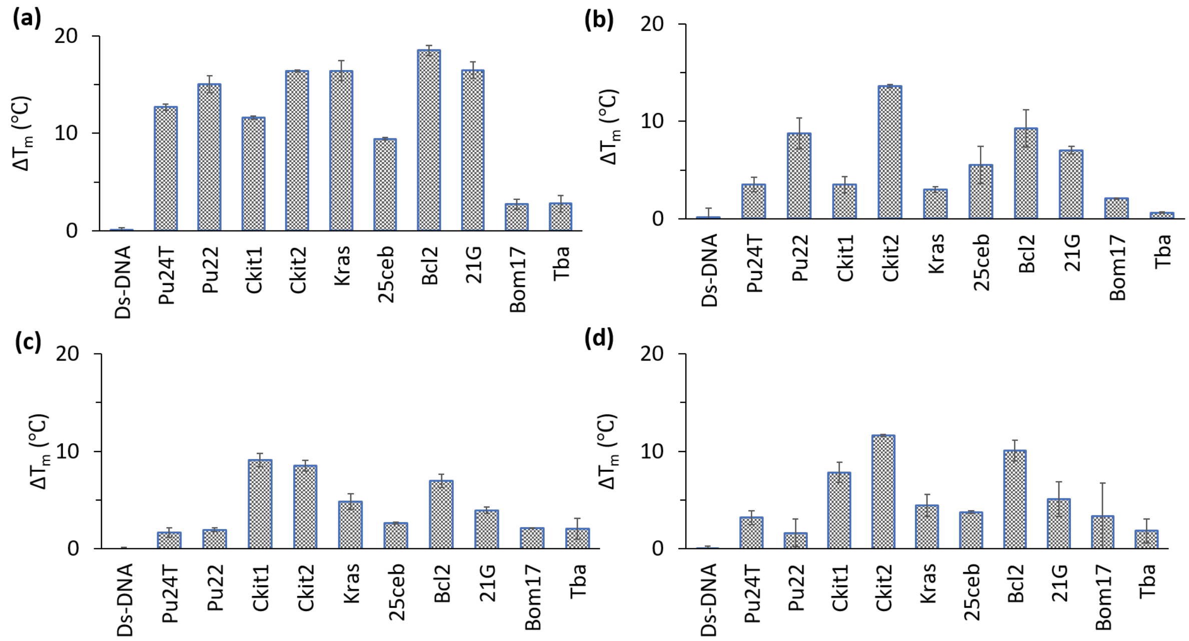

Figure S19. FRET melting assay of different G4 DNAs (0.2 µM) in presence of 2 µM of (a) **14a2**, (b) **14a3,** (c) **14b3** (d) **14b5**. All the experiments except for Pu22 were performed in 10 mM lithium cacodylate buffer (pH 7.2), 90 mM LiCl, and 10 mM KCl. T_m in absence of ligands for dsDNA is 68.0 ± 0.2 °C; Pu24T is 62.7 ± 0.3 °C; *c-KIT1* is 51.7 ± 0.2 °C; *c-KIT2* is 65.3 ± 0.1 °C; KRAS is 42.8 ± 0.4 °C; 25ceb is 74.4 ± 0.07 °C; Bcl2 is 60.2 ± 0.5 °C; 21G is 55.4 ± 0.3 °C and Bom17 is 38.7 ± 0.05°C. For Pu22 10 mM lithium cacodylate buffer (pH 7.2), 98 mM LiCl, and 2 mM KCl buffer was used. T_m in absence of ligands for Pu22 is 68.2 ± 0.5 °C.

G4 complex modeling

The structure coordinates for Pu24T *c-MYC* G4 DNA were downloaded from PDB (PDB-ID 2MGN)[1] and the bound ligand was removed from the Pu24T structure. Three-dimensional structural coordinates for **14a2** were generated using the Avogadro package.[2] **14a2** was manually placed on top of 5'-terminal G-tetrad using Chimera package.[3] Two potassium ions were preserved in the center channel of the Pu24T *c-MYC* G4 to maintain its stability during the simulations. Drug-like descriptors and lowest energy conformers were calculated in MOE (v.

2019.0102). TPSA were generated by first conducting a conformational search and then calculating the TPSA for the lowest energy conformation(s).

Table S3. Drug-like descriptors for the macrocyclic compounds and compound **3** calculated in MOE (v. 2019.0102) calculated from the lowest energy conformation(s) for each compound.

Compound	Molecular Weight (g/mol)	LogP	H-Bond Donor	H-Bond Acceptor	TPSA (Å^2)
14a1	843.94	5.78	6	5	327.01
14a2	756.87	5.59	6	4	296.24
14a3	770.89	6.09	6	4	271.42
14a4	784.92	6.60	6	4	275.88
14b1	872.00	6.00	6	5	353.96
14b2	756.87	5.60	6	4	316.88
14b3	770.89	6.10	6	4	280.18
14b4	784.92	6.61	6	4	323.92
14b5	798.95	7.12	6	4	274.62
15a	743.83	4.56	7	5	293.55
15b	771.88	5.01	7	5	299.31
3	616.73	6.06	4	2	196.81

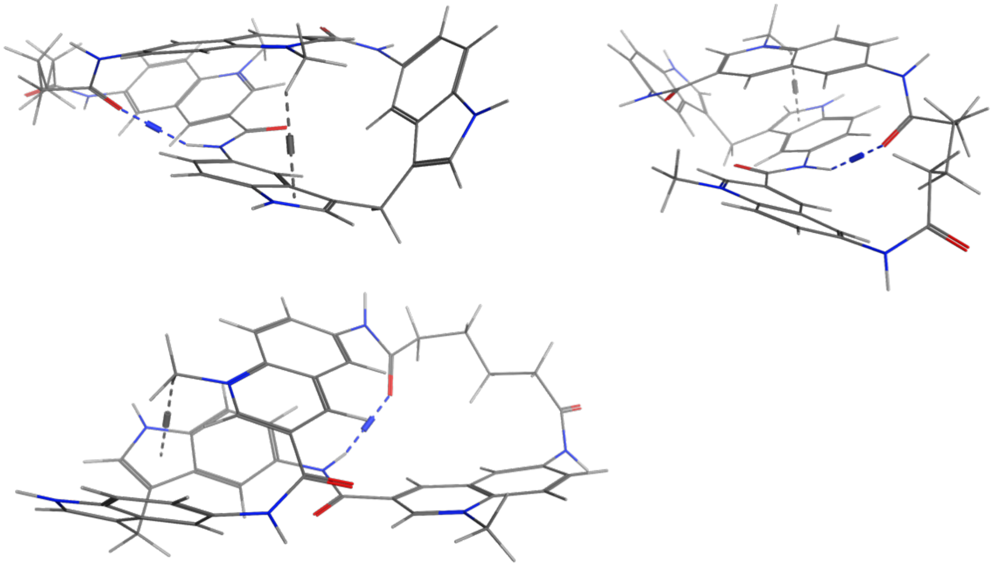

Figure S20. Lowest energy conformer of **14a2** from different angles showing its intramolecular interactions.

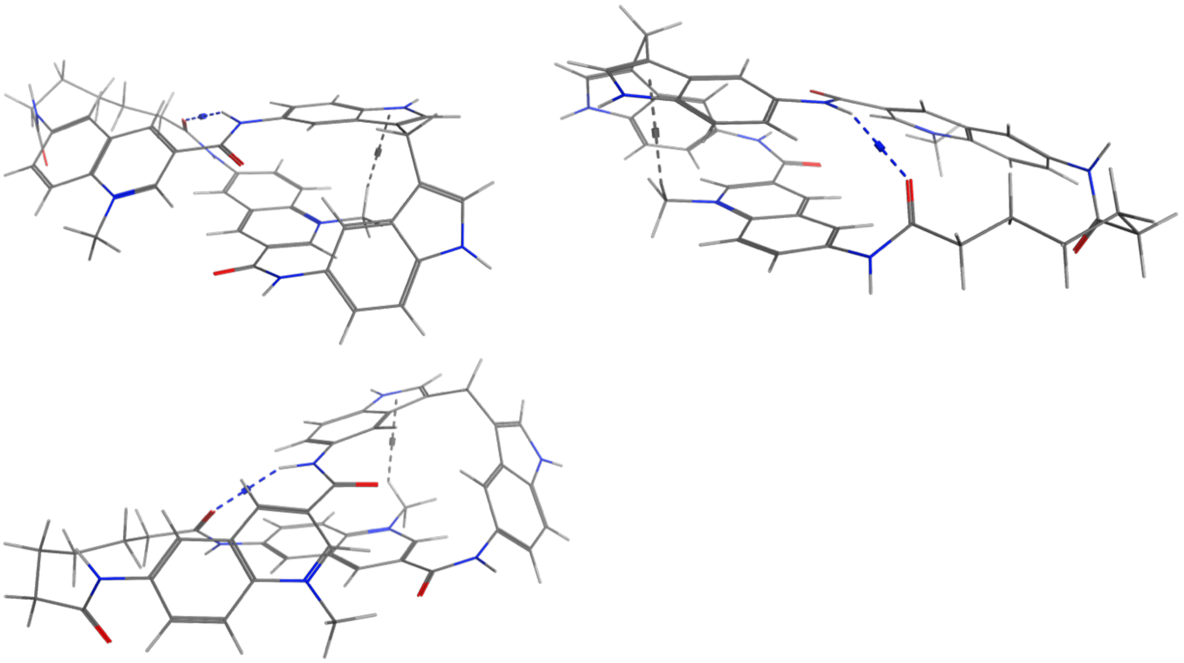

Figure S21. Lowest energy conformer of **14a3** from different angles showing its intramolecular interactions.

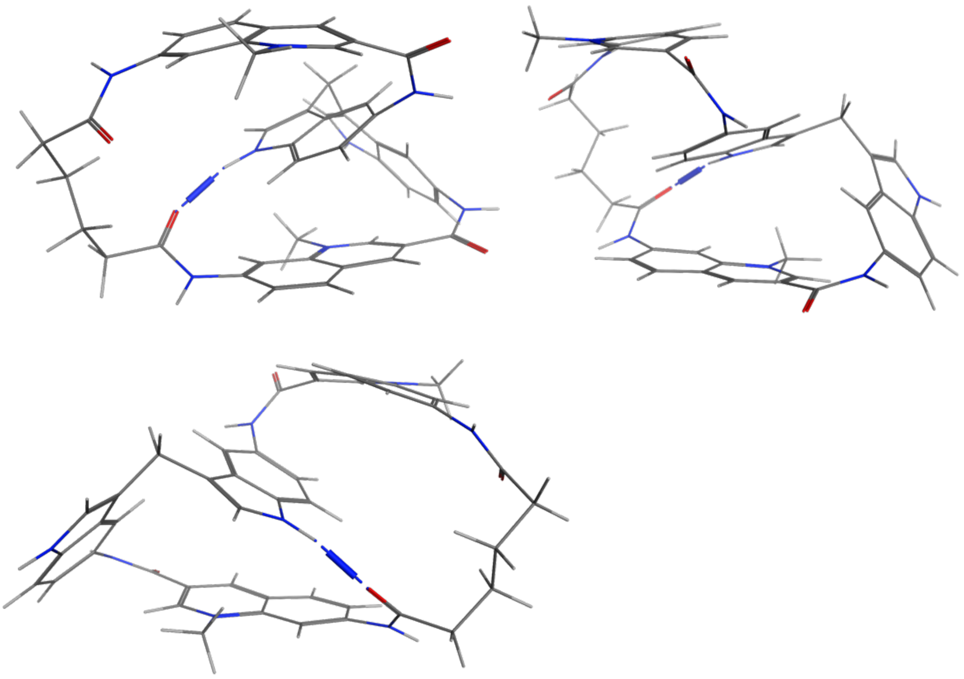

Figure S22. Lowest energy conformer of **14b2** from different angles showing its intramolecular interactions.

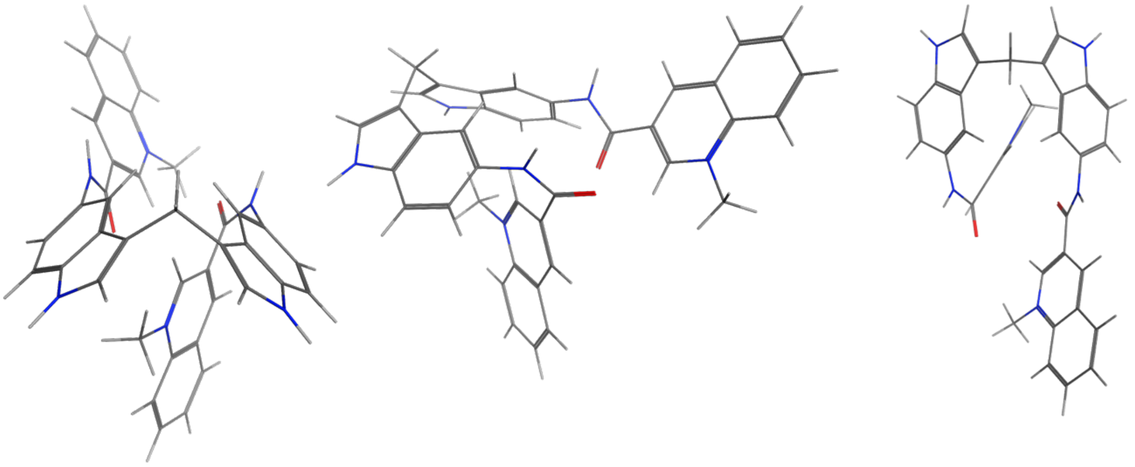

Figure S23. Lowest energy conformer of **3** from different angles.

Molecular dynamics simulations

The Pu24T *c-MYC* G4-**14a2** complex was prepared for molecular dynamics simulations using GROMACS[4] by placement at the center of a periodic dodecahedron box and solvating with water molecules. Subsequently, the system was neutralized by adding an excess of 100 mM KCl using the GROMACS tools. For the DNA, Amber99SB[5] with PARMBSC1[6] improvements were used as force-field parameters. For water, the TIP3P model[7] was used while ion parameters were taken from the following reference.[8] The geometry of **14a2** was first geometry optimized using PM6 method, ESP was calculated with B3LYP/6-31G+(p,d) basis set using Gaussian-16[9] and subsequently the partial atomic charges were calculated with the RESP method using the AmberTools package.[10] The force-field parameters were generated from GAFF using the AmberTools package and converted to GROMACS format using acpype script.[11] Subsequently, MD simulations were performed using GROMACS package.[4] Parameter settings for all these stages were previously described in the following reference.[12] Length of the MD simulations were 5*200 ns. All trajectories were merged, processed, and further used for the analysis. Conformational clustering for **14a2** bound to the Pu24T *c-MYC* G4 DNA structure was performed with the *gmx_clusterByFeatures* tool using PCA based conformational clustering (https://github.com/rjdkmr/gmx_clusterByFeatures). Subsequently, the first 50 frames of each cluster were considered for binding energy calculation using the *g_mmpbsa* tool.[13, 14] The obtained MD trajectories were visualized and images were rendered using VMD.[15]

Table S4: Binding energy for 14a2 with Pu24T *c-MYC* G4

Clusters	van der Waals	Electrostatic	Polar solvation	Non-polar solvation	Binding energy	Occurrence
1	-384 ± 2	-465 ± 1	386 ± 4	-28 ± 0	-491 ± 2	22
2	-442 ± 2	-486 ± 1	417 ± 3	-32 ± 0	-544 ± 3	19
3	-377 ± 2	-462 ± 1	410 ± 4	-27 ± 0	-456 ± 4	16
4	-356 ± 3	-446 ± 1	353 ± 3	-27 ± 0	-475 ± 4	14
5	-309 ± 1	-451 ± 1	399 ± 3	-20 ± 0	-382 ± 2	11
6	-369 ± 2	-446 ± 1	350 ± 5	-24 ± 0	-489 ± 5	10

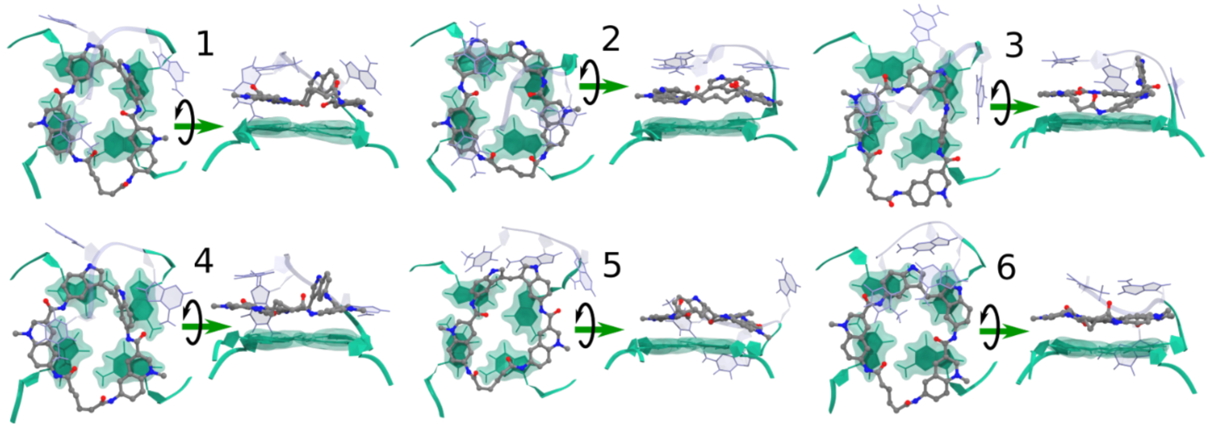

Figure S24. MD clusters of **14a2** with Pu24T *c-MYC* G4 DNA. Numbering is based on occurrence, see table S2.

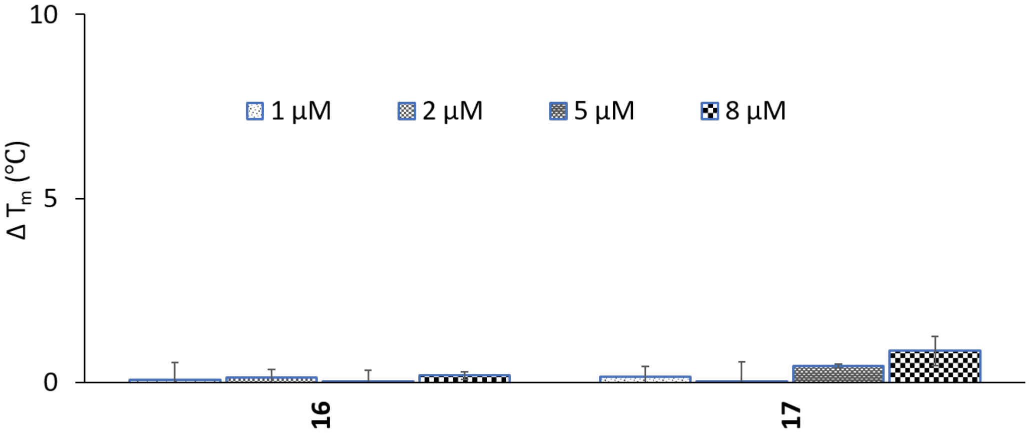

Figure S25. FRET melting study using dsDNA. Stabilization of Fds26T (0.2 µM) by the ligands **16** and **17** (1, 2, 5 and 8 µM). Experiments were performed in 10 mM lithium cacodylate buffer (pH 7.2), 90 mM LiCl, and 10 mM KCl. T_m in absence of ligands of Fds26T is 67.8 ± 0.3 °C. Error bars correspond to SD of at least three independent experiments.

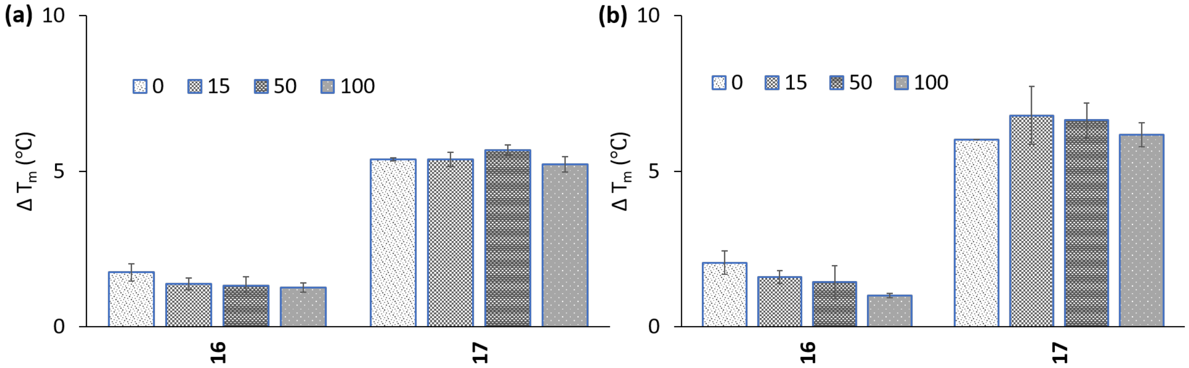

Figure S26. Stabilization ability of ligands **16** and **17** (5 µM) for (a) Pu24T and (b) Pu22 G4-DNA (0.2 µM) in presence of excess amount (0-100 equivalent) of a double-stranded competitor dsDNA (ds26).

General Experimental

All reagents and solvents were used as received from commercial suppliers unless stated otherwise. TLC was performed on aluminium backed silica gel plates (median pore size 60 Å, fluorescent indicator 254 nm) and detected with UV light. Flash column chromatography was performed using silica gel with an average particle diameter of 50 µm (range 40−65 µm, pore diameter 53 Å), eluents are given in brackets. DMF, THF and CH_2Cl_2 were dried in a solvent drying system (THF and CH_2Cl_2 drying agent: neutral alumina; DMF drying agent: activated molecular sieves, also equipped with an isocyanatescrubber) and were collected fresh prior to every reaction. ^{1}H and ^{13}C NMR spectra were recorded on a Bruker 400 MHz or 600 MHz spectrometer at 298 K, calibrated by using the residual peak of the solvents as the internal standard ($CDCl_3$: δ (ppm) H = 7.26; δ (ppm) C = 77.16. DMSO-d_6: δ (ppm) H = 2.50; δ (ppm) C = 39.50. Acetone-d_6: δ (ppm) H = 2.05; δ (ppm) C = 29.84, 206.26. CD_3OD: δ (ppm) H = 3.31; δ (ppm) C = 49.00). LC-MS was performed on an Agilent 6150 Series Quadrupole LC/MS system. HRMS was performed by using a Agilent 1290 binary LC System connected to a Agilent 6230 Accurate-Mass TOF LC/MS (ESI+); calibrated with Agilent G1969-85001 ESTOF Reference Mix containing ammonium trifluoroacetate, purine and hexakis (1H, 1H, 3H tetrafluoropropoxy) phosphazine in 90:10 CH_3CN:H_2O. Microwave reactions were carried out in an Initiator + microwave instrument from Biotage, using sealed 0.2–0.5 mL and 10-20 mL process vials. Reaction times refer to irradiation time at the target temperature, not the total irradiation time. The temperature was measured with an IR sensor.

General Procedure 1: Quinoline and Linker Amide Coupling

A flask of appropriate size was charged with **7** (2 equiv.) and desired di-acid linker (1 equiv.), the vial was then sealed and purged with N_2. Then, anhydrous CH_2Cl_2 and NMM (8 equiv.) were added followed by T3P (4 equiv.) and the solution was allowed to stir at ambient temperature for 4 h. The solvent was then removed under reduced pressure and DI water was added to form a precipitate. The formed precipitate was then collected by suction-filtration and washed with DI water and Et_2O to afford the pure compound.

General Procedure 2: Bis-Quinoline-Ester Hydrolysis

A flask of appropriate size was charged with bis-quinoline, then, THF:H_2O (1:1, 2 mL/0.1 mmol) was added followed by aq. LiOH (6 equiv. 1 M) and the mixture was stirred at ambient temperature for 4 h. The THF was then removed under reduced pressure and aq. HCl (1.2 equiv. 2 M) was added. The formed precipitate was collected with suction filtration and washed with DI water to afford the pure compound.

General Procedure 3: Macrocyclization

A pre-dried flask of appropriate size was charged with di-acid (1 equiv.) and TCFH (4 equiv.), sealed, and kept under N_2. Then, anhydrous DMF (15 mL) and N-methylimidazole (8 equiv.) were added and the solution was stirred for 10 min. Anhydrous DMF was then added (2 mM sol. in relation to the di-acid) and the flask was

evacuated and backfilled with N_2 (× 3). A solution of **4** (1.2 equiv.) in anhydrous DMF (6 mM conc.) was then added (20 μL/min) to the solution at ambient temperature and the reaction was allowed to stir for 24 h from the start of addition. The DMF was then removed under reduced pressure and sat. $NaHCO_3$ sol. was added (20 – 30 mL) to form a precipitate which was collected by suction filtration. Purification by flash column chromatography afforded the pure compound.

General Procedure 4: Methylation of Macrocycle

To a flask of appropriate size was added macrocycle (1 equiv.) and DMF (1 mL/4 mg) and the solution was heated to 40 °C. Then, CH_3I (20 equiv.) was added and the solution was stirred for 20 h at 40 °C. The solution was then concentrated under reduced pressure and then diluted with CH_2Cl_2, the formed precipitate was collected with suction filtration, washed with CH_2Cl_2 and n-heptane and dried to afford the pure compound.

3,3'-azanediyldipropionic acid

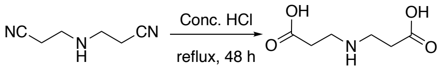

To a RBF (50 mL) containing 3,3'-iminodipropionitrile (5.0 g, 40.6 mmol) was added HCl (30 mL, 12 M) and the solution was refluxed for 48 h. While cooling to room temperature precipitate appeared and acetone was added, and the precipitate was filtered. The precipitate was recrystallized in hot water to obtain 3,3'-azanediyldipropionic acid (5.1 g, 78 %). *The Data is consistent with that reported in the literature.*[16]

3,3'-((*tert*-butoxycarbonyl)azanediyl)dipropionic acid

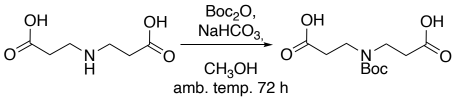

To a RBF (100 mL) containing 3,3'-azanediyldipropionic acid (2.70 g, 16.7 mmol) in CH_3OH (60 mL) Boc_2O (4.40 g, 20.1 mmol) and $NaHCO_3$ (2.80 g, 33.5 mmol) were added and stirred at ambient temperature for 72 hours. The solvent was removed under reduced pressure. Water (60 mL) was added to the residue and the resulting solution was washed with diethyl ether (40 mL x 2). The aqueous solution was adjusted to pH 3 with 2 N HCl. The acidic solution was extracted with EtOAc (100 mL × 3) and the combined organic layer was dried over Na_2SO_4, filtered and the solvent was removed under reduced pressure to afford 3,3'-((tert-butoxycarbonyl)azanediyl)dipropionic acid as colorless solid (3.5 g, 80 %). *The Data is consistent with that reported in the literature.*[17]

Bis(5-nitro-1*H*-indol-3-yl)methane (4)

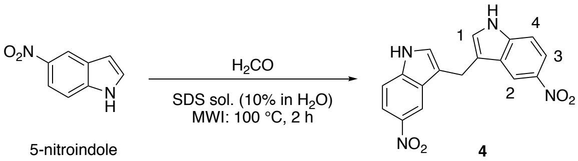

To a MW-vial (2-5 mL) was added 5-nitroindole (500 mg, 3.02 mmol) and H_2CO (226 µL, 3.02 mmol) followed by SDS sol. (10% in H_2O, 3 mL). The vial was then exposed to MWI, 100 °C, 2.5 h. The mixture was then diluted with EtOAc (10 mL) and sat. NaCl sol. (10 mL), the layers were then separated, and the aq. layer was then extracted with EtOAc (10 mL x 3). The combined org. layer was then dried over Na_2SO_4, filtered and concentrated under reduced pressure. Purification by flash column chromatography (eluent: 25% → 60% acetone in n-heptane) afforded **4** (470 mg, 92%) as a yellow solid.

^1H NMR (400 MHz, Acetone-d_6) δ (ppm) 10.74 (s, 2H, NH), 8.57 (d, J = 2.3 Hz, 2H, H-2), 8.02 (dd, J = 9.0, 2.3 Hz, 2H, H-3), 7.57 (d, J = 9.0 Hz, 2H, H-4), 7.52 (d, J = 2.3 Hz, 2H, H-1), 4.46 (s, 2H, CH_2). ^{13}C NMR (100 MHz, Acetone-d_6) δ (ppm) 141.95, 140.91, 127.72, 127.48, 117.99, 117.58, 116.82, 112.55, 21.46.

3,3'-methylenebis(1*H*-indol-5-amine) (5)

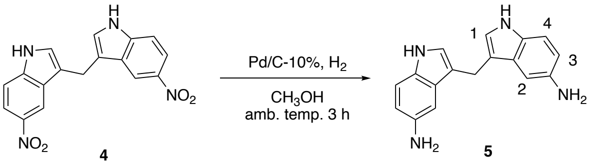

To a RBF (100 mL) was added **4** (752 mg, 2.23 mmol) and Pd/C-10% (237 mg, 0.223 mmol), then, CH_3OH (50 mL) was added and the flask was sealed and purged with H_2 for 10 minutes. The mixture was then stirred at ambient temperature under a balloon of H_2 for 3 h. After completion was the mixture filtered through a plug of celite with CH_3OH and the resulting org. layer was concentrated under reduced pressure to afford **5** without purification (616 mg, 95%) as beige solid.

^1H NMR (400 MHz, DMSO-d_6) δ (ppm) 10.20 (s, 2H, NH), 7.01 (d, J = 8.4 Hz, 2H, H-4), 6.82 (d, J = 2.3 Hz, 2H, H-1), 6.67 (d, J = 2.1 Hz, 2H, H-2), 6.44 (dd, J = 8.4, 2.2 Hz, 2H, H-3), 4.36 (s, 4H, NH_2), 3.87 (s, 2H, CH_2). ^{13}C NMR (100 MHz, DMSO-d_6) δ (ppm) 140.37, 130.28, 128.07, 122.49, 112.67, 111.55, 111.30, 102.08, 30.70.

Methyl 2-(hydroxy(3-nitrophenyl)methyl)acrylate (6a)

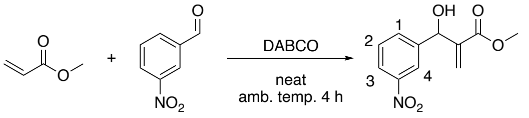

To a MW-vial was added 3-nitrobenzaldehyde (4.08g, 26.5 mmol) and DABCO (1.48 g, 13.2 mmol). Then, methyl acrylate (4.80 mL, 53.2 mmol) was added and the solution was stirred at ambient temperature for 4 h after which the solution had solidified. The solid was then dissolved in EtOAc and washed with sat. NaCl sol. (25 mL x 4). The resulting org. layer was then dried over Na_2SO_4, filtered and concentrated under reduced pressure. Purification by flash column chromatography (10% → 20% EtOAc in n-heptane) afforded **6a** (6.1 g, 97%) as a pale-yellow oil.

^{1}H NMR (400 MHz, $CDCl_3$) δ (ppm) 8.25 (t, J = 1.7 Hz, 1H, H-4), 8.14 (dd, J = 7.9, 1.7 Hz, 1H, H-3), 7.74 (d, J = 7.9 Hz, 1H, H-1), 7.52 (t, J = 7.9 Hz, 1H, H-2), 6.41 (s, 1H, CH_2), 5.90 (s, 1H, CH_2), 5.63 (s, 1H, CH), 3.74 (s, 3H, CH_3). ^{13}C NMR (100 MHz, $CDCl_3$) δ (ppm) 166.48, 148.44, 143.73, 141.06, 132.79, 129.47, 127.36, 122.88, 121.66, 72.69, 52.32.

Methyl 2-(hydroxy(4-nitrocyclohexa-2,4-dien-1-yl)methyl)acrylate (6b)

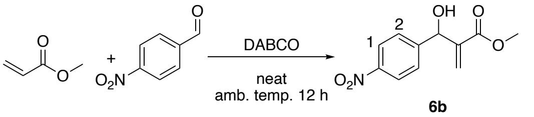

6b

To a RBF (50 mL) was added 4-nitrobenzaldehyde (10.4, 68.8 mmol) and DABCO (3.80 g, 34.4 mmol). Then, methyl acrylate (12.4 mL, 138 mmol) was added and the solution was stirred at ambient temperature for 12 h after which the solution had solidified. The solid was then dissolved in EtOAc and washed with sat. NaCl sol. (25 mL x 4). The resulting org. layer was then dried over Na_2SO_4, filtered and concentrated under reduced pressure. Purification by flash column chromatography (10% → 20% EtOAc in n-heptane) afforded **6b** (16 g, 98%) as a pale-yellow oil.

^{1}H NMR (400 MHz, $CDCl_3$) δ (ppm): 8.26 – 8.21 (m, 2H, H-1), 7.63 – 7.58 (m, 2H, H-2), 6.42 (s, 1H, CH_2), 5.89 (t, J = 1.0 Hz, 1H, CH_2), 5.66 (d, J = 6.2 Hz, 1H, CH), 3.78 (s, 3H, CH_3).

Methyl 2-(acetoxy(3-nitrophenyl)methyl)acrylate (7a)

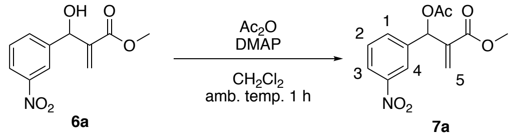

6a **7a**

To a RBF (100 mL) containing **6a** (3.21 g, 13.5 mmol) was added DMAP (323 mg, 2.64 mmol) and Ac_2O (1.92 mL, 20.3 mmol). Then, CH_2Cl_2 (50 mL) was added and the solution was stirred at ambient temperature for 1 h. The solution was then washed with sat. NaCl sol. (30 mL x 4) and the resulting the resulting org. layer was then dried over Na_2SO_4, filtered and concentrated under reduced pressure. Purification by flash column chromatography (eluent: 20% EtOAc in n-heptane) afforded **7a** (3.53 g, 93.7%) as a pale-yellow oil.

^{1}H NMR (400 MHz, $CDCl_3$) δ (ppm) 8.23 (t, J = 2.2 Hz, 1H, H-4), 8.17 (ddd, J = 8.0, 2.2, 1.2 Hz, 1H, H-3), 7.75 (dt, J = 8.0, 1.2 Hz, 1H, H-1), 7.53 (t, J = 8.0 Hz, 1H, H-2), 6.72 (s, 1H, H-5), 6.47 (s, 1H, H-5), 6.01 (d, J = 1.3 Hz, 1H, CH), 3.72 (s, 3H, CH_3), 2.14 (s, 3H, OAc). ^{13}C NMR (100 MHz, $CDCl_3$) δ (ppm) 169.27, 165.00, 148.42, 140.31, 138.64, 134.12, 129.55, 126.76, 123.43, 122.50, 72.19, 52.27, 21.07.

Methyl 2-(acetoxy(4-nitrocyclohexa-2,4-dien-1-yl)methyl)acrylate (7b)

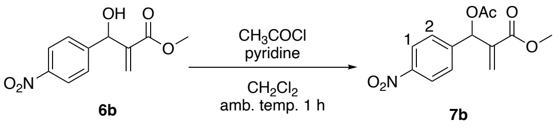

To a RBF (250 mL) containing **6b** (10 g, 42.1 mmol) in 120 mL anhydrous CH_2Cl_2 at 0 °C under an N_2 atmosphere pyridine (5.10 mL, 63.2 mmol) was added followed by acetyl chloride (4.50 mL, 63.2 mmol) and the solution was stirred at ambient temperature for 1.5 h. The progression of the reaction was monitored by TLC (40 % EtOAC in n-heptane). After completion was the solution washed with water followed by sat. NaCl sol. The organic layer was then dried over Na_2SO_4, filtered and concentrated under reduced pressure. Purification by flash column chromatography (eluent: 30% EtOAc in n-heptane) afforded **7b** (11.2 g, 95%) as white crystal.

^{1}H NMR (400 MHz, $CDCl_3$) δ (ppm): 8.20 (d, J = 8.8 Hz, 2H, H-1), 7.57 (d, J = 8.8 Hz, 2H, H-2), 6.72 (s, 1H, CH_2), 6.46 (s, 1H, CH_2), 5.97 (d, J = 1.3 Hz, 1H, CH), 3.72 (s, 3H, CH_3), 2.14 (s, 3H, OAc).

Methyl (*E*)-2-(azidomethyl)-3-(3-nitrophenyl)acrylate (8a)

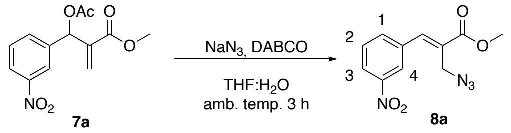

To a RBF (100 mL) containing **7a** (3.84 g, 13.7 mmol) was added DABCO (770 mg, 6.86 mmol) and NaN_3 (2.68 g, 41.2 mmol) followed by THF:H_2O (1:1, 28 mL). The cloudy solution was then stirred at ambient temperature for 3 h. The solution was then extracted with EtOAc (20 mL × 3). The combined org. layer was then washed with sat. NaCl sol. (20 mL x 4), dried over Na_2SO_4, filtered and concentrated under reduced pressure to afford **8a** (3.39 g, 94%) without further purification as a pale yellow solid.

^{1}H NMR (400 MHz, $CDCl_3$) δ (ppm) 8.30 (t, J = 1.7 Hz, 1H, H-4), 8.26 (dd, J = 7.9, 1.7 Hz, 1H, H-3), 7.96 (s, 1H, CH), 7.77 (d, J = 7.9 Hz, 1H, H-1), 7.63 (t, J = 7.9 Hz, 1H, H-3), 4.16 (s, 2H, CH_2), 3.92 (s, 3H, CH_3). ^{13}C NMR (100 MHz, $CDCl_3$) δ (ppm) 166.88, 148.54, 141.47, 135.74, 135.19, 130.02, 129.61, 124.36, 124.20, 52.86, 46.71.

Methyl (E)-2-(azidomethyl)-3-(4-nitrocyclohexa-2,4-dien-1-yl)acrylate (8b)

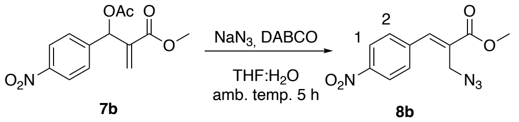

To a RBF (100 mL) containing suspension of **7b** (5.20 g, 18.6 mmol) in THF:H_2O (30 mL, 1:1) DABCO (3.10 g, 27.9 mmol) was added and stirred for 15 mins. The cloudy solution become clear and NaN_3 (3.60 g, 55.9 mmol) was then added stirring was continued at ambient temperature for 5 h. The solution was then extracted with EtOAc

(30 mL × 3). The combined org. layer was then washed with sat. NaCl sol. (20 mL x 4), dried over Na_2SO_4, filtered and concentrated under reduced pressure to afford **8b** (4.8 g, 98%) without further purification as a pale-yellow solid.

^{1}H NMR (400 MHz, $CDCl_3$) δ (ppm): 8.31 (d, J = 8.7 Hz, 2H), 7.99 (s, 1H, CH), 7.62 (d, J = 8.6 Hz, 2H, H-2), 4.16 (s, 2H, CH_2), 3.95 (s, 3H, CH_3).

Methyl-6-nitroquinoline-3-carboxylate (9a)

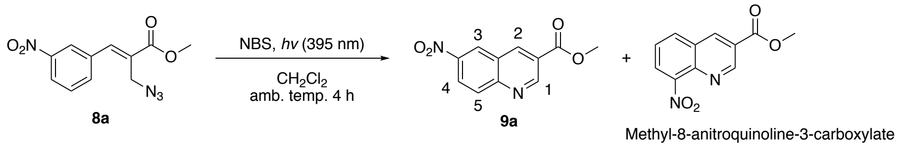

A RBF (500 mL) was charged with **8a** (1.39 g, 5.29 mmol) and NBS (1.88 g, 10.6 mmol), then, CH_2Cl_2 (220 mL) was added and the solution was deaerated with N_2 for 30 min and was then stirred under a LED light (395 nm) at ambient temperature for 4 h. The solution was then washed with sat $NaHCO_3$ sol. (50mL) and sat. $Na_2S_2O_3$ sol. (25 mL), and the org. layer was dried over Na_2SO_4, filtered and concentrated under reduced pressure. Purification by flash column chromatography (eluent: 4% EtOAc in PhMe) afforded **9a** (340 mg, 28%) as a white solid along with the unwanted isomer (combined yield: 87%). The individual yield was calculated by ^{1}H NMR. The two isomers were easily separated in the following step.

^{1}H NMR (400 MHz, $CDCl_3$) δ (ppm) 9.61 (d, J = 2.1 Hz, 1H, H-1), 9.03 (d, J = 2.1 Hz, 1H, H-2), 8.91 (d, J = 2.5 Hz, 1H, H-3), 8.59 (dd, J = 9.2, 2.5 Hz, 1H, H-4), 8.32 (d, J = 9.2 Hz, 1H, H-5), 4.06 (s, 3H, CH_3). ^{13}C NMR (100 MHz, $CDCl_3$) δ (ppm) 164.98, 153.48, 151.73, 146.30, 140.39, 131.65, 126.00, 125.78, 125.21, 124.96, 53.08.

Methyl 7-nitroquinoline-3-carboxylate (9b)

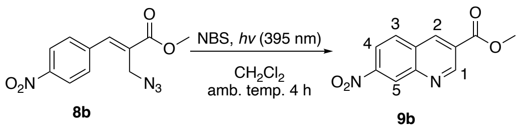

To a RBF (500 mL) containing **8b** (2.5 g, 9.5 mmol) in CH_2Cl_2 (400 mL), NBS (3.4 g, 19 mmol) was added and the solution was stirred under a LED light (395 nm) at ambient temperature for 4 h. The solution was then washed with saturated $NaHCO_3$ solution (50mL × 2) followed by saturated $Na_2S_2O_3$ solution (25 mL × 2), and the organic layer was dried over Na_2SO_4, filtered and concentrated under reduced pressure. The solid obtained was sonicated in Et_2O and filtered and washed with Et_2O to obtain **9b** (1.40 g, 63.5%) as a white powder.

^{1}H NMR (400 MHz, $CDCl_3$) δ (ppm): 9.62 (d, J = 2.1 Hz, 1H, H-1), 9.09 (d, J = 2.3 Hz, 1H, H-H-5), 8.97 (d, J = 2.1 Hz, 1H, H-2), 8.43 (dd, J = 9.0, 2.3 Hz, 1H, H-4), 8.14 (d, J = 9.0 Hz, 1H, H-3), 4.09 (s, 3H, CH_3). ^{13}C NMR (150 MHz, $CDCl_3$) δ (ppm): 164.9, 152.1, 149.5, 148.9, 138.4, 130.8, 130.0, 125.7, 125.5, 121.0, 52.9.

Methyl-6-aminoquinoline-3-carboxylate (10a)

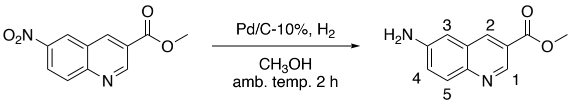

9a 10a

A RBF (100 mL) was charged with **9a** (450 mg, 1.94 mmol) and Pd/C-10% (206 mg, 0.19 mmol) followed by CH_3OH (50 mL). Then, the mixture was purged with H_2 for 5 min and was allowed to stir at ambient temperature for 1-2 h under a balloon of H_2. The mixture was then filtered over a plug of celite with CH_3OH and the resulting org. layer was concentrated under reduced pressure. Purification by flash column chromatography (eluent: 50% $\rightarrow$ 75% EtOAc in n-heptane) afforded **10a** (360 mg, 92%) as a yellow solid.

^{1}H NMR (400 MHz, Acetone-d_6) δ (ppm) 8.99 (d, J = 2.1 Hz, 1H, H-1), 8.52 (d, J = 2.1 Hz, 1H, H-2), 7.83 (d, J = 9.0 Hz, 1H, H-5), 7.39 (dd, J = 9.0, 2.6 Hz, 1H, H-4), 7.06 (d, J = 2.6 Hz, 1H, H-3), 5.33 (s, 2H, NH), 3.95 (s, 3H, CH_3). ^{13}C NMR (100 MHz, CD_3OD) δ (ppm) 167.30, 149.38, 145.56, 144.65, 137.80, 130.43, 129.73, 125.77, 124.46, 108.05, 52.86.

Methyl 7-aminoquinoline-3-carboxylate (10b)

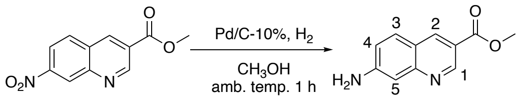

9b 10b

To a RBF (100 mL) containing **9b** (500 mg, 2.15 mmol) and Pd/C-10% (229 mg, 0.21 mmol) was added CH_3OH (80 mL). Then, the mixture was purged with a H_2 balloon for 5 min and allowed to stir at ambient temperature for 1 h under a H_2 balloon. The mixture was then filtered over a plug of celite with CH_3OH and the resulting organic layer was concentrated under reduced pressure to afford **10b** (410 mg, 94.2%) without further purification as a green powder.

^{1}H NMR (600 MHz, DMSO-d_6) δ (ppm): 9.02 (d, J = 2.2 Hz, 1H, H-1), 8.60 (d, J = 2.2 Hz, 1H, H-2), 7.81 (d, J = 8.8 Hz, 1H, H-3), 7.06 (dd, J = 8.8, 2.1 Hz, 1H, H-4), 6.96 (d, J = 2.1 Hz, 1H, H-5), 6.29 (d, J = 7.1 Hz, 2H, NH_2), 3.88 (s, 3H, CH_3). ^{13}C NMR (150 MHz, DMSO-d_6) δ (ppm): 166.3, 153.4, 152.4, 150.0, 137.9, 131.0, 119.9, 119.2, 117.6, 106.1, 52.4.

Dimethyl-6,6'-((2,2'-((*tert*-butoxycarbonyl)azanediyl)bis(acetyl))bis(azanediyl))bis(quinoline-3-carboxylate) (11a1)

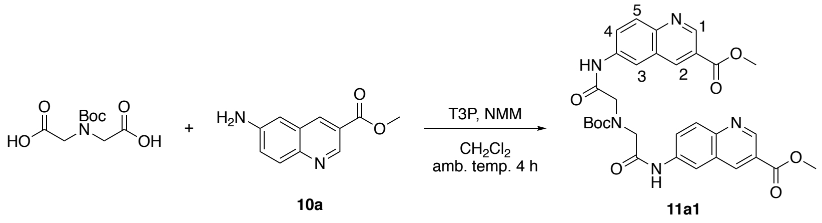

Following general procedure 1: **10a** (150 mg, 0.74 mmol), di-acid (87.0 mg, 0.37 mmol), NMM (330 µL, 3.00 mmol) and T3P (890 µL, 1.50 mmol) in anhydrous CH_2Cl_2 (6 mL) were reacted according to the general procedure to afford **11a1** (182 mg, 81%) as a pale-yellow solid.

^{1}H NMR (400 MHz, $CDCl_3$) δ (ppm) 11.06 (s, 1H, NH), 10.04 (s, 1H, NH), 9.33 (s, 1H, H-1), 9.21 (s, 1H, H-1), 8.87 (s, 1H), 8.66 (s, 1H), 8.59 (s, 1H), 8.36 – 8.22 (m, 2H), 8.11 – 7.99 (m, 2H), 7.90 (d, *J* = 8.7 Hz, 1H), 4.39 (s, 2H, CH_2), 4.26 (s, 2H, CH_2), 4.06 – 4.00 (s, 6H, OCH_3), 1.42 (s, 9H, Boc). ^{13}C NMR (151 MHz, $CDCl_3$) δ (ppm) 169.73, 168.89, 165.99, 165.69, 155.50, 148.99, 147.18, 138.73, 138.36, 137.36, 136.62, 130.41, 129.98, 127.71, 127.17, 125.89, 125.66, 123.58, 123.48, 117.41, 117.01, 82.89, 55.74, 54.65, 52.73, 52.67, 28.35; HRMS: *m/z*: $[M+H]^+$ calcd for $C_{31}H_{32}N_5O_8$ 602.2245; Found 602.2247.

Dimethyl 7,7'-((3,3'-((*tert*-butoxycarbonyl)azanediyl)bis(propanoyl))bis(azanediyl))bis(quinoline-3-carboxylate) (11b1)

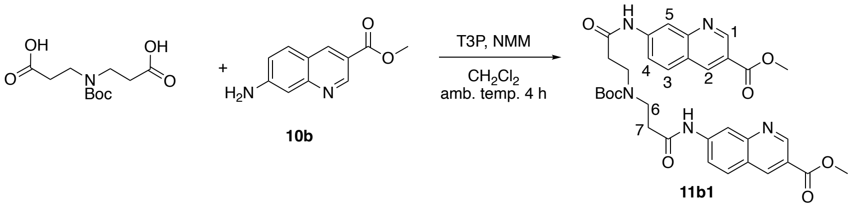

Following general procedure 1: **10b** (80 mg, 0.40 mmol), di-acid (51.7 mg, 0.20 mmol), NMM (174 µL, 1.60 mmol) and T3P (471 µL, 0.80 mmol) in anhydrous CH_2Cl_2 (4 mL) were reacted according to the general procedure to afford **11b1** (120 mg, 96.3%) as a yellow solid.

^{1}H NMR (600 MHz, DMSO-d_6) δ (ppm): 10.51 (s, 2H, NH), 9.22 (d, *J* = 2.2 Hz, 2H, H-1), 8.85 (d, *J* = 2.2 Hz, 2H, H-2), 8.49 (s, 2H, H-5), 8.10 (d, *J* = 8.9 Hz, 2H, H-3), 7.77 (dd, *J* = 8.9, 2.1 Hz, 2H, H-4), 3.94 (s, 6H, CH_3), 3.60 – 3.56 (m, 4H, H-7), 2.75 – 2.65 (m, 4H, H-6), 1.36 (s, 9H, Boc). ^{13}C NMR (151 MHz, DMSO-d_6) δ (ppm): 170.9, 165.8, 156.7, 154.9, 150.7, 150.2, 142.8, 138.3, 130.6, 123.1, 121.4, 115.7, 79.3, 77.5, 52.8, 28.7, 28.4. HRMS: *m/z*: $[M+H]^+$ calcd for $C_{33}H_{36}N_5O_8$ 630.2451; Found 630.2405.

Dimethyl 6,6'-(adipoylbis(azanediyl))bis(quinoline-3-carboxylate) (11a2)

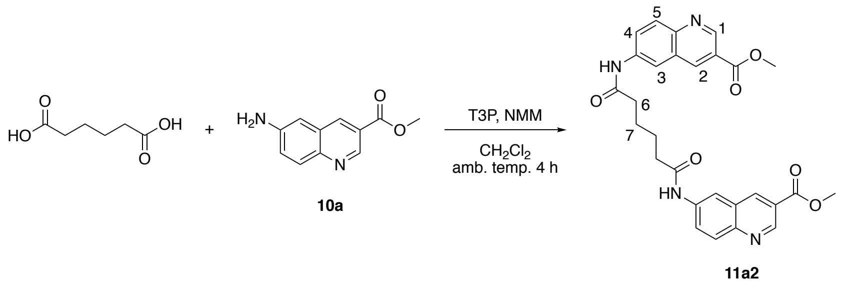

Following general procedure 1: **10a** (100 mg, 0.49 mmol), di-acid (36.0 mg, 0.25 mmol), NMM (220 μL, 2.00 mmol) and T3P (590 μL, 1.00 mmol) in anhydrous CH_2Cl_2 (5 mL) were reacted according to the general procedure to afford **11a2** (98 mg, 77%) as a white solid.

^{1}H NMR (400 MHz, DMSO-d_6) δ (ppm) 10.37 (s, 2H, NH), 9.17 (d, J = 1.7 Hz, 2H, H-1), 8.86 – 8.84 (d, J = 1.7 Hz, 2H, H-2), 8.50 – 8.47 (d, J = 0.4 Hz, 2H, H-3), 8.04 (d, J = 9.0 Hz, 2H, H-5), 8.00 – 7.95 (dd, J = 9.0, 0.4 Hz, 2H, H-4), 3.94 (s, 6H, CH_3), 2.46 (m, 4H, H-6), 1.73 (m, 4H, H-7). ^{13}C NMR (100 MHz, DMSO-d_6) δ (ppm) 171.73, 165.32, 147.62, 146.03, 138.15, 137.73, 129.40, 127.00, 125.90, 122.90, 115.78, 52.46, 36.30, 24.76; HRMS: m/z: [M+H]$^+$ calcd for $C_{28}H_{27}N_4O_6$ 515.1925; Found 515.1921.

Dimethyl 7,7'-(adipoylbis(azanediyl))bis(quinoline-3-carboxylate) (11b2)

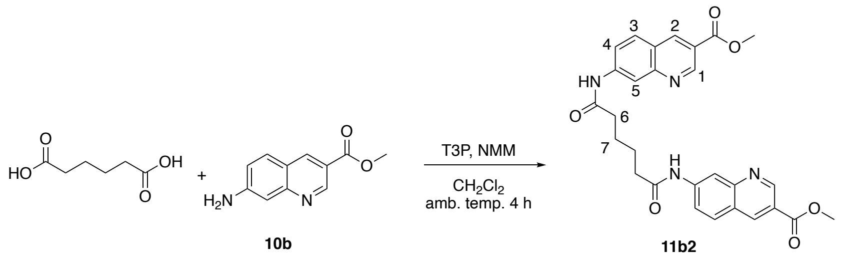

Following general procedure 1: **10b** (138 mg, 0.68 mmol), di-acid (50 mg, 0.34 mmol), NMM (301 μL, 2.70 mmol) and T3P (814 μL, 1.37 mmol) in anhydrous CH_2Cl_2 (4 mL) were reacted according to the general procedure to afford **11b2** (128 mg, 73%) as a yellow solid.

^{1}H NMR (400 MHz, DMSO-d_6) δ (ppm): 10.45 (s, 2H, NH), 9.24 (d, J = 2.2 Hz, 2H, H-1), 8.88 (d, J = 2.2 Hz, 2H, H-2), 8.53 (d, J = 2.0 Hz, 2H, H-5), 8.12 (d, J = 7.9 Hz, 2H, H-3), 7.79 (d, J = 7.9 Hz, 2H, H-4), 3.94 (s, 6H, CH_3), 2.54 – 2.52 (m, 4H, H-6), 1.75 – 1.71 (m, 4H, H-7). ^{13}C NMR (150 MHz, DMSO-d_6) δ (ppm): 172.5, 165.8, 150.8, 150.2, 143.0, 138.4, 130.6, 123.1, 121.5, 121.4, 115.7, 52.8, 36.9, 25.1. HRMS: m/z: [M+H]$^+$ calcd for $C_{28}H_{27}N_4O_6$ 515.1926; Found 515.1978.

Dimethyl 6,6'-(heptanedioylbis(azanediyl))bis(quinoline-3-carboxylate) (11a3)

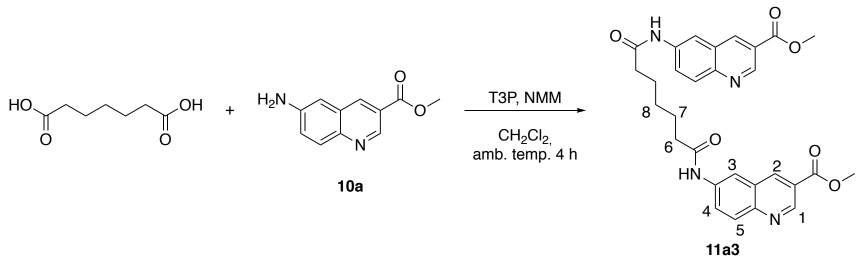

Following general procedure 1: **10a** (150 mg, 0.74 mmol), di-acid (59 mg, 0.37 mmol), NMM (330 μL, 3.00 mmol) and T3P (890 μL, 1.50 mmol) in anhydrous CH_2Cl_2 (6 mL) were reacted according to the general procedure to afford **11a3** (145 mg, 75%) as a white solid.

^{1}H NMR (600 MHz, DMSO-d_6) δ (ppm) 10.34 (s, 2H, NH), 9.16 (d, J = 2.1 Hz, 2H, H-1), 8.82 (d, J = 2.1 Hz, 2H, H-2), 8.47 (d, J = 2.3 Hz, 2H, H-3), 8.02 (d, J = 9.1 Hz, 2H, H-5), 7.96 (dd, J = 9.1, 2.3 Hz, 2H, H-4), 3.94 (s, 6H, CH_3), 2.42 (t, J = 7.4 Hz, 4H, H-6), 1.75 – 1.65 (m, 4H, H-7), 1.42 (m, 2H, H-8). ^{13}C NMR (151 MHz, DMSO-d_6) δ (ppm) 171.84, 165.30, 147.58, 146.01, 138.14, 137.69, 129.37, 126.98, 125.87, 122.86, 115.72, 52.44, 36.27, 28.24, 24.80; HRMS: m/z: [M+H]$^+$ calcd for $C_{29}H_{29}N_4O_6$ 529.2082; Found 529.2088.

Dimethyl 7,7'-(heptanedioylbis(azanediyl))bis(quinoline-3-carboxylate) (11b3)

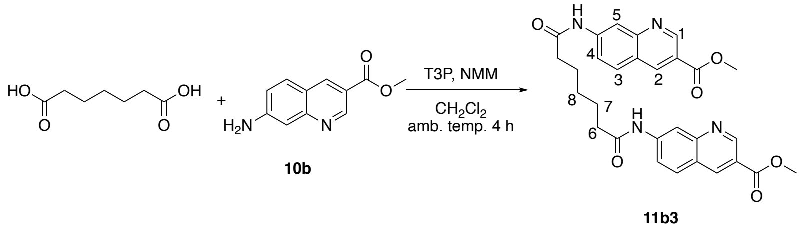

Following general procedure 1: **10b** (151 mg, 0.74 mmol), di-acid (60 mg, 0.37 mmol), NMM (329 μL, 3 mmol) and T3P (892 μL, 1.5 mmol) in anhydrous CH_2Cl_2 (4 mL) were reacted according to the general procedure to afford **11b3** (180 mg, 91%) as a yellow solid.

^{1}H NMR (600 MHz, DMSO-d_6) δ (ppm): 10.42 (s, 2H, NH), 9.23 (d, J = 2.1 Hz, 2H, H-1), 8.86 (d, J = 2.1 Hz, 2H, H-2), 8.51 (d, J = 2.1 Hz, 2H, H-5), 8.10 (d, J = 8.9 Hz, 2H, H-3), 7.77 (dd, J = 8.9, 2.1 Hz, 2H, H-4), 3.94 (s, 6H, CH_3), 2.45 (t, J = 7.5 Hz, 4H, H-6), 1.71 (p, J = 7.5 Hz, 4H, H-7), 1.42 (tt, J = 9.5, 6.4 Hz, 2H, H-8). ^{13}C NMR (150 MHz, DMSO-d_6) δ (ppm): 172.6, 165.8, 150.8, 150.2, 143.0, 138.3, 130.6, 123.1, 121.5, 121.3, 115.6, 52.8, 39.6, 36.9, 28.7, 25.2. HRMS: m/z: [M+H]$^+$ calcd for $C_{29}H_{29}N_4O_6$ 529.2082; Found 529.2029.

Dimethyl 6,6'-(octanedioylbis(azanediyl))bis(quinoline-3-carboxylate) (11a4)

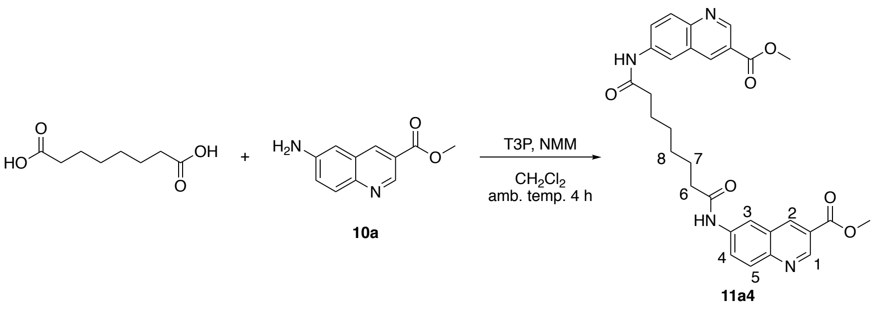

Following general procedure 1: **10a** (150 mg, 0.74 mmol), di-acid (65 mg, 0.37 mmol), NMM (330 μL, 3.00 mmol) and T3P (890 μL, 1.50 mmol) in anhydrous CH_2Cl_2 (6 mL) were reacted according to the general procedure to afford **11a4** (160 mg, 79%) as a white solid.

^{1}H NMR (600 MHz, DMSO-d_6) δ (ppm) 10.33 (s, 2H, NH), 9.17 (d, J = 2.1 Hz, 2H, H-1), 8.84 (d, J = 2.1 Hz, 2H, H-2), 8.48 (d, J = 2.3 Hz, 2H, H-3), 8.03 (d, J = 9.0 Hz, 2H, H-5), 7.96 (dd, J = 9.1, 2.3 Hz, 2H, H-4), 3.94 (s, 6H, CH_3), 2.41 (t, J = 7.4 Hz, 4H, H-6), 1.66 (m, 4H, H-7), 1.42 – 1.36 (m, 4H, H-8). ^{13}C NMR (151 MHz, DMSO-d_6) δ (ppm) 171.86, 165.31, 147.58, 146.01, 138.15, 137.70, 129.36, 126.99, 125.88, 122.87, 115.73, 52.45, 36.36, 28.47, 24.90; HRMS: m/z: [M+H]$^+$ calcd for $C_{30}H_{31}N_4O_6$ 543.2238; Found 543.2243.

Dimethyl 7,7'-(octanedioylbis(azanediyl))bis(quinoline-3-carboxylate) (11b4)

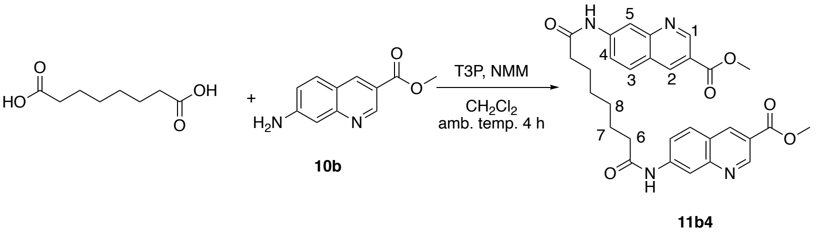

Following general procedure 1: **10b** (116 mg, 0.57 mmol), di-acid (50 mg, 0.28 mmol), NMM (252 μL, 2.30 mmol) and T3P (683 μL, 1.20 mmol) in anhydrous CH_2Cl_2 (4 mL) were reacted according to the general procedure to afford **11b4** (120 mg, 77%) as a yellow solid.

^{1}H NMR (600 MHz, DMSO-d_6) δ (ppm): 10.41 (s, 2H, NH), 9.23 (d, J = 2.2 Hz, 2H, H-1), 8.86 (d, J = 2.2 Hz, 2H, H-2), 8.51 (d, J = 2.1 Hz, 2H, H-5), 8.10 (d, J = 8.9 Hz, 2H, H-3), 7.78 (dd, J = 8.9, 2.1 Hz, 2H, H-4), 3.94 (s, 6H, CH_3), 2.43 (t, J = 7.4 Hz, 4H, H-6), 1.67 (t, J = 7.1 Hz, 4H, H-7), 1.44 – 1.34 (m, 4H, H-8). ^{13}C NMR (150 MHz, DMSO-d_6) δ (ppm): 172.6, 165.8, 150.8, 150.2, 143.0, 138.3, 130.6, 123.1, 121.5, 121.4, 115.6, 52.8, 37.0, 28.9, 25.3. HRMS: m/z: [M+H]$^+$ calcd for $C_{30}H_{31}N_4O_6$ 543.2238; Found 543.2201.

Dimethyl 7,7'-(nonanedioylbis(azanediyl))bis(quinoline-3-carboxylate) (11b5)

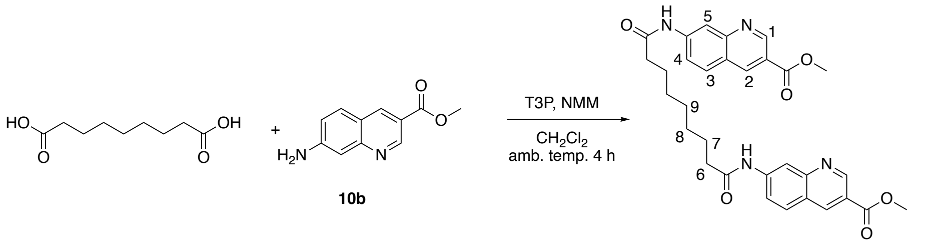

11b5

Following general procedure 1: **10b** (107 mg, 0.52 mmol), di-acid (50 mg, 0.26 mmol), NMM (233 µL, 2.10 mmol) and T3P (632 µL, 1.10 mmol) in anhydrous CH_2Cl_2 (4 mL) were reacted according to the general procedure to afford **11b5** (110 mg, 74%) as a yellow solid.

^{1}H NMR (600 MHz, DMSO-d_6)) δ (ppm): 10.44 (s, 2H, NH), 9.23 (d, J = 2.2 Hz, 2H, H-1), 8.86 (d, J = 2.2 Hz, 2H, H-2), 8.52 (d, J = 2.1 Hz, 2H, H-5), 8.11 (d, J = 8.9 Hz, 2H, H-3), 7.79 (dd, J = 8.9, 2.1 Hz, 2H, H-4), 3.93 (s, 6H, CH_3), 2.42 (t, J = 7.4 Hz, 4H, H-6), 1.69 – 1.61 (m, 4H, H-7), 1.40 – 1.28 (m, 6H, H8 & H9). ^{13}C NMR (151 MHz, DMSO-d_6) δ 172.7, 165.8, 150.8, 150.2, 143.0, 138.3, 130.6, 123.1, 121.5, 121.3, 115.6, 52.8, 37.0, 29.0, 29.0, 25.4. HRMS: *m/z*: $[M+H]^+$ calcd for $C_{31}H_{33}N_4O_6$ 557.2395; Found 557.2245.

6,6'-((2,2'-((*tert*-butoxycarbonyl)azanediyl)bis(acetyl))bis(azanediyl))bis(quinoline-3-carboxylic acid) (12a1)

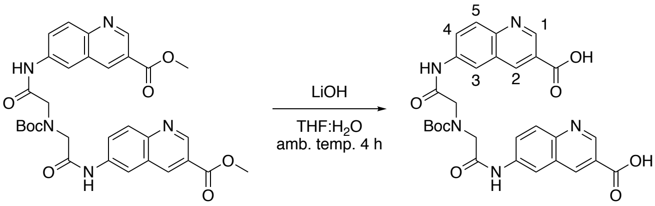

11a1 12a1

Following general procedure 2: **11a1** (241 mg, 0.40 mmol) and aq. LiOH (2.4 mL, 2.4 mmol) in THF: H_2O (8 mL) were reacted according to general procedure 2 to afford **12a1** (201 mg, 88%) as a brown solid.

^{1}H NMR (400 MHz, DMSO-d_6) δ (ppm) 13.48 (s, 2H, OH), 10.94 (s, 2H, NH), 9.21 (d, J = 1.7 Hz, 2H, H-1), 8.92 (d, J = 1.7 Hz, 2H, H-2), 8.56 (dd, J = 4.6, 2.2 Hz, 2H, H-3), 8.16-8.10 (m, 2H, H-5), 8.09-8.03 (m, 2H, H-4), 4.24 (d, J = 9.0 Hz, 4H, CH_2), 1.33 (s, 9H, Boc). ^{13}C NMR (100 MHz, DMSO-d_6) δ (ppm) 169.61, 166.17, 154.74, 148.05, 145.36, 138.59, 137.64, 129.22, 127.32, 125.89, 124.16, 116.14, 80.09, 53.43, 52.79, 27.80; HRMS: *m/z*: $[M+H]^+$ calcd for $C_{29}H_{28}N_5O_8$ 574.1932; Found 574.1942.

7,7'-((3,3'-((*tert*-butoxycarbonyl)azanediyl)bis(propanoyl))bis(azanediyl))bis(quinoline-3-carboxylic acid) (12b1)

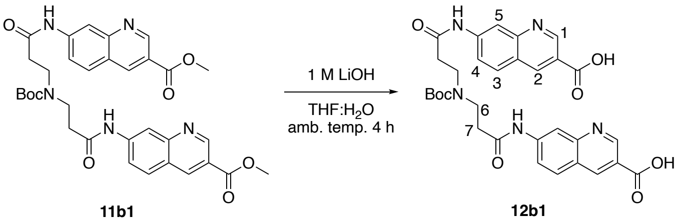

Following general procedure 2: **11b1** (120mg, 0.19 mmol) and aq. LiOH (953 μL, 0.95 mmol) in THF: H_2O (4 mL) were reacted according to general procedure 2 to afford **12b1** (85 mg, 74%) as a beige solid.

^{1}H NMR (600 MHz, DMSO-d_6) δ (ppm): 13.36 (s, 2H, OH), 10.52 (s, 2H, NH), 9.24 (d, J = 2.2 Hz, 2H, H-1), 8.85 (d, J = 2.2 Hz, 2H, H-2), 8.52 (s, 2H, H-5), 8.11 (d, J = 8.9 Hz, 2H, H-3), 7.78 (dd, J = 8.9, 2.1 Hz, 2H, H-4), 3.58-3.50 (m, 4H, H-7), 2.76 – 2.66 (m, 4H, H-6), 1.36 (s, 9H, Boc). ^{13}C NMR (150 MHz, DMSO-d_6) δ (ppm): 170.9, 166.8, 154.9, 150.6, 150.4, 142.7, 138.5, 130.6, 123.3, 122.5, 121.4, 115.6, 79.3, 65.4, 31.2, 28.4. HRMS: *m/z*: [M+H]$^+$ calcd for $C_{31}H_{32}N_5O_8$ 602.2241; Found 602.2206.

6,6'-(adipoylbis(azanediyl))bis(quinoline-3-carboxylic acid) (12a2)

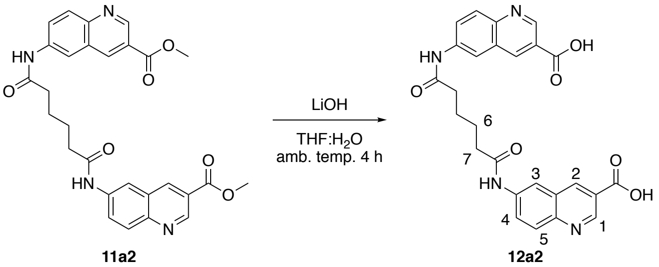

Following general procedure 2: **11a2** (178 mg, 0.35 mmol) and aq. LiOH (2.1 mL, 2.1 mmol) in THF: H_2O (7 mL) were reacted according to general procedure 2 to afford **12a2** (147 mg, 89%) as a beige solid.

^{1}H NMR (400 MHz, DMSO-d_6) δ (ppm) 10.43 (s, 2H, NH), 9.22 – 9.16 (d, J = 1.6 Hz, 2H, H-1), 8.84 (s, 2H, H-2), 8.50 (s, 2H, H-3), 8.05 (d, J = 9.0 Hz, 2H, H-5), 7.99 (d, J = 9.0 Hz, 2H, H-4), 2.46 (m, 4H, H7-), 1.72 (m, 4H, H-6). ^{13}C NMR (100 MHz, DMSO-d_6) δ (ppm) 171.83, 166.25, 147.87, 145.30, 138.27, 128.89, 127.24, 125.96, 124.05, 115.83, 36.32, 24.80; HRMS: *m/z*: [M+H]$^+$ calcd for $C_{26}H_{23}N_4O_6$ 487.1612; Found 487.1617.

7,7'-(adipoylbis(azanediyl))bis(quinoline-3-carboxylic acid) (12b2)

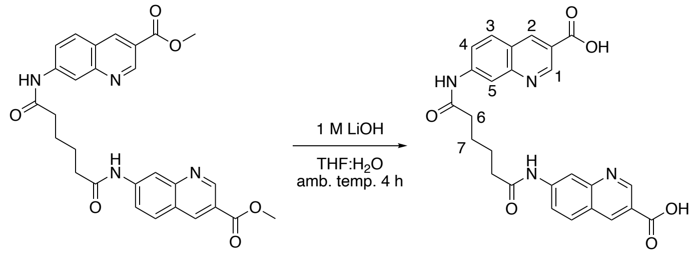

Following general procedure 2: **11b2** (110 mg, 0.21 mmol) and aq. LiOH (1.1 mL, 1.1 mmol) in THF: H_2O (4 mL) were reacted according to general procedure 2 to afford **12b2** (68 mg, 65%) as a beige solid.

^{1}H NMR (600 MHz, DMSO-*d*$_6$) *δ* (ppm): 13.33 (s, 2H, OH), 10.43 (s, 2H, NH), 9.23 (d, *J* = 2.2 Hz, 2H, H-1), 8.83 (d, *J* = 2.2 Hz, 2H, H-2), 8.52 (d, *J* = 2.0 Hz, 2H, H-5), 8.10 (d, *J* = 8.9 Hz, 2H, H-3), 7.79 (dd, *J* = 8.9, 2.1 Hz, 2H, H-4), 2.48 (t, *J* = 6.4 Hz, 4H, H-6), 1.73 (p, *J* = 3.6 Hz, 4H, H-7). ^{13}C NMR (151 MHz, DMSO-*d*$_6$) *δ* (ppm): 172.4, 166.9, 150.8, 150.7, 142.7, 138.3, 130.5, 123.2, 121.4, 115.7, 36.9, 25.2. HRMS: *m/z*: [M+H]$^+$ calcd for $C_{26}H_{23}N_4O_6$ 487.1608; Found 487.1638.

6,6'-(heptanedioylbis(azanediyl))bis(quinoline-3-carboxylic acid) (12a3)

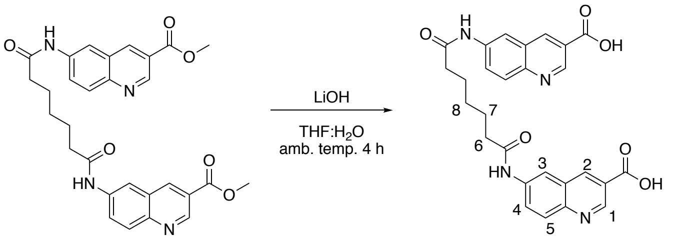

Following general procedure 2: **11a3** (105 mg, 0.20 mmol) and aq. LiOH (1.2 mL, 1.2 mmol) in THF: H_2O (4 mL) were reacted according to general procedure 2 to afford **12a3** (90 mg, 91%) as a pale white solid.

^{1}H NMR (600 MHz, DMSO-*d*$_6$) *δ* (ppm) 10.33 (s, 2H, NH), 9.16 (d, *J* = 2.1 Hz, 2H, H-1), 8.78 (d, *J* = 2.1 Hz, 2H, H-2), 8.46 (d, *J* = 2.3 Hz, 2H, H3-), 8.02 (d, *J* = 9.0 Hz, 2H, H-5), 7.95 (dd, *J* = 9.0, 2.3 Hz, 2H, H-4), 2.42 (t, *J* = 7.4 Hz, 4H, H-6), 1.69 (app. p, *J* = 7.5 Hz, 4H, H-7), 1.42 (app. h, *J* = 7.4, 6.6 Hz, 2H, H-8). ^{13}C NMR (151 MHz, DMSO-*d*$_6$) *δ* (ppm) 171.85, 166.38, 148.15, 145.97, 138.02, 137.66, 129.36, 127.09, 125.63, 123.93, 115.75, 36.31, 28.31, 24.84; HRMS: *m/z*: [M+H]$^+$ calcd for $C_{27}H_{25}N_4O_6$ 501.1769; Found 501.1773.

7,7'-(heptanedioylbis(azanediyl))bis(quinoline-3-carboxylic acid) (12b3)

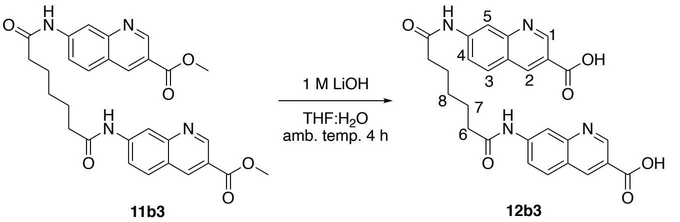

Following general procedure 2: **11b3** (120mg, 0.22 mmol) and aq. LiOH (1.1 mL, 1.1 mmol) in THF: H_2O (4 mL) were reacted according to general procedure 2 to afford **12b3** (95 mg, 84%) as a beige solid.

^{1}H NMR (400 MHz, DMSO-d_6) δ (ppm): 13.37 (s, 2H, OH), 10.46 (s, 2H, NH), 9.32 – 9.17 (m, 2H, H-1), 8.88 (s, 2H, H-2), 8.54 (s, 2H, H-5), 8.12 (d, J = 8.9 Hz, 2H, H-3), 7.79 (d, J = 8.9 Hz, 2H, H-4), 2.46 (t, J = 6.8 Hz, 4H, H-6), 1.70 (p, J = 7.6 Hz, 4H, H-7), 1.43 (p, J = 7.2 Hz, 2H, H-8). ^{13}C NMR (150 MHz, DMSO-d_6) δ (ppm): 172.6, 166.7, 150.4, 150.1, 143.0, 138.8, 130.6, 123.3, 122.5, 121.5, 115.1, 36.9, 28.7, 25.2. HRMS: m/z: [M+H]$^+$ calcd for $C_{27}H_{25}N_4O_6$ 501.1769; Found 501.1717

6,6'-(octanedioylbis(azanediyl))bis(quinoline-3-carboxylic acid) (12a4)

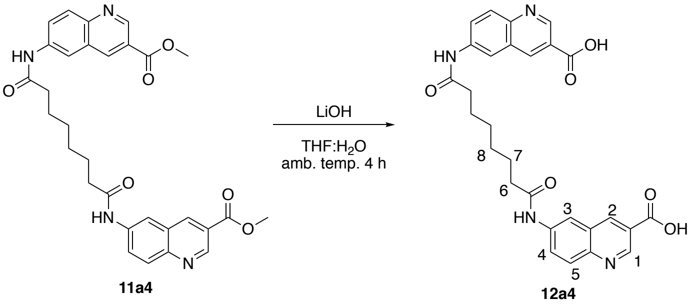

Following general procedure 2: **11a4** (138 mg, 0.24 mmol) and aq. LiOH (0.72 mL, 1.44 mmol) in THF: H_2O (5 mL) were reacted according to general procedure 2 to afford **12a4** (115 mg, 93%) as a pale white solid.

^{1}H NMR (600 MHz, DMSO-d_6) δ (ppm) 10.32 (s, 2H, NH), 9.16 (d, J = 2.3 Hz, 2H, H-1), 8.79 (d, J = 2.3 Hz, 2H, H-2), 8.46 (d, J = 2.4 Hz, 2H, H-3), 8.02 (d, J = 9.1 Hz, 2H, H-5), 7.95 (dd, J = 9.1, 2.4 Hz, 2H, H-4), 2.40 (t, J = 7.4 Hz, 4H, H-6), 1.66 (app. h, J = 7.6, 7.1 Hz, 4H, H-7), 1.42 – 1.37 (m, 4H, H-8). ^{13}C NMR (151 MHz, DMSO-d_6) δ (ppm) 171.87, 166.40, 148.17, 145.95, 138.02, 137.63, 129.35, 127.10, 125.61, 124.04, 115.75, 36.40, 28.51, 24.95; HRMS: m/z: [M+H]$^+$ calcd for $C_{28}H_{27}N_4O_6$ 515.1925; Found 515.1932.

7,7'-(octanedioylbis(azanediyl))bis(quinoline-3-carboxylic acid) (12b4)

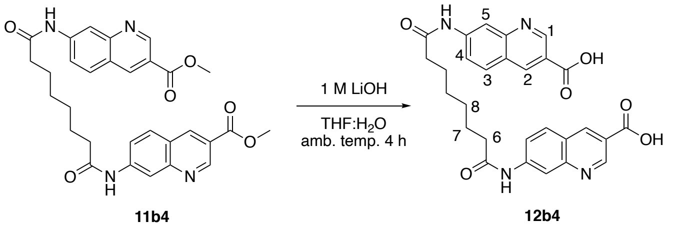

Following general procedure 2: **11b4** (85 mg, 0.16 mmol) and aq. LiOH (0.8 mL, 0.8 mmol) in THF: H_2O (4 mL) were reacted according to general procedure 2 and afforded **12b4** (79 mg, 98%) as orange solid.

^{1}H NMR (600 MHz, DMSO-d_6) δ (ppm): 13.39 (s, 2H, OH), 10.43 (s, 2H, NH), 9.24 (d, J = 2.1 Hz, 2H, H-1), 8.86 (d, J = 2.1 Hz, 2H, H-2), 8.53 (d, J = 2.0 Hz, 2H, H-5), 8.10 (d, J = 8.9 Hz, 2H, H-3), 7.79 (dd, J = 8.9, 2.0 Hz, 2H, H-4), 2.43 (t, J = 7.5 Hz, 4H, H-6), 1.67 (t, J = 7.1 Hz, 4H, H-7), 1.40 (p, J = 3.8 Hz, 4H, H-8). ^{13}C NMR (150 MHz, DMSO-d_6) δ (ppm): 172.6, 166.8, 150.5, 150.3, 142.9, 138.7, 130.6, 123.2, 122.4, 121.5, 115.3, 37.0, 28.9, 25.3. HRMS: *m/z*: [M+H]$^+$ calcd for $C_{28}H_{27}N_4O_6$ 515.1925; Found 515.1917.

7,7'-(nonanedioylbis(azanediyl))bis(quinoline-3-carboxylic acid) (12b5)

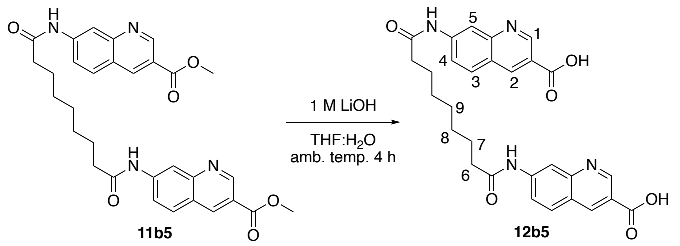

Following general procedure 2: **11b5** (100 mg, 0.18 mmol) and aq. LiOH (0.9 mL, 0.9mmol) in THF: H_2O (4 mL) were reacted according to general procedure 2 and afforded **12b5** (78 mg, 82%) as beige solid.

^{1}H NMR (600 MHz, DMSO-d_6) δ (ppm): 13.45 (s, 2H, OH), 10.48 (s, 2H, NH), 9.27 (s, 2H, H-1), 8.92 (s, 2H, H-2), 8.57 (s, 2H, H-5), 8.14 (d, J = 8.8 Hz, 2H, H-3), 7.80 (d, J = 8.8 Hz, 2H, H-4), 2.43 (t, J = 7.5 Hz, 4H, H-6), 1.73 – 1.59 (m, 4H, H-7), 1.37-1.32 (m, J = 31.1 Hz, 6H, H-8 & H-9). ^{13}C NMR (150 MHz, DMSO-d_6) δ (ppm): 172.8, 166.6, 150.1, 149.5, 143.2, 139.3, 130.7, 123.4, 122.5, 121.7, 114.6, 37.1, 29.1, 29.0, 25.4. HRMS: *m/z*: [M+H]$^+$ calcd for $C_{29}H_{29}N_4O_6$ 529.2082; Found 529.2045.

tert-butyl 5,8,12,15-tetraoxo-11_H_,31_H_-4,7,10,13,16-pentaaza-6(3,6),14(6,3)-diquinolina-1,3(3,5)-diindolacyclohexadecaphane-10-carboxylate (13a1)

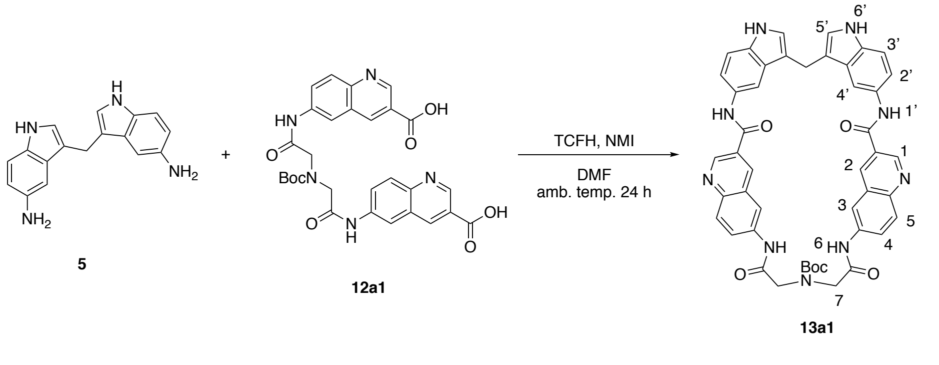

Following general procedure 3: **12a1** (50.0 mg, 0.087 mmol), TCFH (98.0 mg, 0.349 mmol), NMI (56 μL, 0.70 mmol) and **5** (28.9 mg, 0.105 mmol) were reacted according to general procedure 3. Purification by flash column chromatography (eluent: 6% → 10% CH_3OH in CH_2Cl_2) afforded **13a1** (16 mg, 23%) as a pale white solid.

^{1}H NMR (400 MHz, DMSO-d_6) δ (ppm) 11.30 (s, 1H, H-6'), 11.24 (s, 1H, H-6'), 10.82 (s, 2H, H-1'), 10.42 (s, 2H, H-6), 9.28 (d, J = 2.1 Hz, 2H, H-1), 9.07 (d, J = 2.1 Hz, 2H, H-2), 9.05 (d, J = 2.1 Hz, 1H, H-2), 8.49 (d, J = 2.4 Hz, 2H, H-3), 8.28 (dd, J = 9.0, 2.3 Hz, 2H, H-4) 8.17 – 8.10 (m, 4H, H-4' & H-5), 7.32 (dd, J = 8.4, 1.4 Hz, 2H, H-2'), 7.21 (s, 2H, H-5'), 7.18 (d, J = 8.4 Hz, 2H, H-3'), 4.27 (s, 4H, H-7), 4.08 (s, 2H, CH_2), 1.34 (s, 9H, Boc). ^{13}C NMR (151 MHz, DMSO-d_6) δ (ppm) 170.22, 169.95, 163.70, 154.44, 147.57, 145.48, 137.48, 137.43, 135.92, 133.36, 133.32, 129.99, 129.92, 129.74, 129.66, 128.76, 128.74, 127.30, 127.27, 126.84, 124.47, 124.35, 123.80, 123.78, 116.36, 116.29, 115.78, 115.71, 115.45, 115.44, 111.41, 111.28, 111.10, 80.46, 54.41, 53.69, 27.71, 19.33; HRMS: m/z: $[M+H]^+$ calcd for $C_{46}H_{40}N_9O_6$ 814.3096; Found 814.3121.

tert-butyl 5,8,14,17-tetraoxo-11H,31H-4,7,11,15,18-pentaaza-6(3,7),16(7,3)-diquinolina-1,3(3,5)-diindolacyclooctadecaphane-11-carboxylate (13b1)

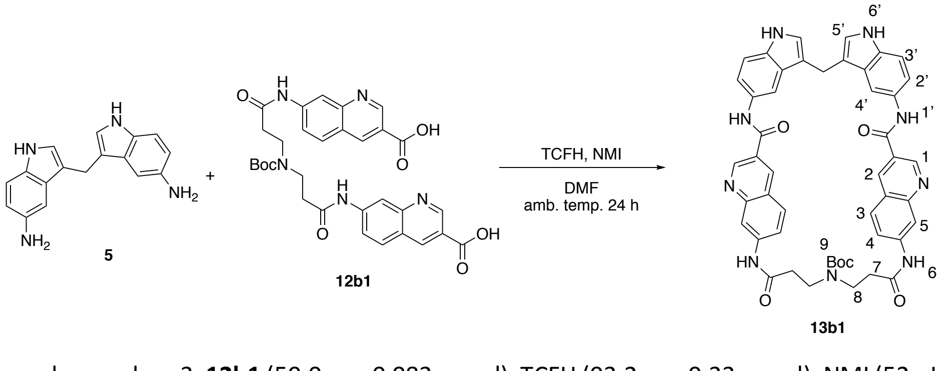

Following general procedure 3: **12b1** (50.0 mg, 0.083 mmol), TCFH (93.2 mg, 0.33 mmol), NMI (52 μL, 0.66 mmol) and **5** (25.2 mg, 0.914 mmol) were reacted according to general procedure 3. Purification by flash column chromatography (eluent: 6% → 10% CH_3OH in CH_2Cl_2) afforded **13b1** (15 mg, 21%) as a pale yellow solid.

^{1}H NMR (600 MHz, DMSO-d_6) δ (ppm) 10.79 (d, J = 2.4 Hz, 2H, H-6'), 10.32 (s, 2H, H-1'), 10.12 (s, 2H, H-6), 8.92 (s, 2H, H-1), 8.46 (s, 2H, H-2), 8.30 (s, 2H, H-5), 8.15 (s, 2H, H-4'), 7.50 (s, 2H, H-4), 7.33 – 7.27 (m, 4H, H-3', H-5'),

7.24 (br, 4H, H-3), 4.10 (s, 2H, CH_2), 3.62 (t, *J* = 6.1 Hz, 4H, H-7), 2.67 (t, *J* = 6.2 Hz, 5H, H-8), 1.48 (s, 9H, Boc). ^{13}C NMR (151 MHz, DMSO-d_6) δ (ppm) 164.84, 160.89, 155.25, 149.16, 146.52, 141.28, 138.55, 134.98, 133.77, 130.36, 128.84, 127.03, 126.33, 124.09, 122.61, 120.59, 117.32, 116.08, 112.33, 111.31, 79.35, 31.76, 29.48, 28.61, 22.57. HRMS: *m/z*: $[M+H]^+$ calcd for $C_{48}H_{44}N_9O_6$ 842.3409; Found: 842.3411.

1[1]*H*,3[1]*H*-4,7,14,17-tetraaza-6(3,6),15(6,3)-diquinolina-1,3(3,5)-diindolacycloheptadecaphane-5,8,13,16-tetraone (13a2)

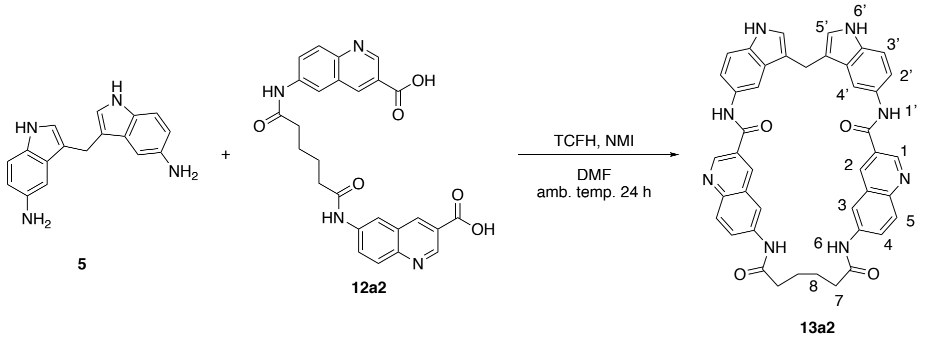

Following general procedure 3: **12a2** (60 mg, 0.12 mmol), TCFH (138 mg, 0.492 mmol), NMI (80 μL, 1.0 mmol) and **5** (41.0 mg, 0.148 mmol) were reacted according to general procedure 3. Purification by flash column chromatography (eluent: 6% → 12% CH_3OH in CH_2Cl_2) afforded **13a2** (19 mg, 21%) as a pale yellow solid.

^1H NMR (600 MHz, DMSO-d_6) *δ* (ppm) 10.86 (s, 2H, H-6'), 10.42 (s, 2H, H-1'), 10.33 (s, 2H, H-6), 9.14 (d, *J* = 2.2 Hz, 2H, H-1), 8.78 (d, *J* = 2.2 Hz, 2H, H-2), 8.51 (d, *J* = 2.1 Hz, 2H, H-3), 7.94 (d, *J* = 9.0 Hz, 2H, H-5), 7.80 (dd, *J* = 9.0, 2.1 Hz, 2H, H-4), 7.78 (s, 2H, H-4'), 7.75 (d, *J* = 8.6 Hz, 2H, H-2'), 7.35 (d, *J* = 8.6 Hz, 2H, H-3'), 7.25 (d, *J* = 2.3 Hz, 2H, H-5'), 4.11 (s, 2H, CH_2), 2.45 (t, *J* = 5.7 Hz, 4H, H-7), 1.77 (t, *J* = 5.7 Hz, 4H, H-8). ^{13}C NMR (151 MHz, DMSO-d_6) *δ* (ppm) 171.77, 163.73, 147.56, 145.14, 137.83, 134.71, 133.51, 130.32, 129.10, 127.96, 126.91, 126.77, 124.68, 123.90, 115.84, 115.65, 114.03, 111.01, 110.54, 36.22, 24.60, 19.66; HRMS: *m/z*: $[M+H]^+$ calcd for $C_{43}H_{35}N_8O_4$ 727.2776; Found 727.2786.

1[1]H,3[1]H-4,7,14,17-tetraaza-6(3,7),15(7,3)-diquinolina-1,3(3,5)-diindolacycloheptadecaphane-5,8,13,16-tetraone (13b2)

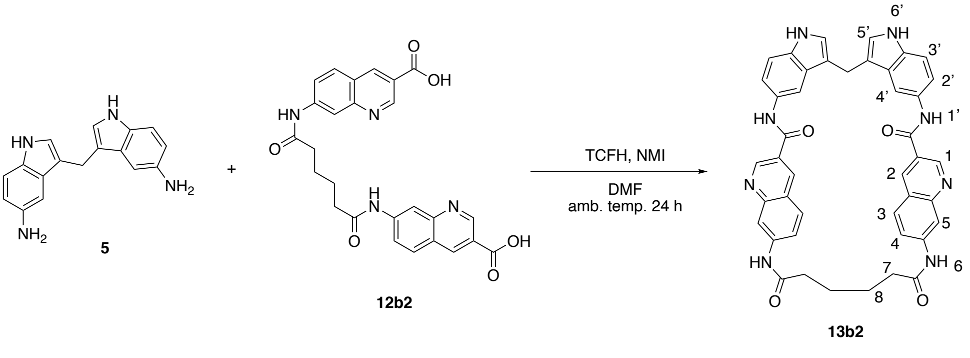

Following general procedure 3: **12b2** (50.0 mg, 0.11 mmol), TCFH (115 mg, 0.41 mmol), NMI (65 μL, 0.82 mmol) and **5** (31 mg, 0.11 mmol) were reacted according to general procedure 3. Purification by flash column chromatography (eluent: 5% → 12% CH_3OH in CH_2Cl_2) afforded **13b2** (16 mg, 21%) as a beige solid.

^{1}H NMR (600 MHz, DMSO-d_6) δ (ppm): 10.78 (d, J = 2.3 Hz, 2H, H-6'), 10.47 (s, 2H, H-1'), 9.98 (s, 2H, H-6), 9.04 (d, J = 2.2 Hz, 2H, H-1), 8.54 (s, 2H, H-2), 8.17 (s, 2H, H-5), 8.08 (s, 2H, H-4'), 7.52 (d, J = 8.5 Hz, 2H, H-4), 7.32 – 7.28 (m, 4H, H-5', H-2'), 7.23 (dd, J = 8.8, 2.0 Hz, 2H, H-3'), 7.07 (d, J = 8.8 Hz, 2H, H-3), 4.11 (s, 2H, CH_2), 2.44 (d, J = 5.6 Hz, 4H, H-7), 1.89 (s, 4H, H-8). ^{13}C NMR (150 MHz, DMSO-d_6) δ (ppm): 172.4, 164.9, 149.5, 149.0, 141.6, 135.1, 133.8, 130.5, 128.4, 127.1, 126.2, 124.1, 122.4, 120.5, 117.2, 116.0, 114.9, 112.4, 111.2, 36.9, 25.2, 24.3. HRMS: *m/z*: [M+H]$^+$ calcd for $C_{43}H_{35}N_8O_4$ 727.2781; Found 727.2799.

11*H*,31*H*-4,7,15,18-tetraaza-6(3,6),16(6,3)-diquinolina-1,3(3,5)-diindolacyclooctadecaphane-5,8,14,17-tetraone (13a3)

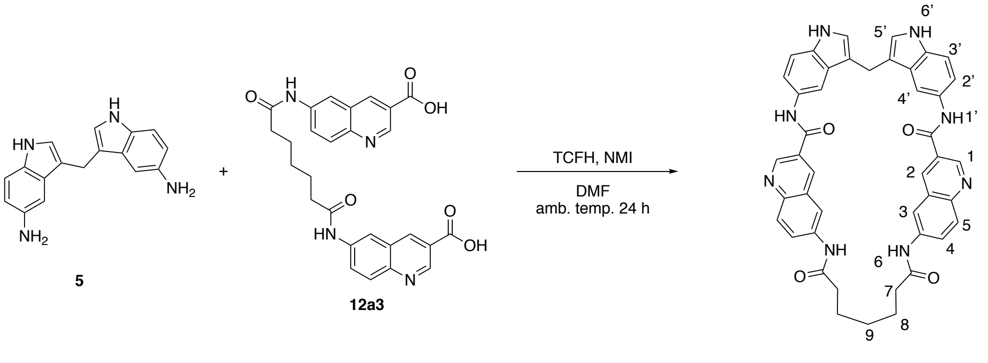

Following general procedure 3: **12a3** (60.0 mg, 0.120 mmol), TCFH (135 mg, 0.481 mmol), NMI (77 μL, 0.97 mmol) and **5** (39.8 mg, 0.144 mmol) were reacted according to general procedure 3. Purification by flash column chromatography (eluent: 6% → 12% CH_3OH in CH_2Cl_2) afforded **13a3** (18 mg, 20%) as a pale brown solid.

^{1}H NMR (600 MHz, DMSO-d_6) δ (ppm) 10.82 (s, 2H, H-6'), 10.37 (s, 2H, H-1'), 10.23 (s, 2H, H-6), 9.10 (d, J = 2.1 Hz, 2H, H-1), 8.68 (d, J = 2.1 Hz, 2H, H-2), 8.42 (d, J = 2.2 Hz, 2H, H-3), 7.92 (s, 2H, H-4'), 7.89 (d, J = 9.0 Hz, 2H, H-5), 7.76 (dd, J = 9.0, 2.2 Hz, 2H, H-4), 7.51 (d, J = 8.6 Hz, 2H, H-2'), 7.33 (d, J = 8.6 Hz, 2H, H-3'), 7.20 (d, J = 2.3 Hz, 2H, H-5'), 4.09 (s, 2H, CH_2), 2.42 (t, J = 6.4 Hz, 4H, H-7), 1.69 (app. p, J = 7.0 Hz, 4H, H-8), 1.45 (app. p, J = 7.7 Hz, 2H, H-9). ^{13}C NMR (151 MHz, DMSO-d_6) δ (ppm) 171.76, 163.83, 147.29, 145.09, 137.76, 134.78, 133.51, 130.11, 129.11, 128.11, 126.83, 126.80, 124.49, 123.87, 116.12, 115.14, 114.84, 111.08, 111.03, 36.14, 27.94, 24.72, 19.67; HRMS: *m/z*: [M+H]$^+$ calcd for $C_{44}H_{37}N_8O_4$ 741.2932; Found 741.2940.

1^1H,3^1H-4,7,15,18-tetraaza-6(3,7),16(7,3)-diquinolina-1,3(3,5)-diindolacyclooctadecaphane-5,8,14,17-tetraone (13b3)

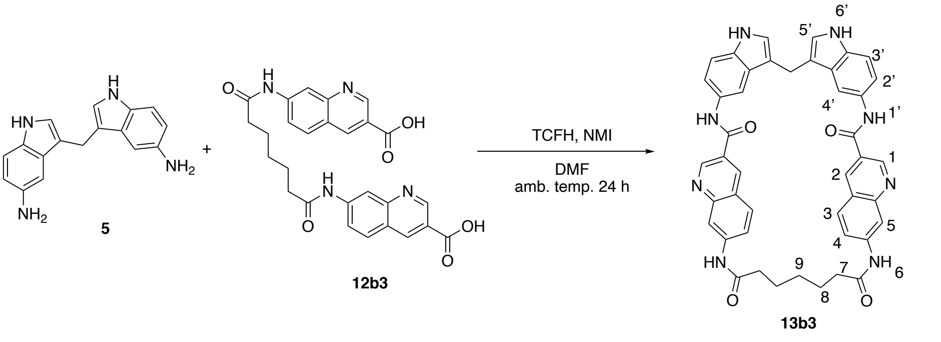

Following general procedure 3: **12b3** (50.0 mg, 0.10 mmol), TCFH (112 mg, 0.41 mmol), NMI (64 μL, 0.80 mmol) and **5** (30 mg, 0.11 mmol) were reacted according to general procedure 3. Purification by flash column chromatography (eluent: 5% → 12% CH_3OH in CH_2Cl_2) afforded **13b3** (15 mg, 20%) as a pale orange solid.

^{1}H NMR (600 MHz, DMSO-d_6) δ (ppm): 10.79 (s, 2H, H-6'), 10.42 (s, 2H, H-1'), 10.18 (s, 2H, H-6), 9.13 (s, 2H, H-1), 8.51 (s, 2H, H-2), 8.42 (s, 2H, H-5), 8.15 (s, 2H, H-4´), 7.47 (d, J = 8.6 Hz, 2H, H-4), 7.35 – 7.26 (m, 6H, H-5', H-2´, H-3´), 7.18 (d, J = 8.8 Hz, 2H, H-3), 4.10 (s, 2H, CH_2), 2.38 (d, J = 6.9 Hz, 4H, H-7), 1.74 (q, J = 6.7 Hz, 4H, H-8), 1.42 – 1.39 (m, 2H, H-9). ^{13}C NMR (151 MHz, DMSO-d_6) δ (ppm): 172.3, 164.6, 149.6, 149.5, 141.4, 135.2, 133.7, 130.5, 129.0, 127.0, 126.6, 124.1, 122.8, 120.9, 117.4, 116.3, 115.7, 112.3, 111.3, 40.5, 40.4, 40.3, 40.1, 40.0, 39.9, 39.7, 39.6, 36.6, 26.7, 25.2, 20.3. HRMS: *m/z*: $[M+H]^+$ calcd for $C_{44}H_{37}N_8O_4$ 741.2938; Found 741.2959.

11*H*,31*H*-4,7,16,19-tetraaza-6(3,6),17(6,3)-diquinolina-1,3(3,5)-diindolacyclononadecaphane-5,8,15,18-tetraone (13a4)

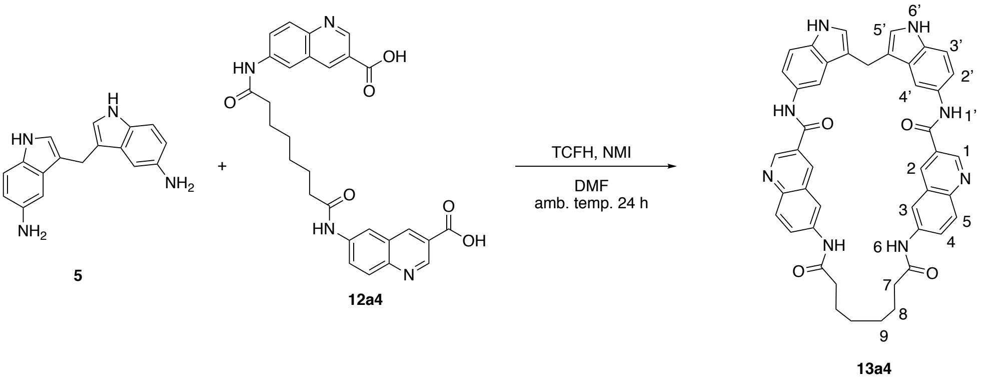

Following general procedure 3: **12a4** (94.0 mg, 0.183 mmol), TCFH (205 mg, 0.730 mmol), NMI (117 μL, 1.46 mmol) and **5** (60.6 mg, 0.219 mmol) were reacted according to general procedure 3. Purification by flash column chromatography (eluent: 6% → 12% CH_3OH in CH_2Cl_2) afforded **13a4** (29 mg, 21%) as a pale brown solid.

^{1}H NMR (600 MHz, DMSO-d_6) δ (ppm) 10.83 (s, 2H, H-6'), 10.43 (s, 2H, H-1'), 10.27 (s, 2H, H6), 9.13 (d, *J* = 2.1 Hz, 2H, H-1), 8.77 (d, *J* = 2.1 Hz, 2H, H-2), 8.41 (s, 2H, H-3), 7.94 (d, *J* = 9.1 Hz, 2H, H-5), 7.92 (s, 2H, H-4'), 7.84 (dd, *J* = 9.1, 2.2 Hz, 2H, H-4), 7.62 (d, *J* = 8.6 Hz, 2H, H-2'), 7.35 (d, *J* = 8.6 Hz, 2H, H-3'), 7.16 (s, 2H, H-5'), 4.13 (s, 2H,

CH_2), 2.40 (t, *J* = 7.0 Hz, 4H, H-7), 1.69 (app. t, *J* = 7.6 Hz, 4H, H-8), 1.40 (app. d, *J* = 6.3 Hz, 4H, H-9). ^{13}C NMR (151 MHz, DMSO-d_6) δ (ppm) 171.82, 163.78, 147.44, 145.11, 137.77, 134.77, 133.60, 130.25, 129.10, 128.00, 126.84, 126.79, 124.55, 123.91, 115.84, 115.50, 114.38, 111.06, 110.78, 36.04, 28.15, 24.66, 19.96; HRMS: *m/z*: $[M+H]^+$ calcd for $C_{45}H_{39}N_8O_4$ 755.3089; Found 755.3098.

1^1*H*,3^1*H*-4,7,16,19-tetraaza-6(3,7),17(7,3)-diquinolina-1,3(3,5)-diindolacyclononadecaphane-5,8,15,18-tetraone (13b4)

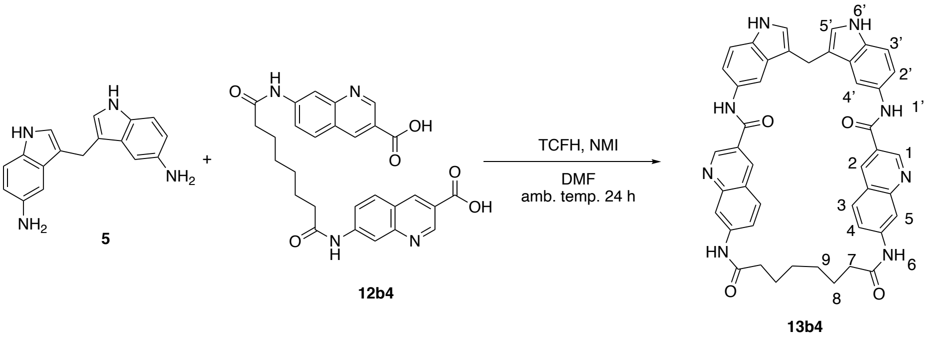

Following general procedure 3: **12b4** (50.0 mg, 0.10 mmol), TCFH (109 mg, 0.40 mmol), NMI (62 μL, 0.80 mmol) and **5** (29 mg, 0.11 mmol) were reacted according to general procedure 3. Purification by flash column chromatography (eluent: 5% → 12% CH_3OH in CH_2Cl_2) afforded **13b4** (20 mg, 27%) as a pale-yellow solid**.**

^1H NMR (600 MHz, DMSO-d_6) δ (ppm): 10.79 (d, *J* = 2.4 Hz, 2H, H-6´), 10.49 (s, 2H, H-1´), 10.10 (s, 2H, H-6), 9.17 (d, *J* = 2.2 Hz, 2H, H-1), 8.50 (s, 2H, H-2), 8.24 (s, 2H, H-5), 8.21 (d, *J* = 2.2 Hz, 2H, H-4´), 7.55 (dd, *J* = 8.6, 1.9 Hz, 2H, H-4), 7.31 (d, *J* = 8.6 Hz, 2H, H-3), 7.29 (d, *J* = 2.3 Hz, 2H, H-5´), 7.19 (dd, *J* = 8.9, 2.0 Hz, 2H, H-2´), 7.01 (d, *J* = 8.7 Hz, 2H, H-3´), 4.11 (s, 2H, CH_2), 2.38 (t, *J* = 9.1 Hz, 4H, H-7), 1.72 (t, *J* = 9.1 Hz, 4H, H-8), 1.42 – 1.39 (m, 4H, H-9). ^{13}C NMR (151 MHz, DMSO-d_6) δ (ppm): 172.1, 164.7, 149.6, 149.3, 141.4, 135.4, 133.8, 130.4, 128.7, 127.1, 126.2, 124.1, 122.5, 120.5, 117.2, 116.1, 115.1, 112.4, 111.3, 36.0, 31.2, 27.9, 25.0. HRMS: *m/z*: $[M+H]^+$ calcd for $C_{45}H_{39}N_8O_4$ 755.3094; Found 755.3119.

1^1H,3^1H-4,7,17,20-tetraaza-6(3,7),18(7,3)-diquinolina-1,3(3,5)-diindolacycloicosaphane-5,8,16,19-tetraone (13b5)

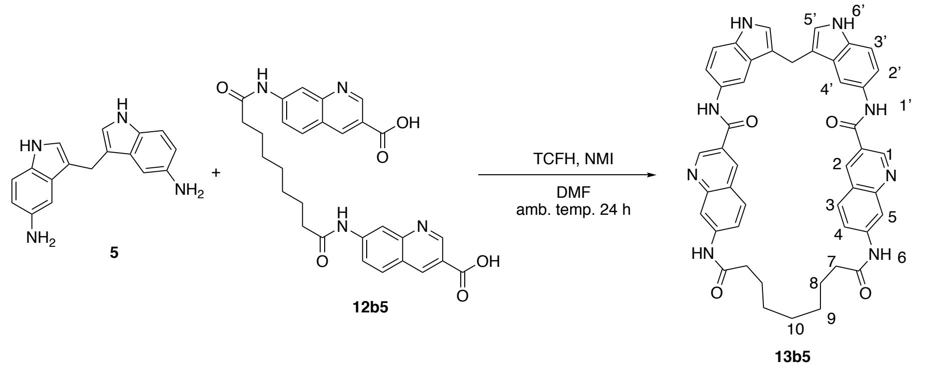

Following general procedure 3: **12b5** (50.0 mg, 0.10 mmol), TCFH (109 mg, 0.40 mmol), NMI (61 µL, 0.80 mmol) and **5** (28 mg, 0.11 mmol) were reacted according to general procedure 3. Purification by flash column chromatography (eluent: 5% → 10% CH_3OH in CH_2Cl_2) afforded **13b5** (17 mg, 23%) as a beige solid.

^{1}H NMR (400 MHz, DMSO-d_6) δ (ppm): 10.81 (s, 2H, H-6´), 10.34 (s, 2H, H-1´), 10.20 (s, 2H, H-6), 9.22 (d, J = 2.2 Hz, 2H, H-1), 8.52 (d, J = 2.3 Hz, 2H, H-2), 8.32 (m, 4H, H-5, H-4'), 7.48-7.35 (m, 6H, H-4, H-3', H-5'), 7.35 – 7.26 (m, 4H, H-3, H-2'), 4.09 (s, 2H, CH_2), 2.38 (t, J = 6.8 Hz, 4H, H-7), 1.67 (s, 4H, H-8), 1.37 (s, 6H, H-9). ^{13}C NMR (151 MHz,) δ (ppm) 172.50, 162.31, 150.09, 149.23, 148.78, 141.76, 135.26, 130.50, 129.50, 127.98, 126.60, 125.39, 124.16, 122.95, 122.61, 121.17, 117.04, 112.09, 111.30, 36.47, 31.76, 29.46, 27.65, 22.56. HRMS: *m/z* calcd for $C_{46}H_{41}N_8O_4$ $[M+H]^+$ calculated: 769.3251; obtained 769.3276.

10-(*tert*-butoxycarbonyl)-6^1,14^1-dimethyl-5,8,12,15-tetraoxo-1^1*H*,3^1*H*-4,7,10,13,16-pentaaza-6(3,6),14(6,3)-diquinolin-1-iuma-1,3(3,5)-diindolacyclohexadecaphane-6^1,14^1-diium (14a1)

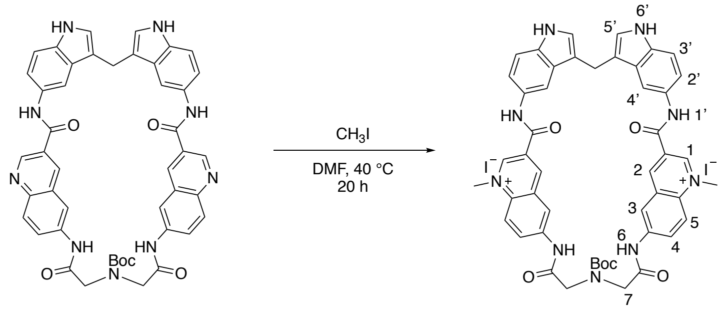

13a1 **14a1**

Following general procedure 4: **13a1** (16.0 mg, 19.7 µmol) and CH_3I (25 µL, 0.40 mmol) in DMF (4 mL) were reacted according to general procedure 4. Compound **14a1** was obtained (19 mg, 88%) after workup as an orange solid.

^{1}H NMR (600 MHz, DMSO-d_6) δ (ppm) 11.62 (s, 1H, H-6'), 11.52 (s, 1H, H-6'), 10.91 (s, 2H, H-1'), 10.69 (s, 1H, H-6), 10.68 (s, 1H, H-6), 9.97 (d, J = 10.5 Hz, 2H, H-2), 9.93 (s, 2H, H-1), 9.01 (s, 1H, H-3), 8.92 (s, 1H, H-3), 8.75 (d, J = 6.0 Hz, 1H, H-5), 8.69-8.62 (m, 3H, H-5 & H-4), 8.25 (s, 1H, H-4'), 8.21 (s, 1H, H-4'), 7.37 (d, J = 8.5 Hz, 2H, H-2'), 7.26 (s, 2H, H-5'), 7.11 (d, J = 8.5 Hz, 2H, H-3'), 4.71 (s, 6H, CH_3), 4.33 (app. d, J = 8.0 Hz, 4H, H-7), 4.10 (s, 2H, CH_2), 1.31 (s, 9H, Boc). ^{13}C NMR (151 MHz, DMSO-d_6) δ (ppm) 171.01, 170.92, 160.24, 160.20, 154.33, 147.66, 147.59, 145.62, 145.57, 139.99, 139.86, 135.35, 135.34, 133.51, 133.49, 129.57, 129.55, 129.43, 129.36, 128.95, 128.64, 126.87, 124.18, 120.94, 120.75, 116.75, 116.66, 115.65, 115.56, 115.45, 111.48, 111.02, 110.87, 80.67, 54.45, 53.70, 45.64, 27.68, 19.31; HRMS: *m/z*: $[M-H]^+$ calcd for $C_{48}H_{44}N_9O_6^+$ 842.3409; Found 842.3432.

6^1,15^1-dimethyl-5,8,13,16-tetraoxo-1^1*H*,3^1*H*-4,7,14,17-tetraaza-6(3,6),15(6,3)-diquinolin-1-iuma-1,3(3,5)-diindolacycloheptadecaphane-6^1,15^1-diium (14a2)

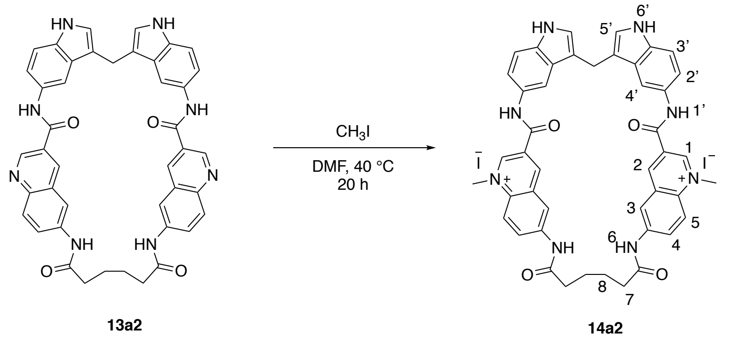

Following general procedure 4: **13a2** (12.0 mg, 16.5 µmol) and CH_3I (21 µL, 0.34 mmol) in DMF (3 mL) were reacted according to general procedure 4. Compound **14a2** was obtained (16 mg, 96%) after workup as an orange solid.

^{1}H NMR (600 MHz, DMSO-*d*$_6$) *δ* (ppm) 10.96 (s, 2H, H-6'), 10.78 (s, 2H, H-1'), 10.76 (s, 2H, H-6), 9.76 (d, *J* = 1.7 Hz, 2H, H-1), 9.69 (d, J = 1.7 Hz, 2H, H-2), 9.02 (d, *J* = 2.4 Hz, 2H, H-3), 8.50 (d, *J* = 9.4 Hz, 2H, H-5), 8.14 (dd, *J* = 9.5, 2.4 Hz, 2H, H-4), 7.79 (dd, *J* = 8.7, 1.9 Hz, 2H, H-2'), 7.74 (d, *J* = 1.9 Hz, 2H, H-4'), 7.43 (d, *J* = 8.7 Hz, 2H, H-3'), 7.30 (d, *J* = 2.4 Hz, 2H, H-5'), 4.66 (s, 6H, CH_3), 4.14 (s, 2H, CH_2), 2.50 (as confirmed by HSQC and HMBC) (app. s, 4H, H-7), 1.78 (app. s, 4H, H-8). ^{13}C NMR (151 MHz, DMSO-*d*$_6$) *δ* (ppm) 172.47, 160.25, 147.90, 143.93, 140.43, 134.84, 133.82, 129.68, 129.16, 129.02, 128.71, 126.93, 124.29, 120.25, 116.37, 115.57, 113.68, 111.35, 110.41, 45.31, 36.12, 24.22, 19.73; HRMS: *m/z*: $[M-H]^+$ calcd for $C_{45}H_{39}N_8O_4^+$ 755.3089; Found 755.3110.

11-(*tert*-butoxycarbonyl)-6^1,16^1-dimethyl-5,8,14,17-tetraoxo-1^1*H*,3^1*H*-4,7,11,15,18-pentaaza-6(3,7),16(7,3)-diquinolin-1-iuma-1,3(3,5)-diindolacyclooctadecaphane-6^1,16^1-diium (14b1)

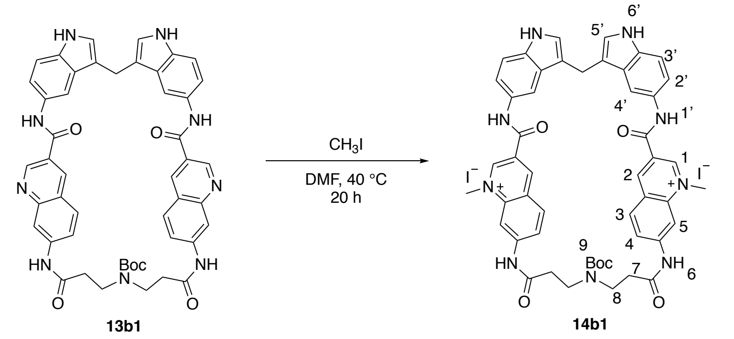

Following general procedure 4: **13b1** (12.0 mg, 14.3 µmol) and CH_3I (18 µL, 0.28 mmol) in DMF (3 mL) were reacted according to general procedure 4. Compound **14b1** was obtained (12 mg, 96%) after workup as an orange solid.

^{1}H NMR (600 MHz, DMSO-*d*$_6$) *δ* (ppm) 11.27 (s, 2H, H-6´), 10.90 (s, 2H, H-1), 10.61 (s, 2H´, H-6), 9.70 (s, 2H, H-1), 9.29 (s, 2H, H-2), 8.52-8.54 (m, 4H, H-5, H-4'), 7.95 (d, *J* = 5.9 Hz, 2H, H-2'), 7.63 (d, *J* = 5.9 Hz, 2H, H-4), 7.37 (m,

6H, H-3´,H-3, H-5'), 4.17 (s, 6H, CH_3), 4.10 (s, 2H, CH_2), 3.44 (s, 4H, H-7), 3.00 (s, 4H, H-8), 1.48 (s, 9H, Boc). ^{13}C NMR (151 MHz, DMSO-d_6) δ (ppm) 173.68, 161.16, 149.89, 146.40, 144.11, 140.04, 134.10, 131.67, 129.59, 129.06, 127.08, 126.86, 124.62, 124.03, 123.03, 117.17, 115.72, 112.52, 111.71, 103.31, 80.26, 45.31, 31.16, 29.01, 27.14. HRMS: *m/z*: [M-H]$^+$ calcd for $C_{50}H_{48}N_9O_6^+$ 870.3717; Found 870.3732.

6^1,15^1-dimethyl-5,8,13,16-tetraoxo-1^1*H*,3^1*H*-4,7,14,17-tetraaza-6(3,7),15(7,3)-diquinolin-1-iuma-1,3(3,5)-diindolacycloheptadecaphane-6^1,15^1-diium (14b2)

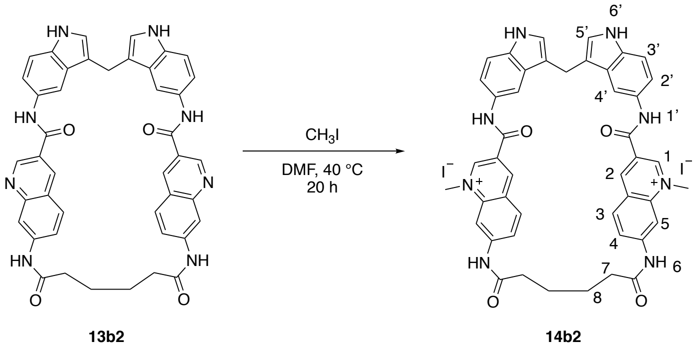

Following general procedure 4: **13b2** (10.0 mg, 13.8 µmol) and CH_3I (21 µL, 0.34 mmol) in DMF (3 mL) were reacted according to general procedure 4. Compound **14b2** was obtained (12 mg, 86%) after workup as an orange solid.

^{1}H NMR (400 MHz, DMSO-d_6) δ (ppm) 10.89 (s, 4H, H-6', H-1'), 10.68 (s, 2H, H-6), 9.79 (s, 2H, H-1), 9.00 (s, 2H, H-2), 8.65 (s, 2H, H-5), 8.58 (s, 2H, H-4'), 7.71 (d, *J* = 8.9 Hz, 2H, H-2'), 7.46 (d, *J* = 9.7 Hz, 4H, H-4, H-3'), 7.37 (d, *J* = 9.0 Hz, 4H, H-3, H-5'), 4.42 (s, 6H, CH_3), 4.12 (s, 2H, CH_2), 2.63 (s, 4H, H-7), 1.95 (s, 4H, H-8). ^{13}C NMR (151 MHz,) δ (ppm) 173.52, 165.03, 149.67, 146.42, 144.12, 140.45, 134.25, 132.58, 129.89, 128.29, 127.92, 127.09, 125.20, 123.55, 119.05, 116.43, 113.55, 112.74, 99.99, 45.30, 36.96, 24.72. HRMS: *m/z*: [M-H]$^+$ calcd for $C_{45}H_{39}N_8O_4^+$ 755.3089; Found 755.3118.

6^1,16^1-dimethyl-5,8,14,17-tetraoxo-1^1*H*,3^1*H*-4,7,15,18-tetraaza-6(3,6),16(6,3)-diquinolin-1-iuma-1,3(3,5)-diindolacyclooctadecaphane-6^1,16^1-diium (14a3)

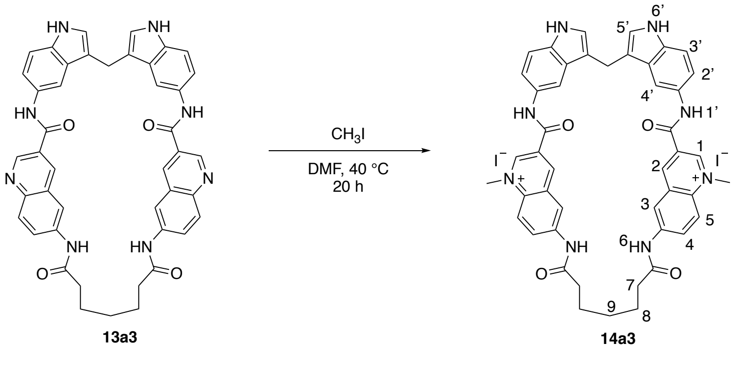

Following general procedure 4: **13a3** (18.0 mg, 24.3 µmol) and CH_3I (30 µL, 0.48 mmol) in DMF (4.5 mL) were reacted according to general procedure 4. Compound **14a3** was obtained (23 mg, 92%) after workup as an orange solid.

^{1}H NMR (600 MHz, DMSO-d_6) δ (ppm) 10.93 (s, 2H, H-6'), 10.70 (s, 2H, H-1'), 10.68 (s, 2H, H-6), 9.72 (d, J = 1.6 Hz, 2H, H-1), 9.61 (d, J = 1.6 Hz, 2H, H-2), 8.93 (d, J = 2.3 Hz, 2H, H-3), 8.46 (d, J = 9.5 Hz, 2H, H-5), 8.11 (dd, J = 9.5, 2.3 Hz, 2H, H-4), 7.88 (s, 2H, H-4'), 7.51 (d, J = 8.4 Hz, 2H, H-2'), 7.40 (d, J = 8.4 Hz, 2H, H-3'), 7.24 (d, J = 2.3 Hz, 2H, H-5'), 4.60 (s, 6H, CH_3), 4.11 (s, 2H, CH_2), 2.49 (s, 4H, H-7, as confirmed by HSQC and HMBC), 1.71 (app. d, J = 9.4 Hz, 4H, H-8), 1.49 (app. q, J = 7.2, 6.7 Hz, 2H, H-9). ^{13}C NMR (151 MHz, DMSO-d_6) δ (ppm) 172.57, 162.29, 160.39, 147.65, 144.05, 140.41, 134.69, 133.83, 129.41, 129.10, 128.91, 126.79, 124.32, 120.13, 115.93, 115.85, 114.71, 111.41, 110.97, 45.23, 35.78, 27.97, 24.32, 19.70; HRMS: m/z: [M-H]$^+$ calcd for $C_{46}H_{41}N_8O_4^+$ 769.3245; Found 769.3265.

6^1,16^1-dimethyl-5,8,14,17-tetraoxo-1^1*H*,3^1*H*-4,7,15,18-tetraaza-6(3,7),16(7,3)-diquinolin-1-iuma-1,3(3,5)-diindolacyclooctadecaphane-6^1,16^1-diiumdiiodide 14b3

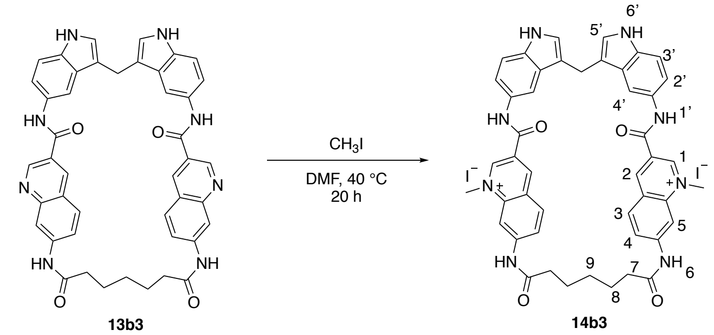

Following general procedure 4: **13b3** (10.0 mg, 13.5 µmol) and CH_3I (21 µL, 0.34 mmol) in DMF (3 mL) were reacted according to general procedure 4. Compound **14b3** was obtained (12 mg, 87%) after workup as an orange solid.

^{1}H NMR (400 MHz, DMSO-d_6) δ (ppm) 10.89 (d, J = 4.8 Hz, 4H, H-6', H-1'), 10.71 (s, 2H, H-6), 9.83 (d, J = 1.8 Hz, 2H, H-1), 9.08 (d, J = 1.7 Hz, 2H, H-2), 8.67 (d, J = 1.7 Hz, 2H, H-5), 8.56 (d, J = 1.9 Hz, 2H, H-4'), 7.68 (d, J = 9.0 Hz, 2H, H-2'), 7.49 (dd, J = 8.7, 1.9 Hz, 2H, H-4), 7.43 (d, J = 9.0 Hz, 2H, H-3'), 7.38 (d, J = 8.6 Hz, 2H, H-3), 7.35 (d, J = 2.4 Hz, 2H, H-5'), 4.41 (s, 6H, CH_3), 4.12 (s, 2H, CH_2), 2.56 (s, 4H, H-7), 1.77 (s, 4H, H-8), 1.43 (s, 4H, H-9). ^{13}C NMR (151 MHz, DMSO) δ (ppm) 173.69, 161.16, 149.90, 146.39, 144.12, 140.05, 134.10, 131.69, 129.61, 127.08, 126.85, 124.62, 124.03, 123.03, 117.17, 115.72, 112.52, 111.71, 103.31, 45.33, 40.51, 40.39, 40.25, 40.11, 39.97, 39.83, 39.69, 39.56, 35.92, 34.86, 27.14, 24.17. HRMS: m/z: [M-H]$^+$ calcd for $C_{46}H_{41}N_8O_4^+$ 769.3245; Found 769.3250.

6^1,17^1-dimethyl-5,8,15,18-tetraoxo-1^1*H*,3^1*H*-4,7,16,19-tetraaza-6(3,6),17(6,3)-diquinolin-1-iuma-1,3(3,5)-diindolacyclononadecaphane-6^1,17^1-diium (14a4)

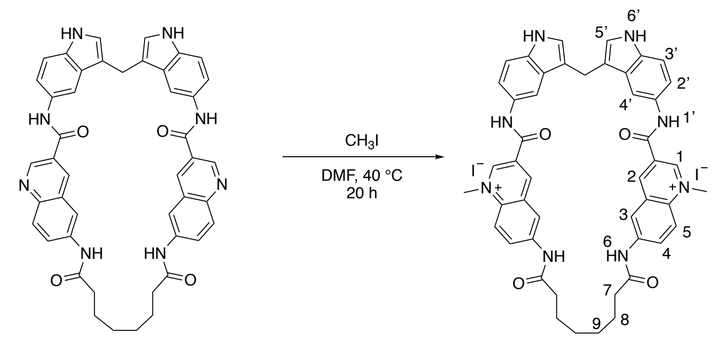

Following general procedure 4: **13a4** (10.0 mg, 13.2 µmol) and CH_3I (17.0 µL, 0.273 mmol) in DMF (3.5 mL) were reacted according to general procedure 4. Compound **14a4** was obtained (13 mg, 95%) after workup as an orange solid.

^{1}H NMR (600 MHz, DMSO-*d*$_6$) *δ* (ppm) 10.91 (s, 2H, H-6'), 10.74 (s, 2H, H-1'), 10.67 (s, 2H, H-6), 9.74 (s, 2H, H-1), 9.67 (s, 2H, H-2), 8.91 (s, 2H, H-3), 8.48 (d, *J* = 9.1 Hz, 2H, H-5), 8.19 (d, *J* = 9.1 Hz, 2H, H-4), 7.86 (s, 2H, H-4'), 7.63 (d, *J* = 8.5 Hz, 2H, H-2'), 7.41 (d, *J* = 8.5 Hz, 2H, H-3'), 7.17 (s, 2H, H-5'), 4.62 (s, 6H, CH_3), 4.13 (s, 2H, CH_2), 2.46 (app. s, 4H, H-7), 1.69 (app. s, 4H, H-8), 1.39 (app. s, 4H, H-9). ^{13}C NMR (151 MHz, DMSO-*d*$_6$) *δ* (ppm) 172.65, 160.31, 147.84, 144.02, 140.41, 134.77, 133.93, 129.57, 129.13, 129.05, 128.82, 126.83, 124.32, 120.20, 116.33, 115.59, 114.18, 111.42, 110.71, 45.26, 36.07, 28.08, 24.31, 20.04; HRMS: *m/z*: $[M-H]^+$ calcd for $C_{47}H_{43}N_8O_4^+$ 783.3402; Found 783.3421.

6^1,17^1-dimethyl-5,8,15,18-tetraoxo-1^1*H*,3^1*H*-4,7,16,19-tetraaza-6(3,7),17(7,3)-diquinolin-1-iuma-1,3(3,5)-diindolacyclononadecaphane-6^1,17^1-diium (14b4)

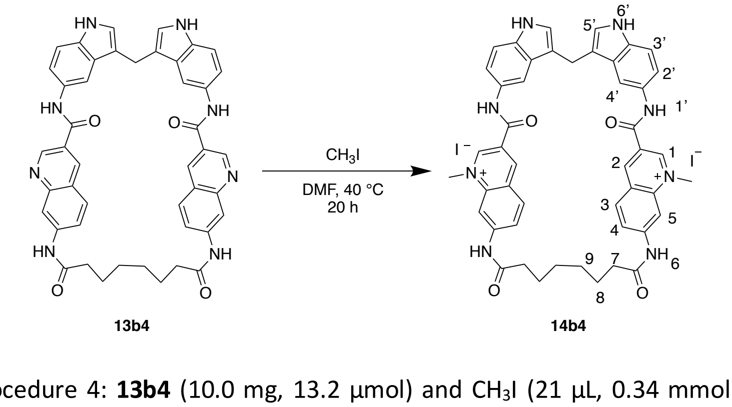

Following general procedure 4: **13b4** (10.0 mg, 13.2 µmol) and CH_3I (21 µL, 0.34 mmol) in DMF (3 mL) were reacted according to general procedure 4. Compound **14b4** was obtained (16 mg, 87%) after workup as an orange solid.

^{1}H NMR (600 MHz, DMSO-d_6) δ (ppm) 10.93 – 10.86 (m, 4H, H-6', H-1'), 10.70 (s, 2H, H-6), 9.82 (d, J = 1.8 Hz, 2H, H-1), 9.07 (s, 2H, H-2), 8.69 – 8.64 (m, 2H, H-5), 8.56 (s, 2H, H-4'), 7.67 (d, J = 8.9 Hz, 2H, H-2'), 7.48 (dd, J = 8.7, 1.9 Hz, 2H, H-4), 7.44 – 7.41 (m, 2H, H-3'), 7.38 (d, J = 8.5 Hz, 2H, H-3), 7.35 (d, J = 2.4 Hz, 2H, H-5'), 4.41 (s, 6H, CH_3), 4.12 (s, 2H, CH_2), 2.59 – 2.55 (m, 4H, H-7), 1.78 (s, 4H, H-8), 1.43 (s, 4H, H-9). ^{13}C NMR (151 MHz, DMSO) δ (ppm) 173.69, 161.16, 149.90, 146.41, 144.11, 140.05, 134.11, 131.70, 129.61, 127.09, 126.86, 124.62, 124.03, 123.03, 117.17, 115.73, 112.54, 111.71, 103.31, 45.33, 40.41, 40.27, 40.14, 40.00, 39.86, 39.72, 39.58, 35.93, 34.84, 27.15, 24.17. HRMS: m/z: $[M-H]^+$ calcd for $C_{47}H_{43}N_8O_4^+$ 783.3402; Found 783.3400.

6^1,18^1-dimethyl-5,8,16,19-tetraoxo-1^1*H*,3^1*H*-4,7,17,20-tetraaza-6(3,7),18(7,3)-diquinolin-1-iuma-1,3(3,5)-diindolacycloicosaphane-6^1,18^1-diium 14b5

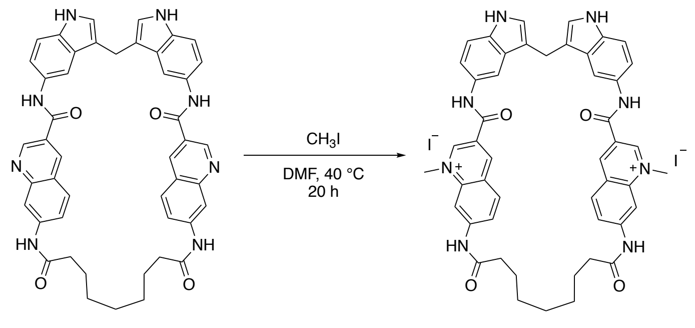

13b5 **14b5**

Following general procedure 4: **13b5** (12.0 mg, 13.0 µmol) and CH_3I (21 µL, 0.34 mmol) in DMF (3 mL) were reacted according to general procedure 4. Compound **14b5** was obtained (13 mg, 95%) after workup as an orange solid.

^{1}H NMR (600 MHz, DMSO-d_6) δ (ppm) 10.83 (s, 1H), 10.75 (s, 2H), 10.45 (s, 1H), 9.73 (s, 2H), 9.54 (s, 1H), 9.30 (s, 1H), 9.21 (s, 1H), 8.85 (s, 1H), 8.64 (s, 1H), 8.41 (d, J = 6.0 Hz, 1H), 8.35 (s, 1H), 8.06 (dd, J = 6.0, 1.2 Hz, 2H), 7.96 (d, J = 5.8 Hz, 2H), 7.75 (d, J = 5.9 Hz, 1H), 7.47 – 7.41 (m, 2H), 7.38 (d, J = 5.7 Hz, 2H), 7.34 (d, J = 1.6 Hz, 2H), 4.52 (s, 3H), 4.37 (s, 3H), 4.12 (s, 2H), 2.61 – 2.57 (br, 4H), 1.73 (br, 4H), 1.44 (br, 6H). ^{13}C NMR (151 MHz, DMSO-d_6) δ (ppm) 172.50, 162.31, 150.09, 149.23, 148.78, 141.76, 135.26, 130.50, 129.50, 127.98, 126.60, 125.39, 124.16, 122.95, 122.61, 121.17, 117.04, 112.09, 111.30, 36.47, 31.76, 29.46, 27.65, 22.56. HRMS: m/z: $[M-H]^+$ calcd for $C_{48}H_{45}N_8O_4^+$ 797.3531; Found 797.3574.

6[1],14[1]-dimethyl-5,8,12,15-tetraoxo-1[1]*H*,3[1]*H*-4,7,10,13,16-pentaaza-6(3,6),14(6,3)-diquinolin-1-iuma-1,3(3,5)-diindolacyclohexadecaphane-6[1],14[1],10-triium trifluoroacetate (15a)

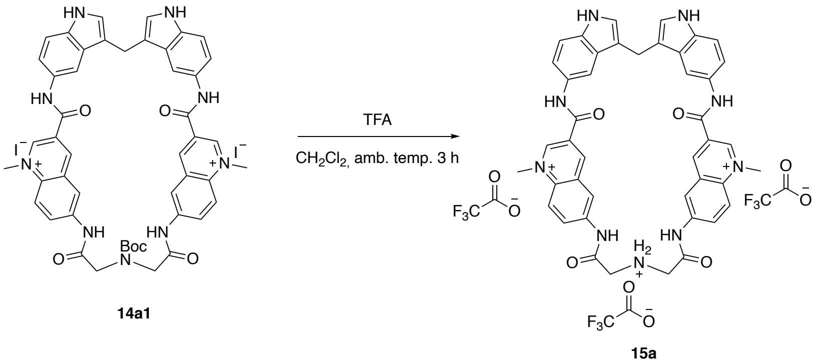

To a RBF (50 mL) containing **14a1** was added CH_2Cl_2 and TFA. The solution was then stirred at ambient temperature until **14a1** was consumed (~3 h). The solution was then concentrated under reduced pressure, diluted with acetone:Et_2O (1:1) and the formed precipitate was collected with suction filtration to afford the product as a red solid. HRMS: *m/z*: $[M-H]^+$ calcd for $C_{43}H_{36}N_9O_4^+$ 742,2885; Found 742.2911.

6[1],16[1]-dimethyl-5,8,14,17-tetraoxo-11*H*,31*H*-4,7,11,15,18-pentaaza-6(3,7),16(7,3)-diquinolin-1-iuma-1,3(3,5)-diindolacyclooctadecaphane-6[1],16[1],11-triium trifluoroacetate (15b)

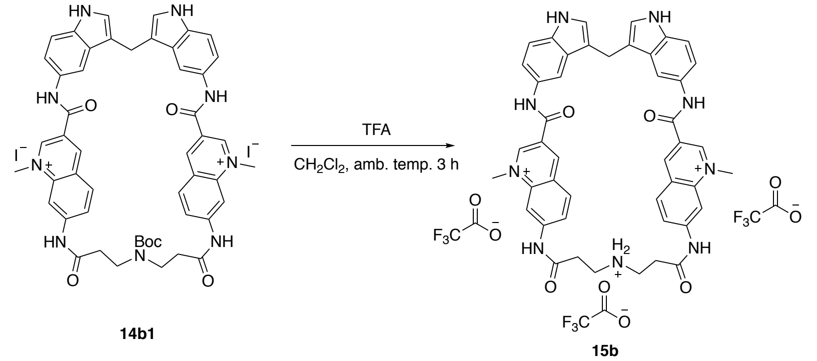

To a RBF (50 mL) containing **14b1** was added CH_2Cl_2 and TFA. The solution was then stirred at ambient temperature until **14b1** was consumed (~3 h). The solution was then concentrated under reduced pressure, diluted with acetone:Et_2O (1:1) and the formed precipitate was collected with suction filtration to afford the product as a red solid.

^1H NMR (600 MHz, DMSO-d_6) δ (ppm) 11.27 (s, 2H), 10.90 (s, 2H), 10.61 (s, 2H), 9.70 (s, 2H), 9.29 (s, 2H), 8.53 (d, J = 6.3 Hz, 4H), 7.95 (d, J = 8.9 Hz, 2H), 7.63 (d, J = 8.8 Hz, 1H), 7.37 (s, 4H), 4.53 (d, J = 41.0 Hz, 4H), 4.18 (s, 6H), 4.10 (s, 2H), 3.44 (s, 4H). HRMS: *m/z*: $[M/2]^+$ calcd for $C_{46}H_{40}N_9O_4^+$ 385.6635; Found 385.6652.

N,N'-(pentane-1,5-diyl)bis(quinoline-3-carboxamide)

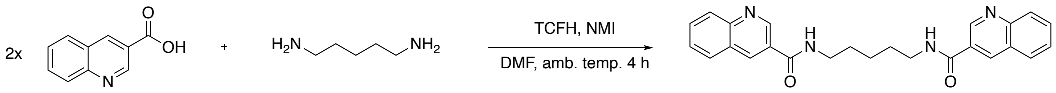

To a vial (5 mL) was added quinoline-3-carboxylic acid (100 mg, 0.57 mmol), TCFH (243 mg, 0.87 mmol), NMI (184 µL, 2.31 mmol) and 1,5-pentanediamine (33 µL, 0.28 mmol), then, anhydrous DMF (3 mL) was added and the solution was stirred at ambient temperature for 4 h. Upon completion was water added and a white precipitate was formed, then washed with water and Et_2O to afford the desired compound (94 mg, 79%) as a white solid.

^1H NMR (400 MHz, DMSO-d_6) δ (ppm) 9.28 (d, J = 2.2 Hz, 2H), 8.83 (d, J = 5.6 Hz, 2H), 8.80 (d, J = 2.2 Hz, 2H), 8.11-8.04 (m, 4H), 7.86 (ddd, J = 8.4, 6.8, 1.5 Hz, 2H), 7.68 (ddd, J = 8.1, 6.8, 1.2 Hz, 2H), 3.41-3.29 (m, 5H), 1.66 (p, J = 7.3 Hz, 4H), 1.53-1.40 (m, 2H).

3,3'-((pentane-1,5-diylbis(azanediyl))bis(carbonyl))bis(1-methylquinolin-1-ium) diiodide (16)

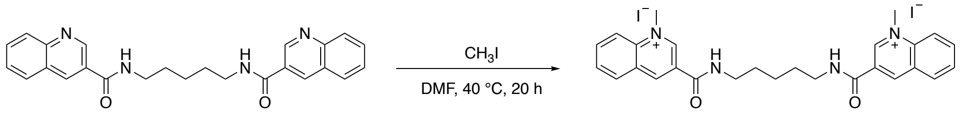

16

Following general procedure 4: **N,N'-(pentane-1,5-diyl)bis(quinoline-3-carboxamide)** (25.0 mg, 0.06 mmol) and CH_3I (75 µL, 1.28 mmol) in DMF (5 mL) were reacted according to general procedure 4. Compound **16** was obtained (26 mg, 97%) after workup as bright yellow powder.

^1H NMR (400 MHz, DMSO-d_6) δ (ppm) 9.88 (d, J = 1.8 Hz, 2H), 9.64 (t, J = 1.3 Hz, 2H), 9.17 (t, J = 5.5 Hz, 2H), 8.59-8.50 (m, 4H), 8.38 (ddd, J = 8.8, 7.0, 1.5 Hz, 2H), 8.16-8.10 (m, 2H), 4.70 (s, 6H), 3.43 (q, J = 6.7 Hz, 4H), 1.69 (p, J = 7.4 Hz, 4H), 1.51 (s, 2H). ^{13}C NMR (100 MHz, DMSO) δ (ppm) 162.11, 150.25, 145.21, 139.16, 137.22, 131.75, 131.09, 128.59, 128.23, 119.81, 46.07, 39.79, 29.09, 24.36. HRMS: m/z: $[M+H]^+$ calcd for $C_{27}H_{31}N_4O_2^+$ 443.2431; Found 443.2414.

2,9,12,18-tetraaza-1,10(6,3)-diquinolinacyclononadecaphane-3,8,11,19-tetraone

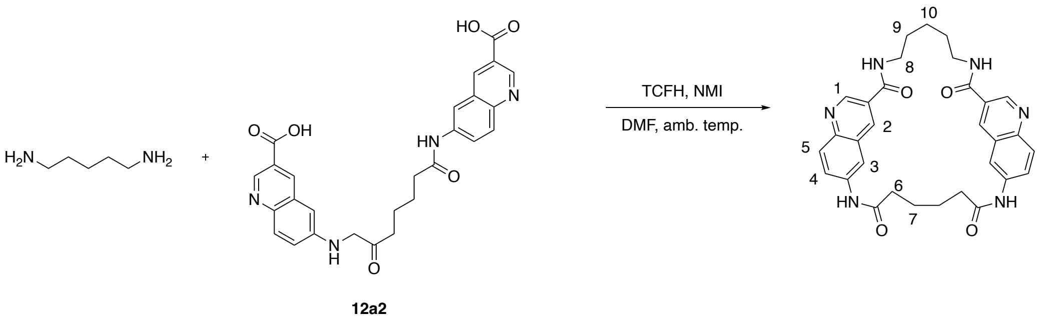

12a2

Following general procedure 3: **12a2** (40.0 mg, 0.082 mmol), TCFH (92.0 mg, 0.328 mmol), NMI (52 µL, 0.65 mmol) and 1,5-pentanediamine (11.6 µL, 0.650 mmol) were reacted according to general procedure 3. Upon completion was water added and a precipitate was formed, then washed with water and Et_2O. Purification by flash column chromatography (eluent: 5% → 12% CH_3OH in CH_2Cl_2) afforded the compound (7.0 mg, 15 %) as a white solid.

^{1}H NMR (400 MHz, CD_3OD) δ (ppm) 8.86 (d, J = 2.0 Hz, 2H, H-1), 8.31 (d, J = 2.0 Hz, 2H, H-2), 7.96 (d, J = 2.3 Hz, 2H, H-3), 7.86 (dd, J = 9.1, 2.3 Hz, 2H, H-4), 7.62 (d, J = 9.1 Hz, 2H, H-5), 3.59 – 3.48 (m, 4H, H-8), 2.54 (app. d, J = 5.1 Hz, 4H, H-6), 1.97 (s, 4H, H-7), 1.81 – 1.71 (m, 4H, H-9), 1.58 (app. dt, J = 13.6, 6.6 Hz, 2H, H-10).

1^1,10^1-dimethyl-3,8,11,19-tetraoxo-2,9,12,18-tetraaza-1,10(6,3)-diquinolin-1-iumacyclononadecaphane-1^1,10^1-diium (17)

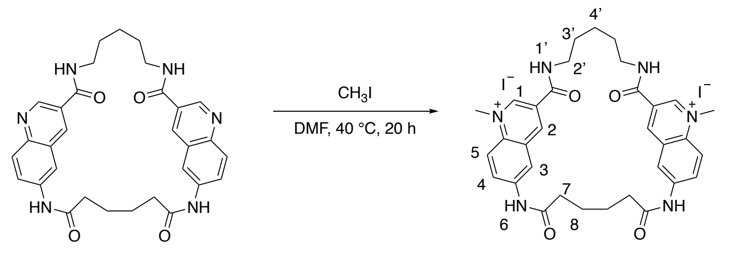

Following general procedure 4: **2,9,12,18-tetraaza-1,10(6,3)-diquinolinacyclononadecaphane-3,8,11,19-tetraone** (7.00 mg, 0.013 mmol) and CH_3I (16.0 µL, 0.257 mmol) in DMF (2 mL) were reacted according to general procedure 4. Compound **17** was obtained (10 mg, 94%) after workup as pale yellow solid.

^{1}H NMR (400 MHz, DMSO-d_6) δ (ppm) 10.68 (s, 2H, H-6), 9.60 (s, 2H, H-1), 9.45 (s, 2H, H-2), 9.00 (s, 2H, H-1'), 8.73 (s, 2H, H-3), 8.40 (d, J = 9.5 Hz, 2H, H-4), 8.19 (d, J = 9.5 Hz, 2H, H-5), 4.59 (s, 6H, CH_3), 3.43 (s, 4H, H-2'), 2.50 (s, 4H, H-7), 1.79 (s, 4H, H-8), 1.63 (s, 6H, H-3´& H-4'). ^{13}C NMR (100 MHz, DMSO-d_6) δ (ppm) 172.63, 161.57, 147.63, 142.78, 140.44, 134.55, 129.08, 128.76, 127.59, 119.98, 115.78, 45.29, 39.88, 36.03, 28.17, 24.24, 24.06. HRMS: m/z: $[M/2]^+$ calcd for $C_{33}H_{39}N_6O_4{}^+$ 291.1477; Found 291.1470.

NMR spectra of Synthesized Molecules

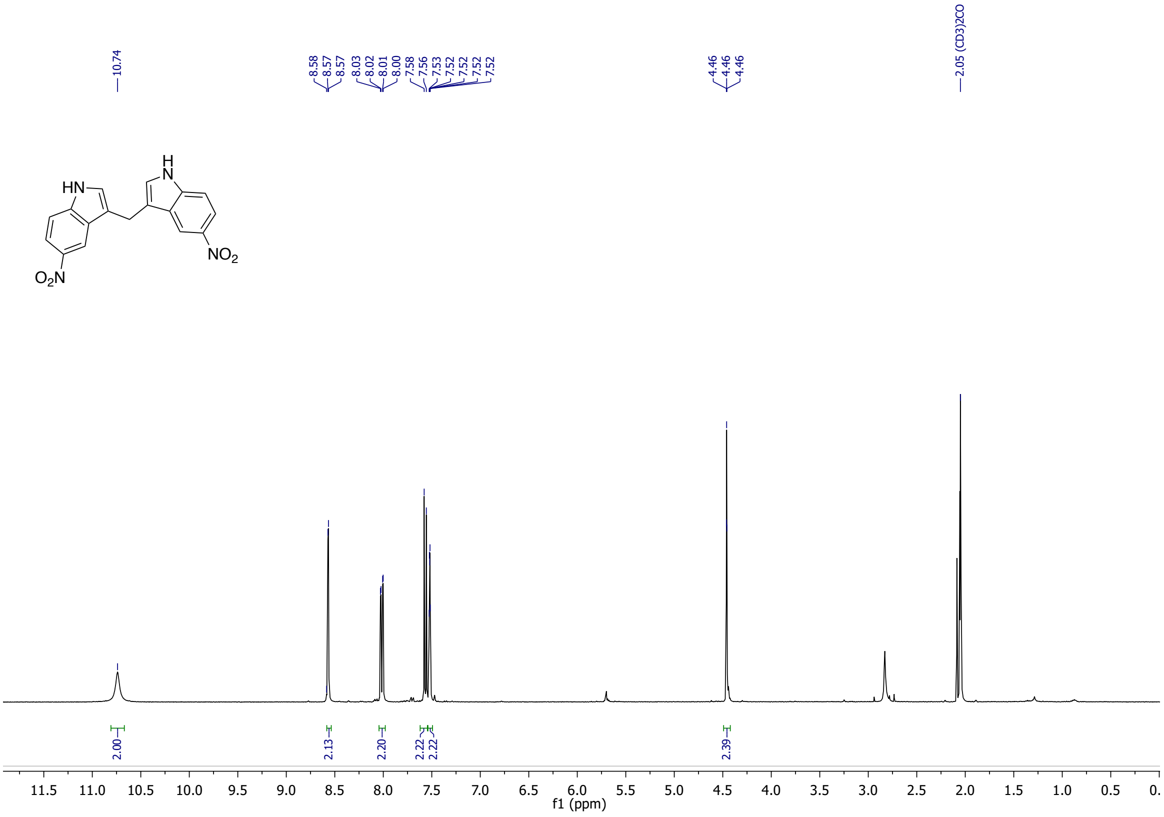

^{1}H NMR spectrum of **4** measured in acetone-d_6 at 400 MHz.

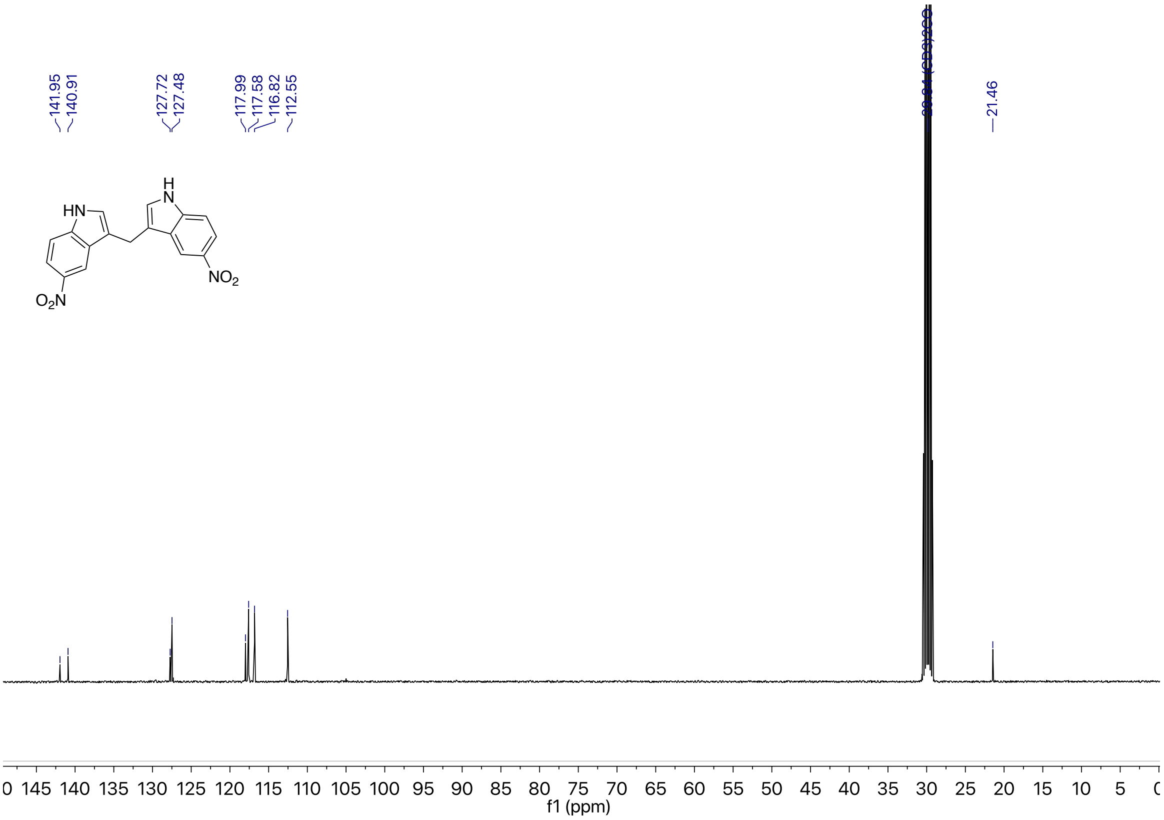

^{13}C NMR spectrum of **4** measured in acetone-d_6 at 100 MHz.

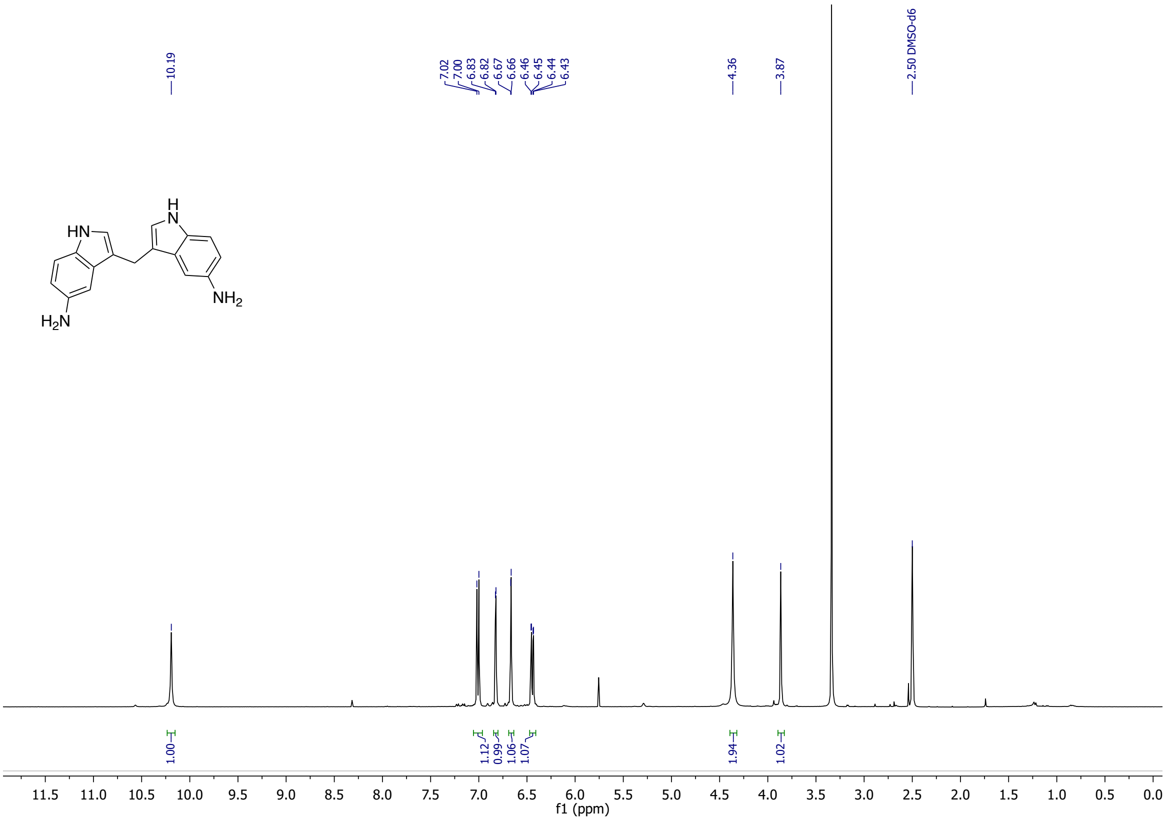

^{1}H NMR spectrum of **5** measured in DMSO-d_6 at 400 MHz.

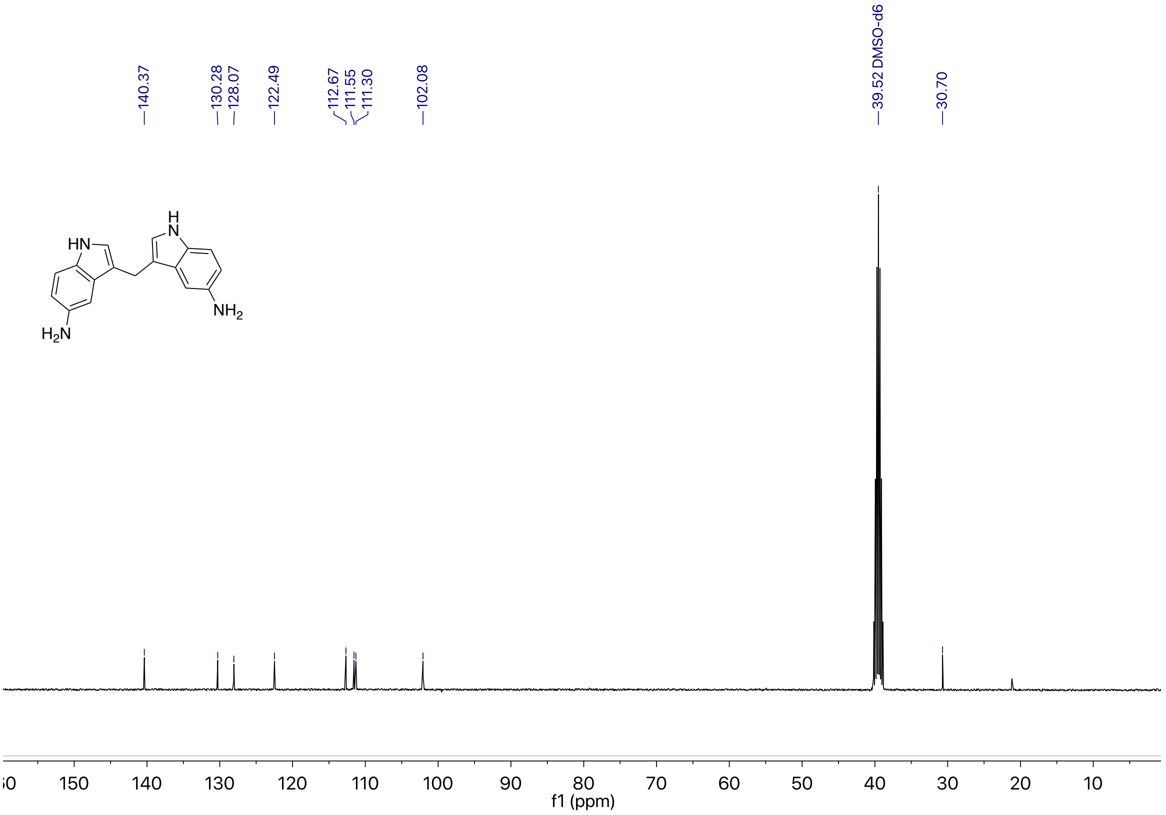

^{13}C NMR spectrum of **5** measured in DMSO-d_6 at 100 MHz.

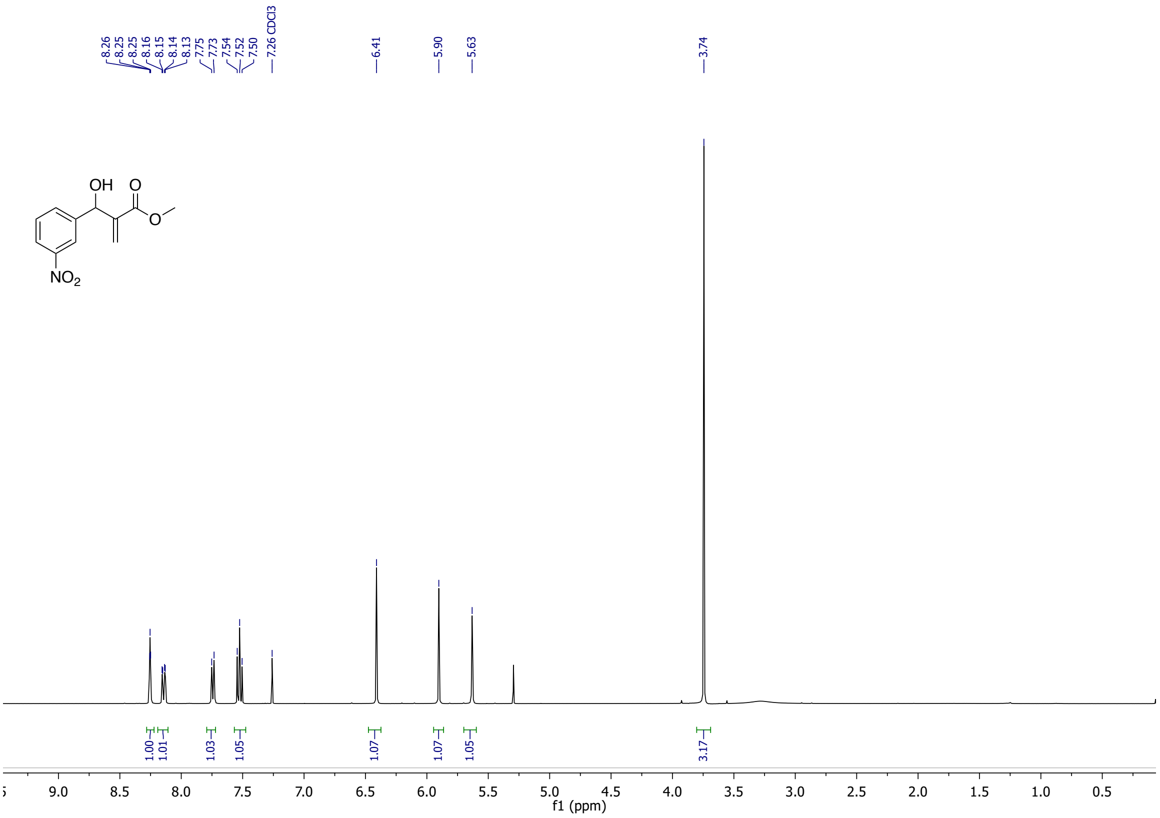

^{1}H NMR spectrum of **6a** measured in $CDCl_3$ at 400 MHz.

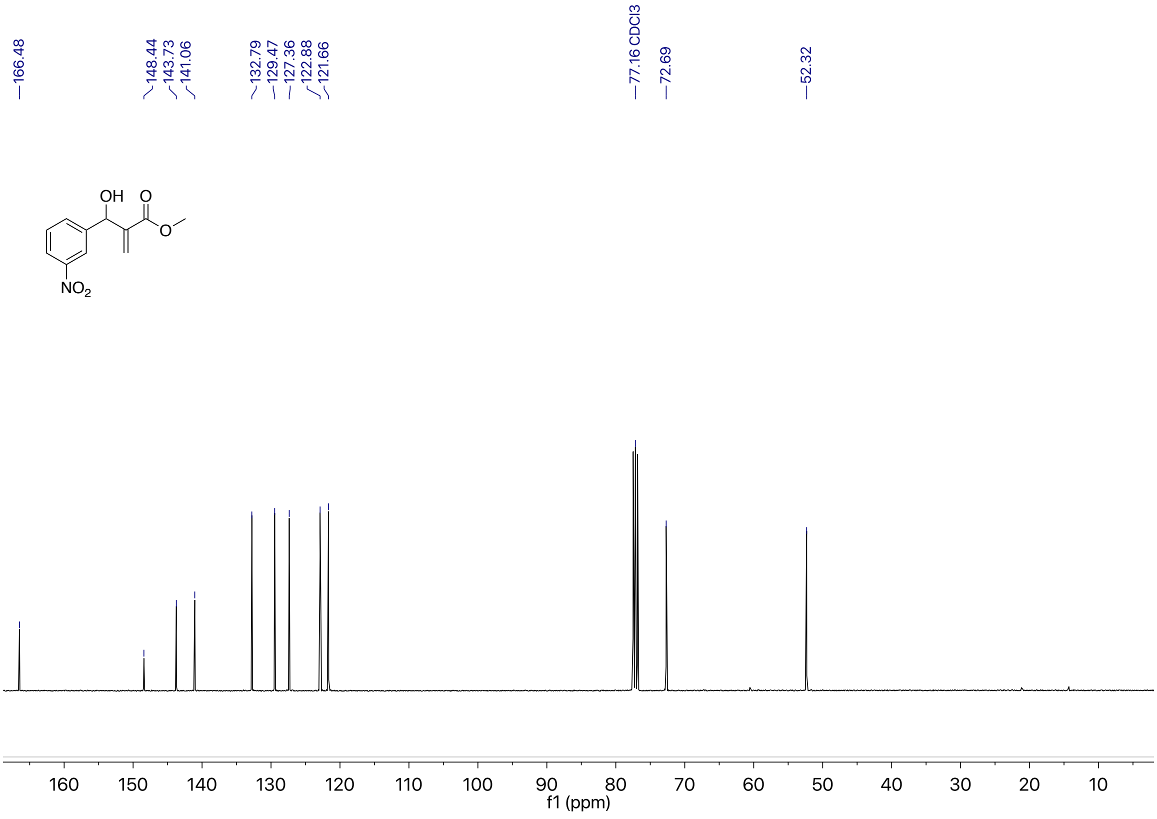

^{13}C NMR spectrum of **6a** measured in $CDCl_3$ at 100 MHz.

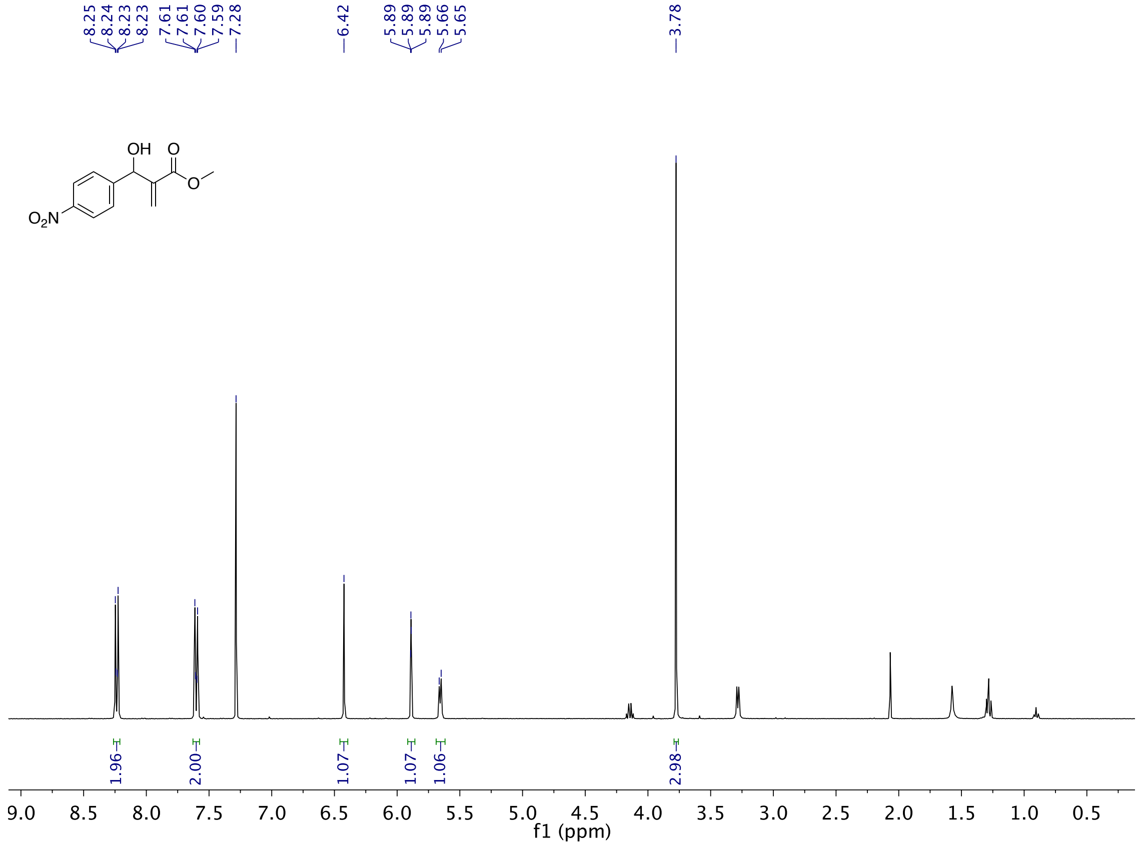

^{1}H NMR spectrum of **6b** measured in $CDCl_3$ at 400 MHz.

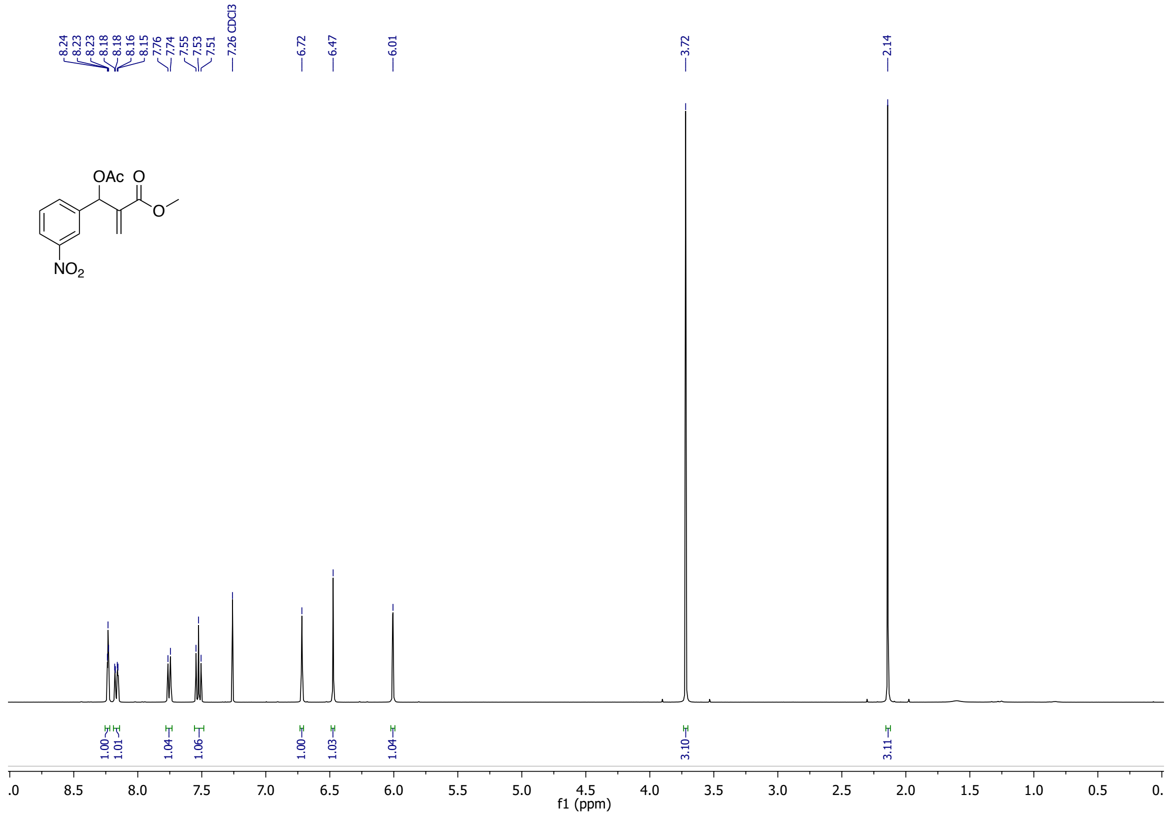

^{1}H NMR spectrum of **7a** measured in $CDCl_3$ at 400 MHz.

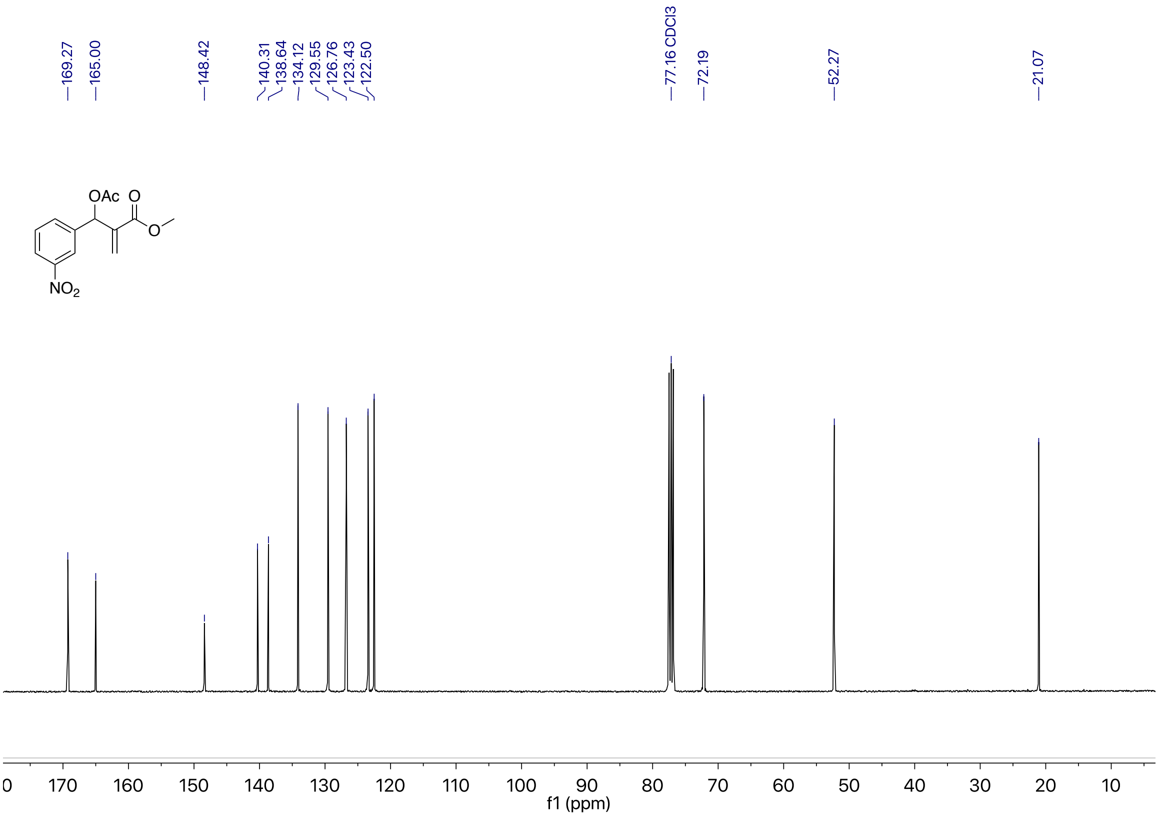

^{13}C NMR spectrum of **7a** measured in $CDCl_3$ at 100 MHz.

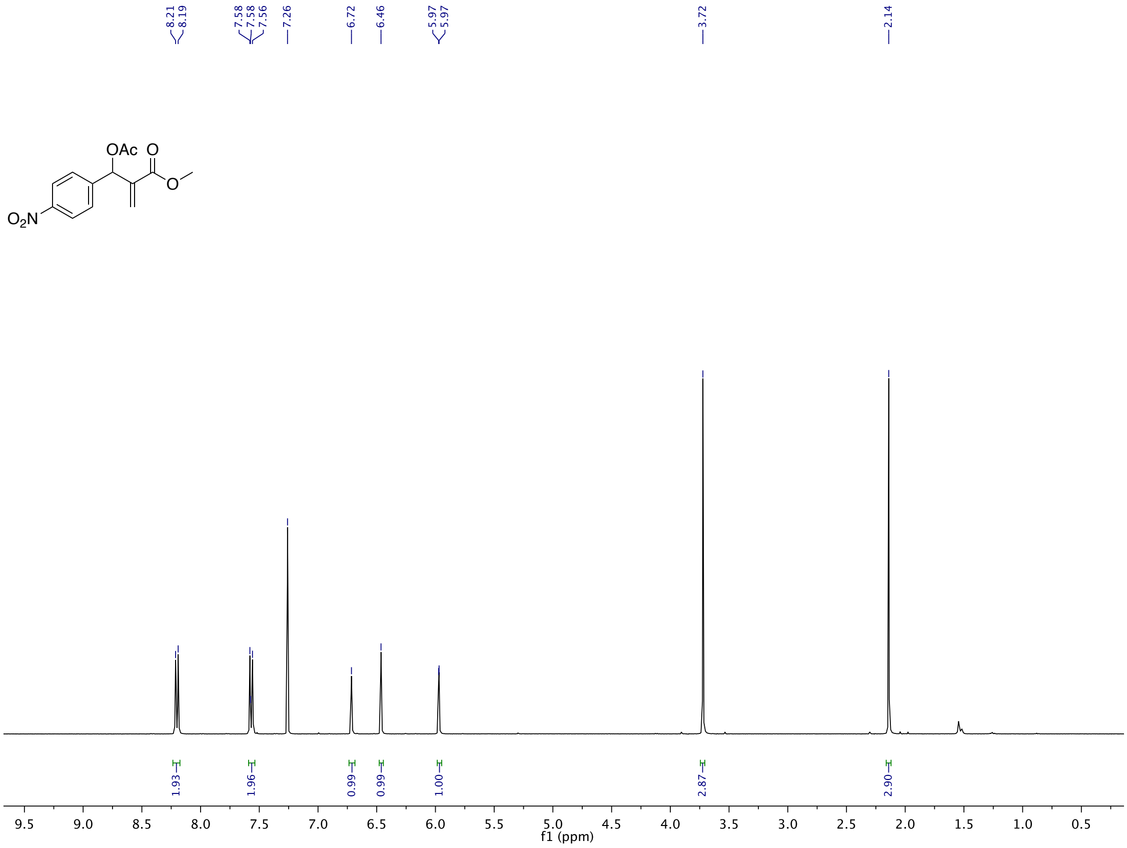

^{1}H NMR spectrum of **7b** measured in $CDCl_3$ at 400 MHz.

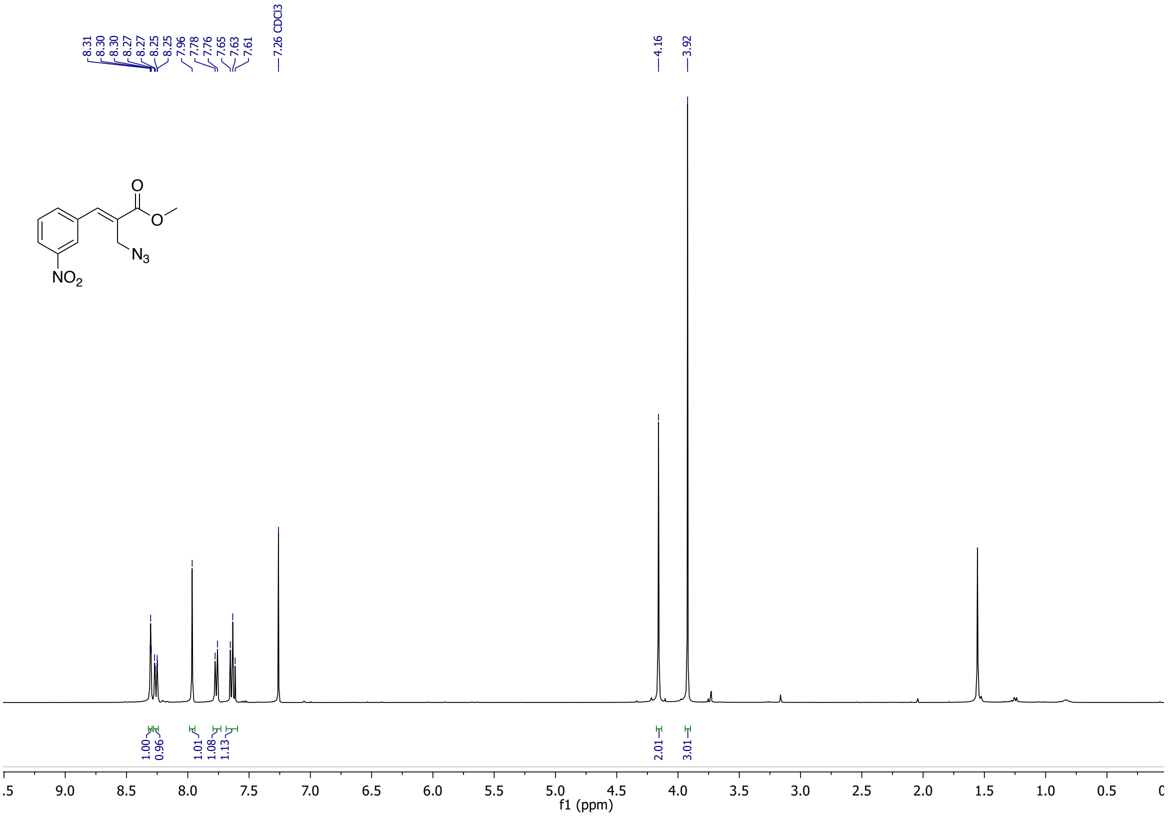

^{1}H NMR spectrum of **8a** measured in $CDCl_3$ at 400 MHz.

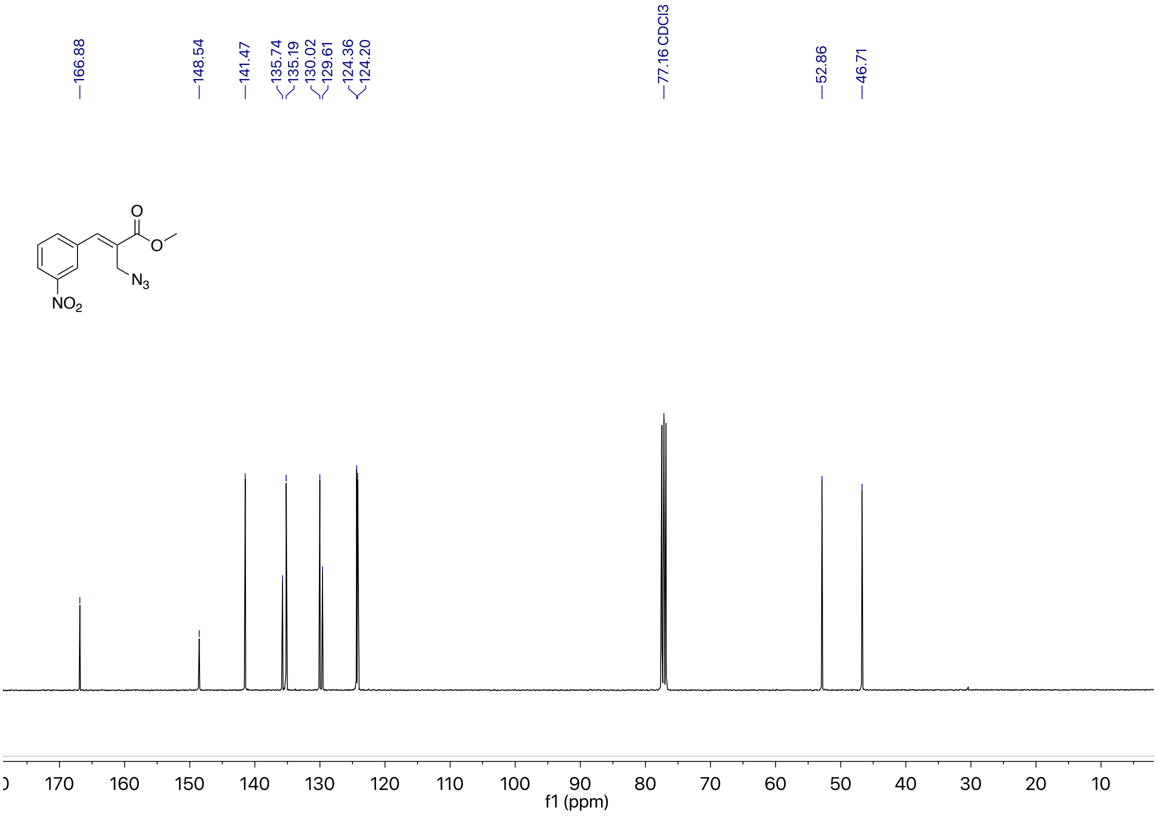

^{13}C NMR spectrum of **8a** measured in $CDCl_3$ at 100 MHz.

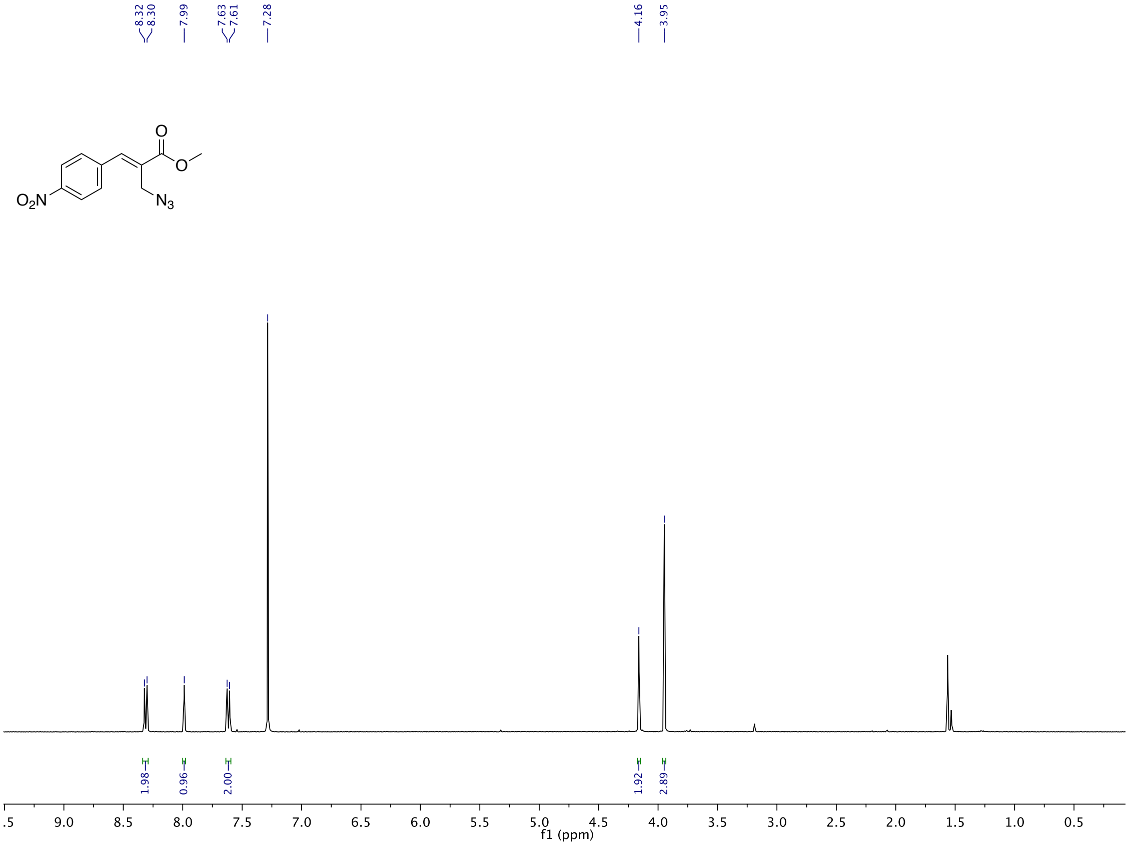

^{1}H NMR spectrum of **8b** measured in $CDCl_3$ at 400 MHz.

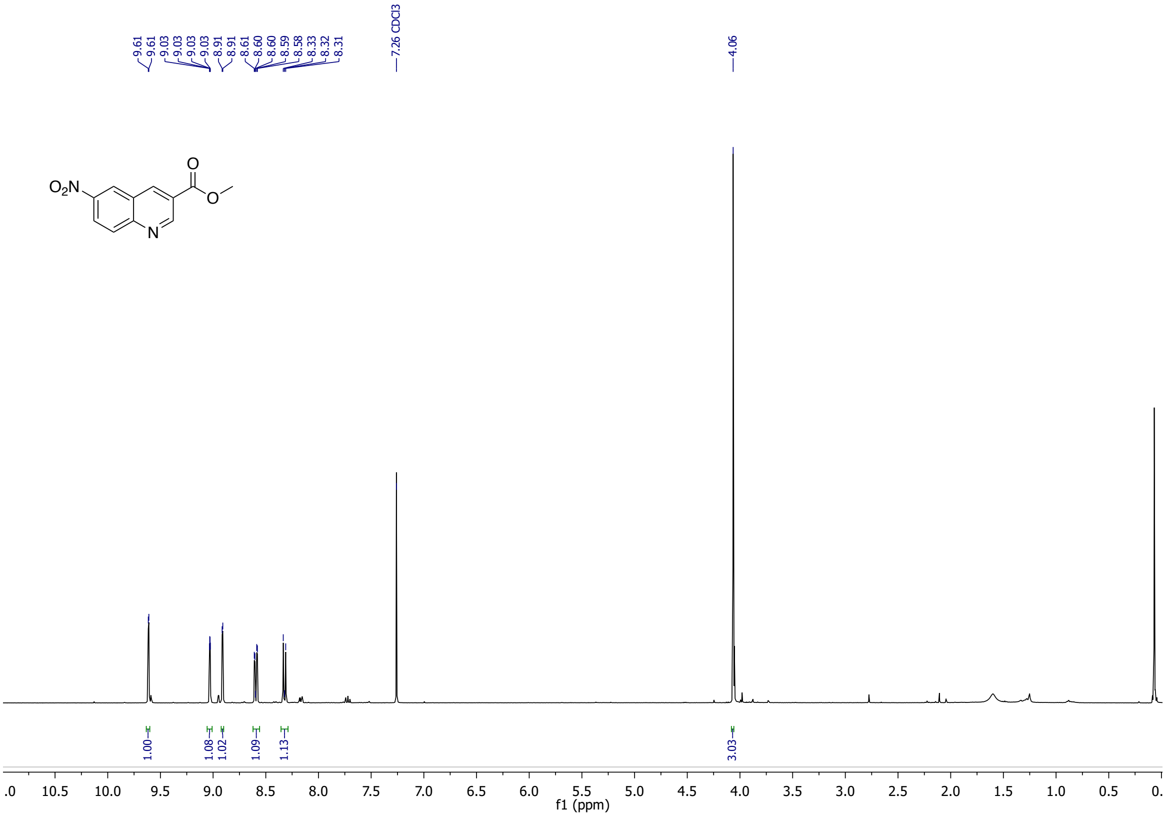

^{1}H NMR spectrum of **9a** measured in $CDCl_3$ at 400 MHz.

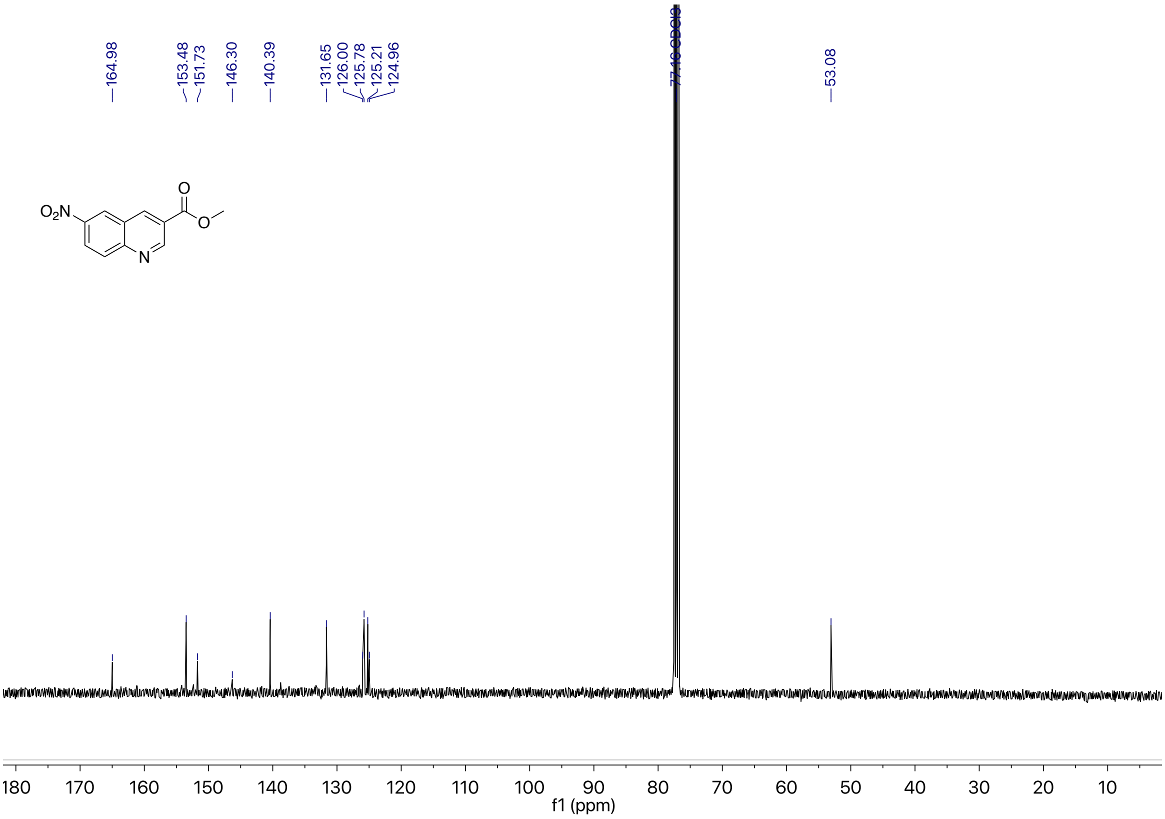

^{13}C NMR spectrum of **9a** measured in $CDCl_3$ at 100 MHz.

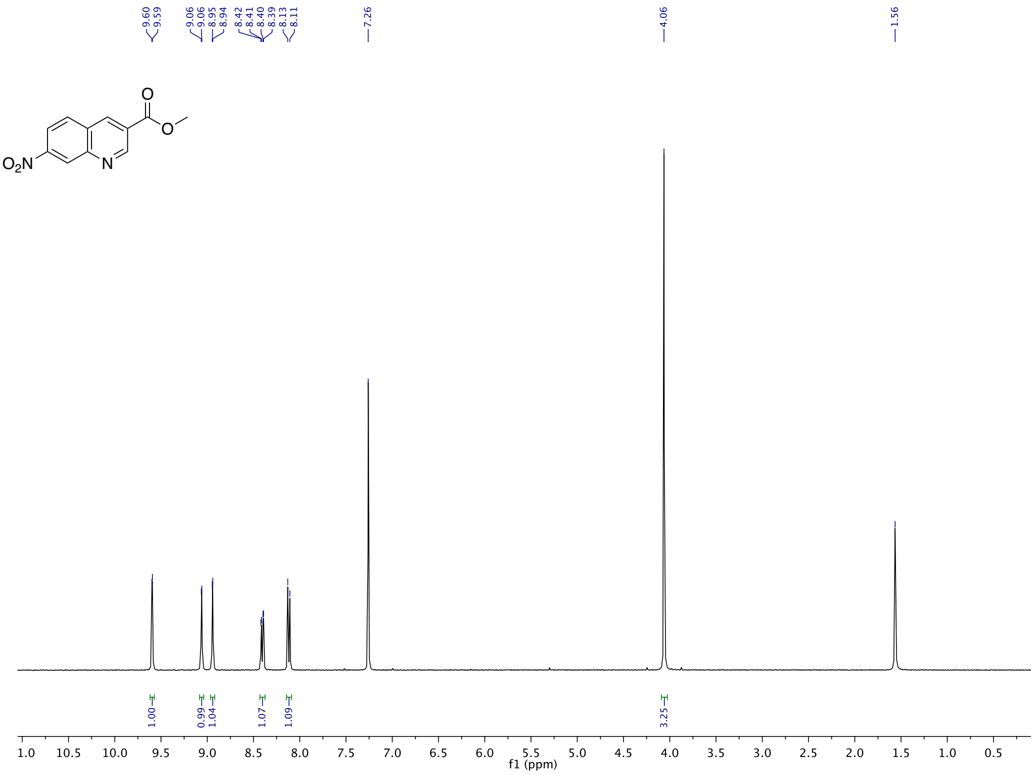

^{1}H NMR spectrum of **9b** measured in $CDCl_3$ at 400 MHz.

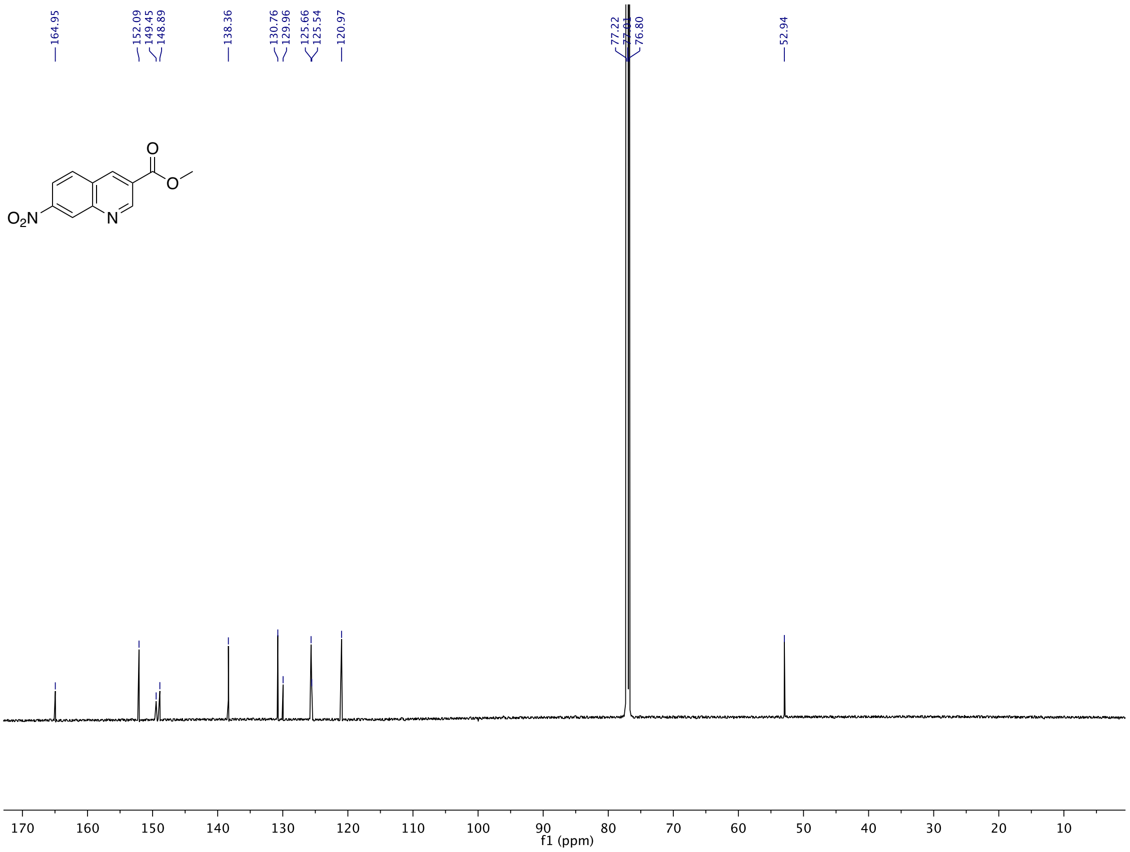

^{13}C NMR spectrum of **9b** measured in $CDCl_3$ at 150 MHz.

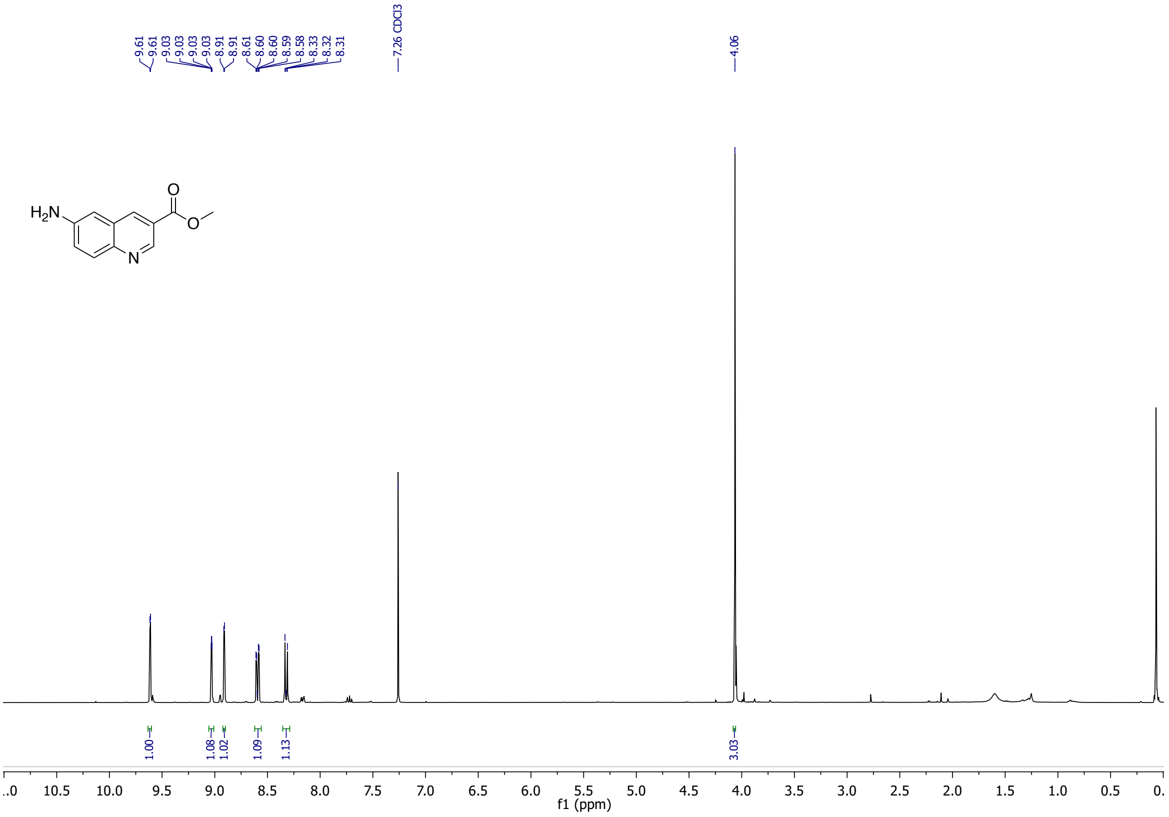

^{1}H NMR spectrum of **10a** measured in acetone-d_6 at 400 MHz.

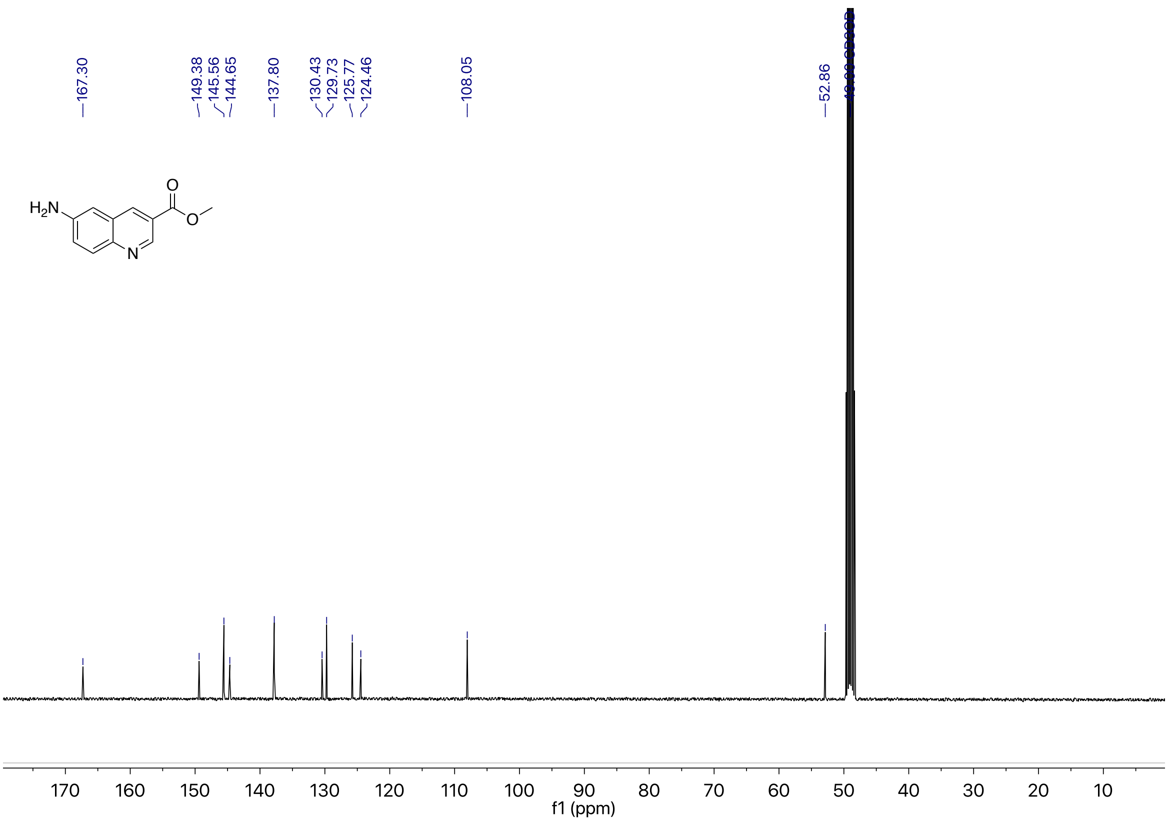

^{13}C NMR spectrum of **10a** measured in MeOD at 100 MHz.

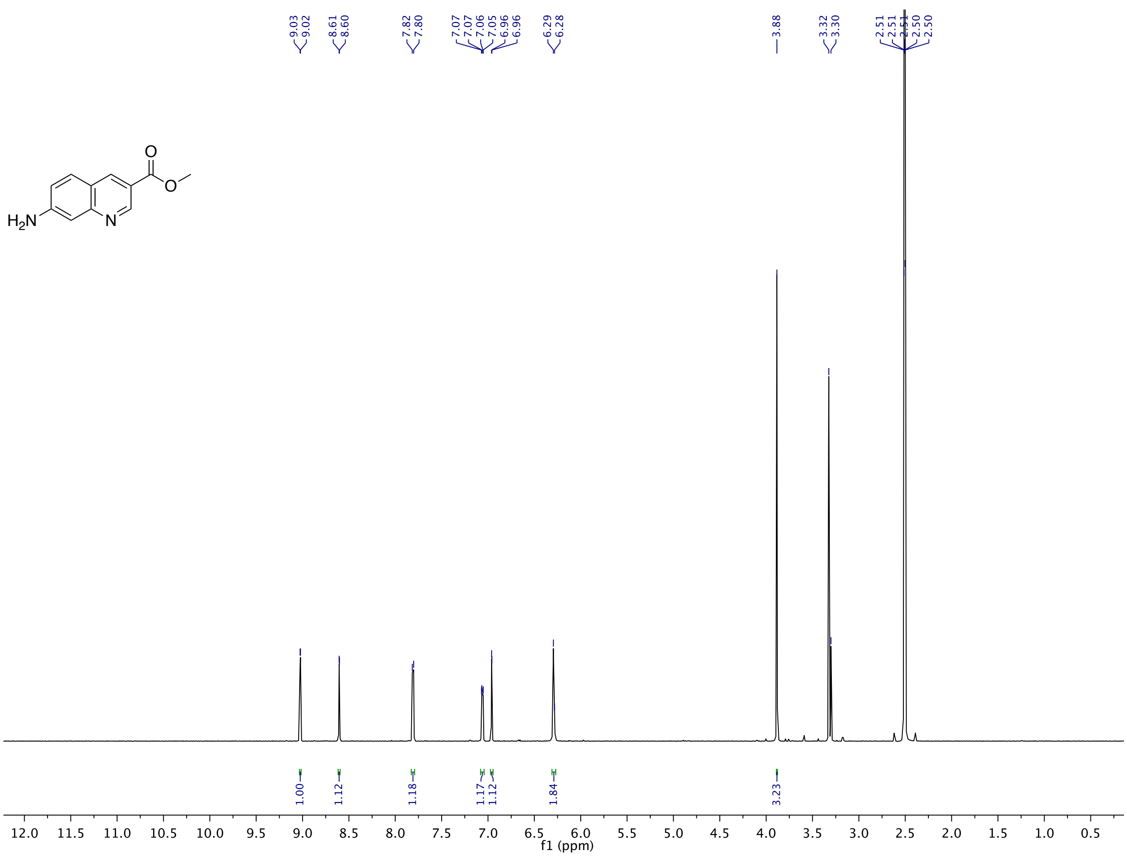

^{1}H NMR spectrum of **10b** measured in DMSO-d_6 at 600 MHz.

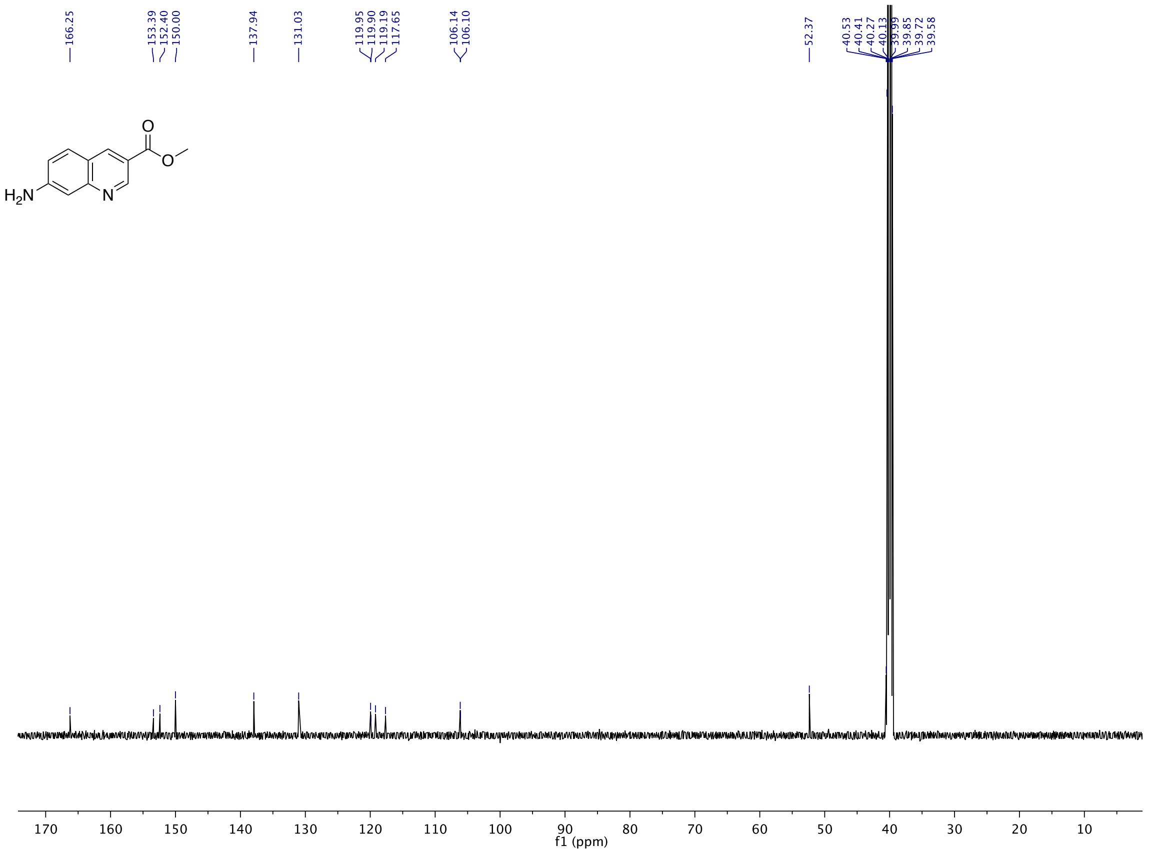

^{13}C NMR spectrum of **10b** measured in DMSO-d_6 at 150 MHz.

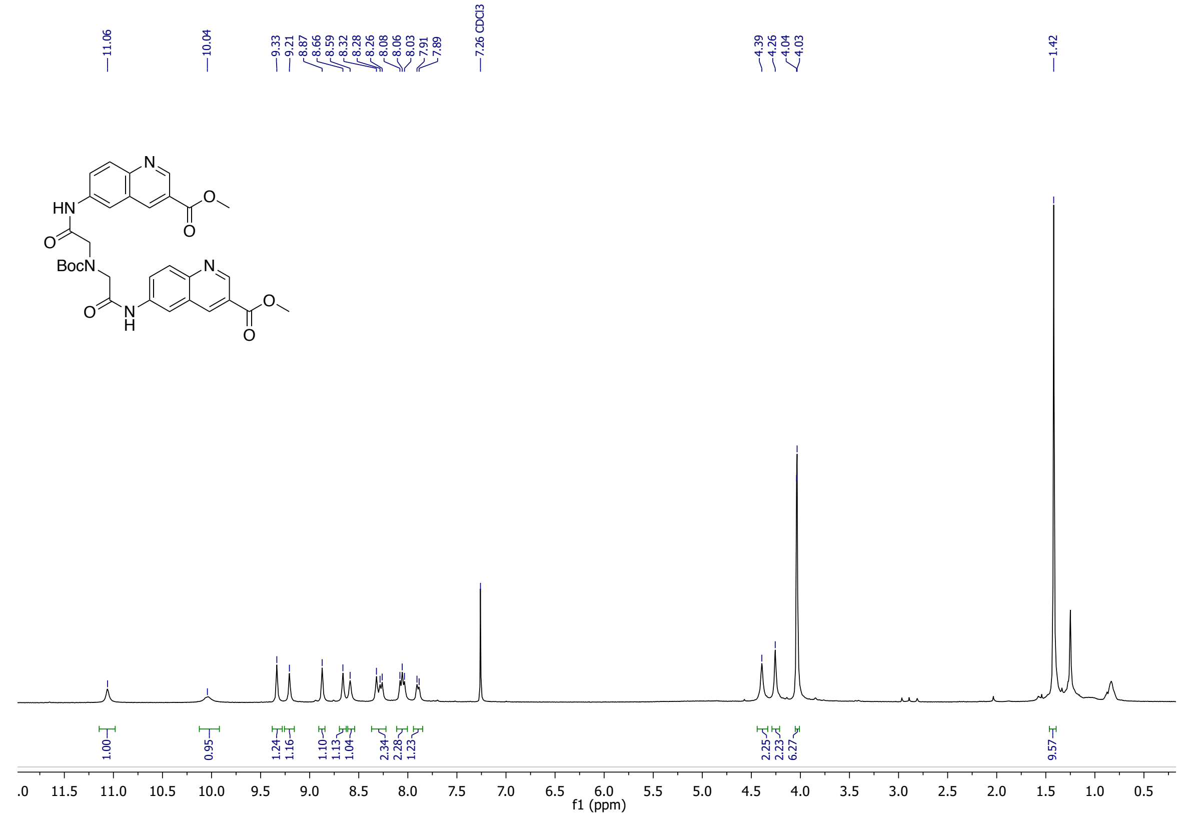

^{1}H NMR spectrum of **11a1** measured in $CDCl_3$ at 400 MHz.

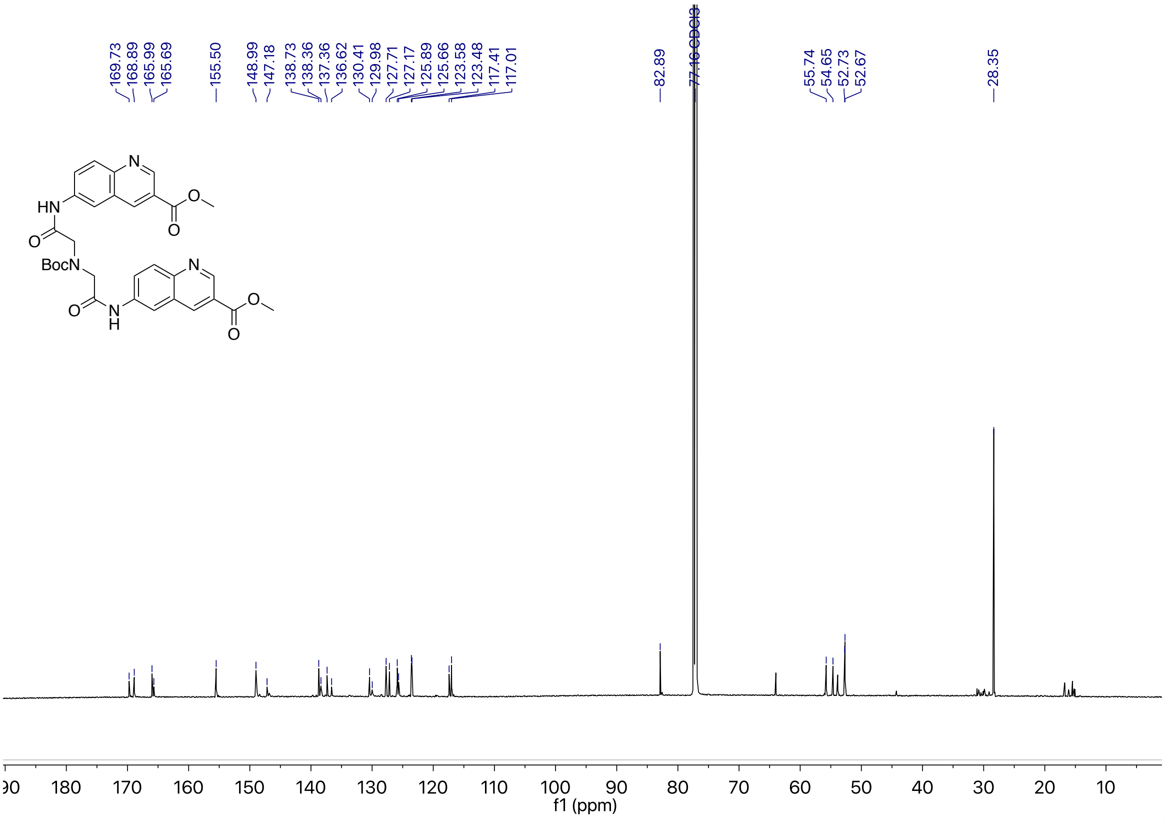

^{13}C NMR spectrum of **11a1** measured in $CDCl_3$ at 151 MHz.

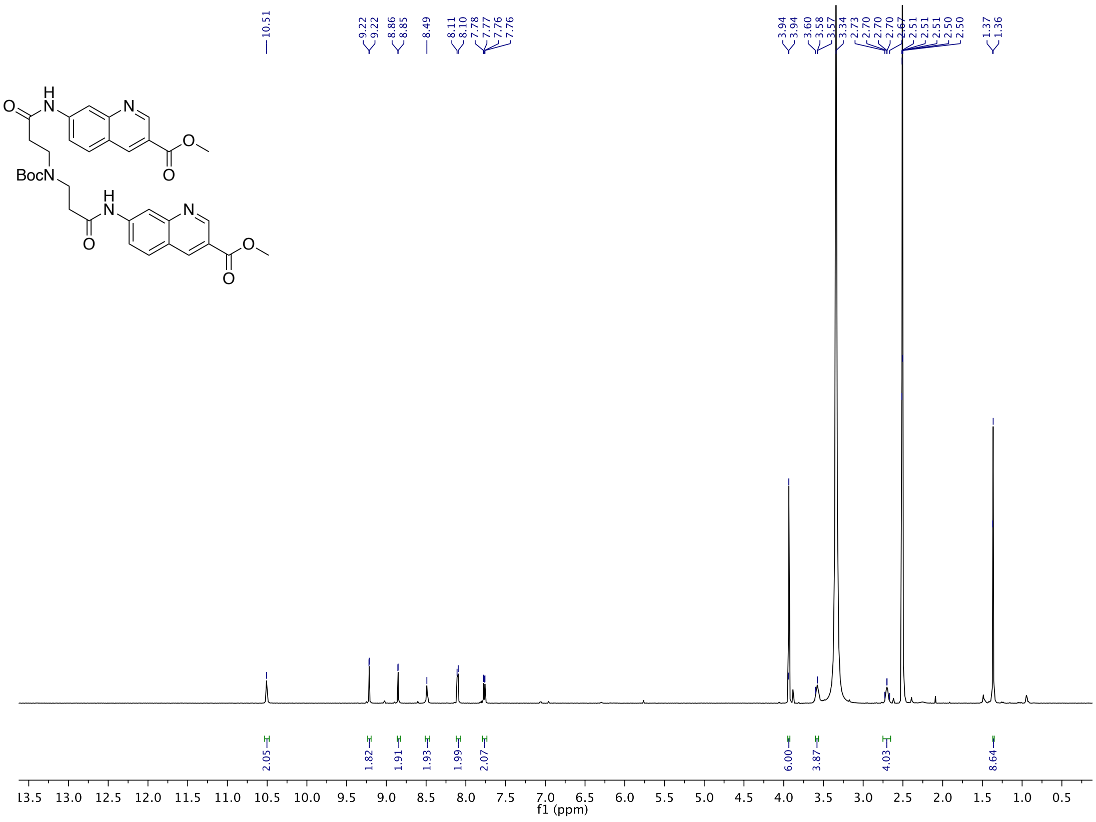

^{1}H NMR spectrum of **11b1** measured in DMSO-d_6 at 600 MHz.

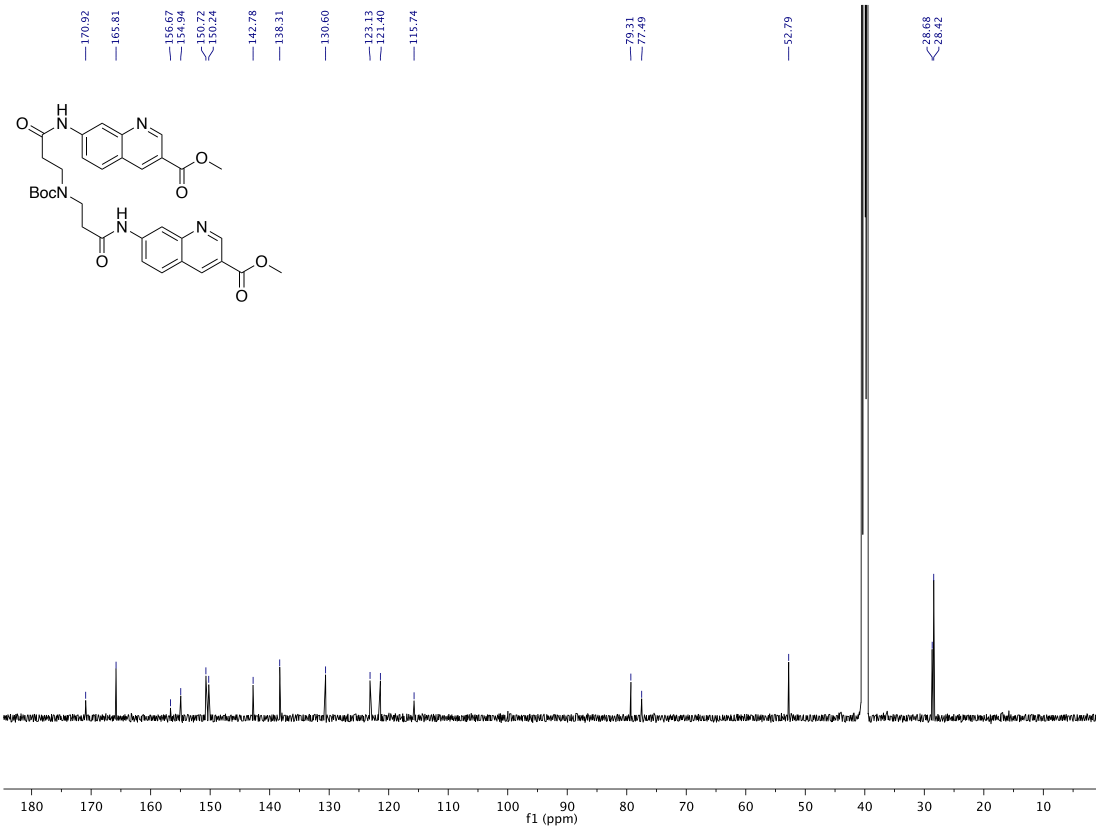

^{13}C NMR spectrum of **11b1** measured in DMSO-d_6 at 150 MHz.

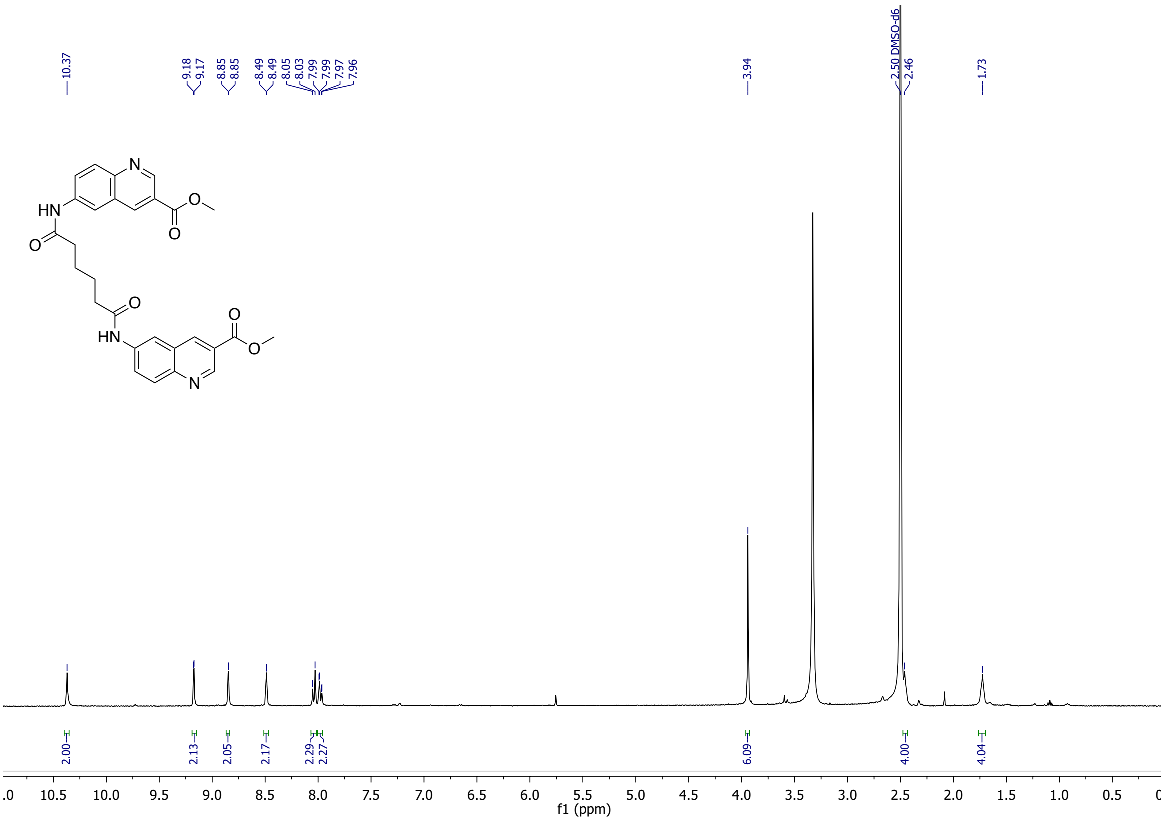

^{1}H NMR spectrum of **11a2** measured in DMSO-d_6 at 400 MHz.

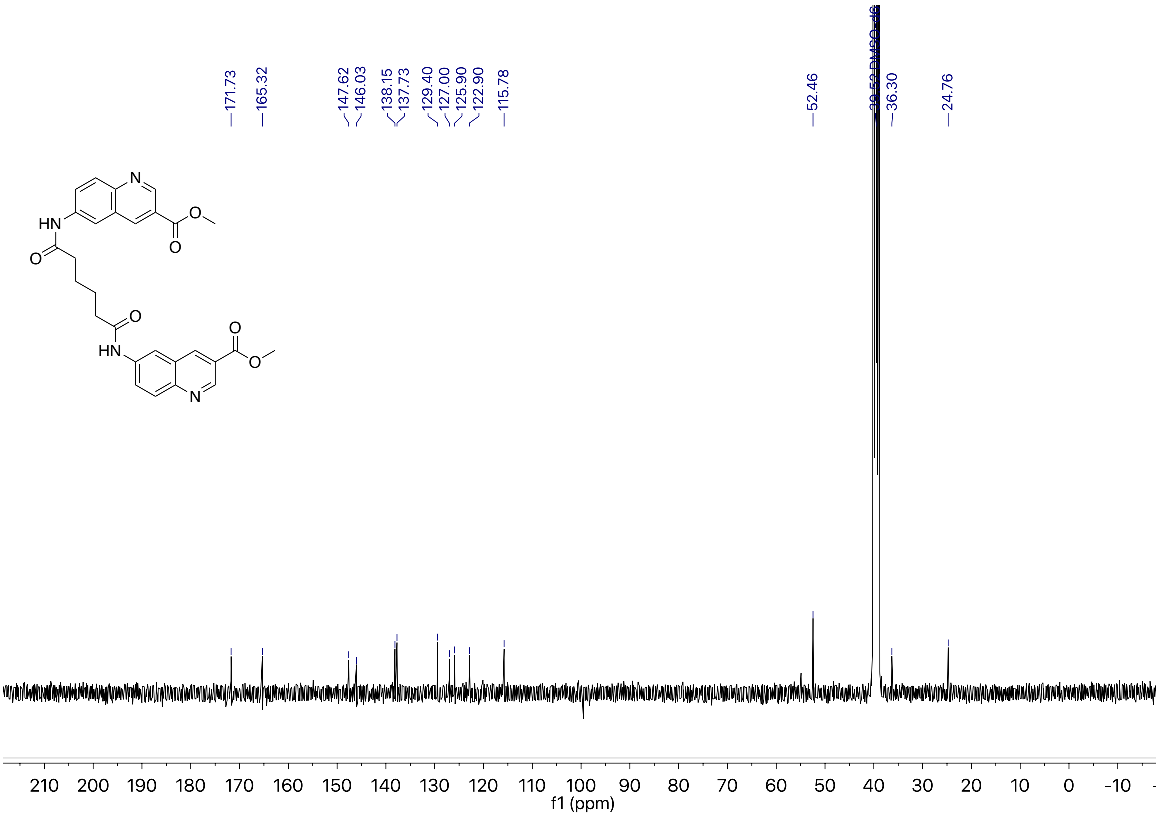

^{13}C NMR spectrum of **11a2** measured in DMSO-d_6 at 100 MHz.

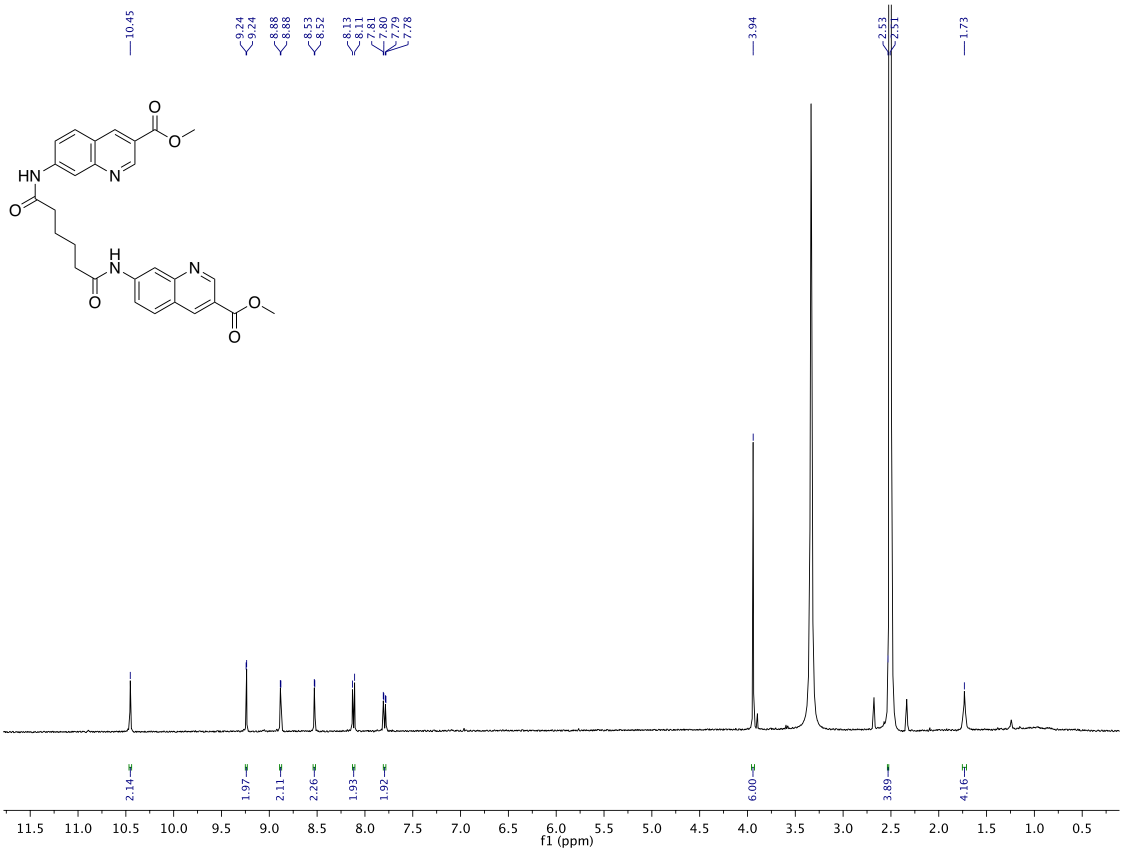

^{1}H NMR spectrum of **11b2** measured in DMSO-d_6 at 400 MHz.

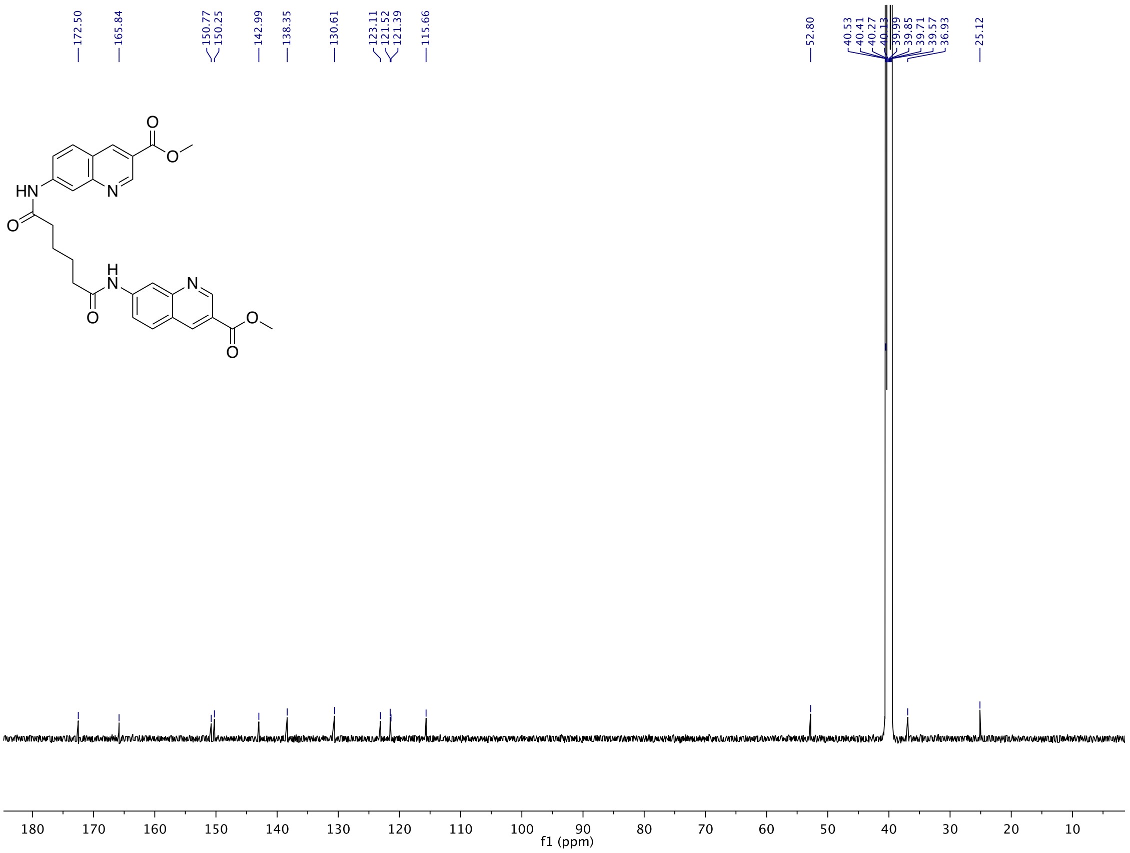

^{13}C NMR spectrum of **11b2** measured in DMSO-d_6 at 150 MHz.

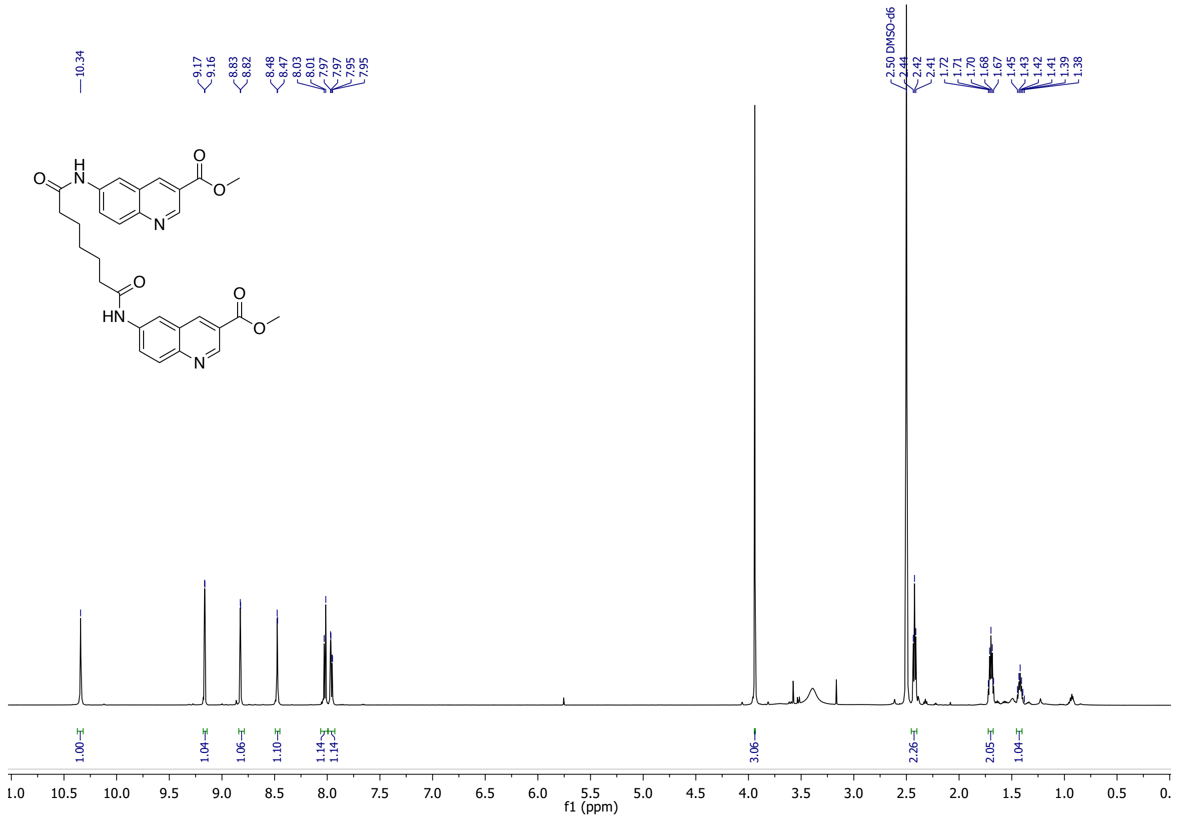

^{1}H NMR spectrum of **11a3** measured in DMSO-d_6 at 600 MHz.

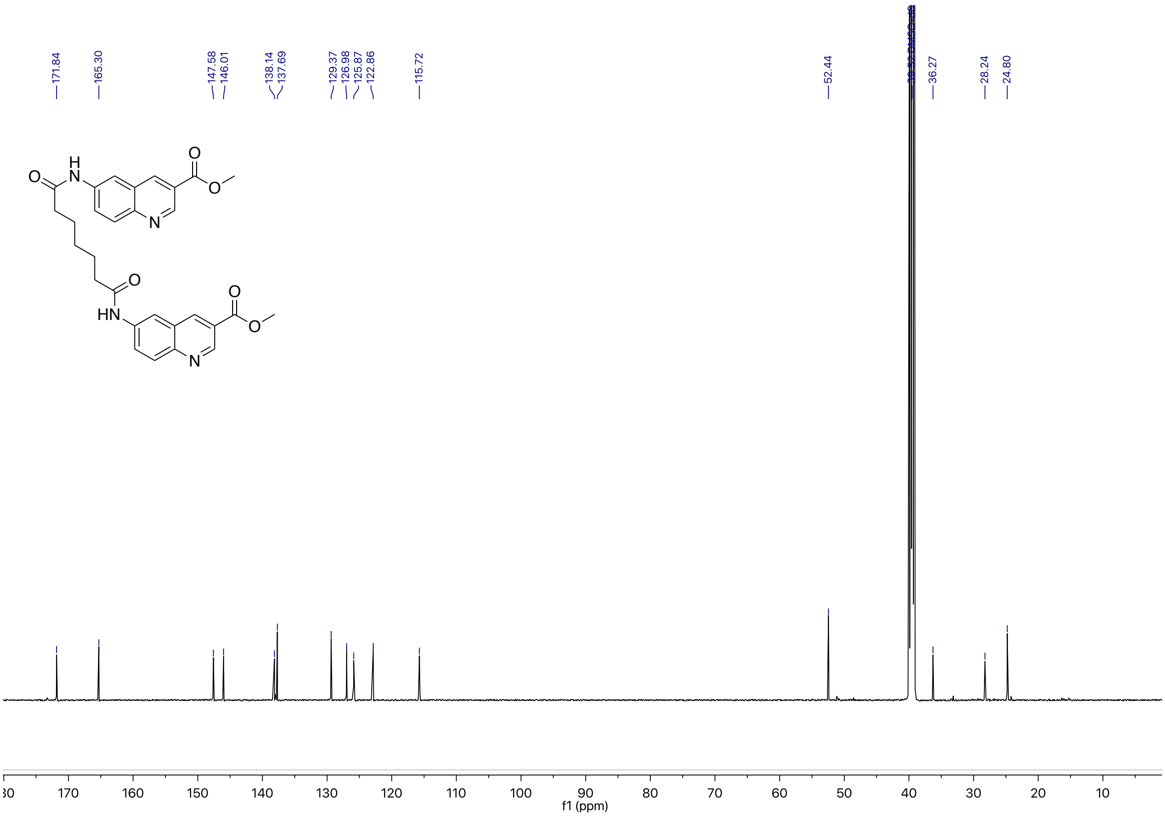

^{13}C NMR spectrum of **11a3** measured in DMSO-d_6 at 151 MHz.

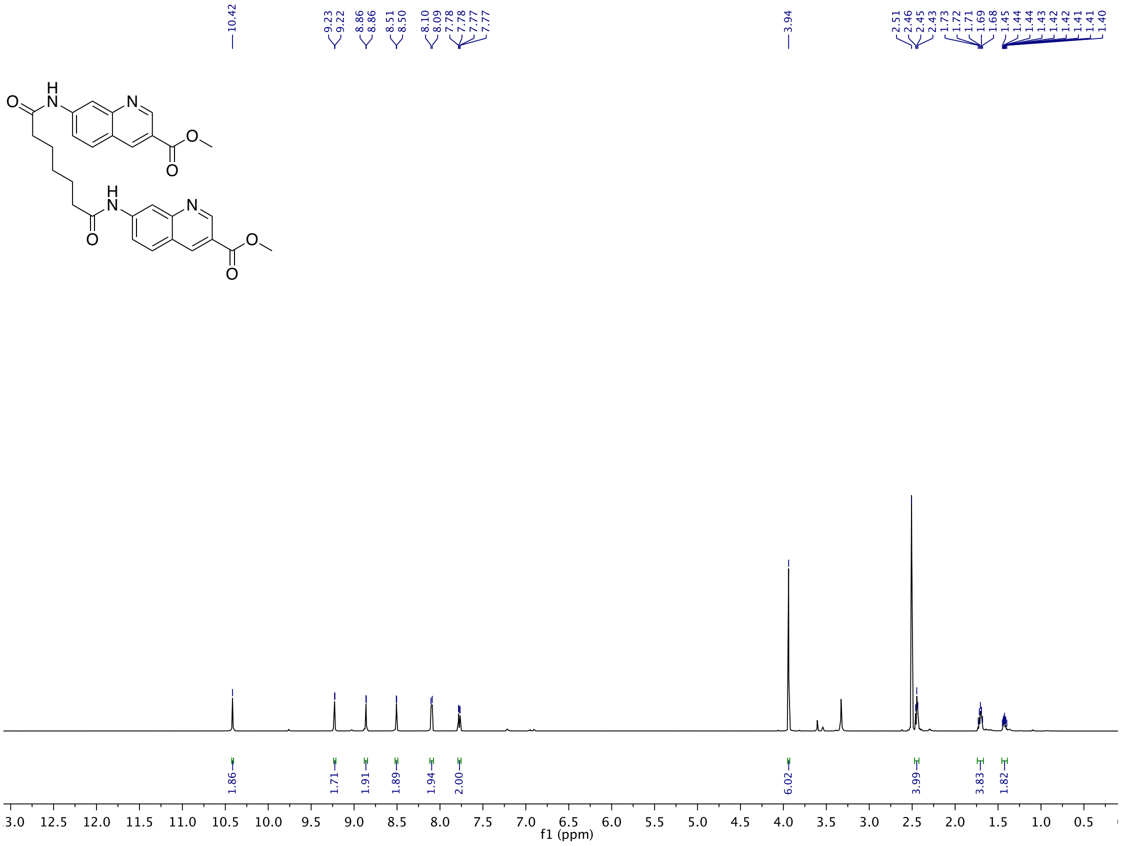

^{1}H NMR spectrum of **11b3** measured in DMSO-d_6 at 600 MHz.

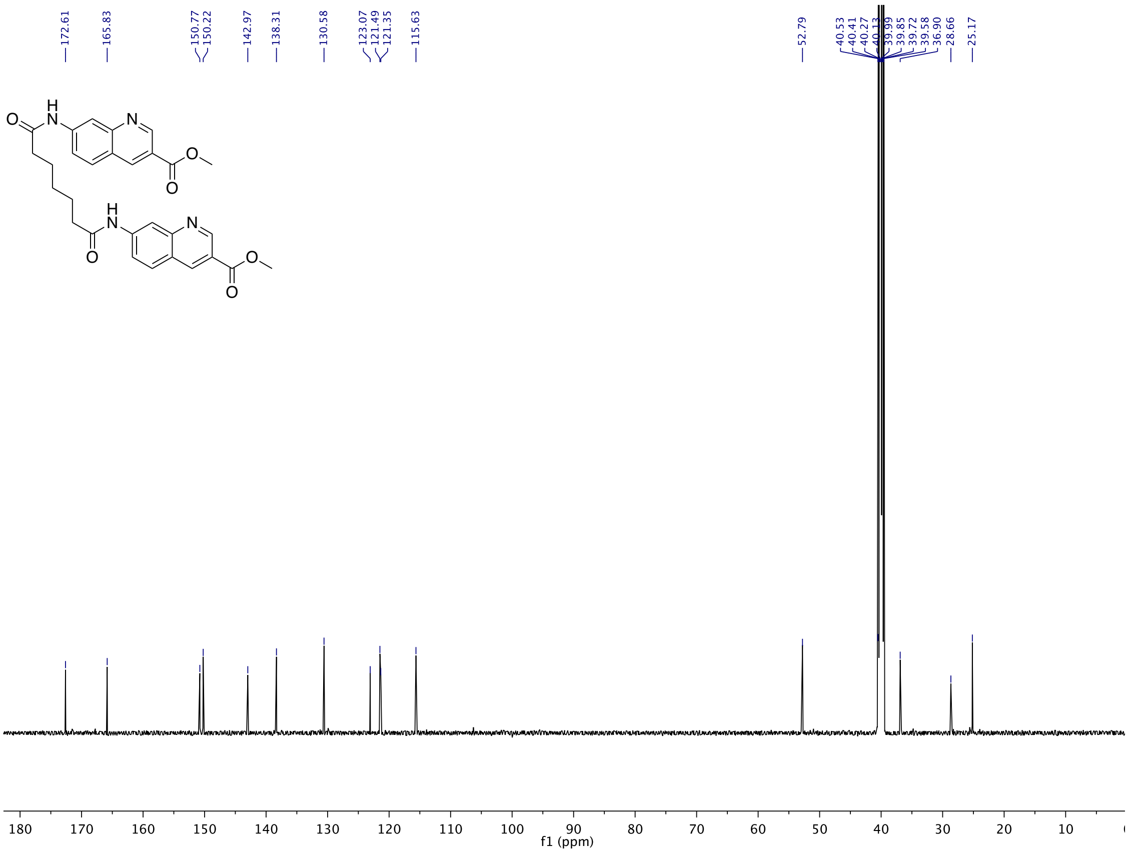

^{13}C NMR spectrum of **11b3** measured in DMSO-d_6 at 151 MHz.

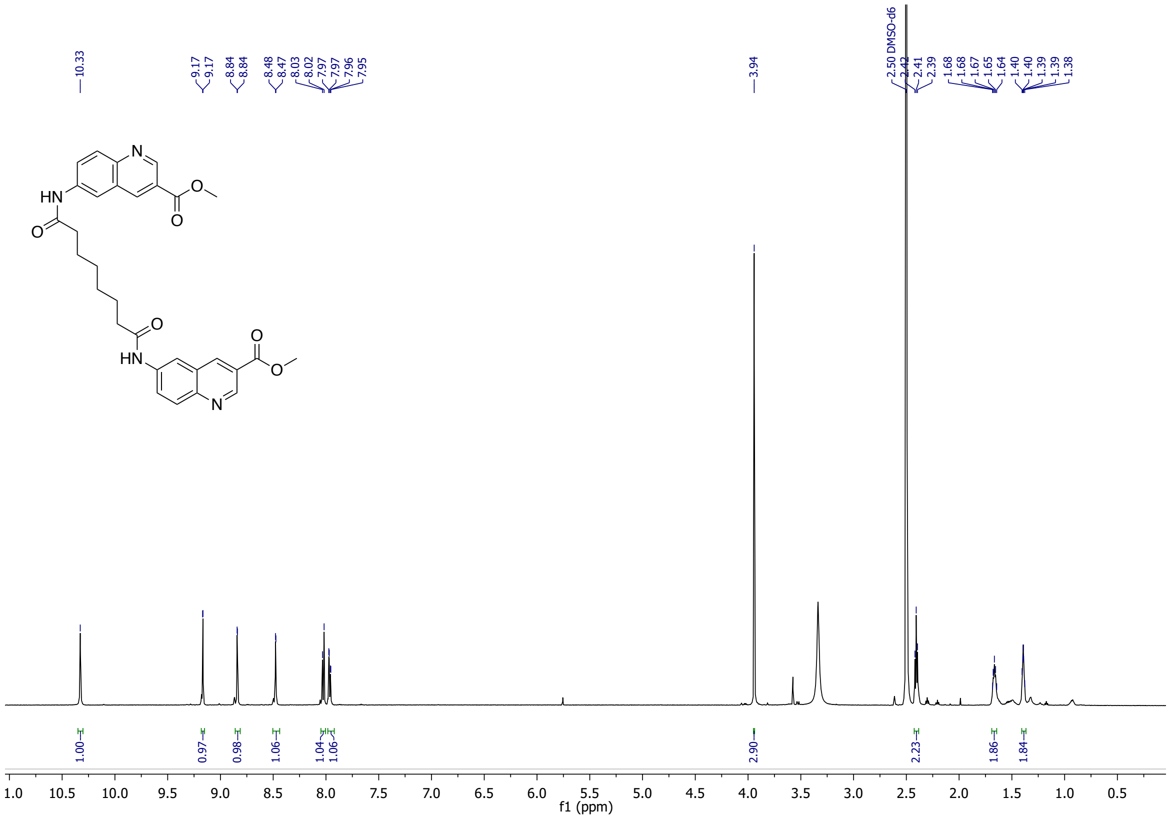

^{1}H NMR spectrum of **11a4** measured in DMSO-d_6 at 600 MHz.

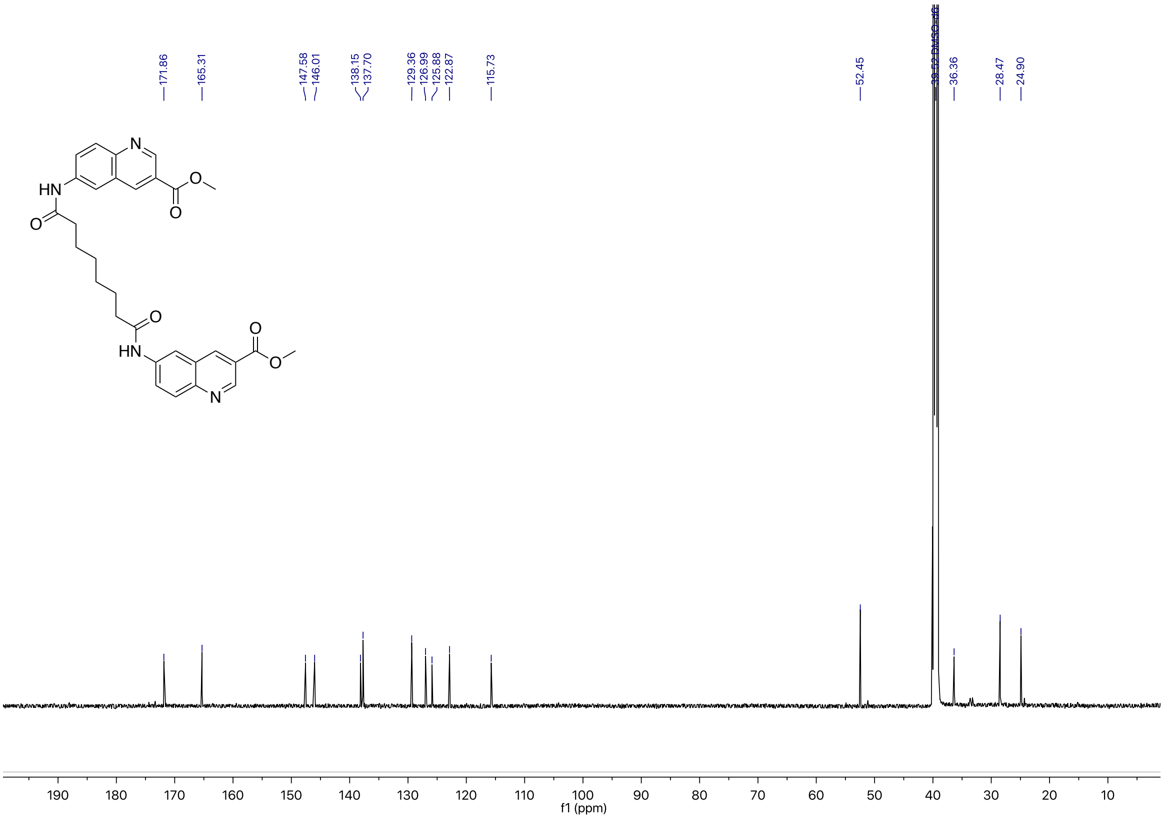

^{13}C NMR spectrum of **11a4** measured in DMSO-d_6 at 151 MHz.

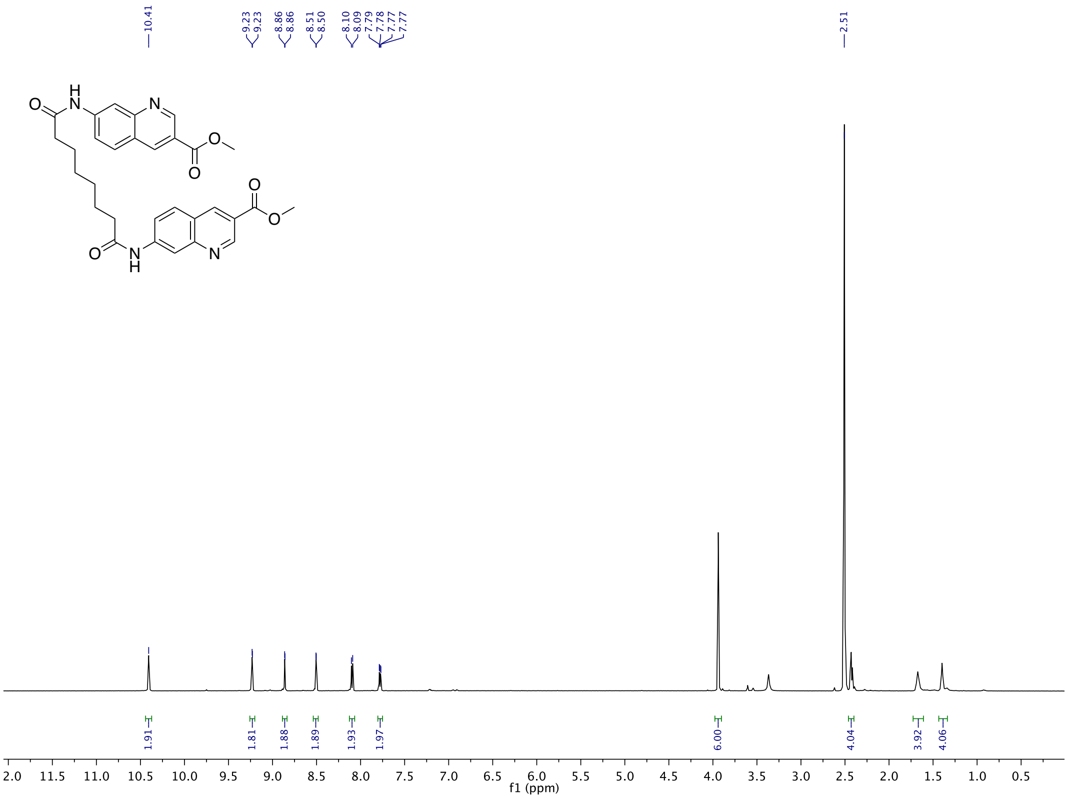

^{1}H NMR spectrum of **11b4** measured in DMSO-d_6 at 600 MHz.

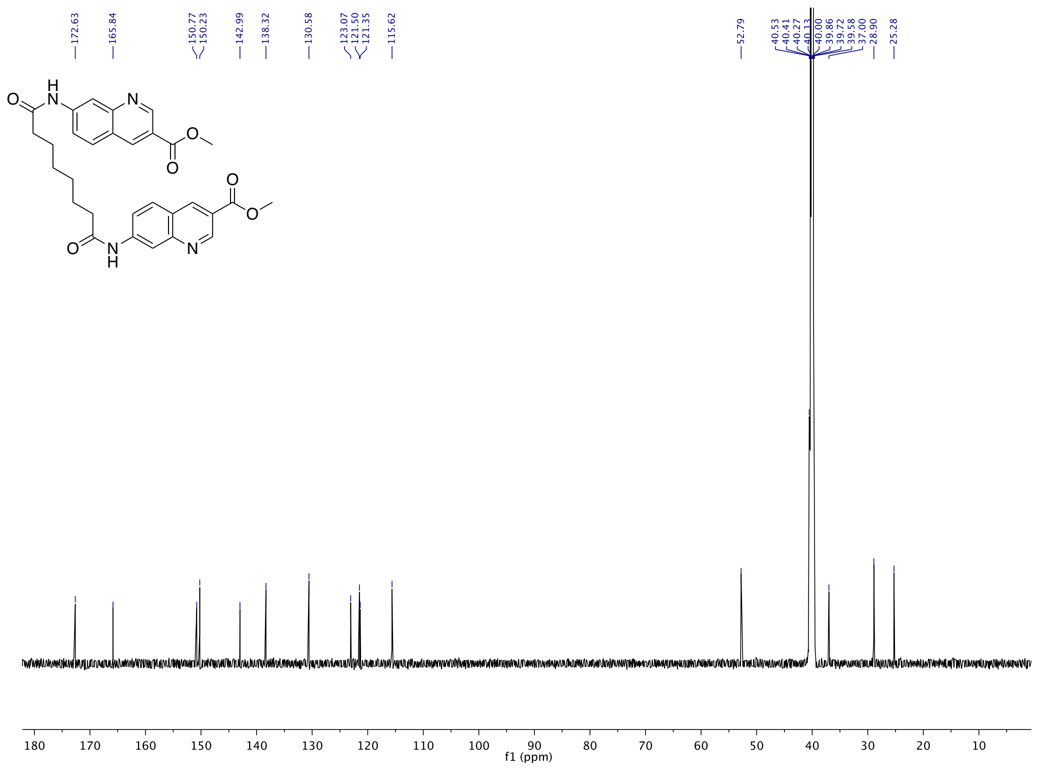

^{13}C NMR spectrum of **11b4** measured in DMSO-d_6 at 150 MHz.

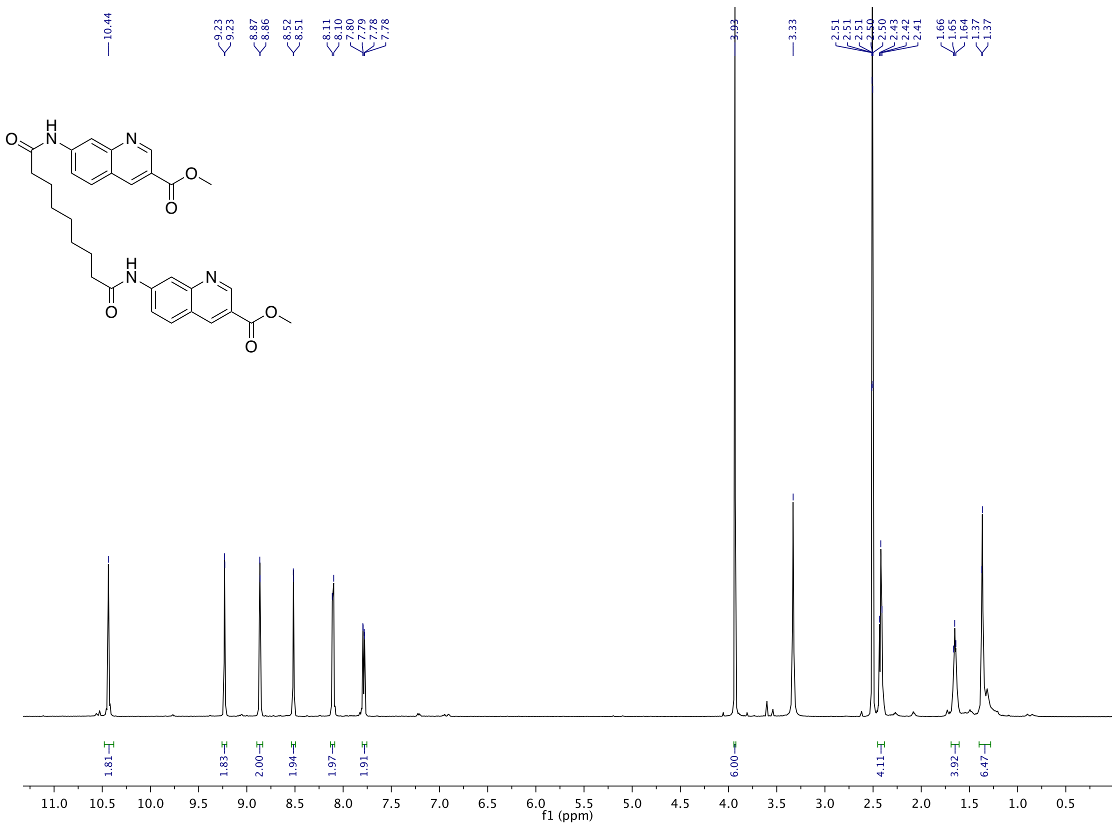

^{1}H NMR spectrum of **11b5** measured in DMSO-d_6 at 600 MHz.

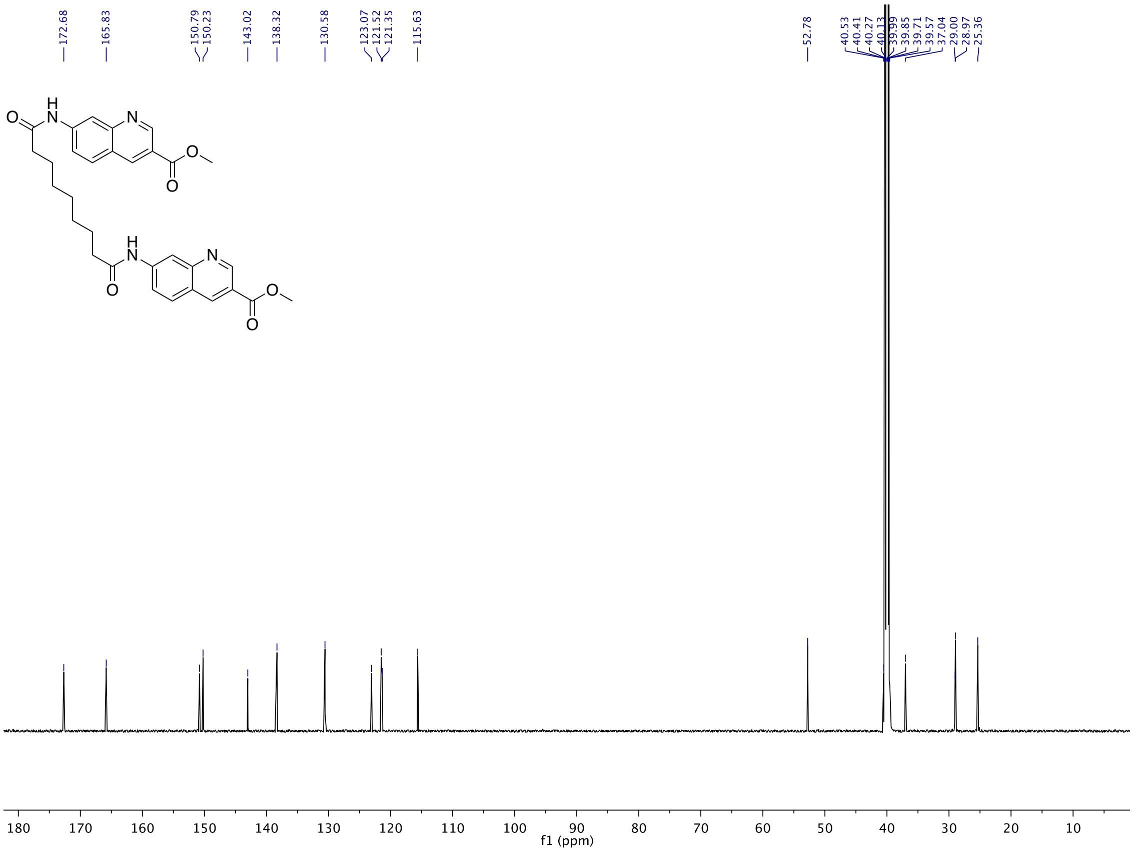

^{13}C NMR spectrum of **11b5** measured in DMSO-d_6 at 150 MHz.

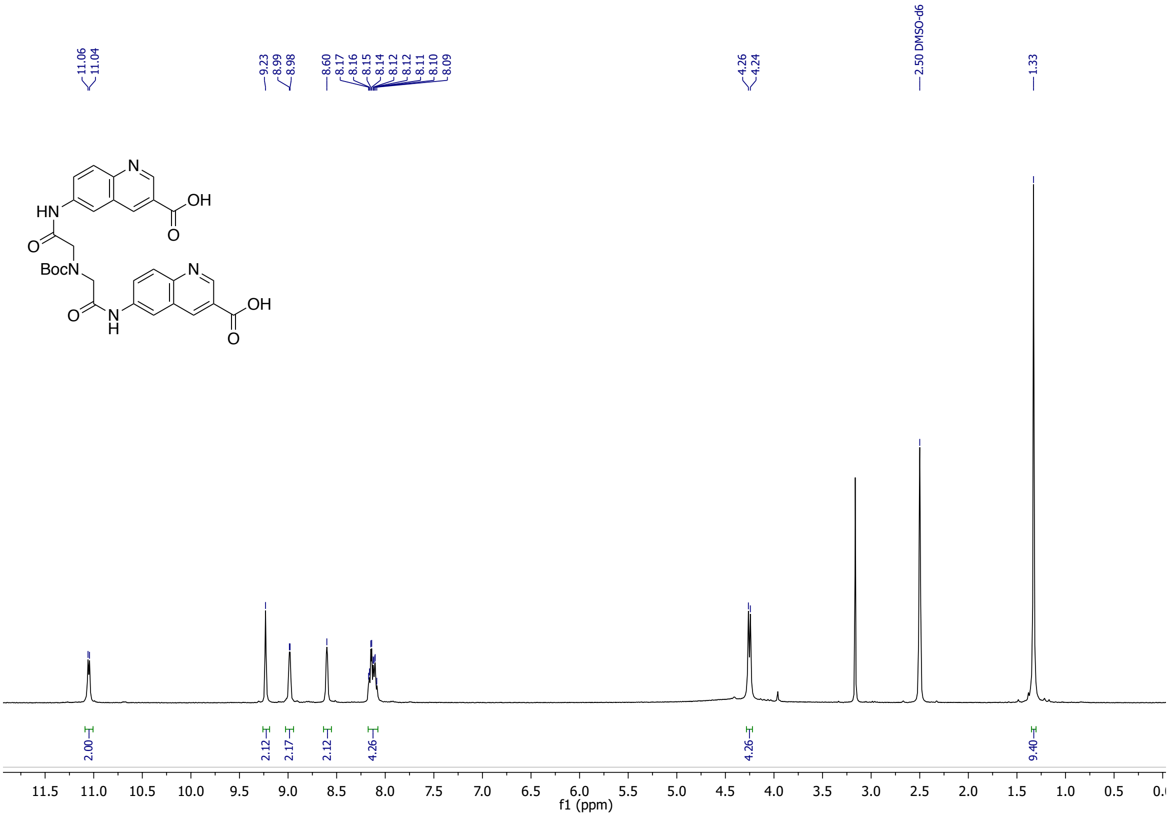

^{1}H NMR spectrum of **12a1** measured in DMSO-d_6 at 400 MHz.

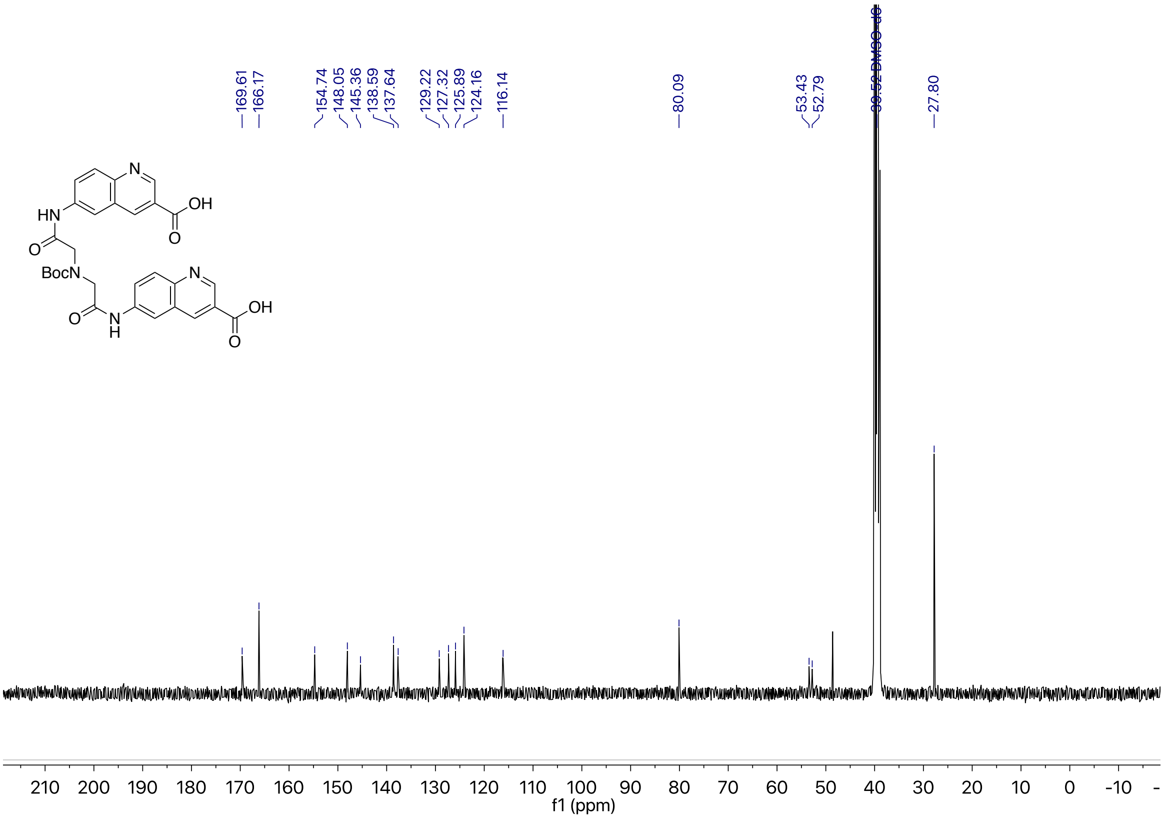

^{13}C NMR spectrum of **12a1** measured in DMSO-d_6 at 100 MHz.

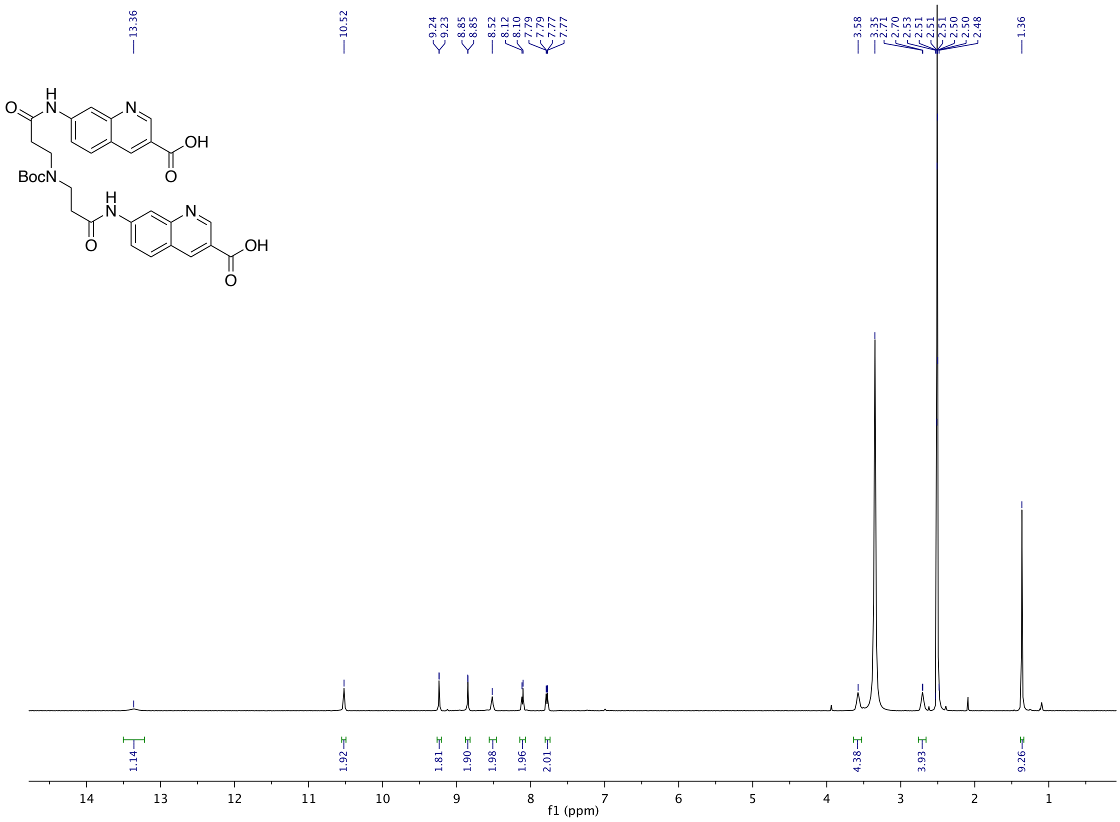

^{1}H NMR spectrum of **12b1** measured in DMSO-d_6 at 600 MHz.

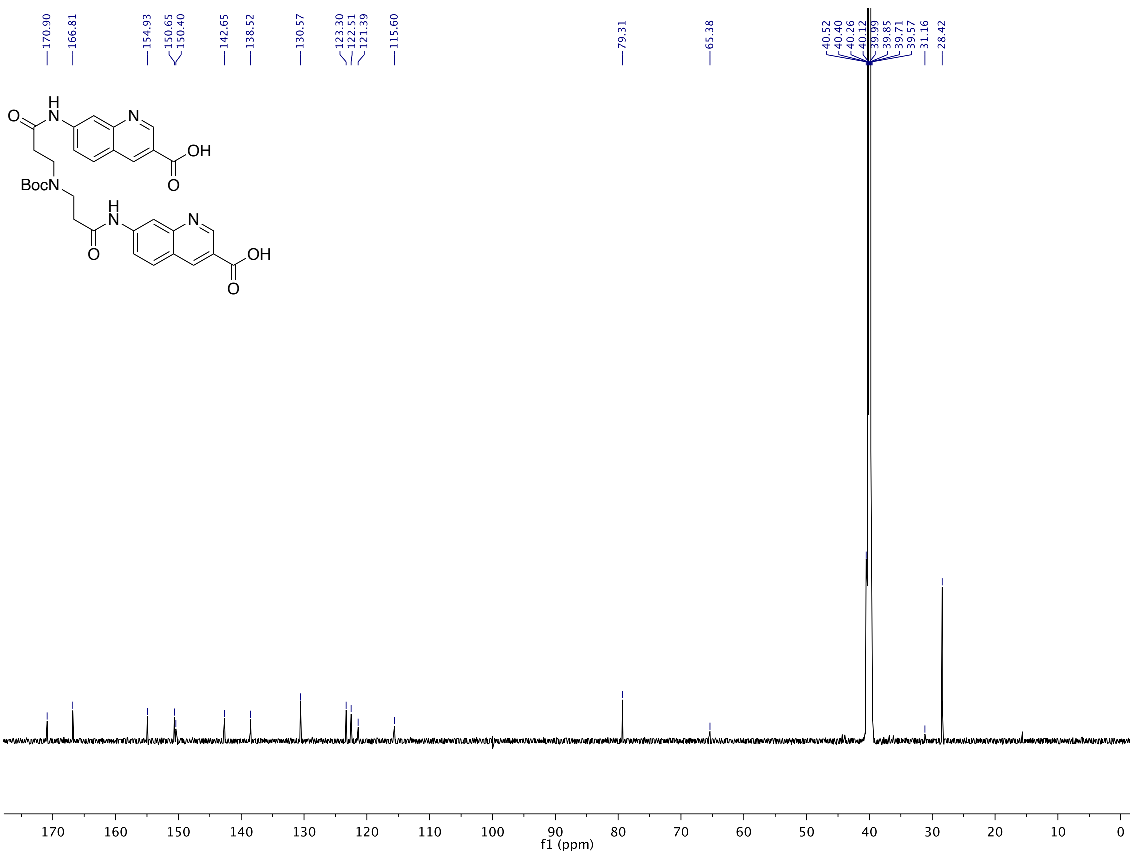

^{13}C NMR spectrum of **12b1** measured in DMSO-d_6 at 150 MHz.

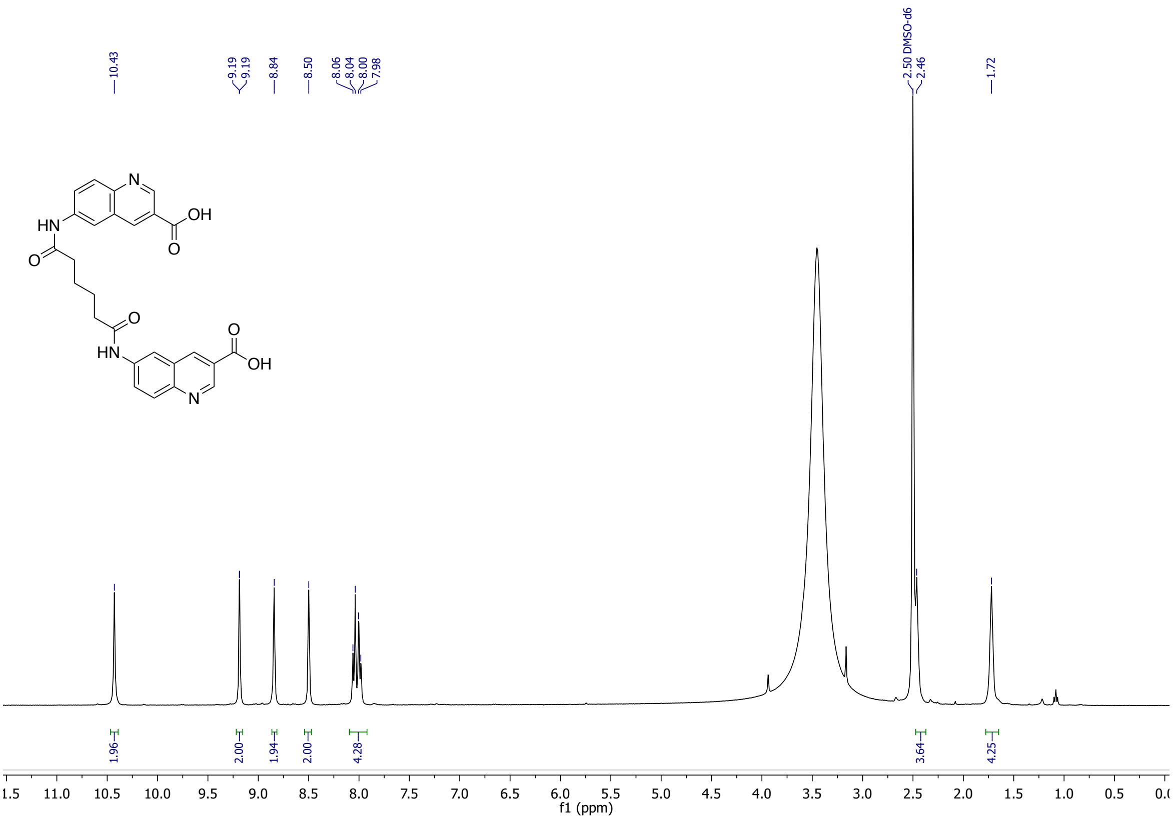

^1H NMR spectrum of **12a2** measured in DMSO-d_6 at 400 MHz.

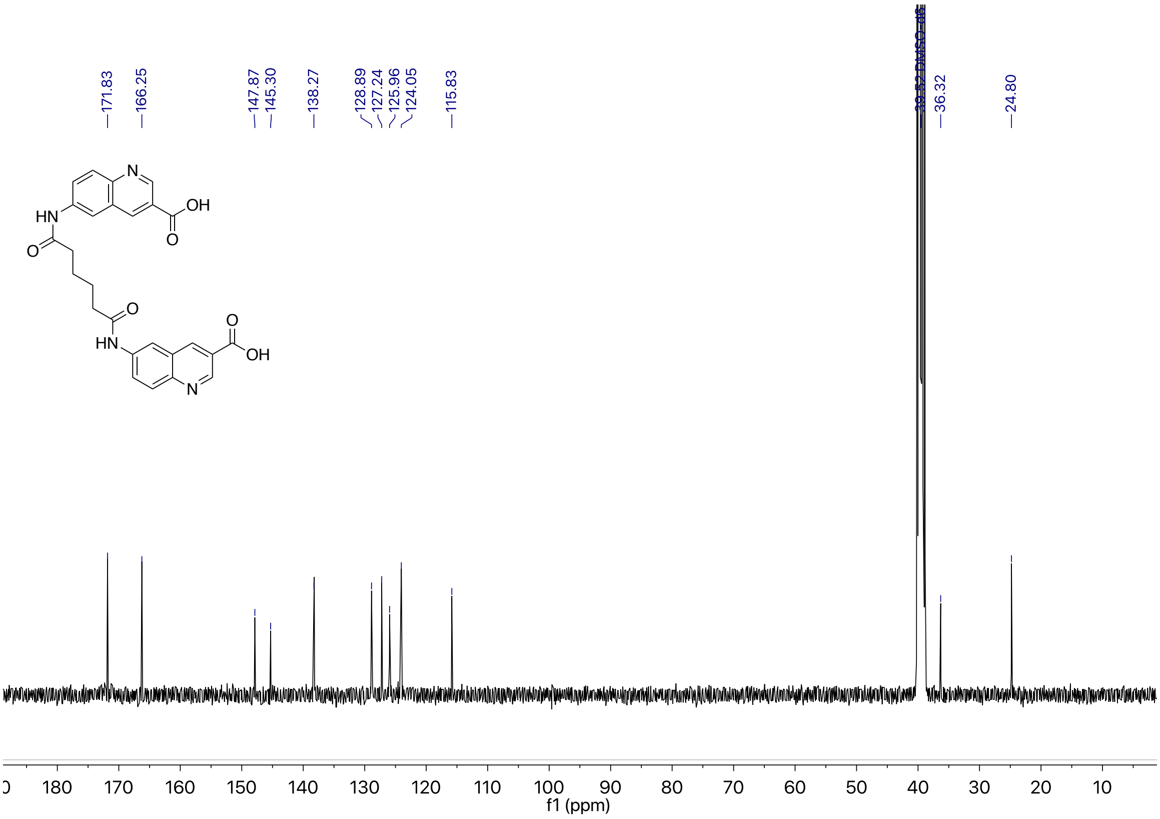

^{13}C NMR spectrum of **12a2** measured in DMSO-d_6 at 100 MHz.

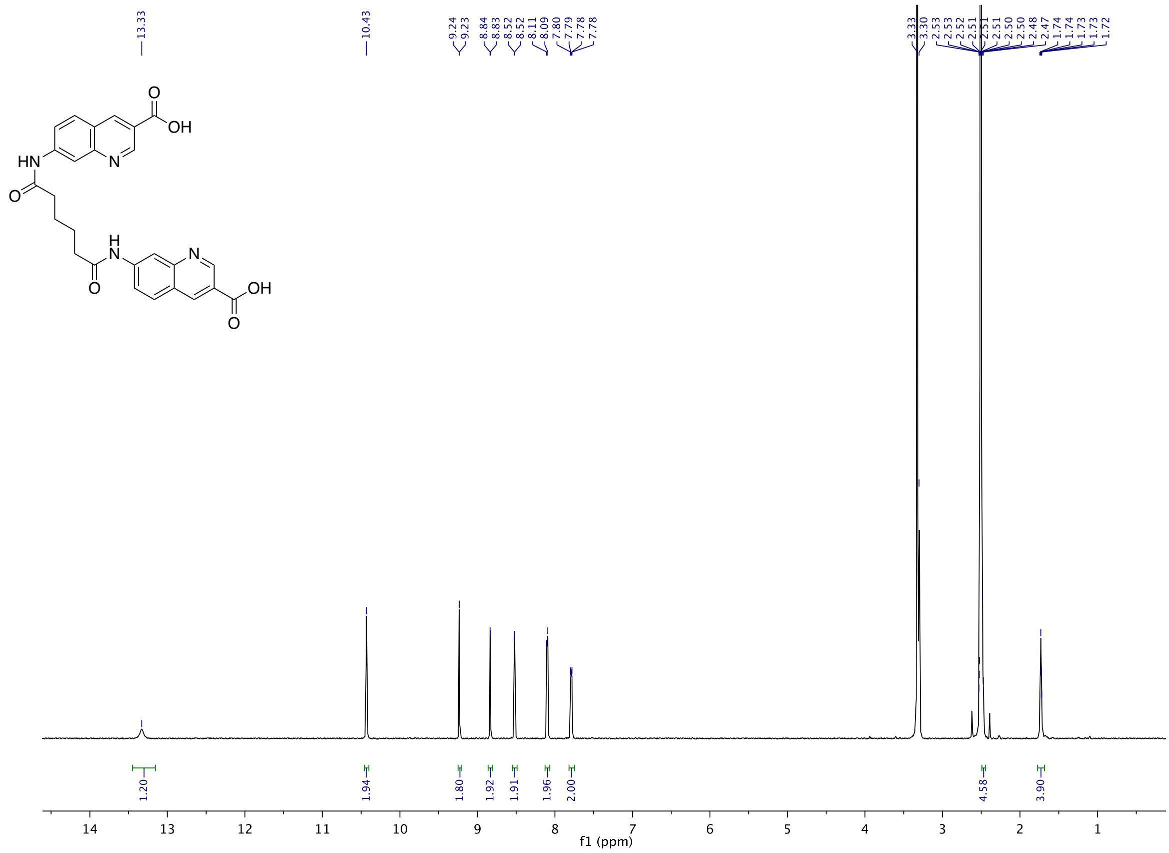

^{1}H NMR spectrum of **12b** measured in DMSO-d_6 at 600 MHz.

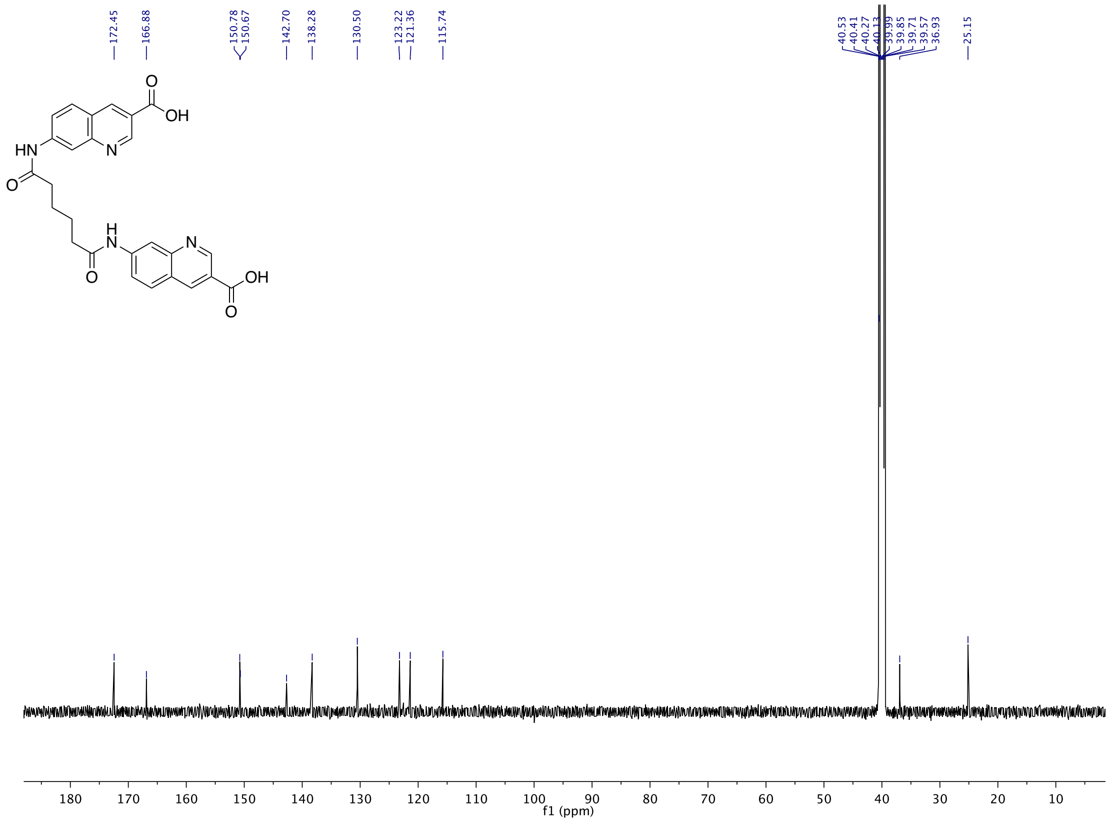

^{13}C NMR spectrum of **12b2** measured in DMSO-d_6 at 150 MHz.

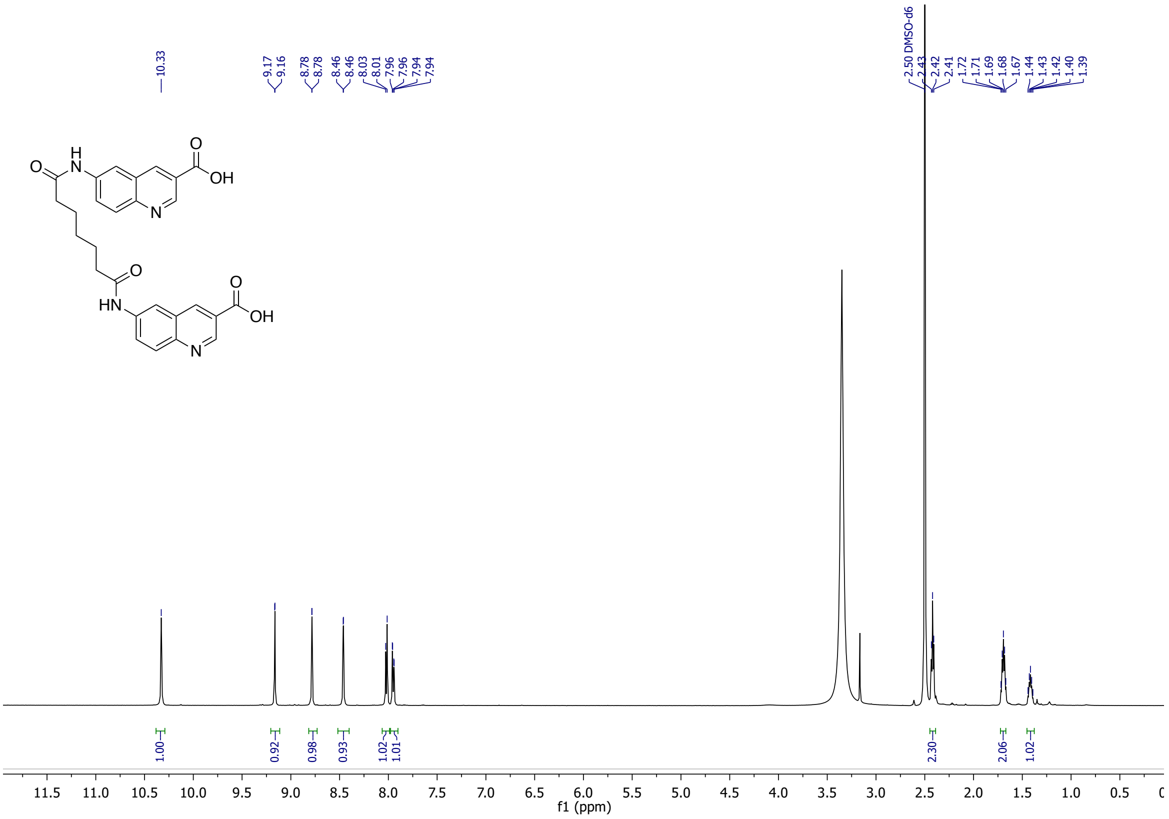

^{1}H NMR spectrum of **12a3** measured in DMSO-d_6 at 600 MHz.

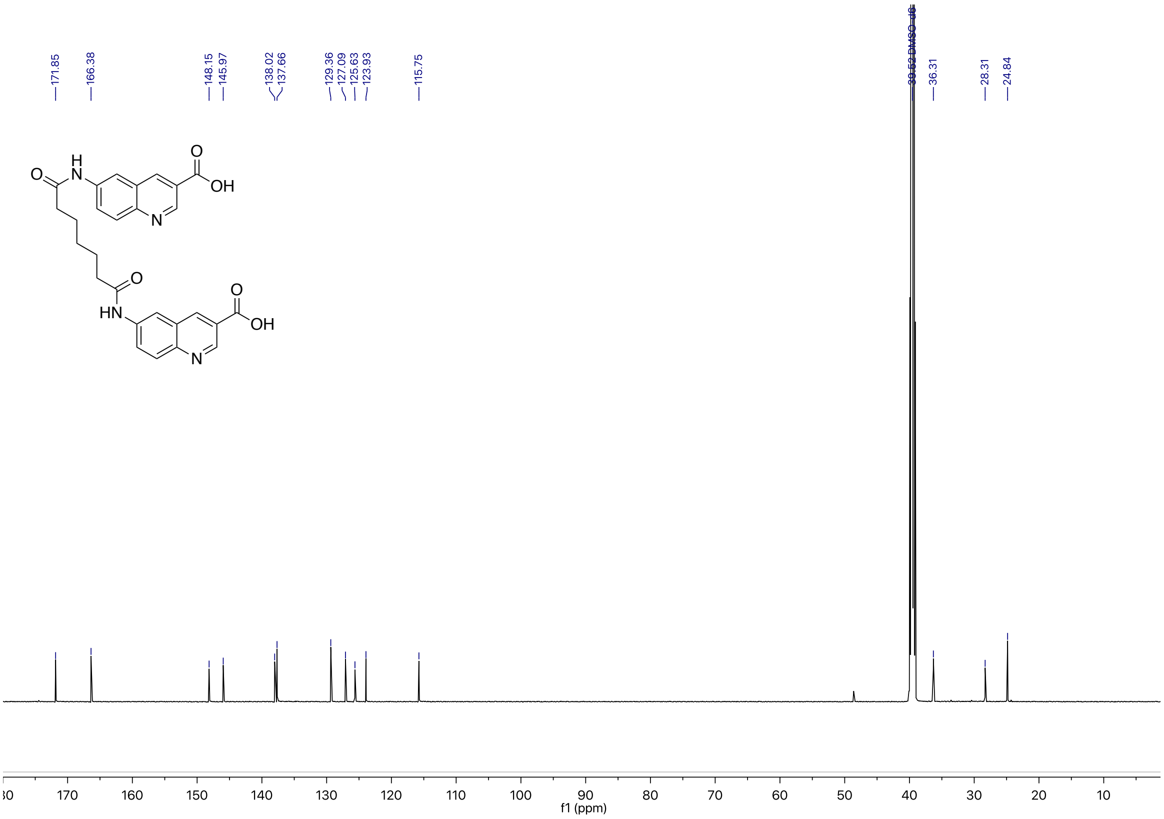

^{13}C NMR spectrum of **12a3** measured in DMSO-d_6 at 151 MHz.

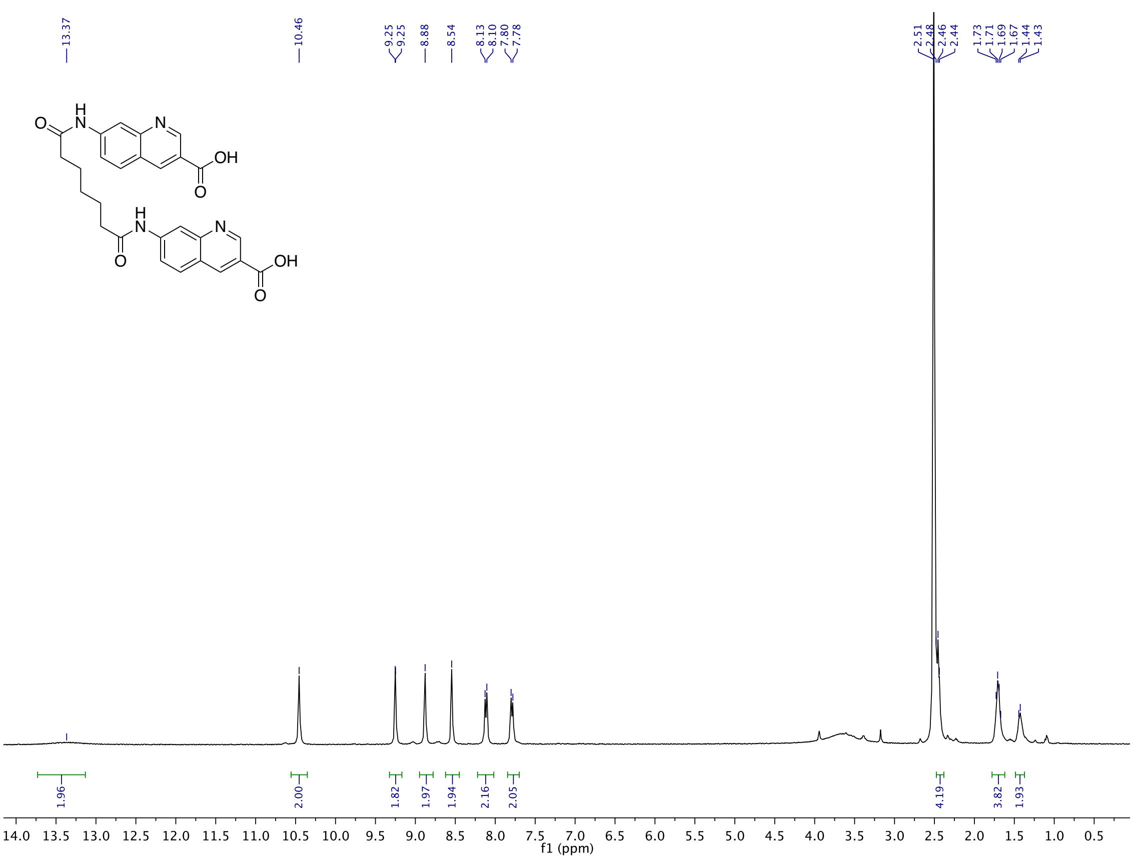

^{1}H NMR spectrum of **12b3** measured in DMSO-d_6 at 400 MHz.

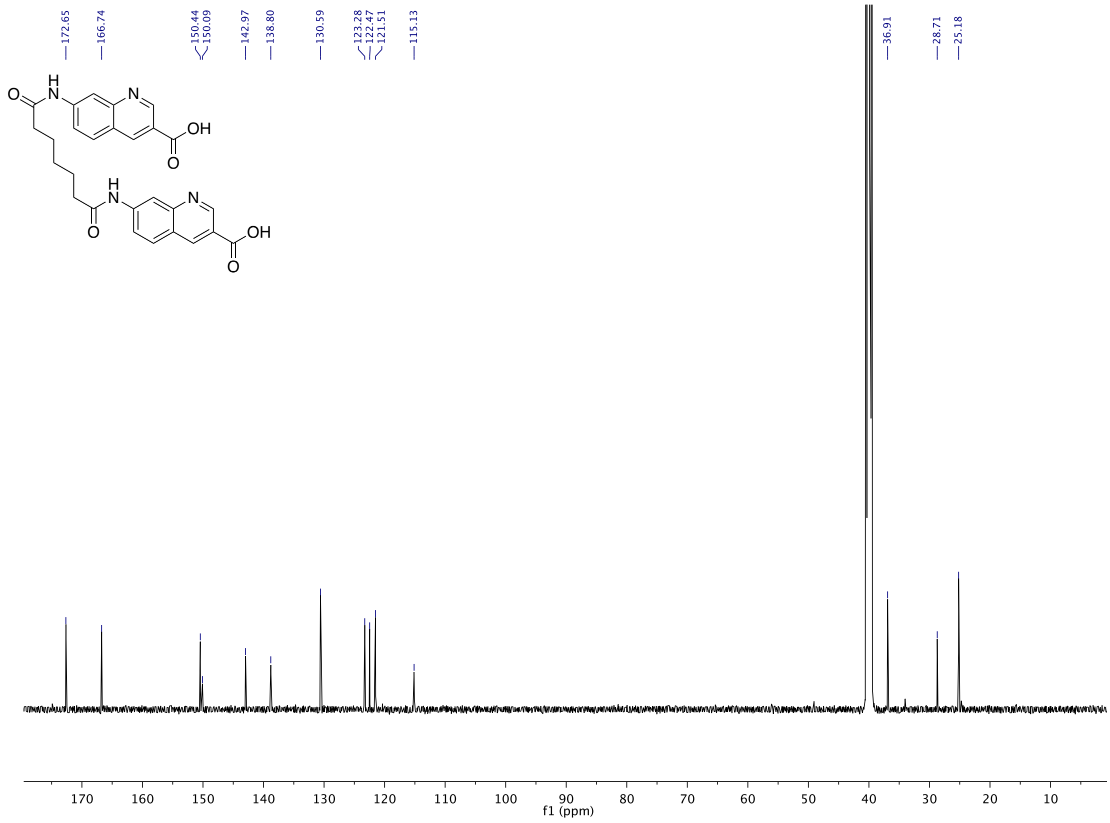

^{13}C NMR spectrum of **12b3** measured in DMSO-d_6 at 150 MHz.

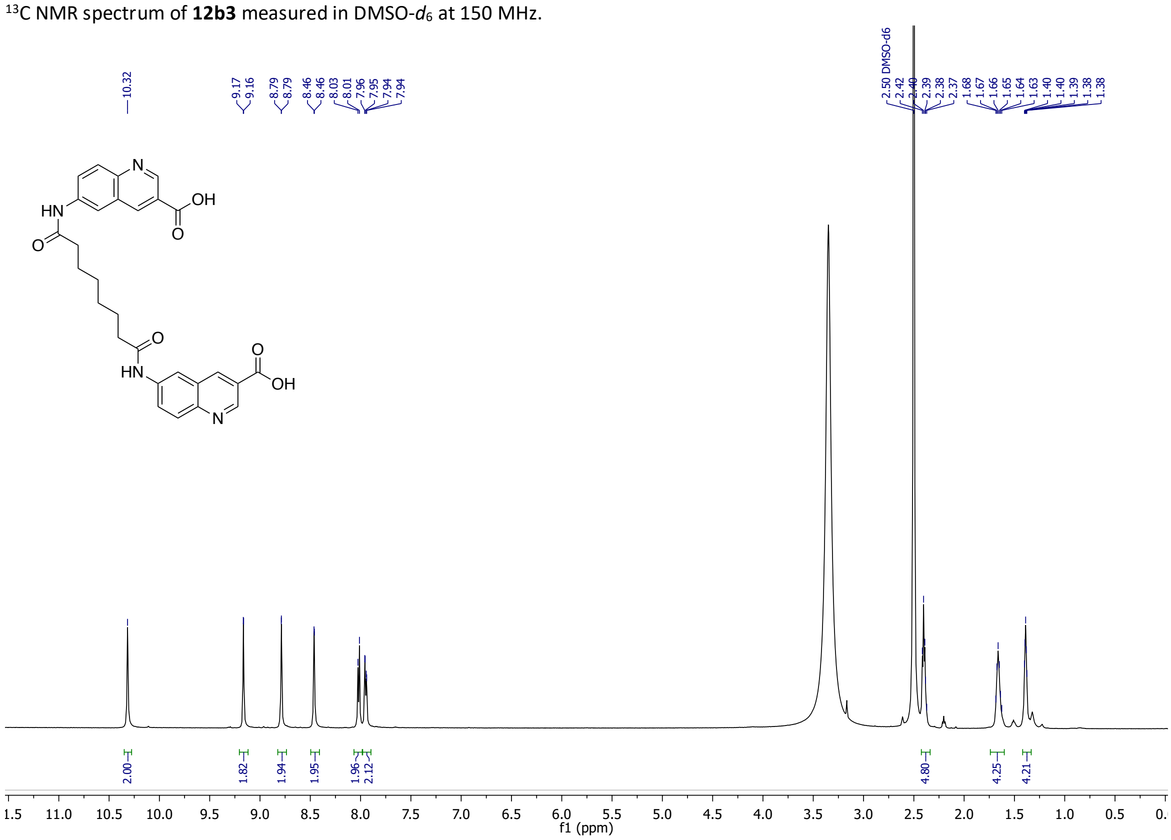

^{1}H NMR spectrum of **12a4** measured in DMSO-d_6 at 600 MHz.

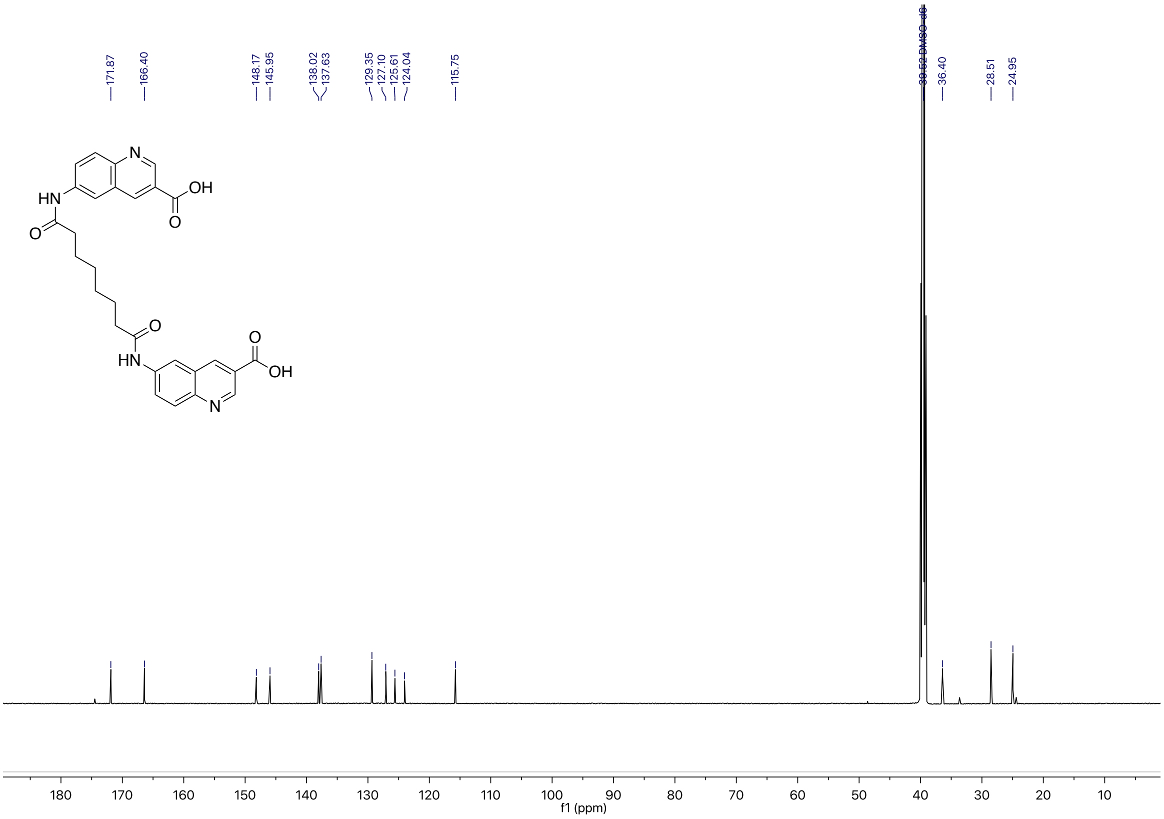

^{13}C NMR spectrum of **12a4** measured in DMSO-d_6 at 151 MHz.

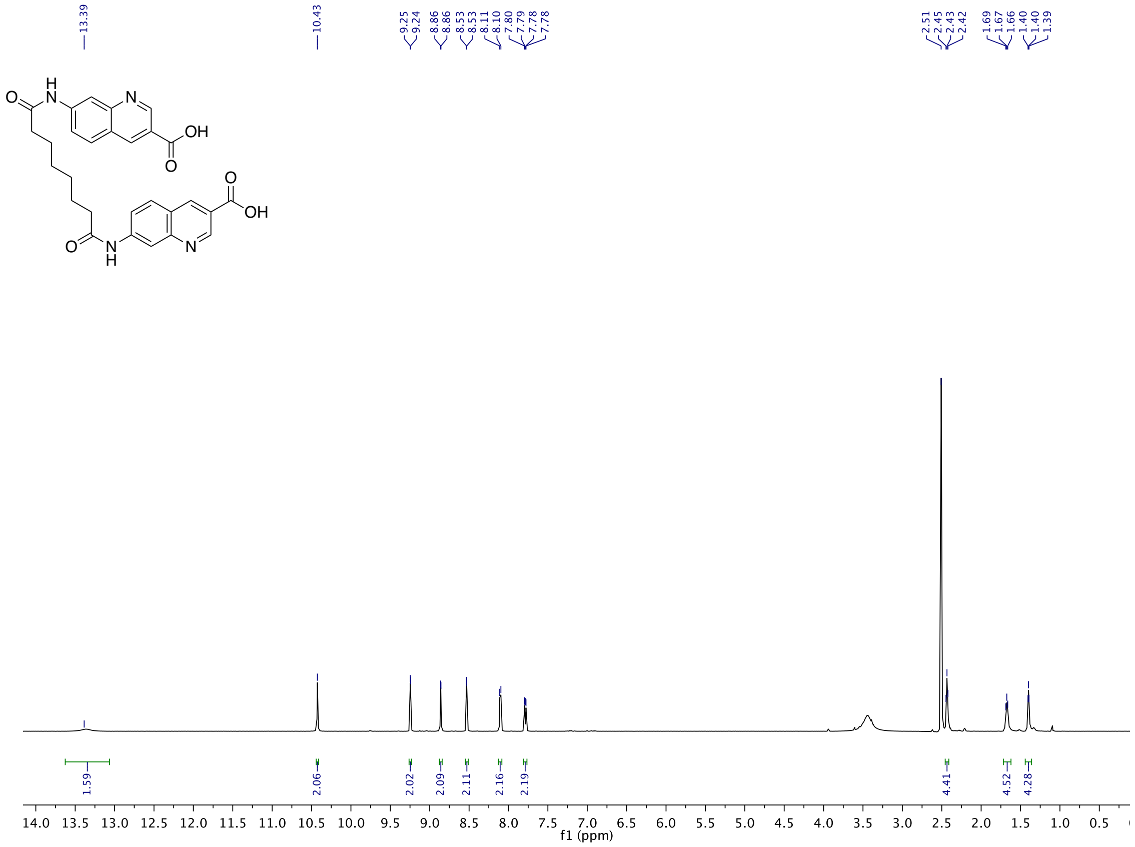

^{1}H NMR spectrum of **12b4** measured in DMSO-d_6 at 600 MHz.

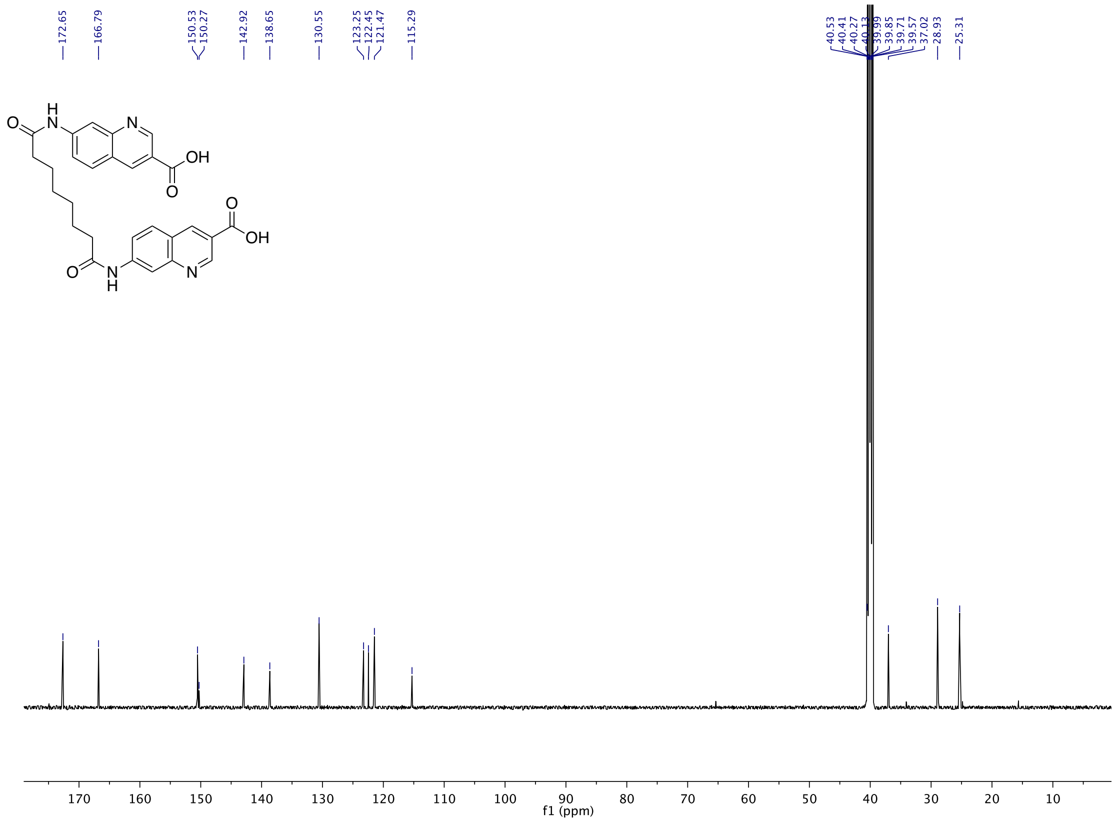

^{13}C NMR spectrum of **12b4** measured in DMSO-d_6 at 151 MHz.

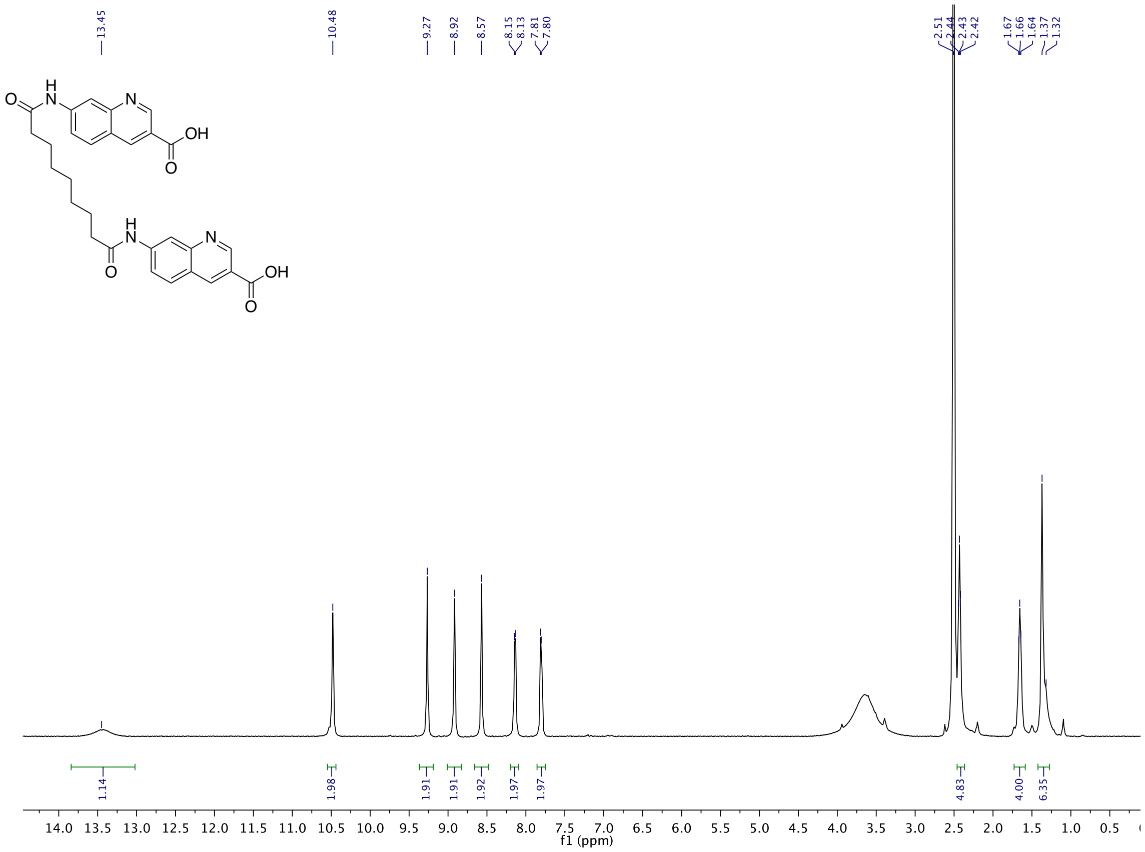

^{1}H NMR spectrum of **12b5** measured in DMSO-d_6 at 600 MHz.

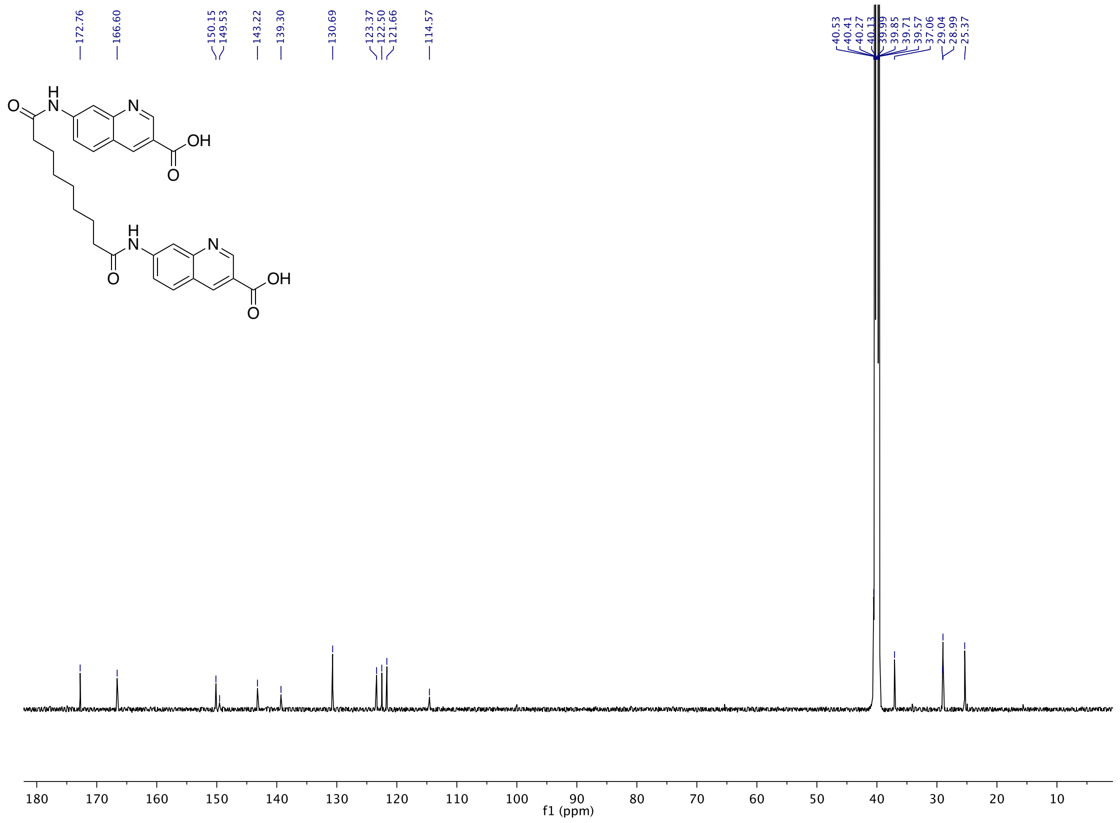

^{13}C NMR spectrum of **12b5** measured in DMSO-d_6 at 151 MHz.

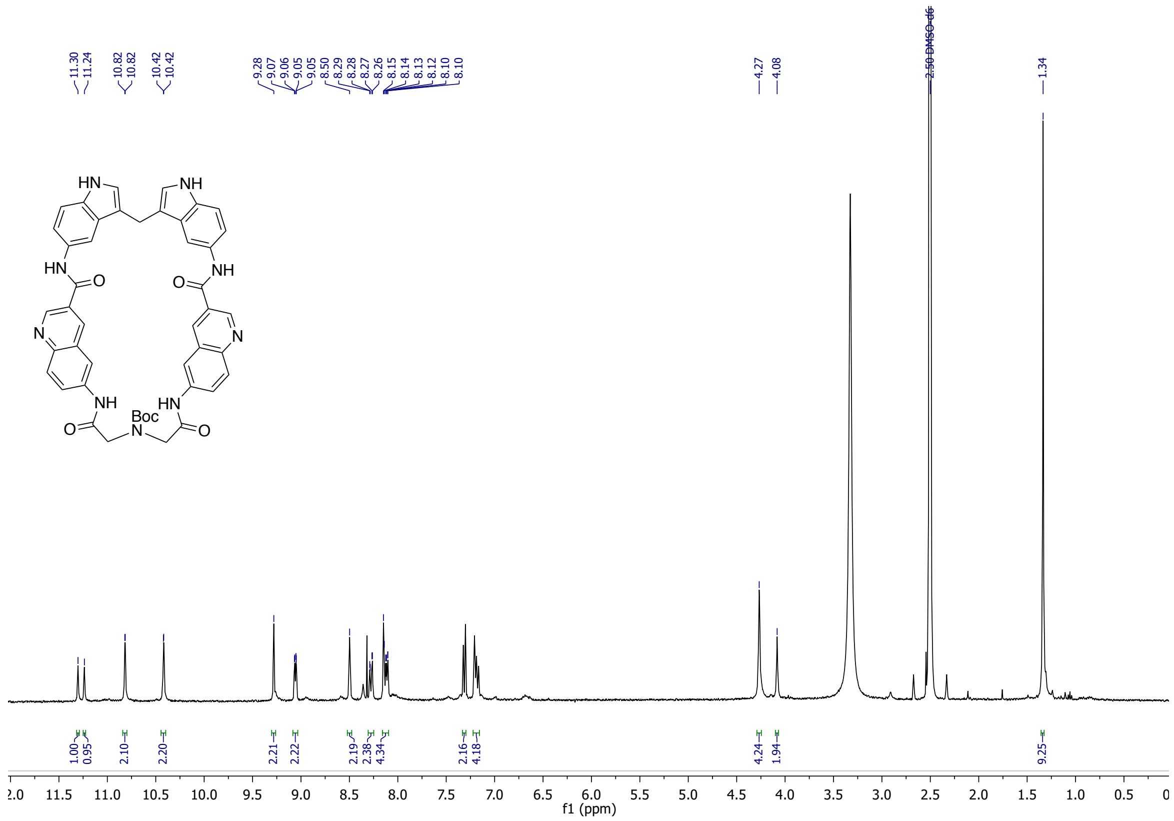

^{1}H NMR spectrum of **13a1** measured in DMSO-d_6 at 400 MHz.

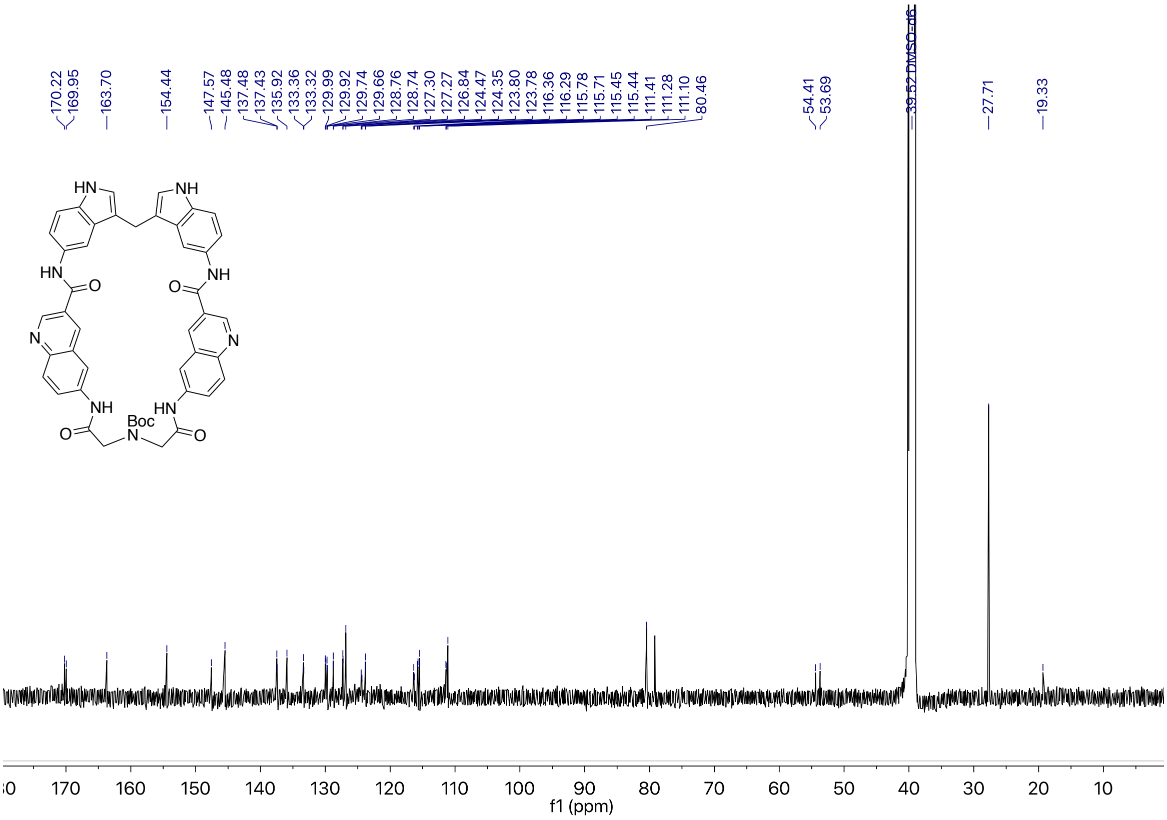

^{13}C NMR spectrum of **13a1** measured in DMSO-d_6 at 151 MHz.

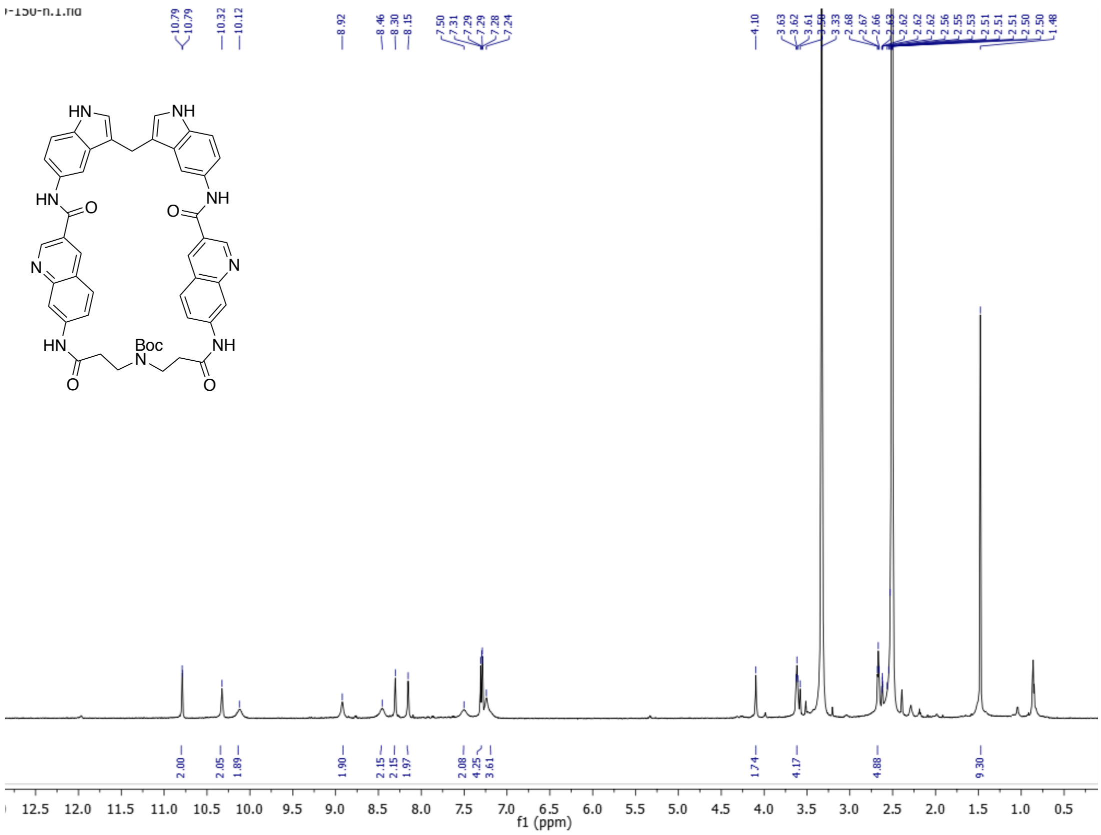

^{1}H NMR spectrum of **13b1** measured in DMSO-d_6 at 600 MHz.

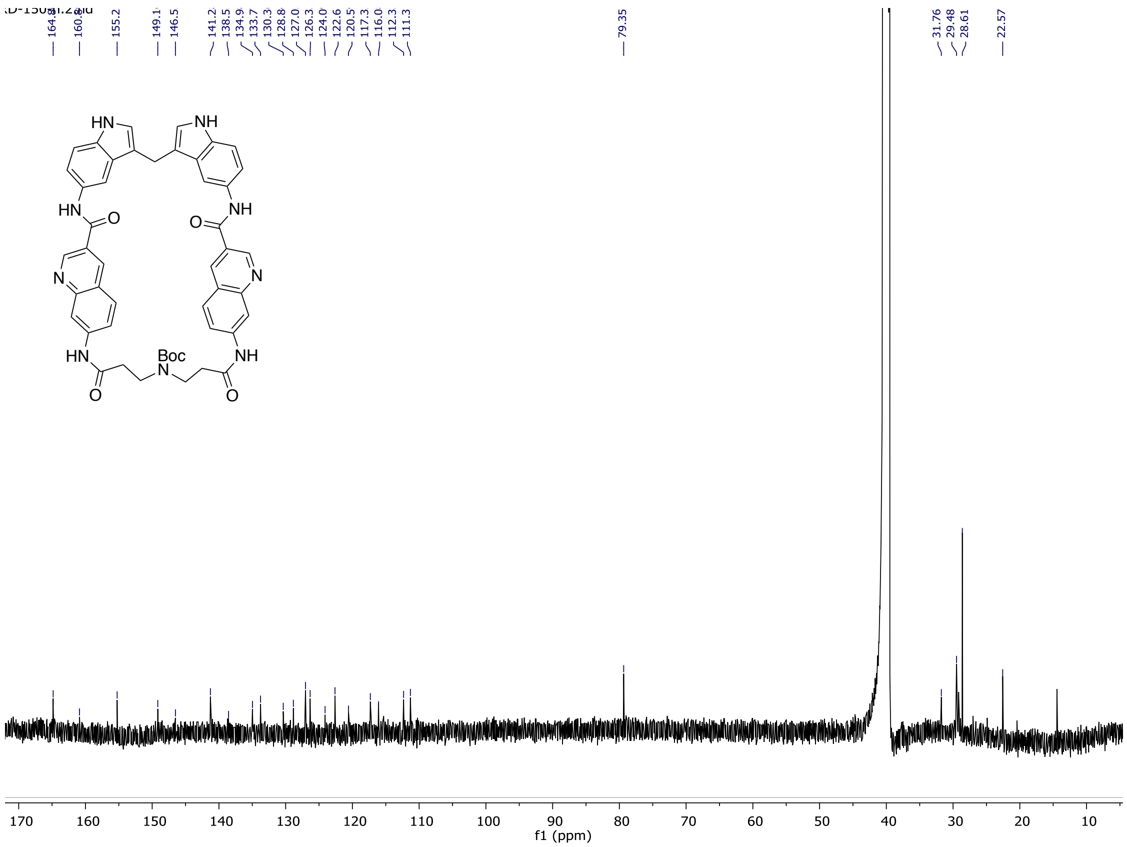

^{13}C NMR spectrum of **13b1** measured in DMSO-d_6 at 151 MHz.

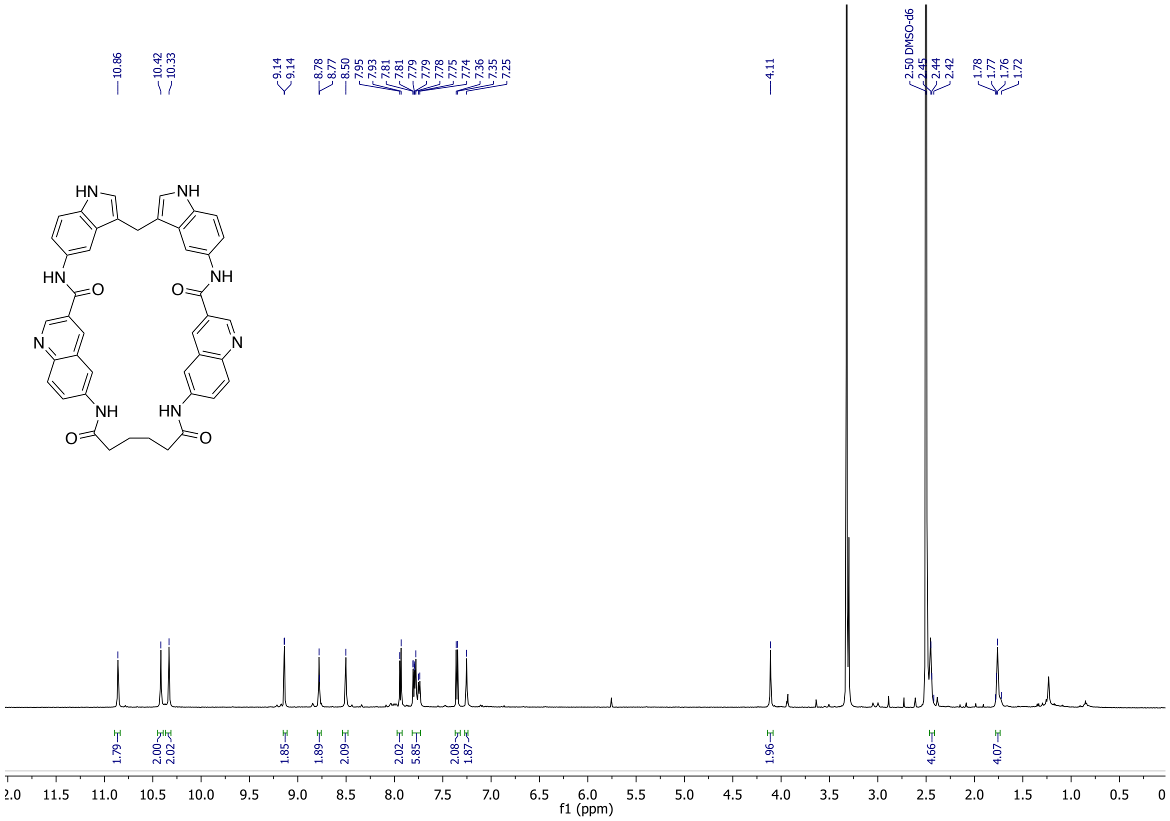

^{1}H NMR spectrum of **13a2** measured in DMSO-d_6 at 600 MHz.

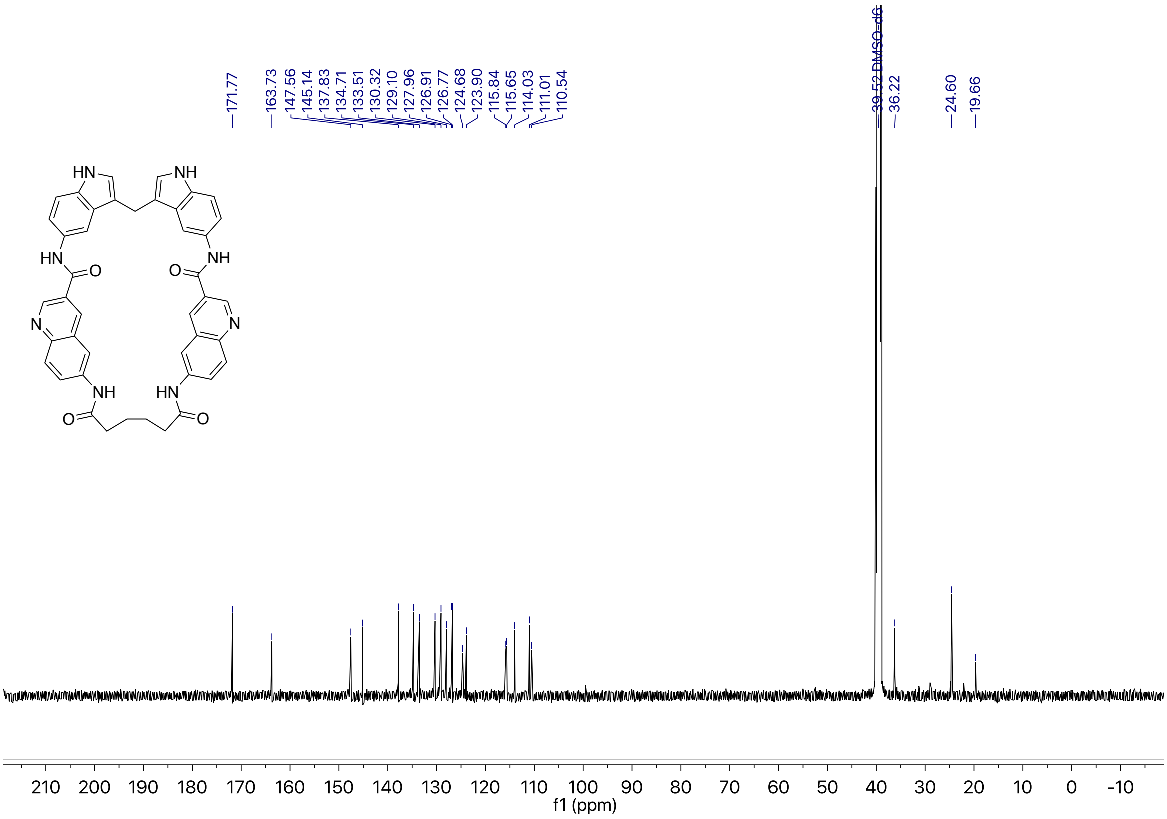

^{13}C NMR spectrum of **13a2** measured in DMSO-d_6 at 151 MHz.

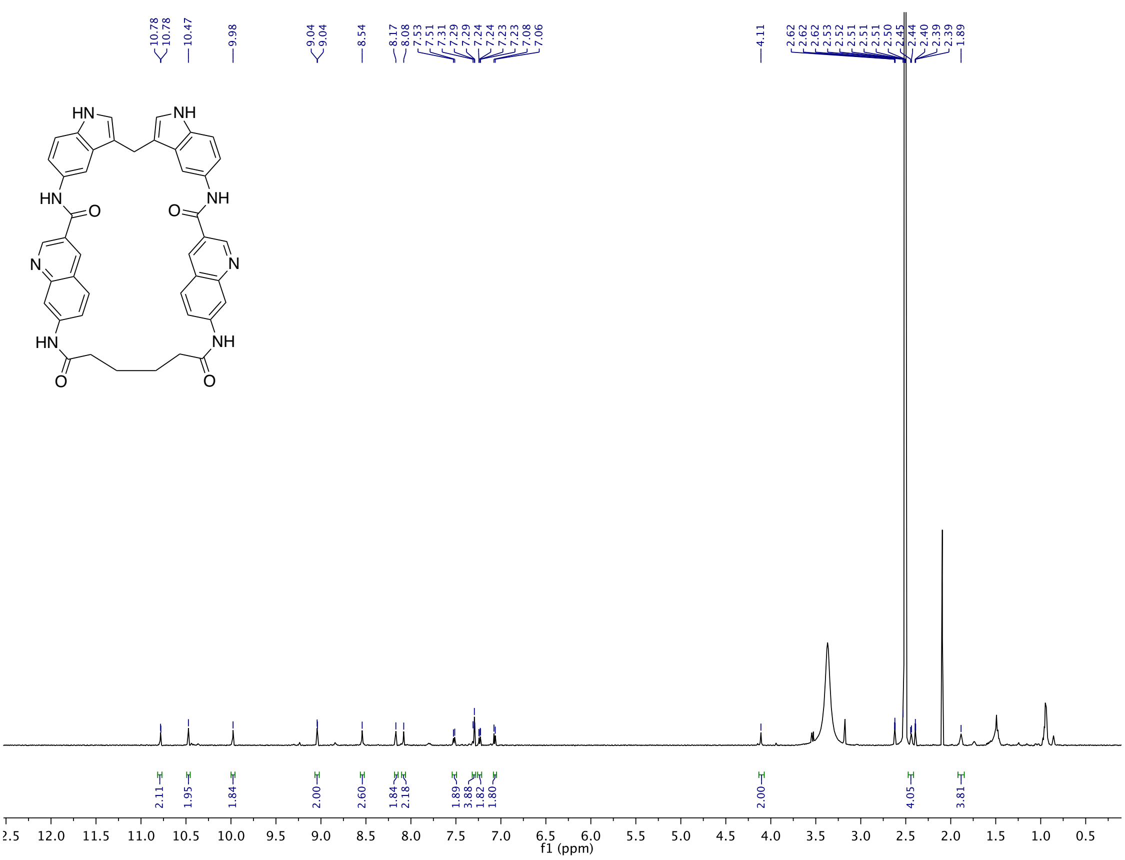

^{1}H NMR spectrum of **13b2** measured in DMSO-d_6 at 600 MHz.

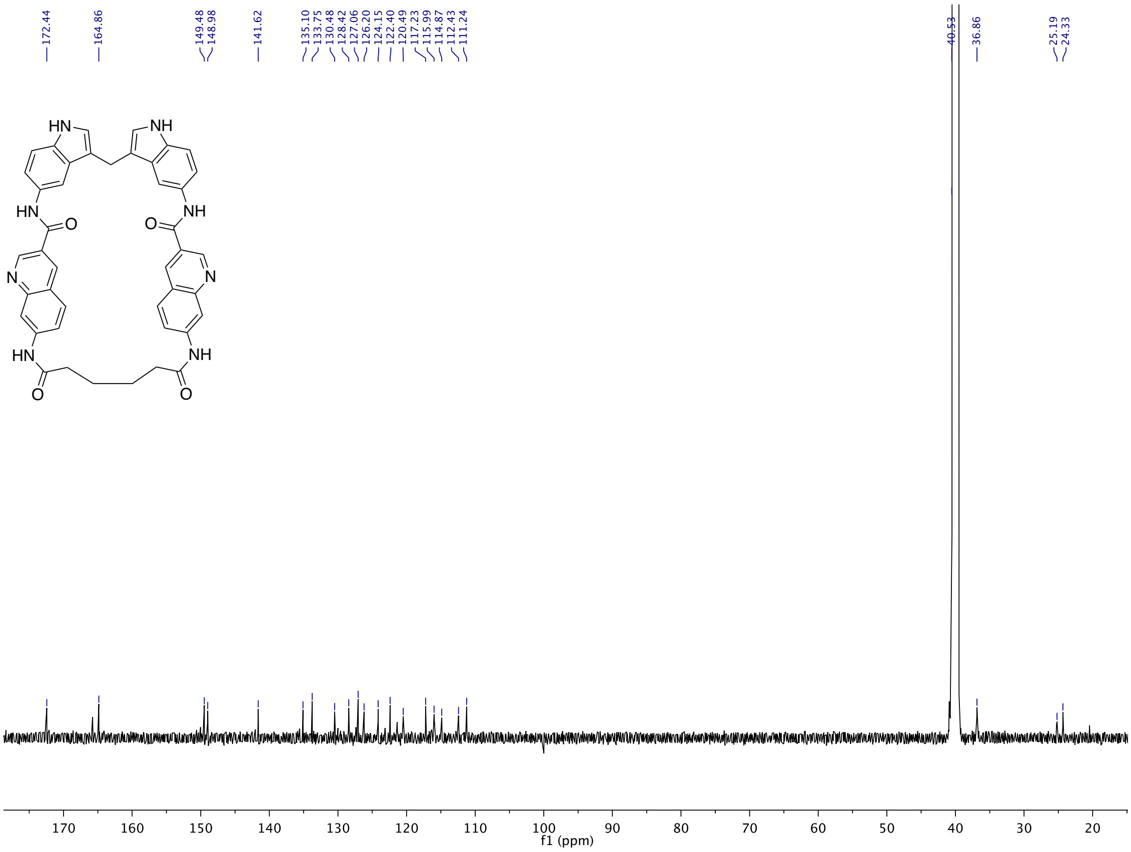

^{13}C NMR spectrum of **13b2** measured in DMSO-d_6 at 150 MHz.

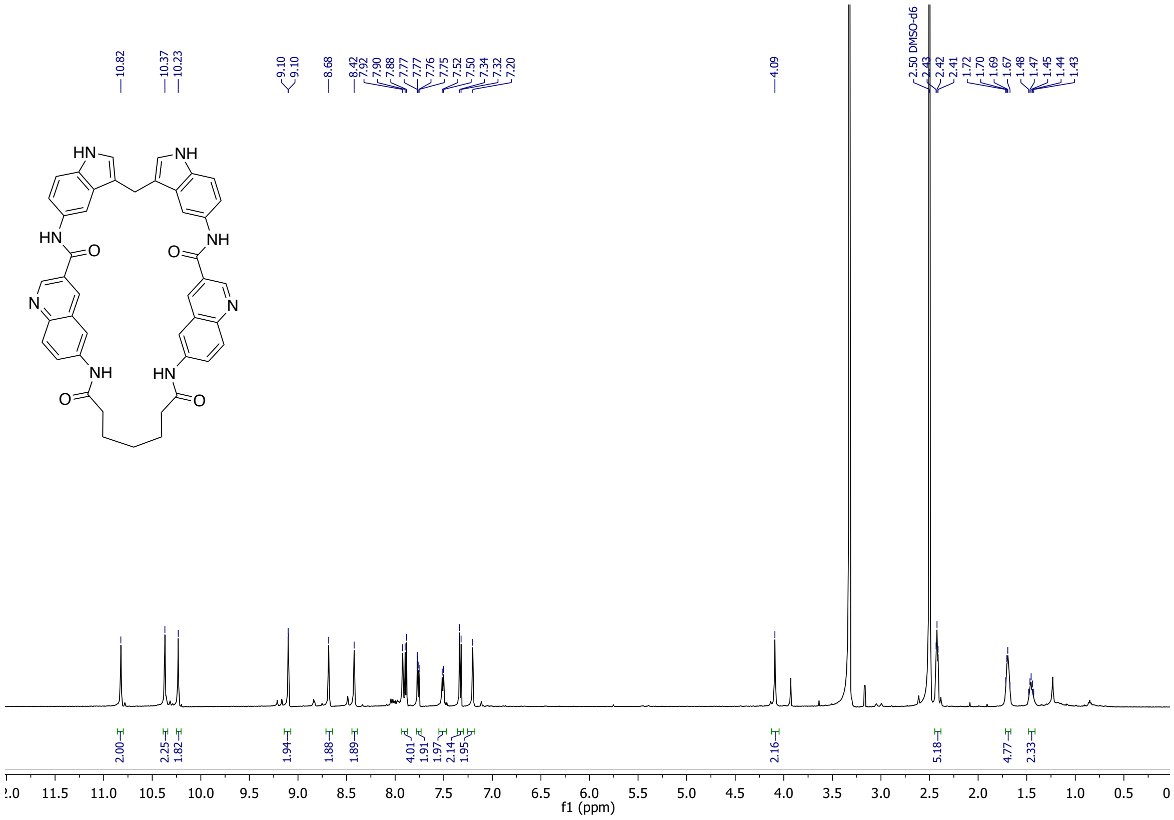

^{1}H NMR spectrum of **13a3** measured in DMSO-d_6 at 600 MHz.

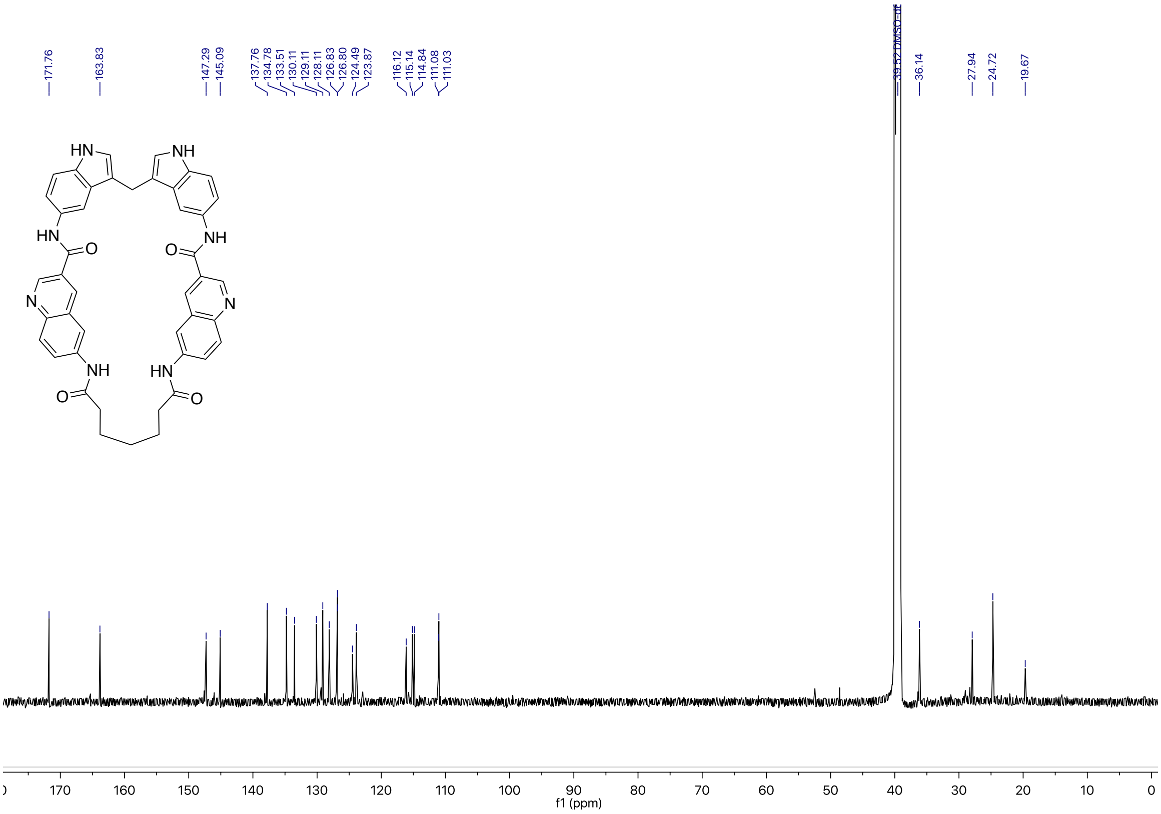

^{13}C NMR spectrum of **13a3** measured in DMSO-d_6 at 151 MHz.

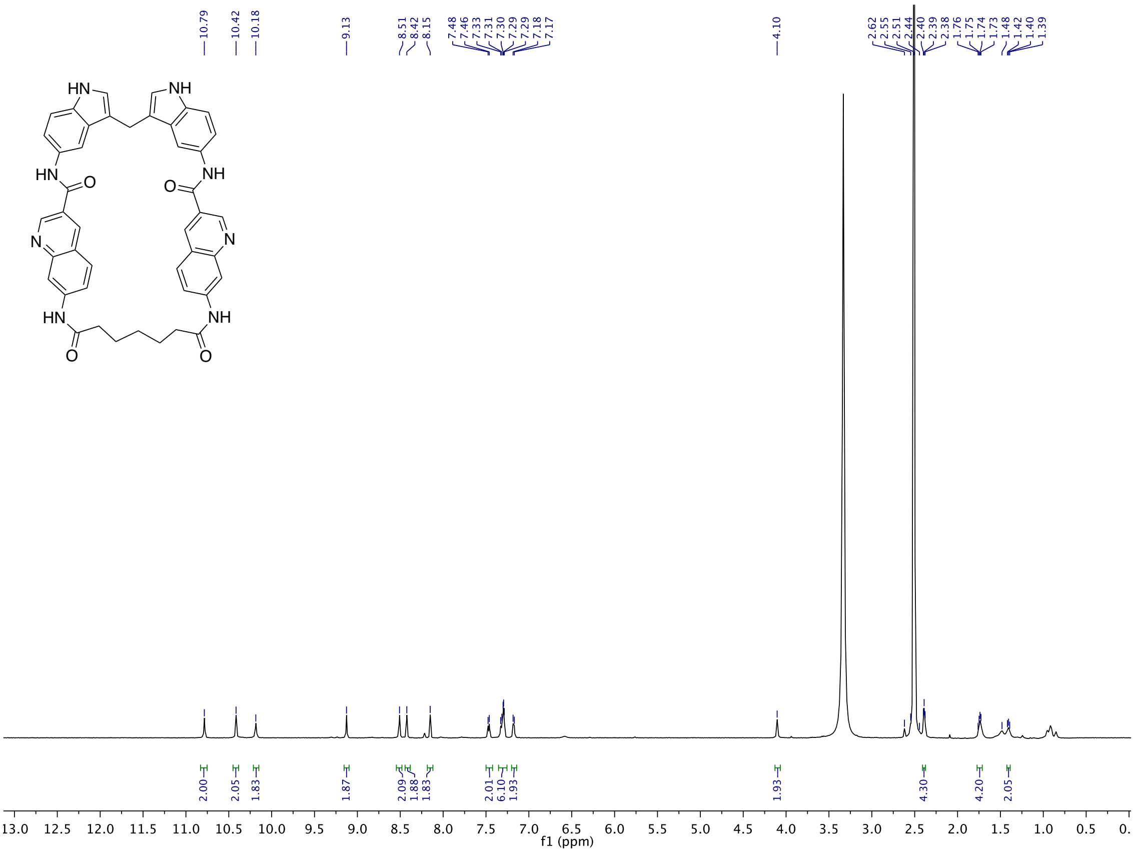

^{1}H NMR spectrum of **13b3** measured in DMSO-d_6 at 600 MHz.

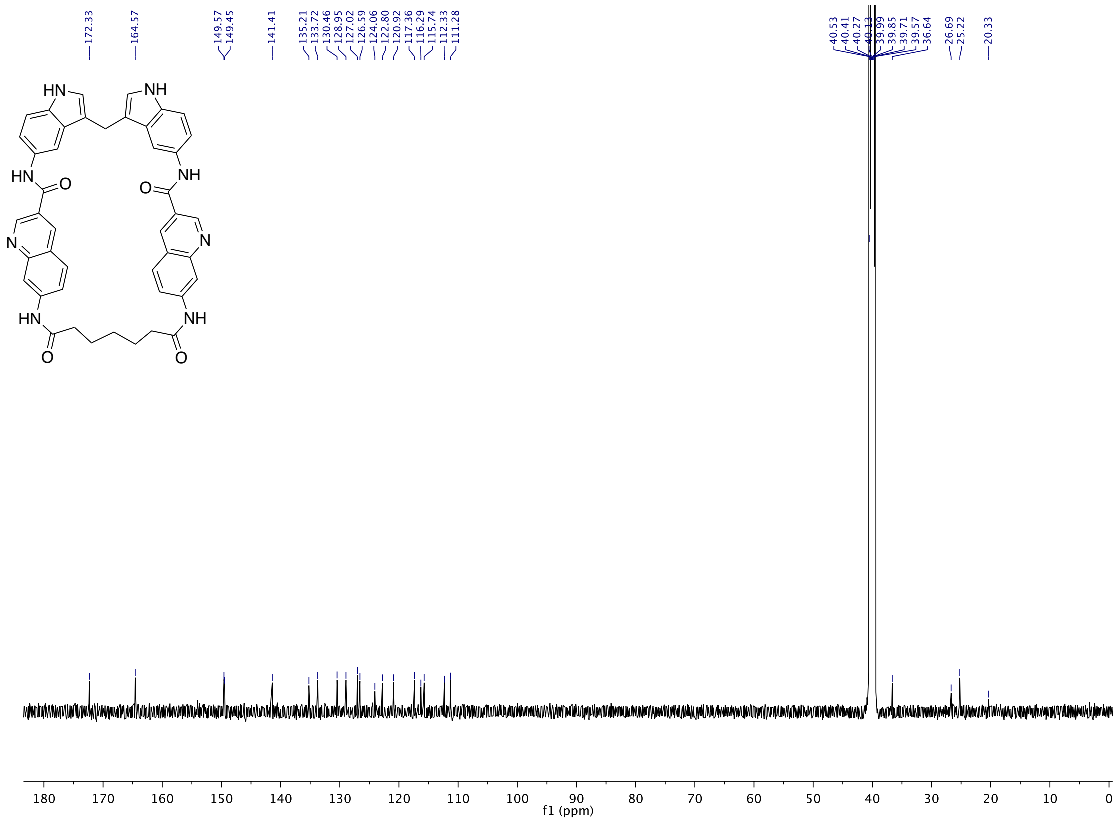

^{13}C NMR spectrum of **13b3** measured in DMSO-d_6 at 151 MHz.

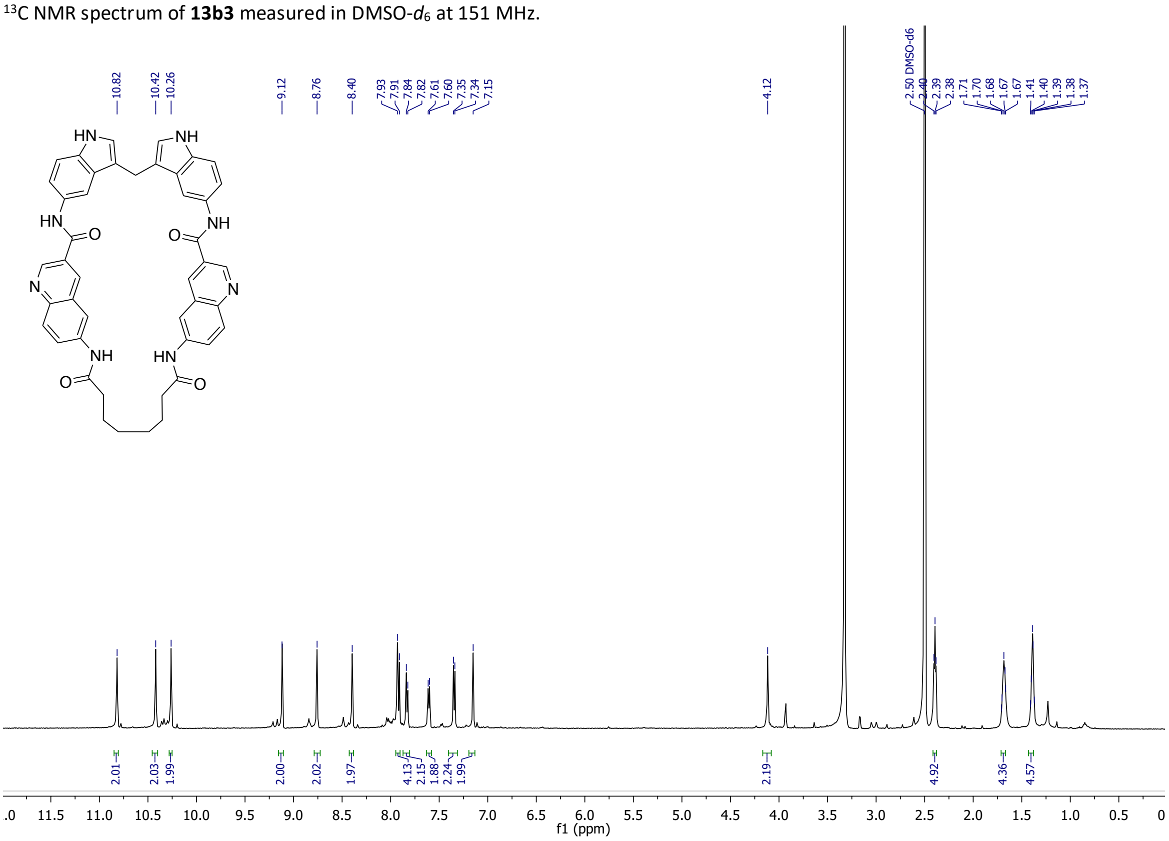

^{1}H NMR spectrum of **13a4** measured in DMSO-d_6 at 600 MHz.

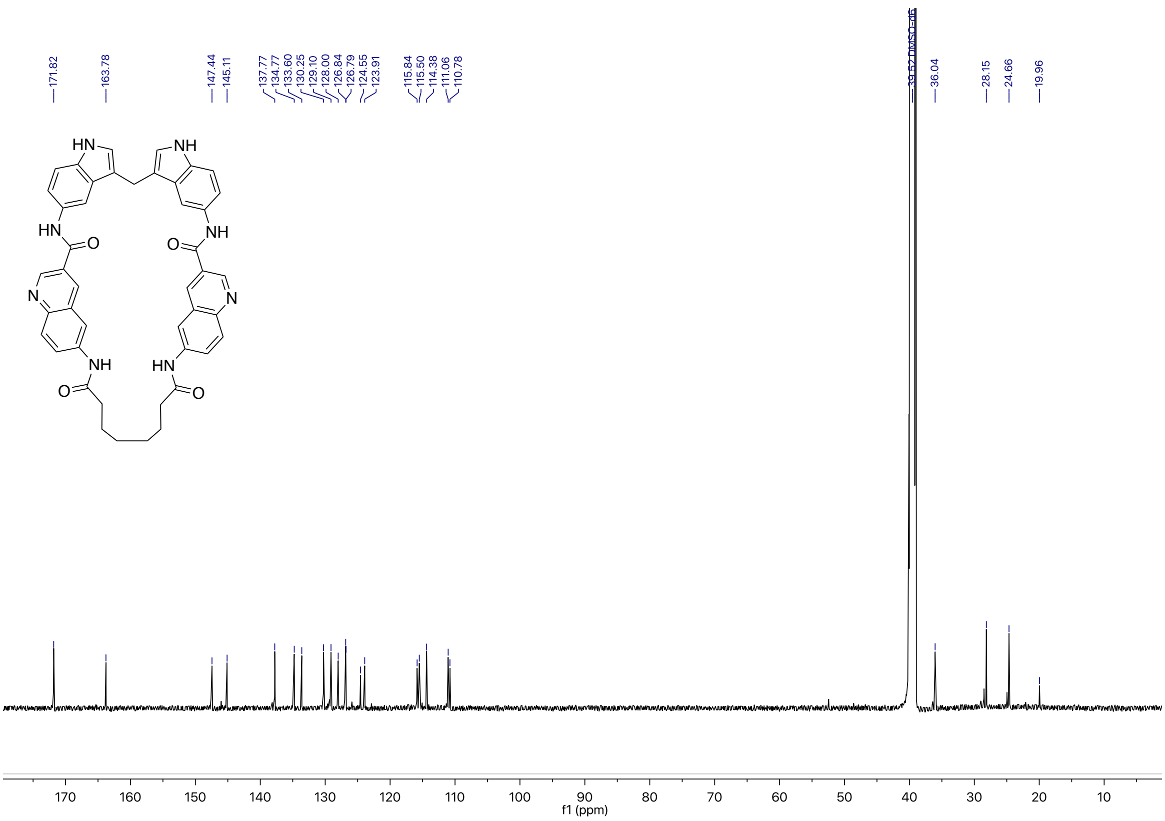

^{13}C NMR spectrum of **13a4** measured in DMSO-d_6 at 151 MHz.

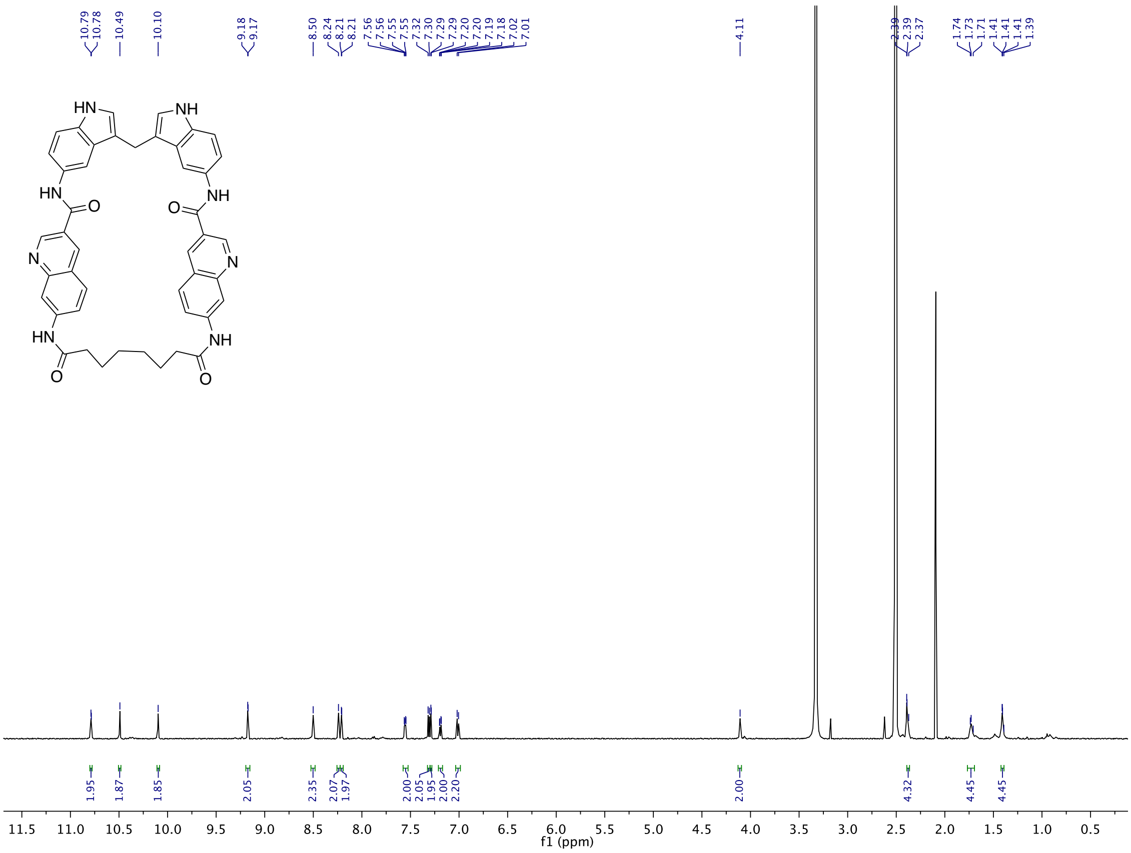

^{1}H NMR spectrum of **13b4** measured in DMSO-d_6 at 600 MHz.

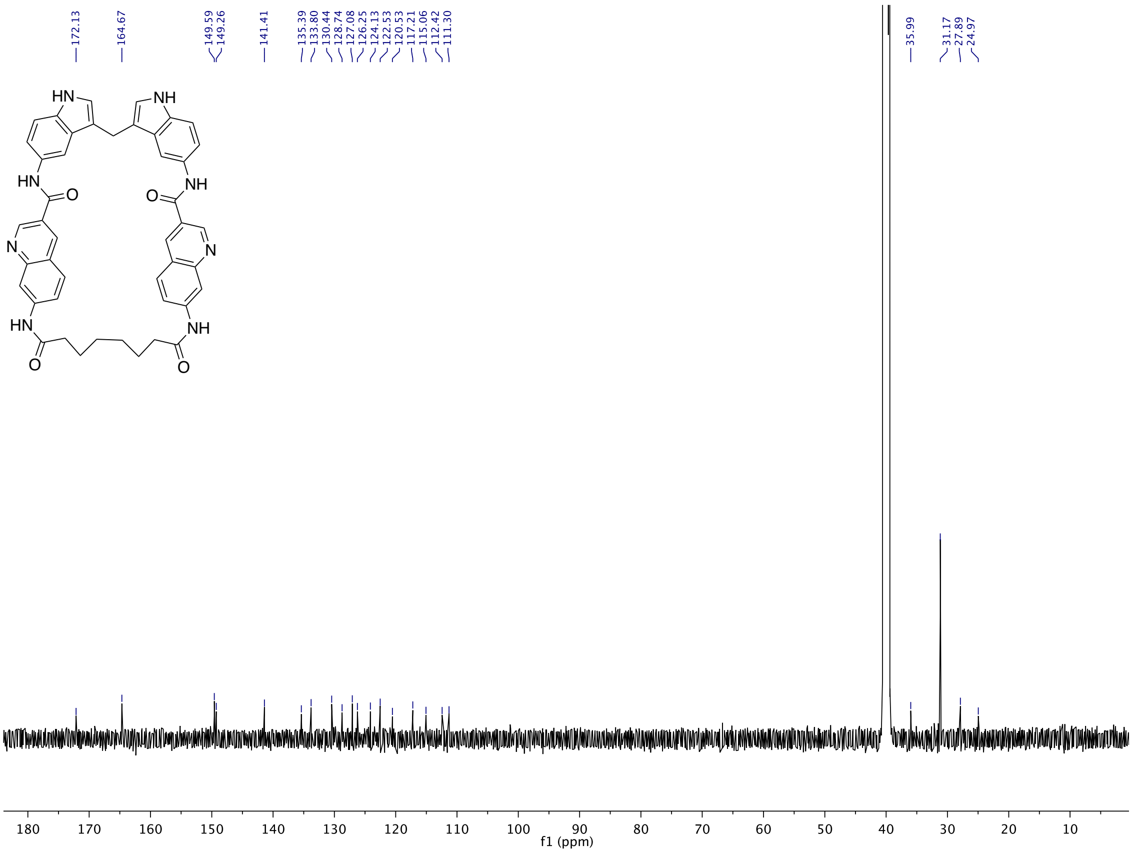

^{13}C NMR spectrum of **13b4** measured in DMSO-d_6 at 151 MHz.

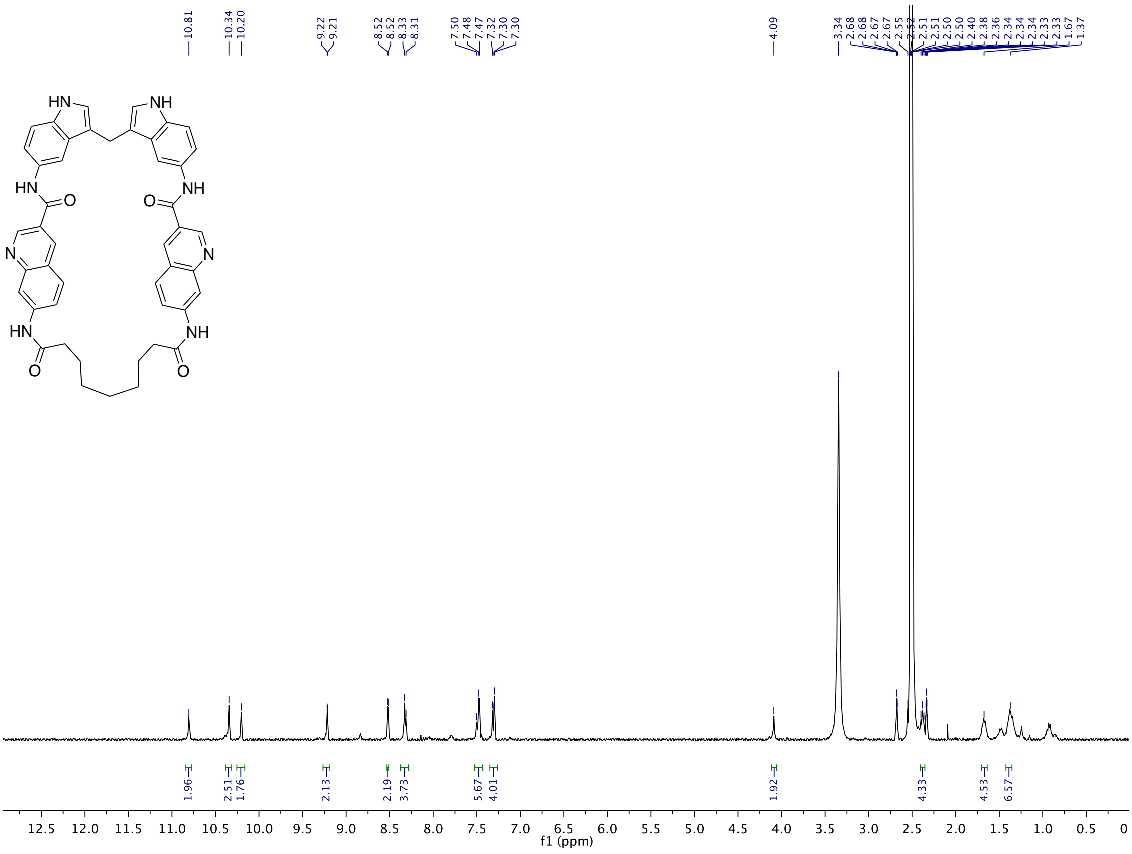

^{1}H NMR spectrum of **13b5** measured in DMSO-d_6 at 400 MHz.

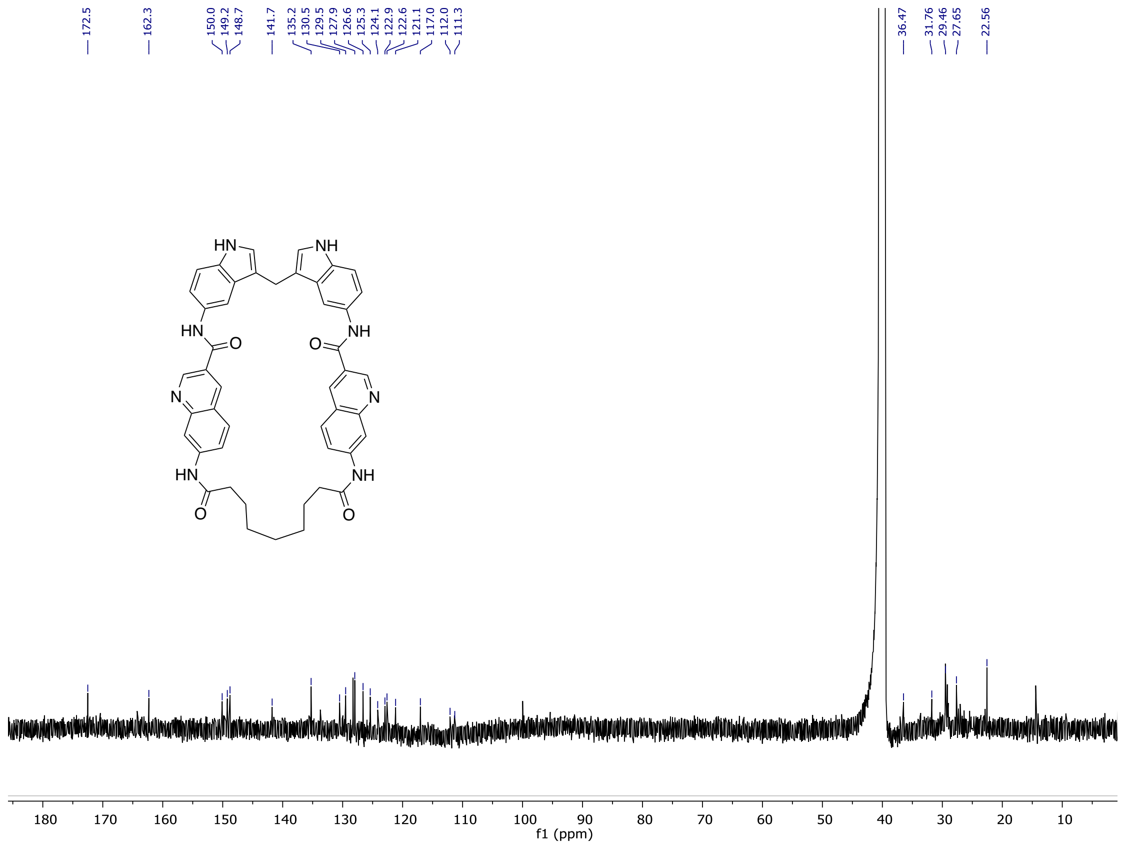

^{13}C NMR spectrum of **13b5** measured in DMSO-d_6 at 151 MHz.

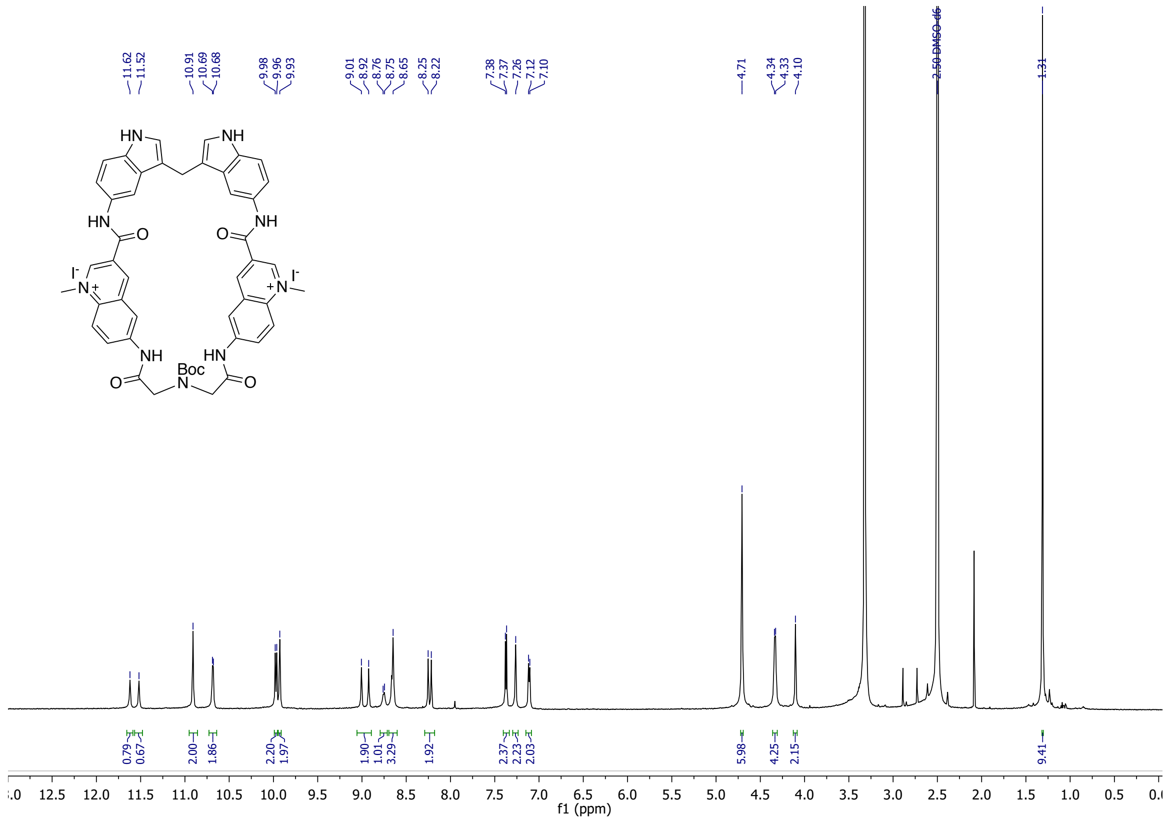

^{1}H NMR spectrum of **14a1** measured in DMSO-d_6 at 600 MHz.

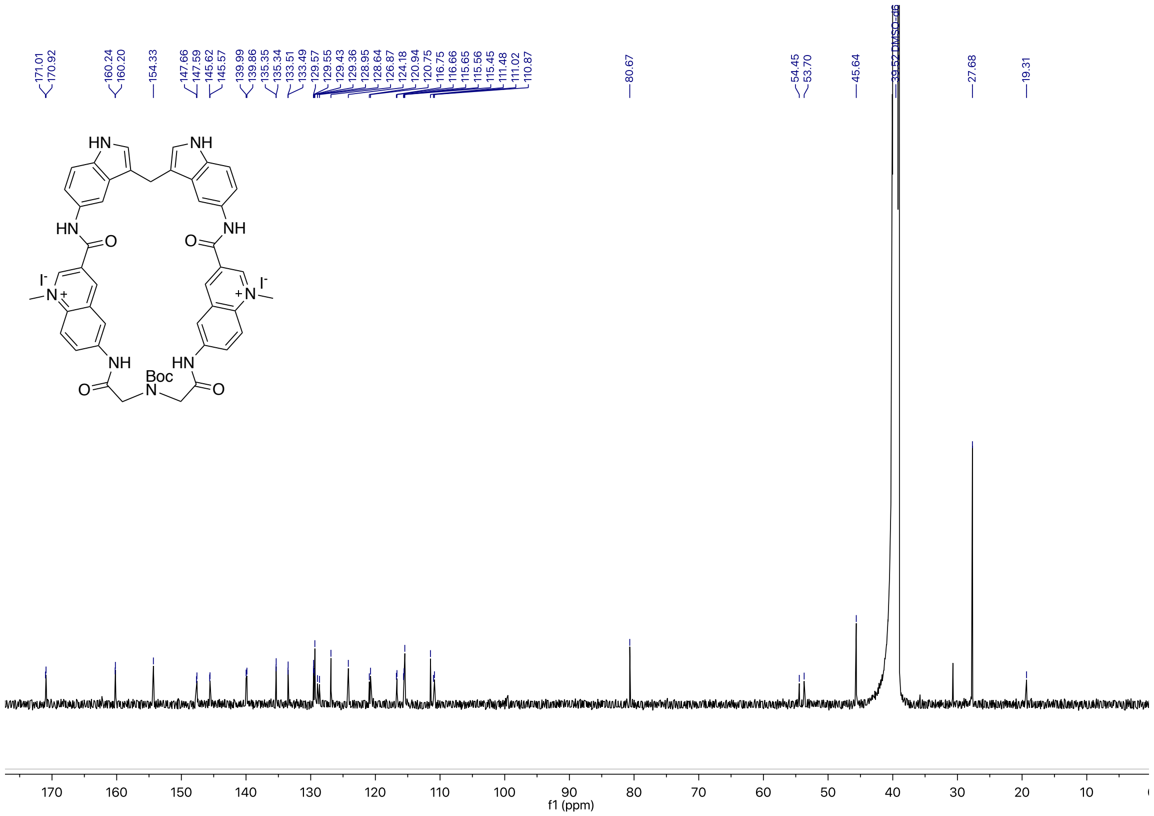

^{13}C NMR spectrum of **14a1** measured in DMSO-d_6 at 151 MHz.

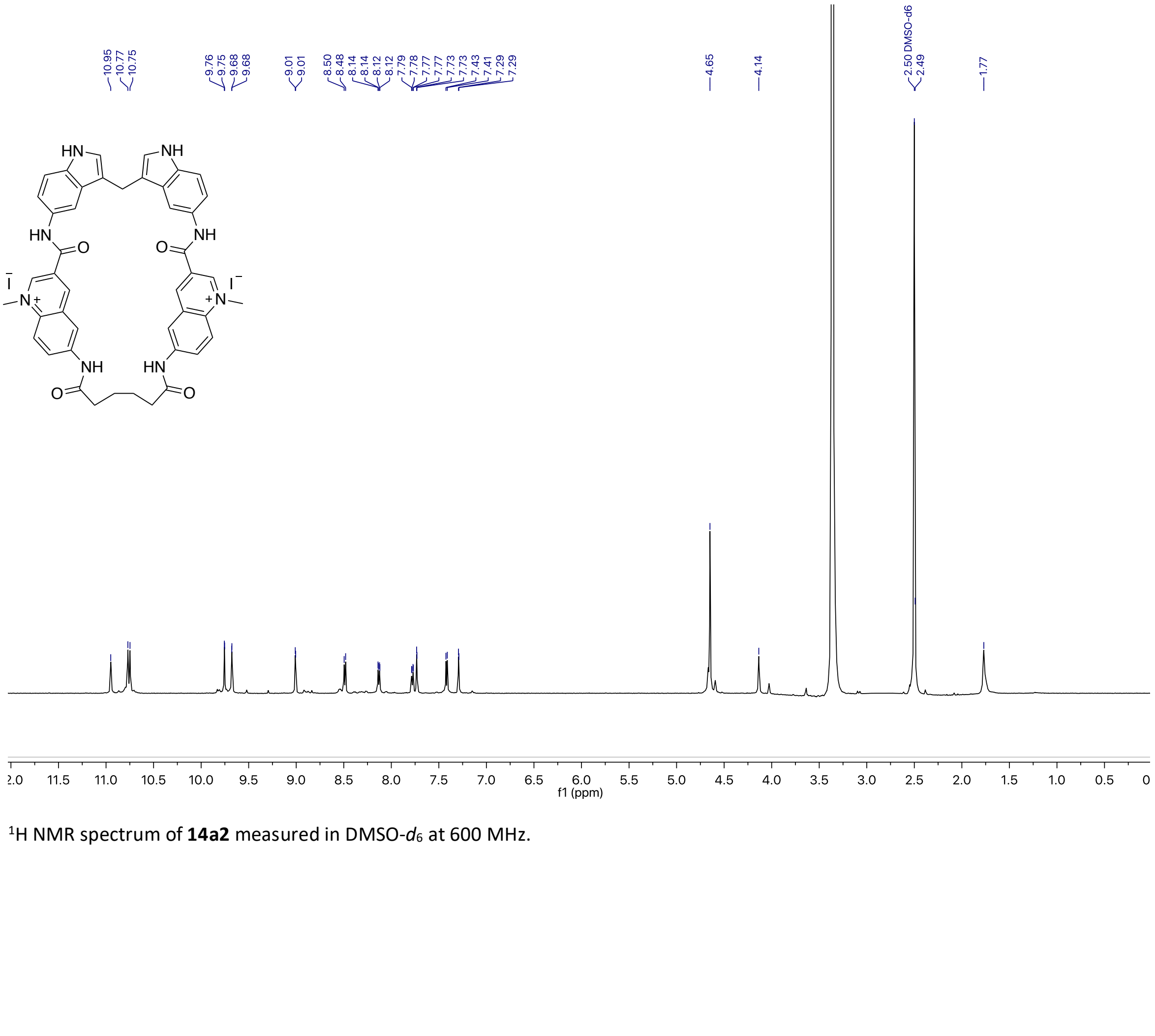

^{1}H NMR spectrum of **14a2** measured in DMSO-d_6 at 600 MHz.

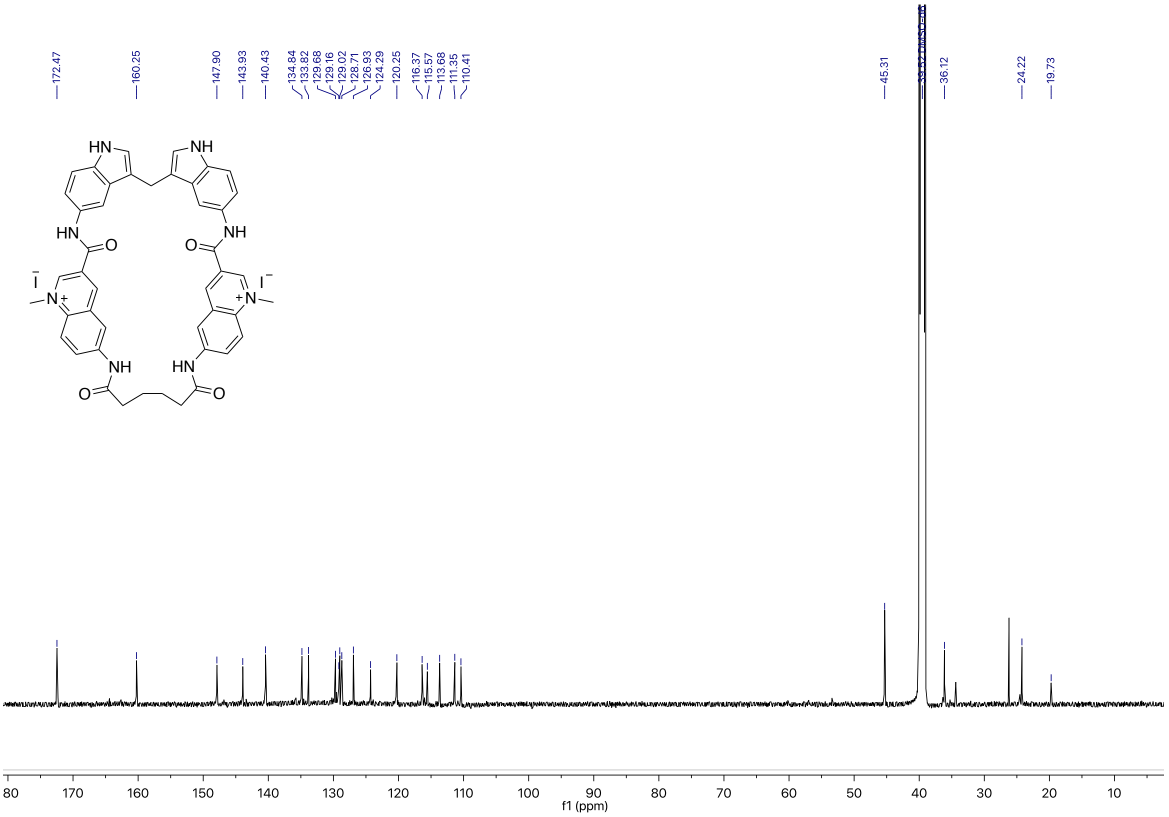

^{13}C NMR spectrum of **14a2** measured in DMSO-d_6 at 151 MHz.

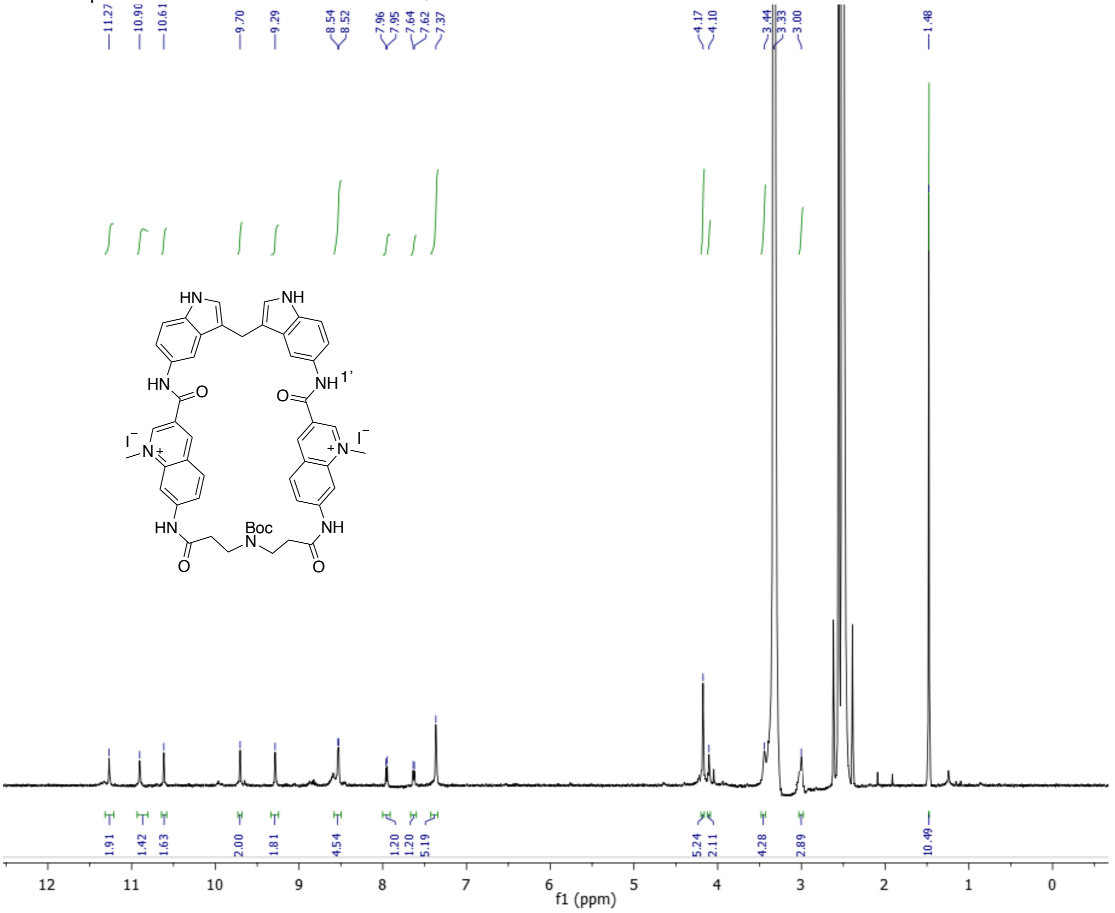

^{1}H NMR spectrum of **14b1** measured in DMSO-d_6 at 600 MHz.

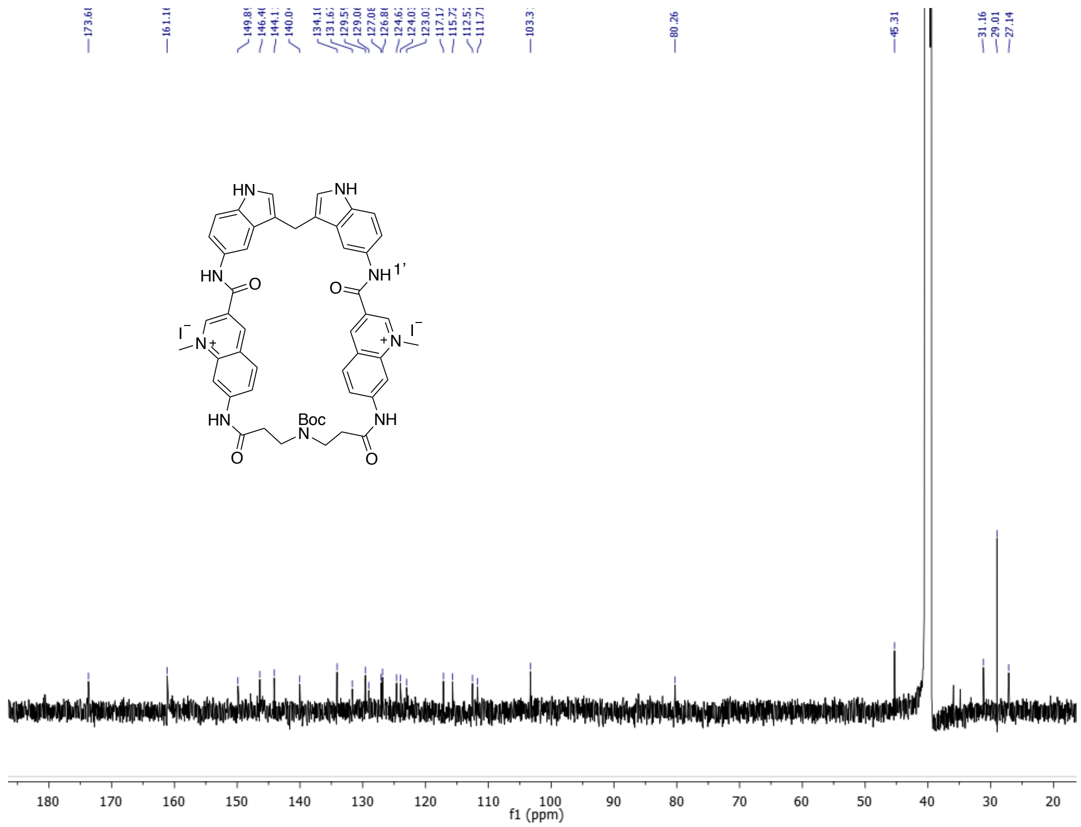

^{13}C NMR spectrum of **14b1** measured in DMSO-d_6 at 151 MHz.

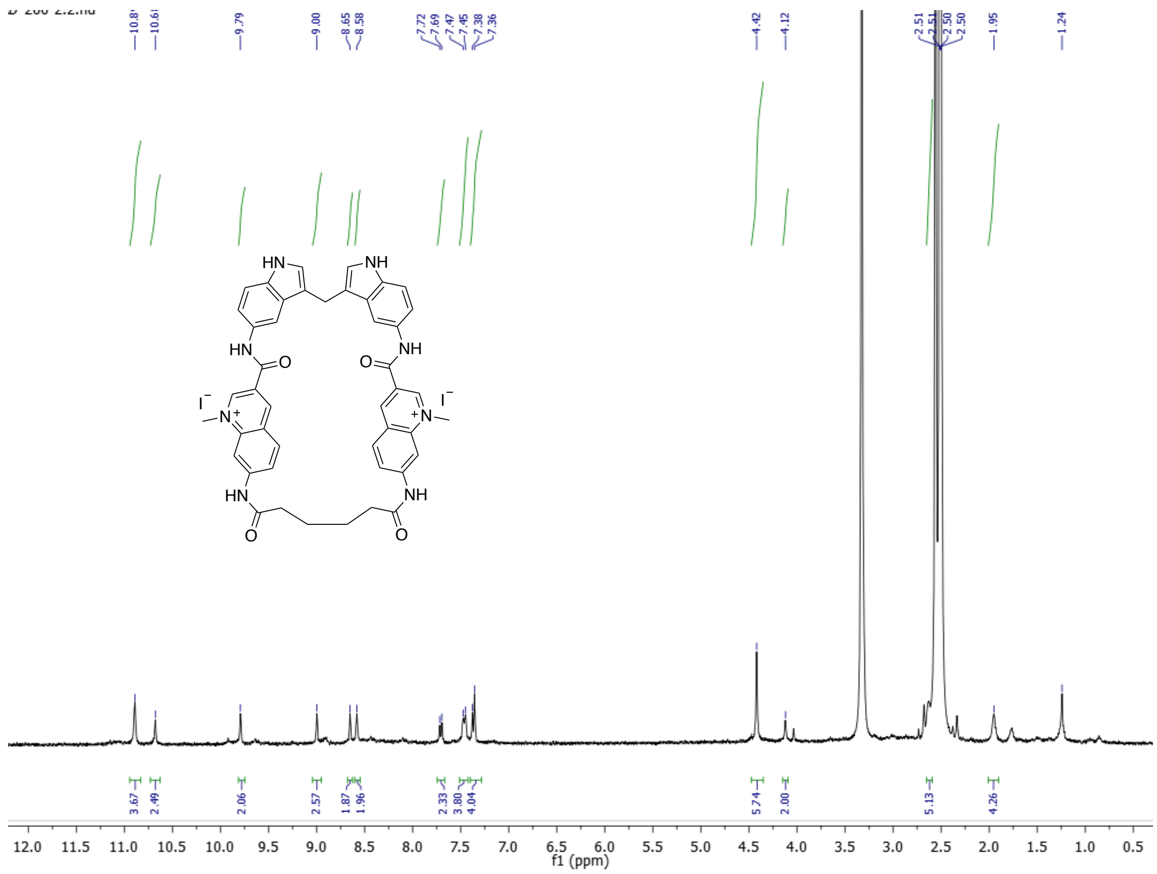

^{1}H NMR spectrum of **14b2** measured in DMSO-d_6 at 600 MHz.

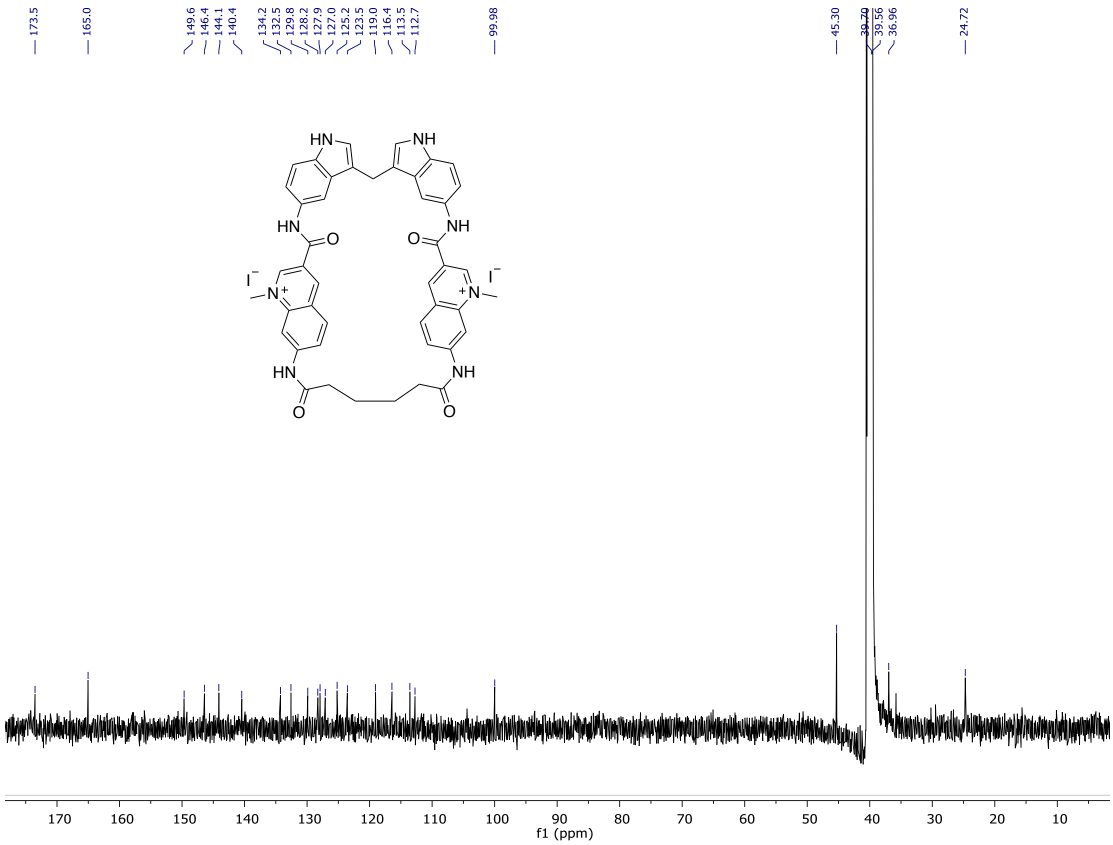

^{13}C NMR spectrum of **14b2** measured in DMSO-d_6 at 151 MHz.

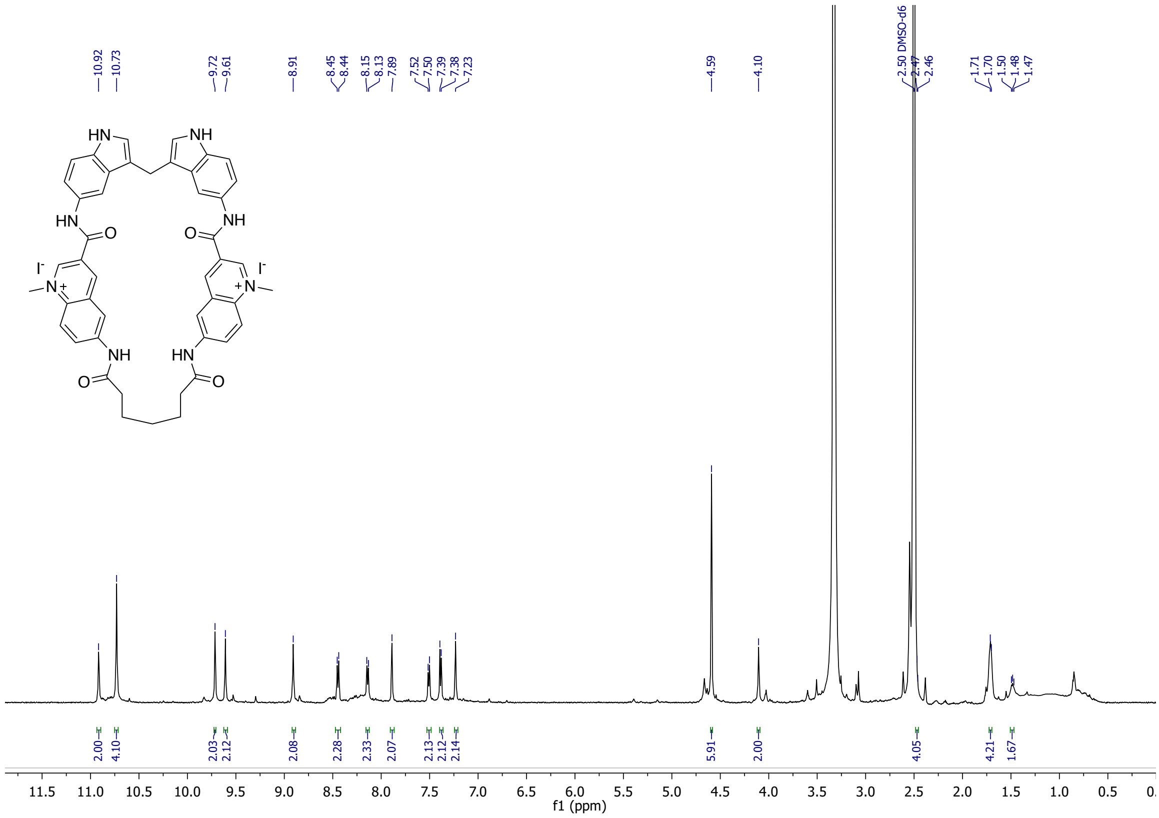

^{1}H NMR spectrum of **14a3** measured in DMSO-d_6 at 600 MHz.

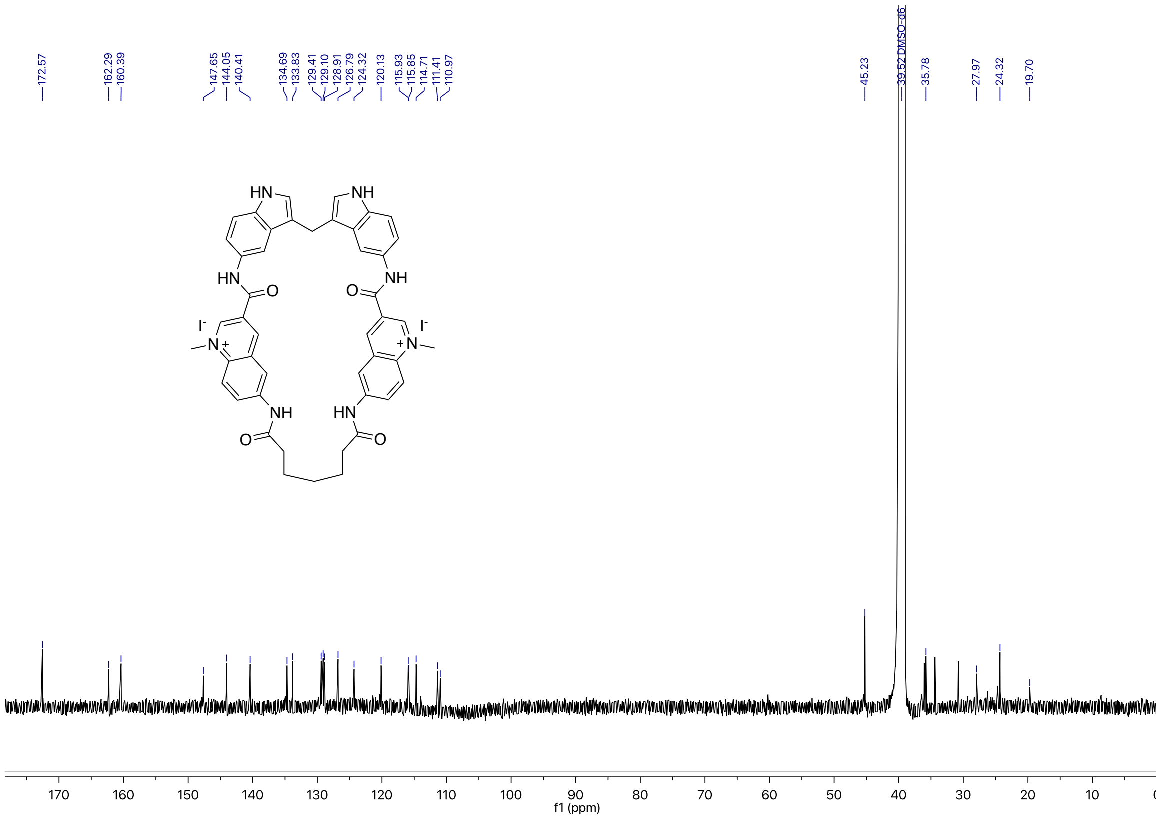

^{13}C NMR spectrum of **14a3** measured in DMSO-d_6 at 151 MHz.

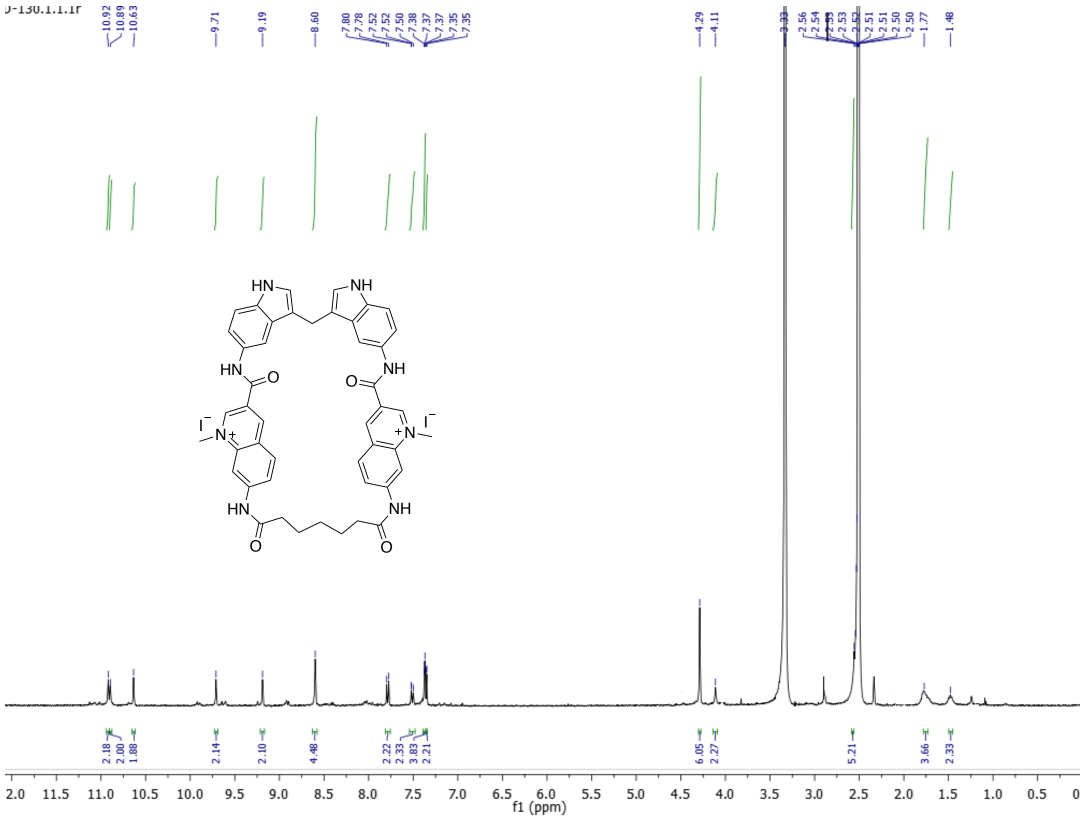

^{1}H NMR spectrum of **14b3** measured in DMSO-d_6 at 600 MHz.

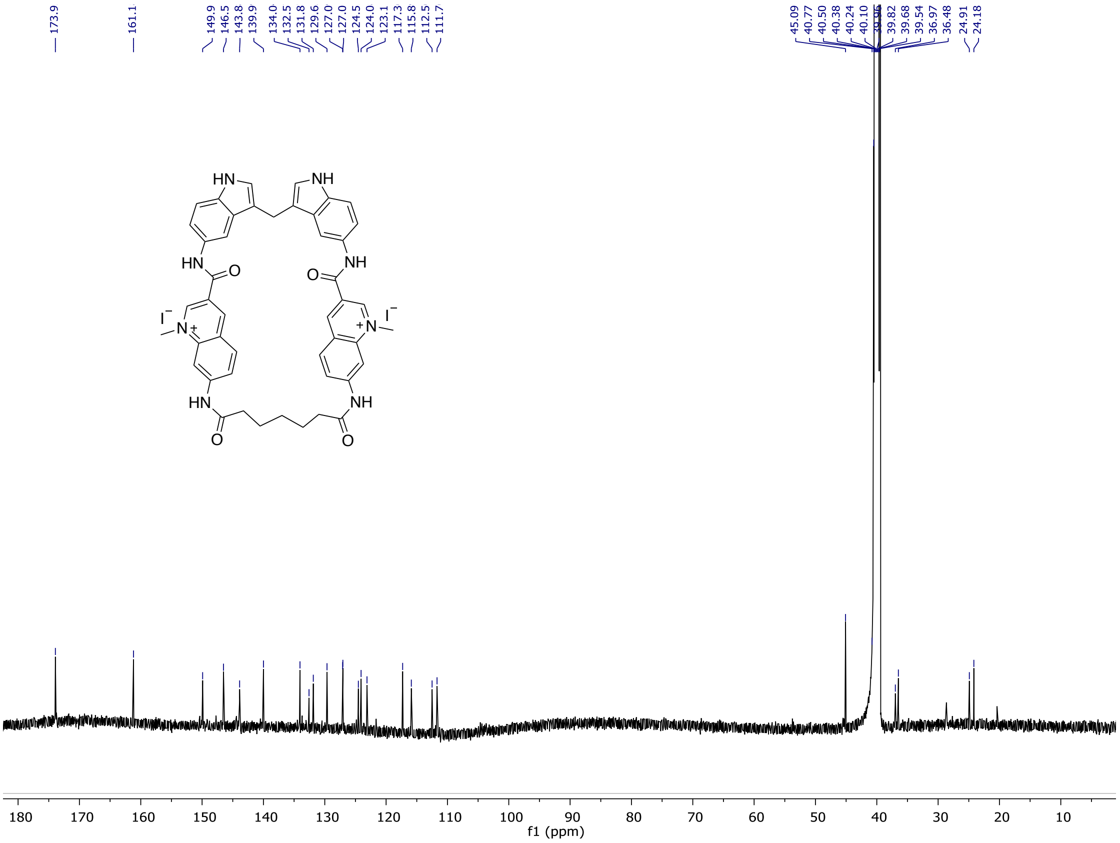

^{13}C NMR spectrum of **14b3** measured in DMSO-d_6 at 151 MHz.

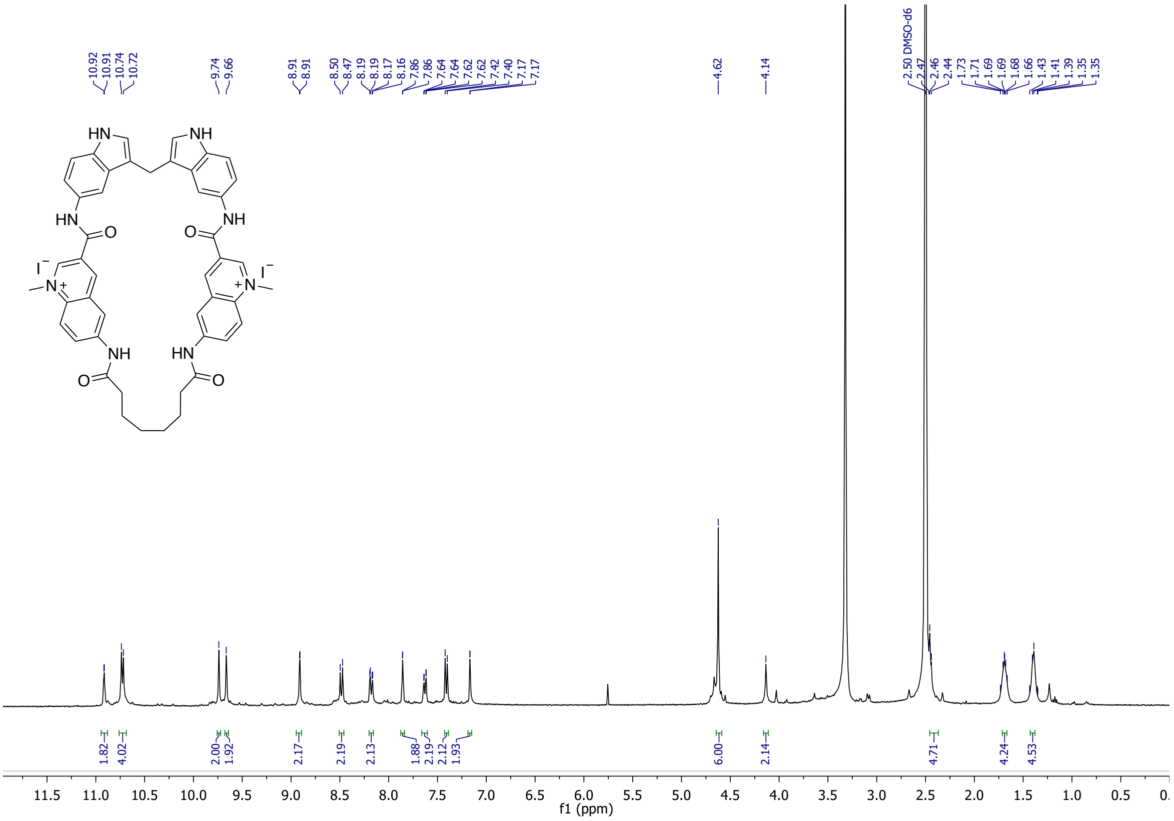

^{1}H NMR spectrum of **14a4** measured in DMSO-d_6 at 600 MHz.

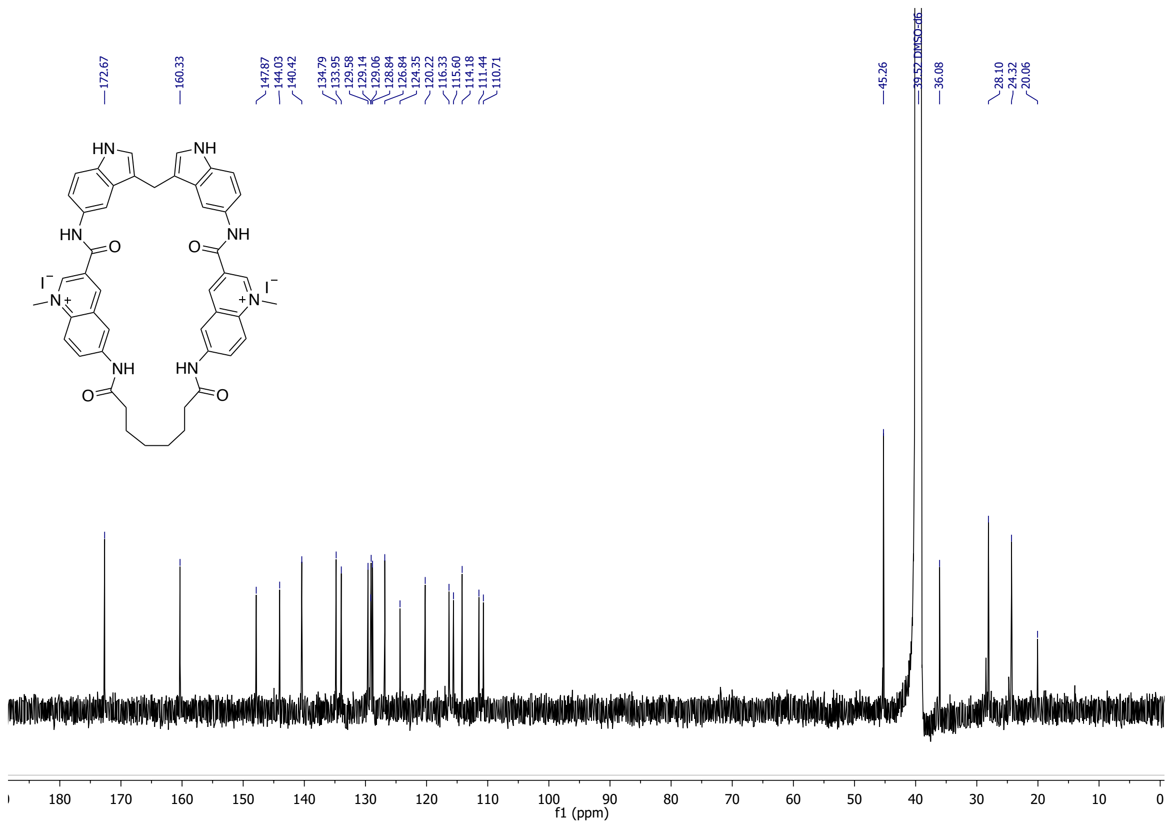

^{13}C NMR spectrum of **14a4** measured in DMSO-d_6 at 151 MHz.

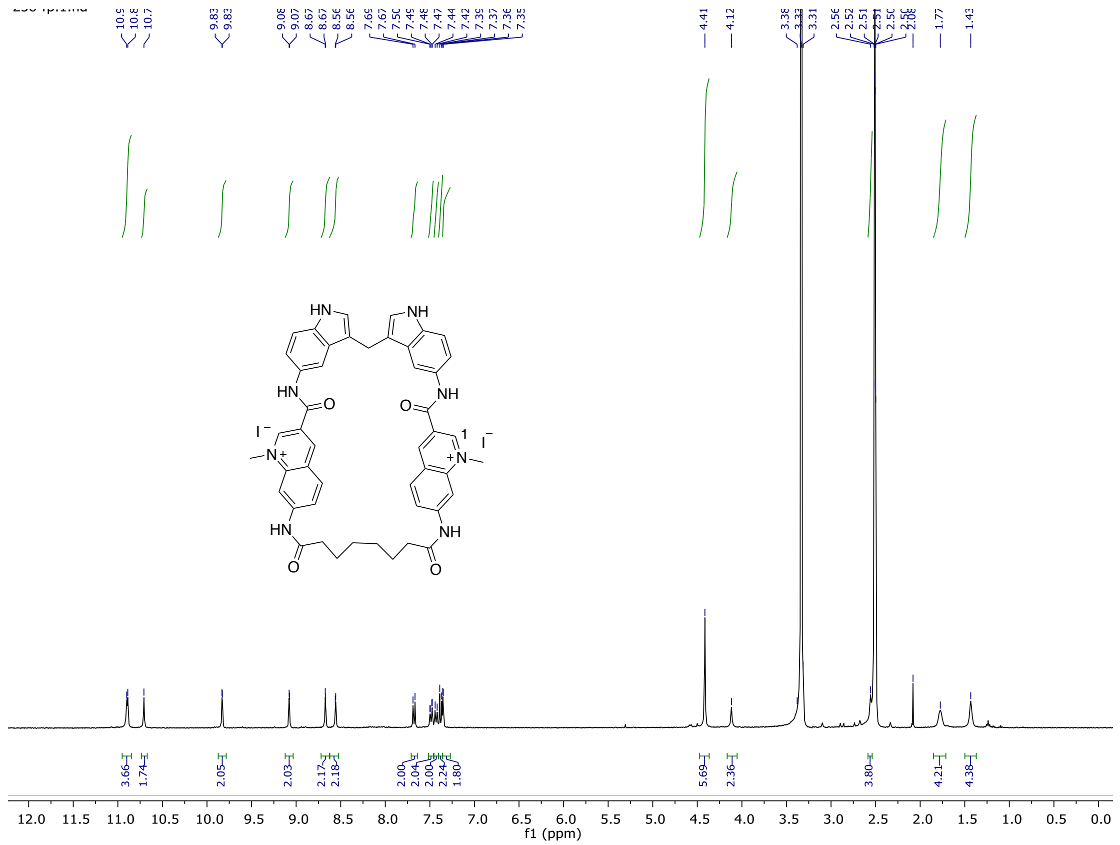

^{1}H NMR spectrum of **14b4** measured in DMSO-d_6 at 600 MHz.

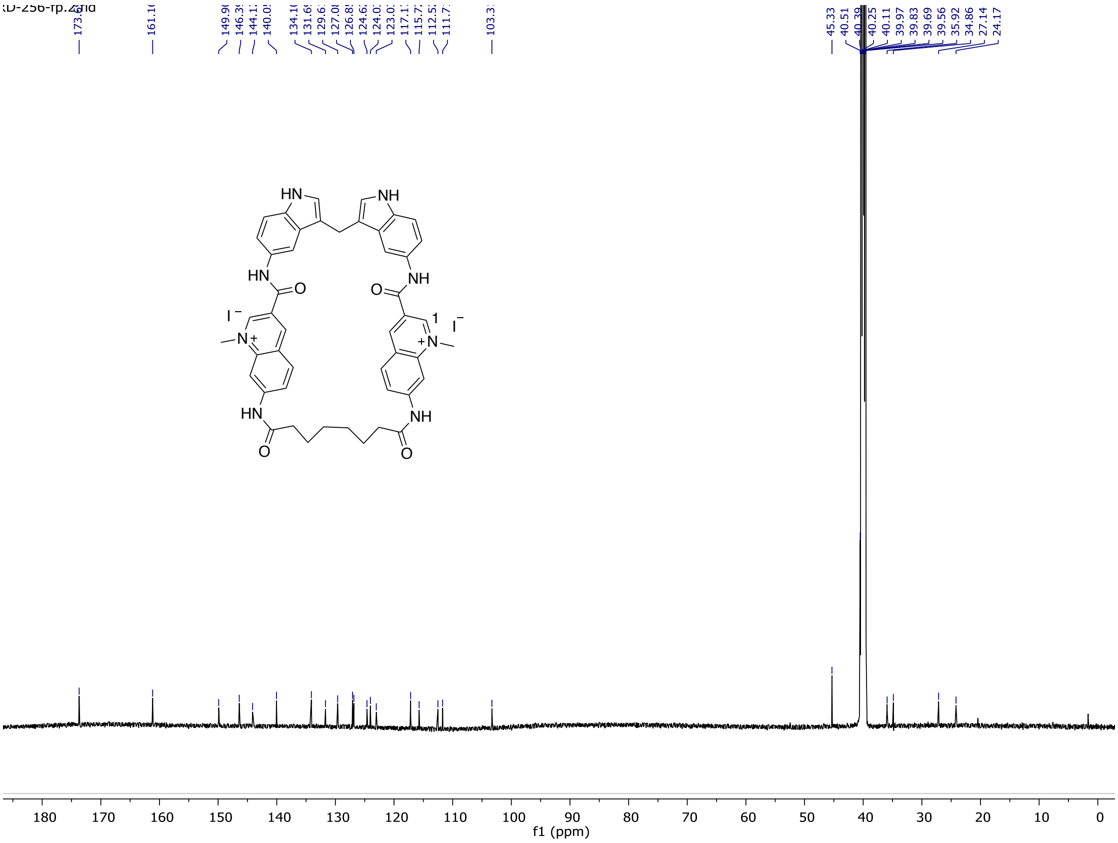

^{13}C NMR spectrum of **14b4** measured in DMSO-d_6 at 151 MHz.

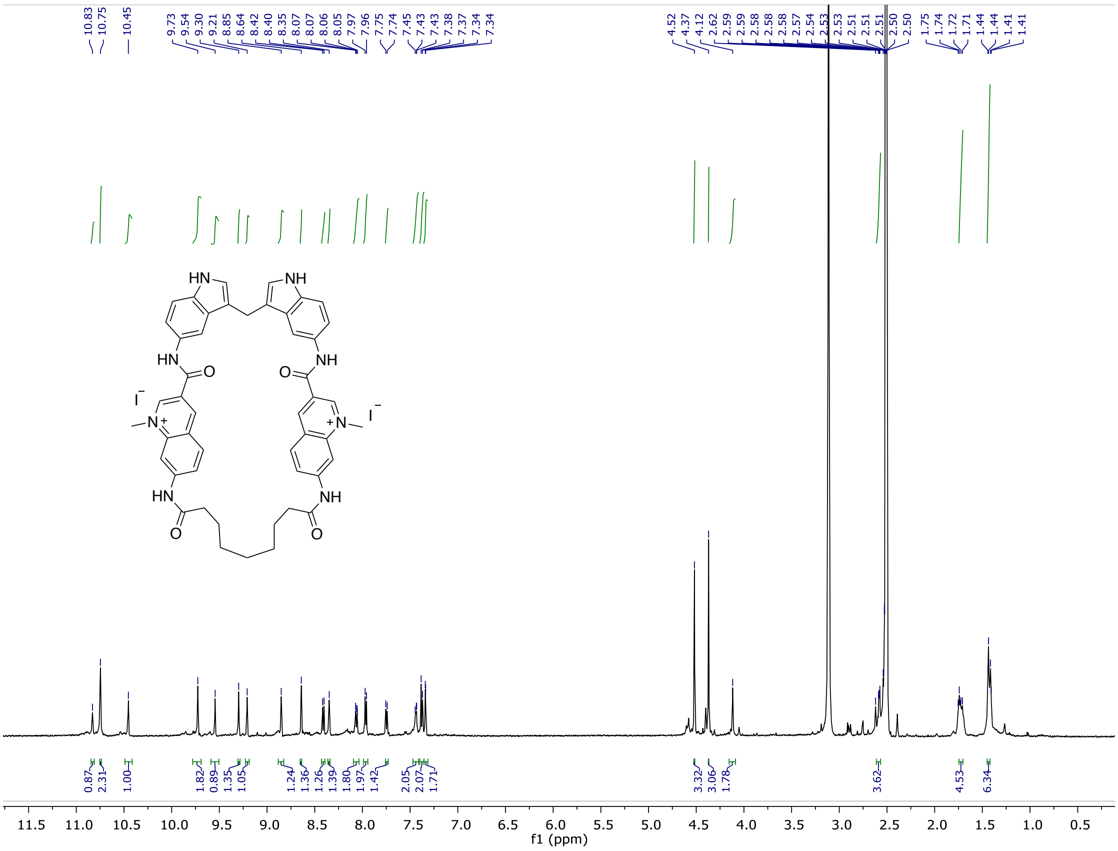

^{1}H NMR spectrum of **14b5** measured in DMSO-d_6 at 600 MHz.

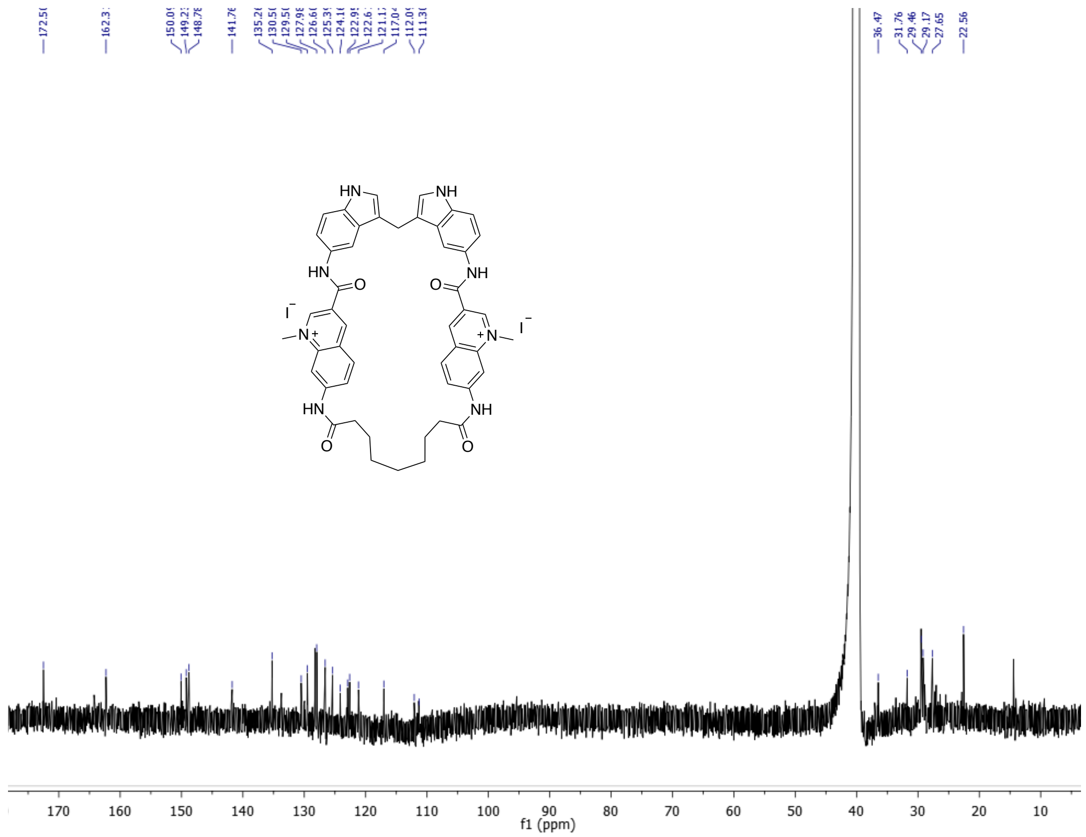

^{13}C NMR spectrum of **14b5** measured in DMSO-d_6 at 151 MHz.

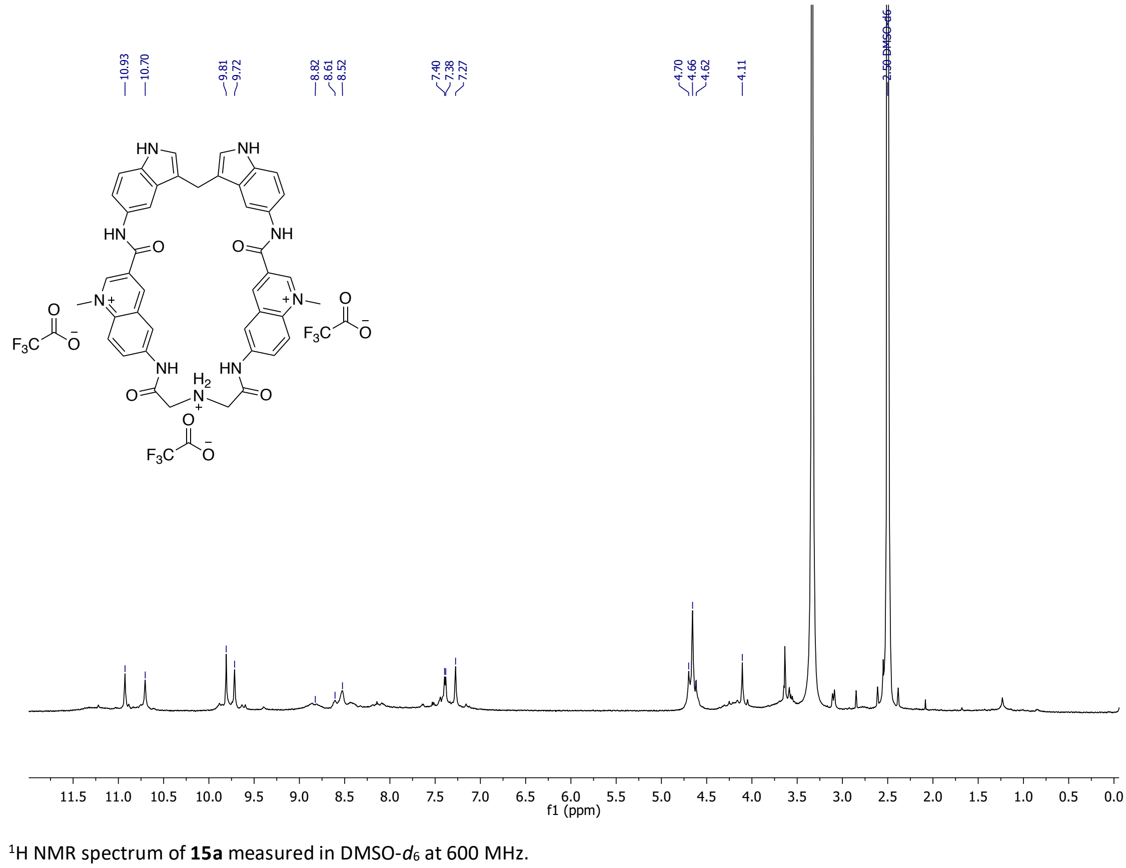

^{1}H NMR spectrum of **15a** measured in DMSO-d_6 at 600 MHz.

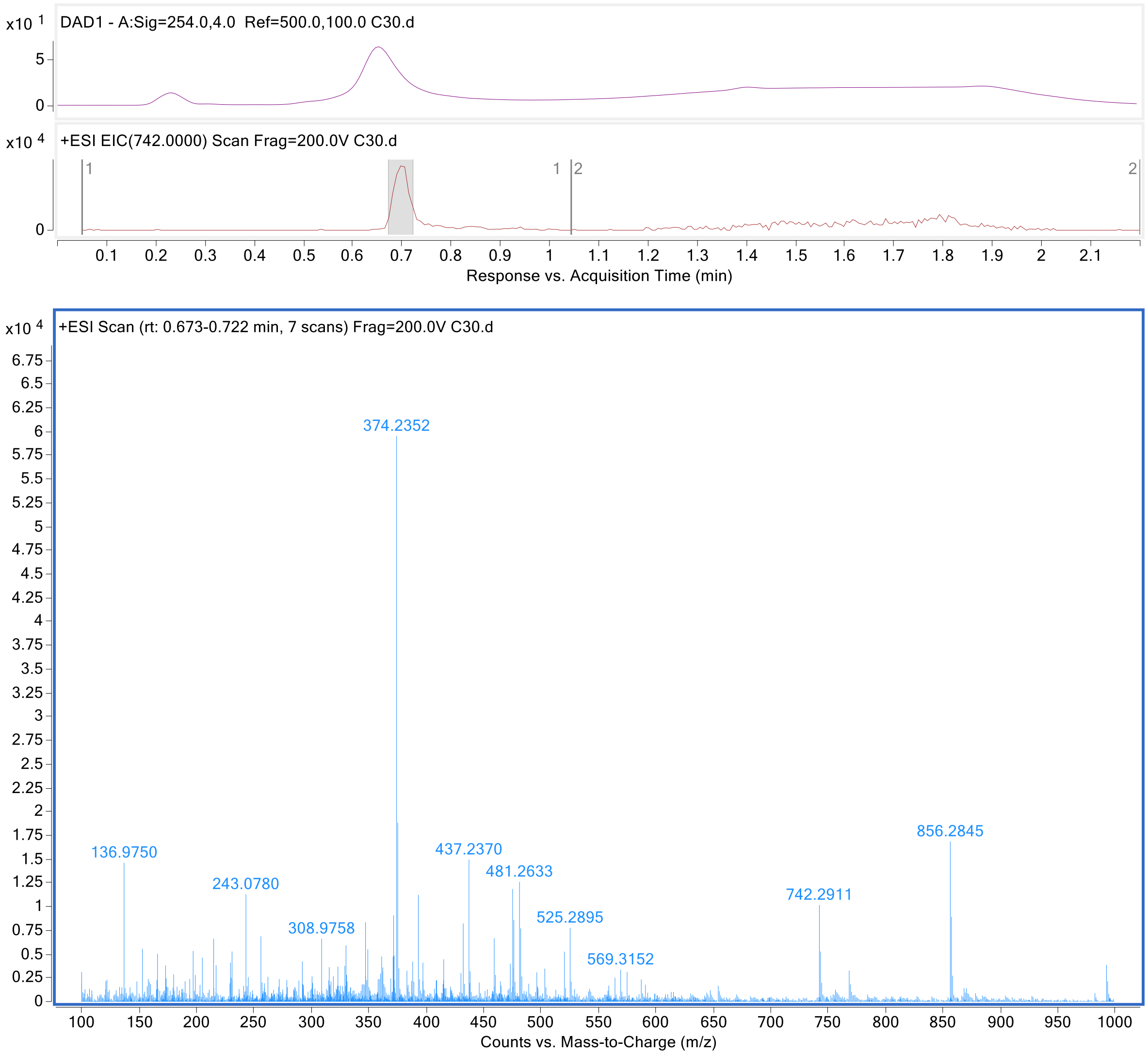

HRMS spectrum of **15a**

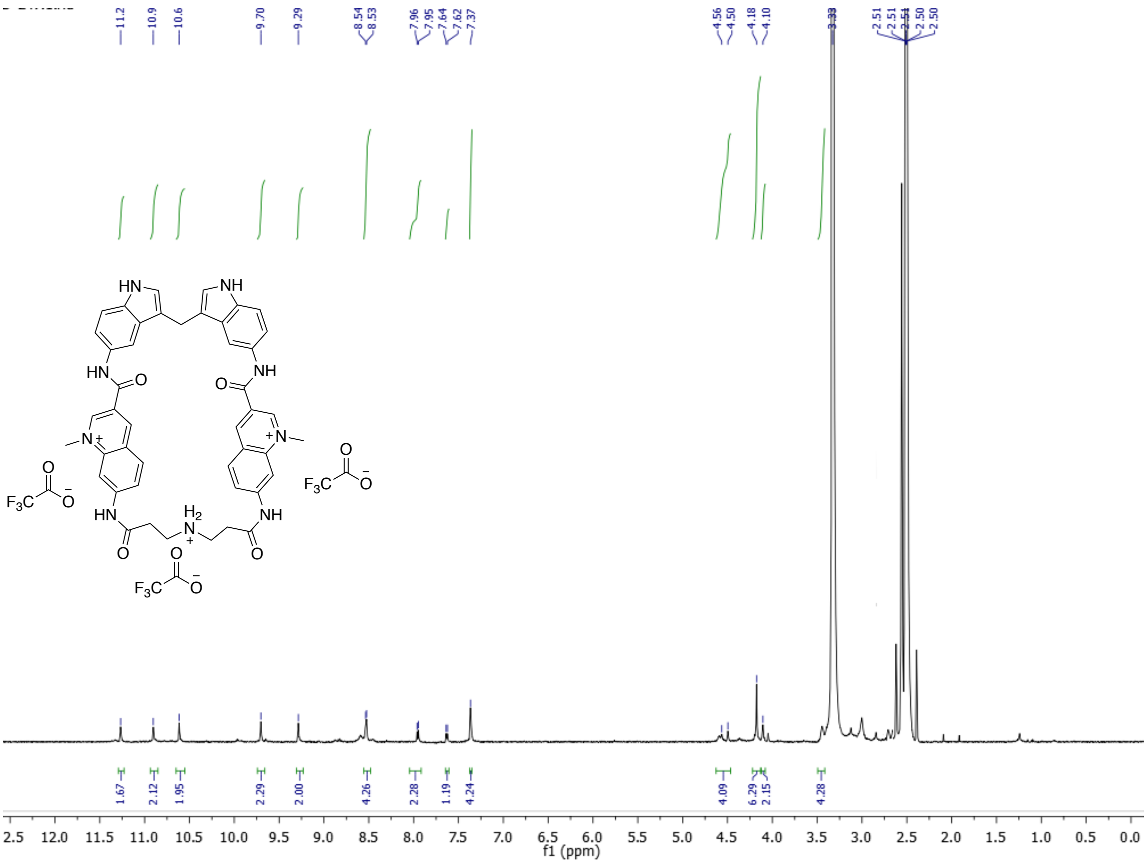

^{1}H NMR spectrum of **15b** measured in DMSO-d_6 at 600 MHz.

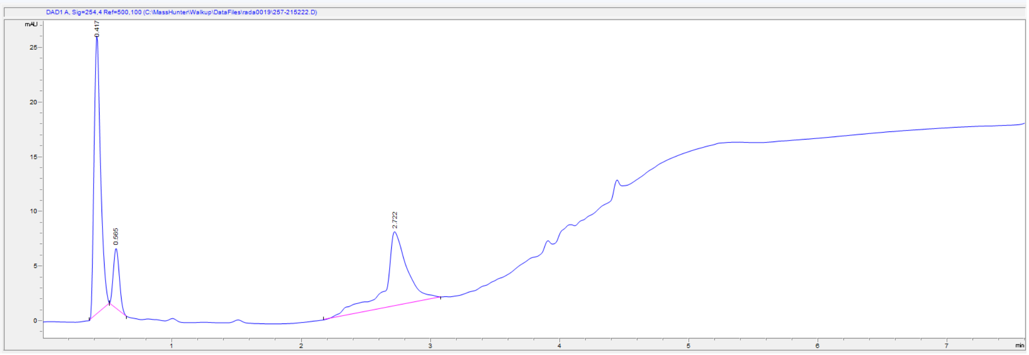

LC-MS spectra of **15b**

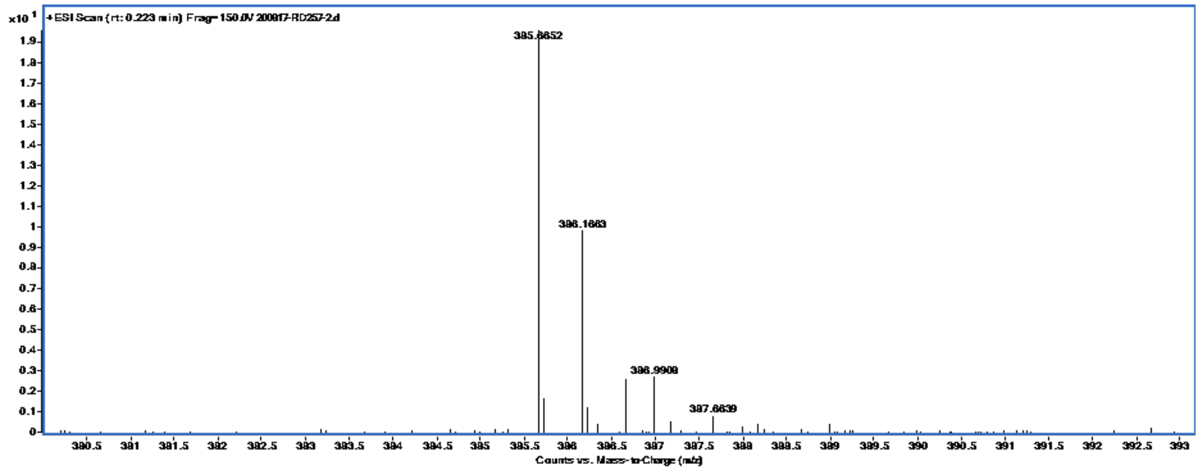

HRMS spectra of **15b**

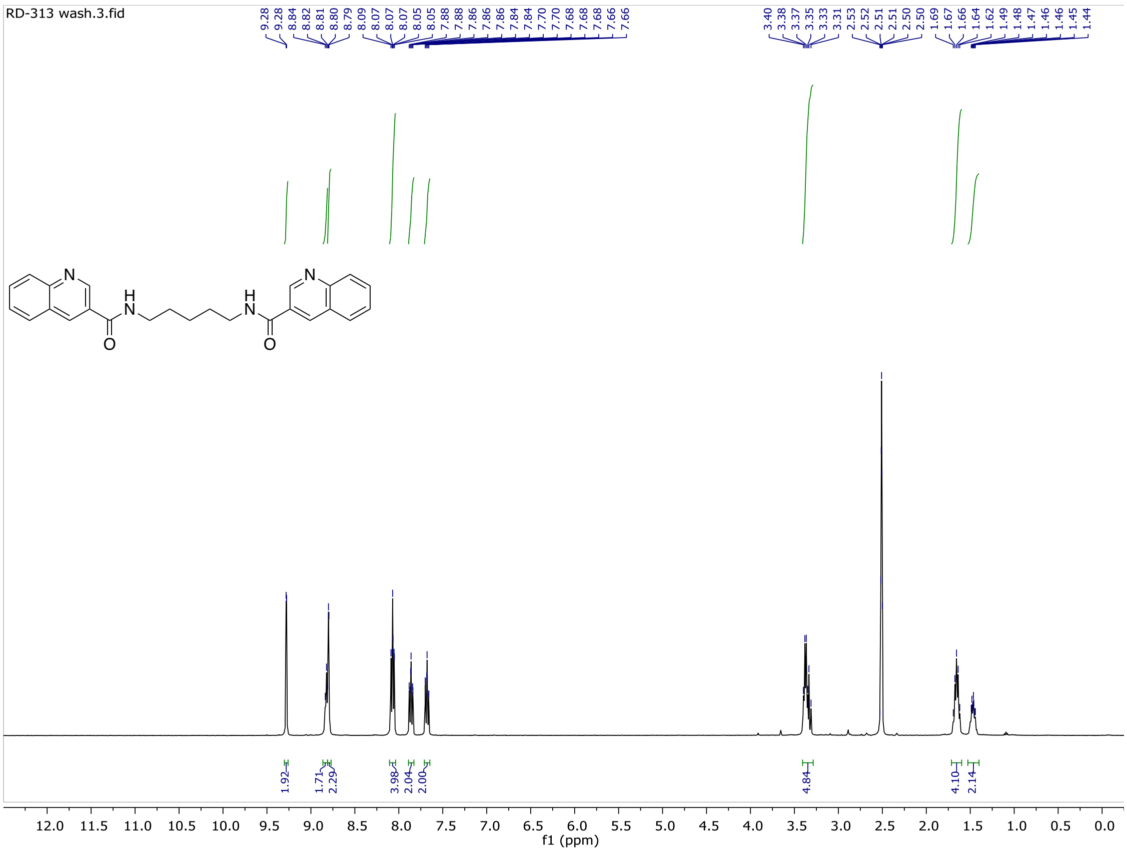

^{1}H NMR spectrum of **N,N'-(pentane-1,5-diyl)bis(quinoline-3-carboxamide** measured in DMSO-d_6 at 400 MHz.

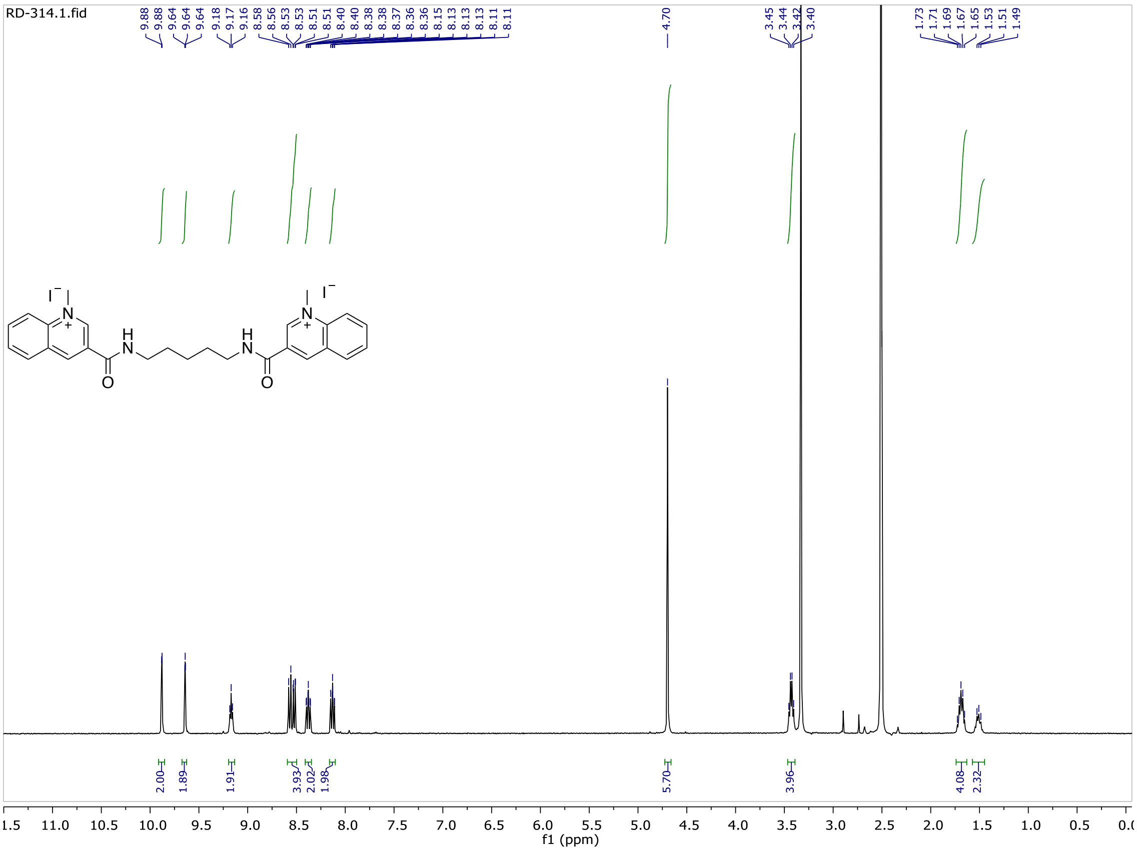

^{1}H NMR spectrum of **16** measured in DMSO-d_6 at 400 MHz.

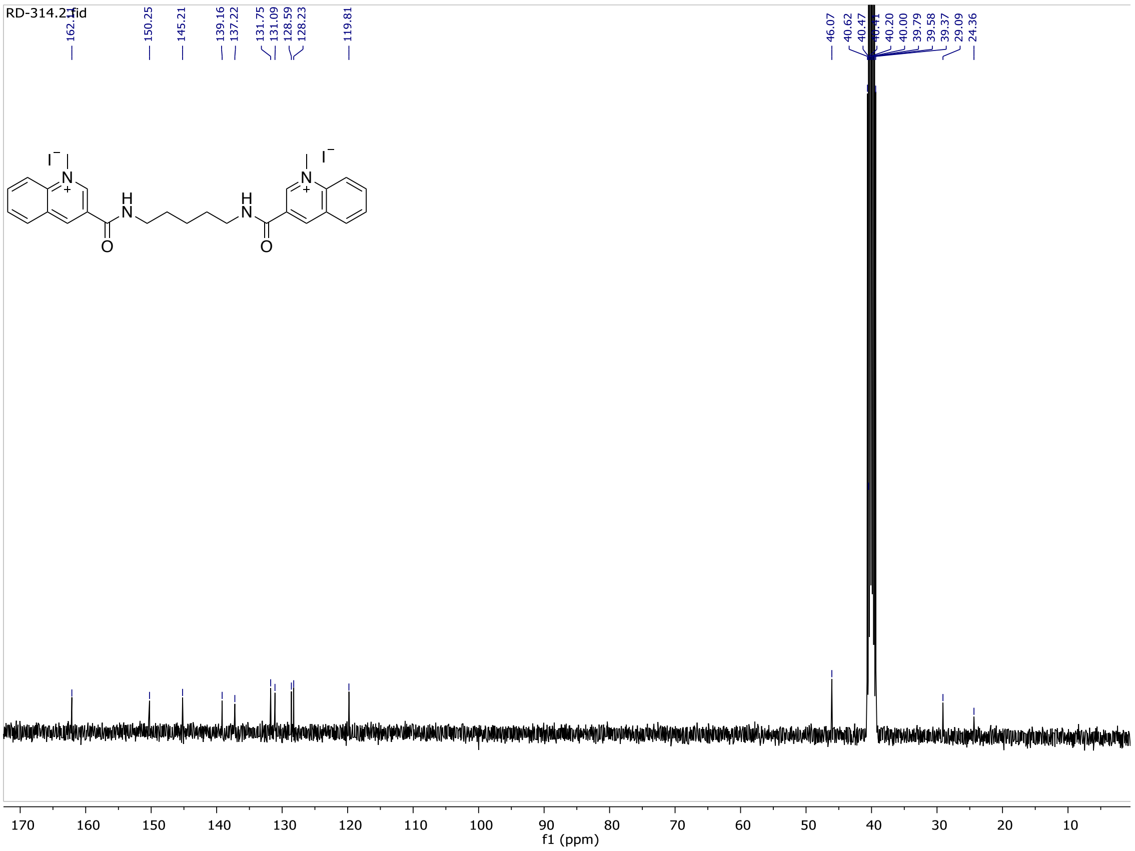

^{13}C NMR spectrum of **16** measured in DMSO-d_6 at 100 MHz.

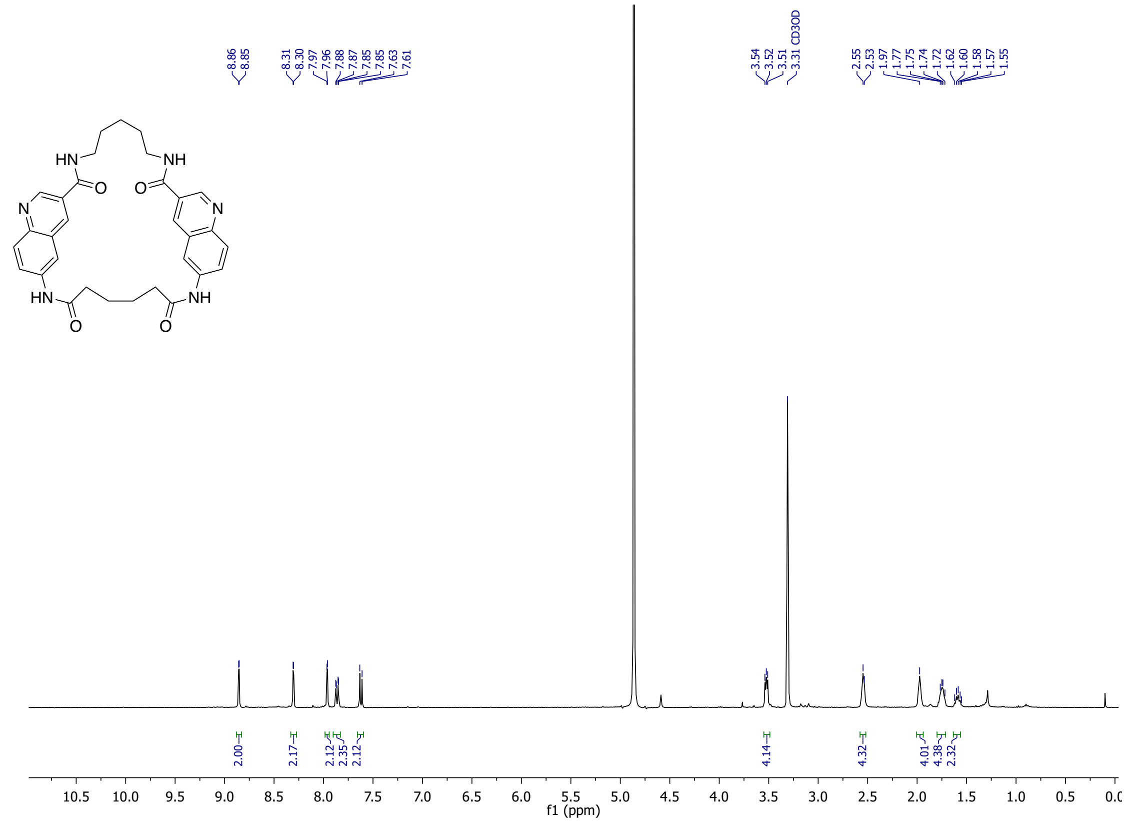

^{1}H NMR spectrum of **2,9,12,18-tetraaza-1,10(6,3)-diquinolinacyclononadecaphane-3,8,11,19-tetraone** measured in CD_3OD at 400 MHz.

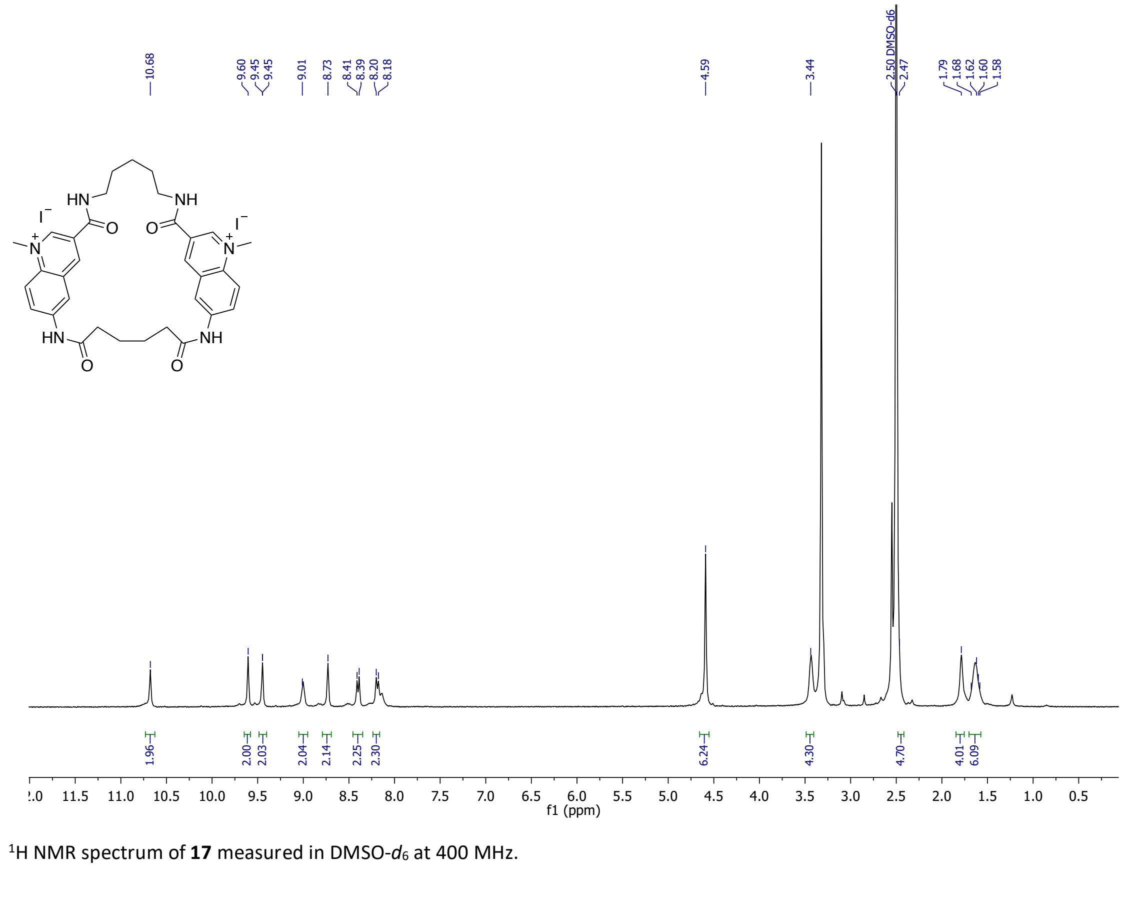

^{1}H NMR spectrum of **17** measured in DMSO-d_6 at 400 MHz.

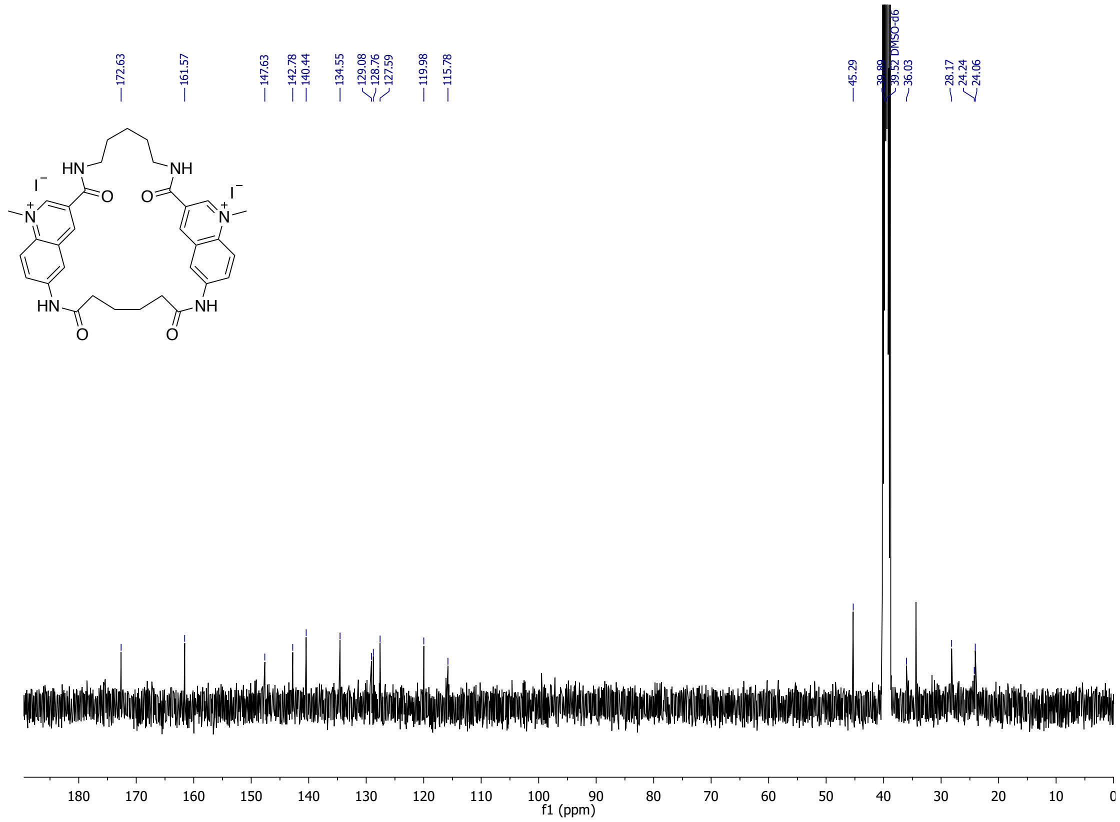

^{13}C NMR spectrum of **17** measured in DMSO-d_6 at 100 MHz.

References

[1] W. J. Chung, B. Heddi, F. Hamon, M. P. Teulade-Fichou and P. Anh Tuan, *Angew. Chem. Int. Ed.,* 2014, **53**, 999.
[2] M. D. Hanwell, D. E. Curtis, D. C. Lonie, T. Vandermeersch, E. Zurek and G. R. Hutchison, *J. Cheminform.,* 2012, **4**, 17.
[3] Pettersen *et al. J. Comput. Chem.,* 2004, **25**, 1605.
[4] M. J. Abraham, T. Murtola, R. Schulz, S. Páll, J. C. Smith, B. Hess and E. Lindahl, *SoftwareX,* 2015, **1–2**, 19.
[5] V. Hornak, R. Abel, A. Okur, B. Strockbine, A. Roitberg and C. Simmerling, *Proteins,* 2006, **65**, 712.
[6] I. Ivani, P. D. Dans, A. Noy, A. Perez, I. Faustino, A. Hospital, J. Walther, P. Andrio, R. Goni, A. Balaceanu, G. Portella, F. Battistini, J. L. Gelpi, C. Gonzalez, M. Vendruscolo, C. A. Laughton, S. A. Harris, D. A. Case and M. Orozco, *Nat. Methods*, 2016, **13**, 55.
[7] W. L. Jorgensen, J. Chandrasekhar, J. D. Madura, R. W. Impey and M. L. Klein, *J. Chem. Phys.*, 1983, **79**, 926.
[8] L. X. Dang, *J. Am. Chem. Soc.*, 1995, **117**, 6954.
[9] Gaussian 16, M. J. Frisch, G. W. Trucks, H. B. Schlegel, G. E. Scuseria, M. A. Robb, J. R. Cheeseman, G. Scalmani, V. Barone, G. A. Petersson, H. Nakatsuji, X. Li, M. Caricato, A. V. Marenich, J. Bloino, B. G. Janesko, R. Gomperts, B. Mennucci, H. P. Hratchian, J. V. Ortiz, A. F. Izmaylov, J. L. Sonnenberg, Williams, F. Ding, F. Lipparini, F. Egidi, J. Goings, B. Peng, A. Petrone, T. Henderson, D. Ranasinghe, V. G. Zakrzewski, J. Gao, N. Rega, G. Zheng, W. Liang, M. Hada, M. Ehara, K. Toyota, R. Fukuda, J. Hasegawa, M. Ishida, T. Nakajima, Y. Honda, O. Kitao, H. Nakai, T. Vreven, K. Throssell, J. A. Montgomery Jr., J. E. Peralta, F. Ogliaro, M. J. Bearpark, J. J. Heyd, E. N. Brothers, K. N. Kudin, V. N. Staroverov, T. A. Keith, R. Kobayashi, J. Normand, K. Raghavachari, A. P. Rendell, J. C. Burant, S. S. Iyengar, J. Tomasi, M. Cossi, J. M. Millam, M. Klene, C. Adamo, R. Cammi, J. W. Ochterski, R. L. Martin, K. Morokuma, O. Farkas, J. B. Foresman and D. J. Fox, *Wallingford, CT*, 2016.
[10] D. A. Case, D. S. Cerutti, I. T. E. Cheatham, T. A. Darden, R. E. Duke, T. J. Giese, H. Gohlke, A. W. Goetz, D. Greene, N. Homeyer, S. Izadi, A. Kovalenko, S. T. S. Lee, P. LeGrand, Li, C. Lin, J. Liu, T. Luchko, R. Luo, D. Mermelstein, K. M. Merz, G. Monard, H. Nguyen, I. Omelyan, A. Onufriev, F. Pan, R. Qi, D. R. Roe, A. Roitberg, C. Sagui, C. L. Simmerling, W. M. Botello-Smith, J. Swails, R. C. Walker, J. Wang, R. M. Wolf, X. Wu, L. Xiao, D. M. York and P. A. Kollman, *AMBER, University of California, San Francisco*, 2017.

[11] A. W. Sousa da Silva and W. F. Vranken, *BMC Res. Notes*, 2012, **5**, 367.
[12] P. Bagineni, J. Jamroskovic, S. Bhowmik, R. Kumar, T. Romell, N. Sabouri and E. Chorell, *Chem. Eur. J.,* 2018, **24**, 7926.
[13] N. A. Baker, D. Sept, S. Joseph, M. J. Holst, J. A. McCammon, *Proc. Natl. Acad. Sci. U. S. A*., 2001, **98**, 10037.
[14] R. Kumari, R. Kumar, C. Open Source Drug Discovery, Lynn, A. *J. Chem. Inf. Model*., 2014, **54**, 1951.
[15] W. Humphrey, A. Dalke and K. Schulten, *J. Mol. Graph*., 1996, **14**, 33.
[16] K. Sakamoto, S. Yoshino, M. Takemoto and N. Furuya, *J. Porphyrins Phthalocyanines*, 2013, **17**, 605.
[17] J. Rousseau, Z. Zhang, G. M. Dias, C. Zhang, N. Colpo, F. Bénard and K. S. Lin, *Bioorganic & Medicinal Chemistry Letters*, 2017, **27**, 708.
